# Developmental plasticity in thermal tolerance: Ontogenetic variation, persistence, and future directions

**DOI:** 10.1111/ele.14083

**Published:** 2022-08-25

**Authors:** Patrice Pottier, Samantha Burke, Rose Y. Zhang, Daniel W. A. Noble, Lisa E. Schwanz, Szymon M. Drobniak, Shinichi Nakagawa

**Affiliations:** ^1^ Evolution & Ecology Research Centre, School of Biological Earth and Environmental Sciences, The University of New South Wales Sydney New South Wales Australia; ^2^ Division of Ecology and Evolution Research School of Biology, College of Science, The Australian National University Canberra Australian Capital Territory Australia; ^3^ Institute of Environmental Sciences Jagiellonian University Kraków Poland

**Keywords:** acclimation, climate vulnerability, critical thermal limits, CT_max_, heat tolerance, ontogenetic variation, persistence, phenotypic plasticity, reversibility, systematic review

## Abstract

Understanding the factors affecting thermal tolerance is crucial for predicting the impact climate change will have on ectotherms. However, the role developmental plasticity plays in allowing populations to cope with thermal extremes is poorly understood. Here, we meta‐analyse how thermal tolerance is initially and persistently impacted by early (embryonic and juvenile) thermal environments by using data from 150 experimental studies on 138 ectothermic species. Thermal tolerance only increased by 0.13°C per 1°C change in developmental temperature and substantial variation in plasticity (~36%) was the result of shared evolutionary history and species ecology. Aquatic ectotherms were more than three times as plastic as terrestrial ectotherms. Notably, embryos expressed weaker but more heterogenous plasticity than older life stages, with numerous responses appearing as non‐adaptive. While developmental temperatures did not have persistent effects on thermal tolerance overall, persistent effects were vastly under‐studied, and their direction and magnitude varied with ontogeny. Embryonic stages may represent a critical window of vulnerability to changing environments and we urge researchers to consider early life stages when assessing the climate vulnerability of ectotherms. Overall, our synthesis suggests that developmental changes in thermal tolerance rarely reach levels of perfect compensation and may provide limited benefit in changing environments.

## Graphical abstract

Understanding the role developmental plasticity plays in allowing populations to cope with thermal extremes is critical. Here, we meta‐analysed how thermal tolerance is impacted by early thermal environments using data from 138 ectothermic species. We found that developmental changes in thermal tolerance are not sufficient to buffer ectotherms from rising temperatures and that embryonic stages may represent a critical window of vulnerability to changing environments.

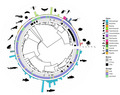

## INTRODUCTION

Ectotherms represent most of the animal diversity on the planet (Zhang, 2013), yet they are particularly vulnerable to extreme heat events (Angilletta, 2009). Extreme heat events are predicted to become fourteen times more likely to occur, and to generate temperatures 2.7°C higher by 2100 relative to the previous century (Arias et al., 2021). As such, it is crucial to understand how ectotherms will adjust to rapidly changing temperatures in the future (Chevin et al., 2010; Hoffmann & Sgrò, 2011; Noble et al., 2019; Seebacher et al., 2015). While genetic adaptation is a key mechanism by which populations can adapt, it can be slow and is constrained by genetic (co)variation (Chevin et al., 2010). Instead, phenotypic changes within an animal's lifetime (i.e., phenotypic plasticity) may be a more effective mechanism to cope with abrupt environmental changes—allowing ectotherms to withstand extreme heat events for longer, buying time for evolutionary rescue to occur (Bush et al., 2016; Morley et al., 2019). Thermal tolerance is a key trait permitting organisms to deal with thermal stress and is known to respond to the environment plastically (Gunderson et al., 2017; Gunderson & Stillman, 2015; Morley et al., 2019; Pottier, Burke, Drobniak et al., 2021; Rohr et al., 2018). Thermal tolerance traits (e.g., CT_max_, CT_min_) can be used to understand how species distributions will be impacted by climate change (e.g., Sunday et al., 2012, 2014; Comte & Olden, 2017; Pinsky et al., 2019). Nonetheless, broad‐scale ecophysiological models rarely account for plasticity in thermal tolerance (Bush et al., 2016; Huey et al., 2012). In addition, most syntheses examining plasticity in thermal tolerance have not assessed whether embryonic, juvenile and adult stages differ in the extent of their plasticity (Bodensteiner et al., 2021).

Early life stages, however, are crucial periods during development that are often the most impacted by temperature (Bodensteiner et al., 2021; Fawcett & Frankenhuis, 2015; Noble et al., 2018; O'Dea et al., 2019; Refsnider et al., 2019; Truebano et al., 2018; Turriago et al., 2015; While et al., 2018). Neglecting how early (embryonic and/or juvenile) environmental experiences shape thermal tolerance (i.e., developmental plasticity) may be an important oversight given that early life experiences have major and often long‐lasting effects on phenotypes (Bodensteiner et al., 2021; Noble et al., 2018; O'Dea et al., 2019; Refsnider et al., 2019; While et al., 2018). Importantly, examining whether thermal tolerance is persistently shaped by early thermal environments has critical implications for ecophysiological modelling and experimental research. In fact, experimental studies often assume that laboratory acclimation erase the effects of thermal history (Kellermann et al., 2017) and that adult plasticity does not vary with early thermal conditions (Beaman et al., 2016; Healy et al., 2019; Kellermann & Sgrò, 2018). However, early life stages are predicted to differ in their levels of plasticity relative to adults because these stages often coincide with limited mobility—forcing organisms to cope with the environmental conditions in which they settle. Without resort to behavioural thermoregulation, selection for more plastic responses may occur disproportionately in early life stages relative to adults (Bodensteiner et al., 2021; Muñoz, 2021; but see Mitchell et al., 2013). In addition, plastic responses are expected to be costly (DeWitt et al., 1998, but see Murren et al., 2015). As such, the self‐reliance of early life stages on endogenous energy reserves and the costs imposed by developmental processes (Marshall et al., 2020; Pettersen et al., 2018) may constrain the allocation of energy to diverse functions, including plastic responses to temperatures.

Taken together, weaker plastic responses are expected in early life stages if energy allocation trade‐offs have a predominant role, whereas selection for stronger plasticity could occur due to limited thermoregulatory abilities. Importantly, the interplay between basal thermal tolerance and plasticity throughout ontogeny is essential to consider in broad‐scale quantifications of climate vulnerability (Ruthsatz et al., 2022). Ontogenetic variation in absolute thermal tolerance may be mitigated or further exacerbated by varying levels of plasticity throughout the life cycle. For instance, if the lower thermal tolerance of embryos (Truebano et al., 2018; Dahlke et al., 2020; but see Pottier, Burke, Drobniak, et al., 2022 and Dahlke et al., 2022) is associated with low plasticity, then this life stage may be the most sensitive to abrupt climate change. Therefore, it is crucial to investigate whether early life stages can acclimate to new temperatures (i.e., initial effects), whether those responses persist (i.e., persistent effects), and how the magnitude of plastic responses to temperatures vary with ontogeny. Yet, no study has systematically addressed those questions across ectothermic species. A meta‐analysis of the published experimental data could help delineate the initial and persistent effects of developmental temperatures on thermal tolerance, as well as explaining the heterogeneity across studies. For instance, a meta‐analysis may resolve discrepancies between studies by increasing statistical power (Duffy et al., 2021) and highlighting potential differences between species based on their ecology, evolutionary history, or differences in experimental methodology (Gurevitch et al., 2018).

Here, we synthesize the current evidence to quantify the magnitude and variability of developmental plasticity in heat tolerance across ectotherms, using a meta‐analysis of the experimental literature. We hypothesised that, overall, early‐life stages acclimated to higher temperatures would be more heat tolerant than animals acclimated to lower temperatures, reflecting similar patterns as in adult animals (e.g., Gunderson & Stillman, 2015). We also hypothesised that the levels of developmental plasticity would vary with ontogeny. Specifically, we hypothesised that temperatures experienced during embryonic development would have stronger influence on heat tolerance than during juvenile development because the limited ability for embryos to thermoregulate behaviourally may have selected for greater plastic responses. In addition, manipulating the temperature of both embryonic and juvenile development may increase an animal's plasticity by spanning various developmental windows of sensitivity to temperatures. Alternatively, we predicted the opposite pattern if juveniles can invest additional energy into physiological regulation via feeding. Indeed, the reliance of embryos on endogenous energy reserves might constrain the resource investment into plastic responses. For all life stages, we hypothesised that the effects of developmental temperatures on heat tolerance will persist throughout the life of the animals. However, the magnitude of persistent responses should decline as animals are re‐acclimated to common garden conditions for extended periods after the initial acclimation.

We also hypothesised that the developmental plasticity of ectotherms will vary based on their ecology. Because terrestrial habitats tend to have a greater seasonal and daily temperature variability than aquatic habitats, we predicted terrestrial animals to be more developmentally plastic than their aquatic counterparts as greater seasonality may select for greater plastic responses (Janzen, 1967; Ghalambor et al., 2006; Chevin & Hoffmann, 2017). Alternatively, because changes in water temperature result in faster changes in body temperature (Angilletta, 2009; Denny, 1993), plastic responses may be more strongly selected in aquatic taxa because of increased exposure to temperature variability (Chevin & Hoffmann, 2017). Finally, we investigated sources of methodological variation such as differences in thermal tolerance metrics (i.e., LT_50_ or CT_max_) and assay heating rates.

## MATERIALS AND METHODS

### Protocol, registration, and reporting

We preregistered our predictions (see introduction), methods and planned analyses prior to data extraction and analysis (https://osf.io/zkx6u; Pottier, Burke, Zhang, et al., 2021). We followed the PRISMA‐EcoEvo (Preferred Reporting Items for Systematic reviews & Meta‐Analyses in Ecology and Evolutionary biology; O'Dea et al., 2021) guidelines for reporting this study (Table S4). Data, code, and additional resources are available at https://github.com/p‐pottier/Dev_plasticity_thermal_tolerance (Pottier, Burke, Zhang, et al., 2022).

### Literature searches and study selection

We aimed to obtain a relatively comprehensive and representative sample of the experimental literature (published or unpublished) testing for the developmental plasticity of heat tolerance in ectotherms. We accessed Scopus, ISI Web of Science (core collection), and ProQuest (dissertations & theses) on 2021/03/05 and did not apply a timespan limit. Search strings were tailored to each database (full search strings are presented in Supporting Information S1; supplementary methods) to capture studies manipulating developmental temperatures of ectothermic animals, and subsequently measuring their heat tolerance. In addition to database searches, we performed backward searches in Scopus to search for relevant studies citing four influential publications (Schaefer and Ryan (2006); and Bodensteiner et al., 2021, Bowler & Terblanche, 2008 and Refsnider et al., 2019). We also included studies testing CT_max_ in Table I and Table II of Bodensteiner et al. (2021) and Refsnider et al. (2019), respectively. Finally, we included all studies cited in Bowler and Terblanche (2008) but did not perform a forward search from Schaefer and Ryan (2006) because it was not a literature review.

**FIGURE 1:**
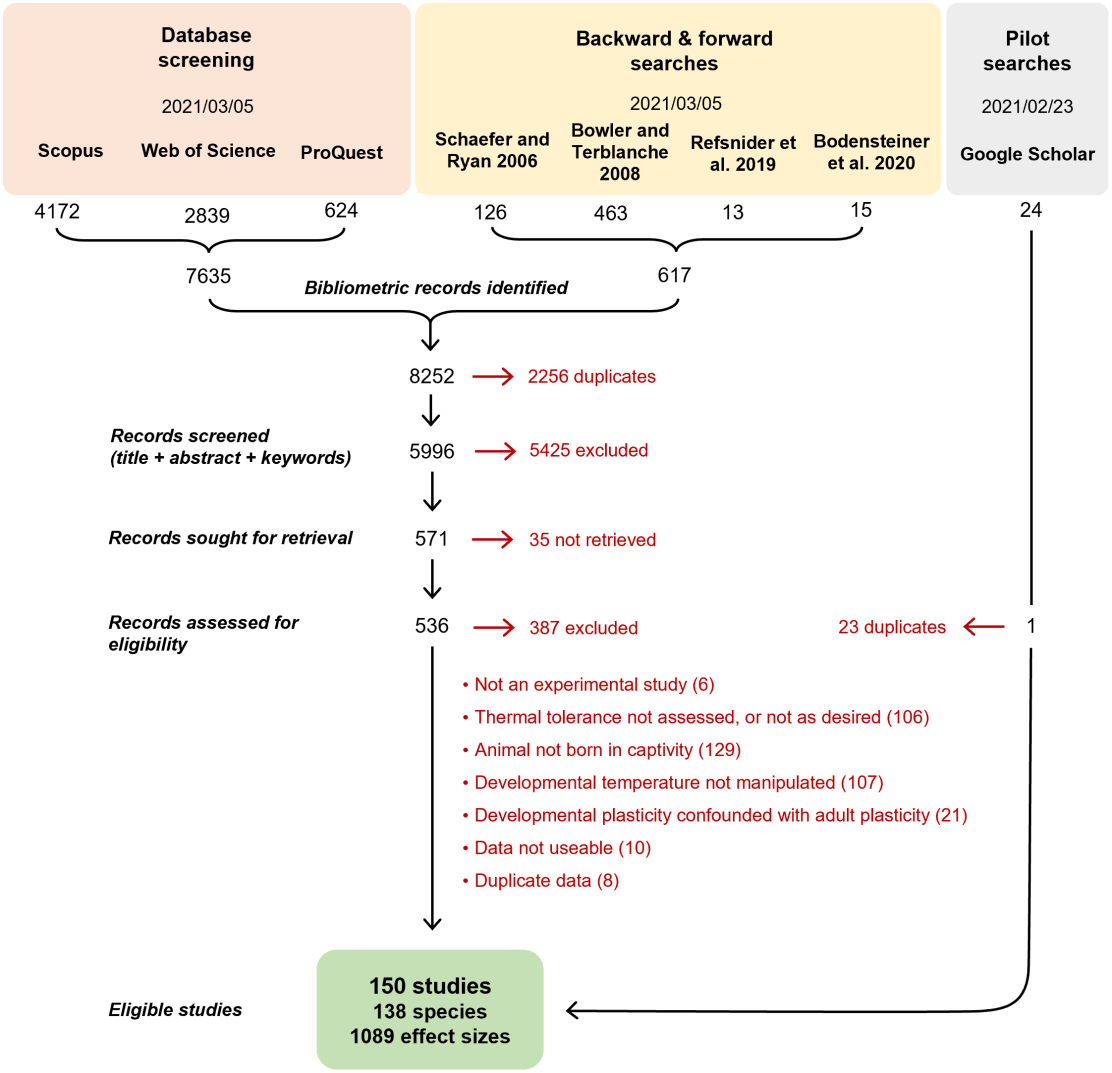
PRISMA flow chart summarizing the search methods, the number of studies excluded, and the reasons for exclusion.

Our searches found 5996 unique documents. Titles, abstracts, and keywords were screened by PP (90%), SB (5%) and RZ (5%) in Rayyan QCRI (Ouzzani et al., 2016). A total of 571 documents were further assessed for eligibility by PP. Thirty‐five documents were not accessible to the authors, and 32 studies were missing descriptive statistics for their direct inclusion in the meta‐analysis (mean, sample size, and measure of dispersion). We contacted the authors of the original studies to request missing information if the study was published after 1995. We imputed missing standard deviations when authors did not respond but we could not impute missing standard errors (see *Data extraction and effect size calculation*). One study (Cheung, 2019) was found to be eligible during pilot searches using Google Scholar (i.e., benchmarking, sensu Foo et al., 2021), but was not captured by our search methods. Search methods are summarized in our PRISMA flowchart (Figure 1), and included studies are listed in the *Data sources* section.

### Eligibility criteria

We focused on studies that chronically manipulated the developmental (embryonic or juvenile) temperature of ectothermic animals, and subsequently measured their heat tolerance. We selected studies based on seven eligibility criteria (Figures S1, S2). First, we only included studies on ectothermic animals. Second, we focused our study on manipulative laboratory experiments. Third, we only considered studies using standard and ecologically‐relevant measures of heat tolerance (Terblanche et al., 2011). Eligible heat tolerance metrics were (i) the critical thermal maximum (CT_max_), where temperature is incrementally increased until animals reach an endpoint (dynamic method; Lutterschmidt & Hutchison, 2011), and (ii) the temperature lethal for 50% of the animals (LT_50_), where animals are subjected to constant temperatures and their survival is measured after a given period (static method; Fry, 1947). We also considered studies measuring the time to death (or heat knockdown) at different static temperatures because these measures can be converted to CT_max_ using regression approaches (see Rezende et al., 2014; Jørgensen et al., 2019, 2021). To increase the comparability of our estimates, we excluded alternative proxies for heat tolerance such as heat knockdown recovery time, or extrapolations from physiological performance curves. Fourth, we only included studies where animals experienced controlled early thermal environments during their embryonic development. Therefore, only data from animals born in captivity were included. Fifth, we included studies using ≥2 developmental constant or fluctuating temperatures differing by their mean and controlled in a laboratory setting. Fluctuating treatments were included provided they were comparable (i.e., differing by their mean, but having a comparable fluctuation). Sixth, we considered any prolonged (≥ 24 h) temperature experienced during the embryonic or juvenile stage as a relevant manipulation of developmental temperature. Hence, we excluded studies solely acclimating adult animals. We also excluded studies where developmental plasticity was confounded with adult acclimation. In other words, adult measures of heat tolerance must have been performed on adults acclimated to the same temperature but differing by their developmental thermal history. For logistical reasons, the developmental thermal exposure may have been continued for hours after the transition to adulthood in some studies (e.g., emergence from pupa). We tolerated such an overlap with adult acclimation when thermal conditions experienced by adults was ≤24 h. Ectotherms can take days to acclimate to new temperatures (e.g., Layne & Claussen, 1982) and 24 h was chosen as cut‐off to separate acclimatory responses from passive physiological plasticity or responses to heat shocks. Decision trees and further details about our inclusion criteria are presented in Figures S1, S2 and Tables S1, S2.

### Data extraction and effect size calculations

We extracted mean heat tolerance for developmental temperature along with associated sample sizes and measures of dispersion (i.e., standard deviations and standard errors). Data extractions were performed by PP (72.8%), SB (13.6%) and RZ (13.6%) and all data were further checked for accuracy by PP. Data presented in the text or tables were directly extracted from the study. Data from figures were digitized using the *metaDigitise* package in R (Pick et al., 2019; version 1.0.1). Means, standard deviations and sample sizes were estimated from the raw data when available. When data were presented in different sources, we prioritized the source having the finest resolution. For studies measuring the time to death (or heat knockdown) at different static temperatures, we performed a linear regression of the logarithm of the time to death against the test temperatures and estimated the temperature the animals could tolerate for 1 h as a proxy for CT_max_ (following Jørgensen et al., 2019, 2021). We did not use the temperature the animals could tolerate for 1 min because extrapolations beyond thermal death time curves provide less accurate estimates than interpolations of the data (Jørgensen et al., 2019, 2021). In addition to heat tolerance data, we extracted information required to address our a priori hypotheses (see *Introduction*). We also collected additional data from the studies, such as the origin of the animals, their body mass, body length, sex, or details about the heat tolerance methodology.

We defined our effect size as the developmental acclimation response ratio (*dARR*), which is analogous to the acclimation response ratio (ARR; Claussen, 1977). Such a metric defines the variation in heat tolerance associated with a one‐degree change in developmental temperature. For instance, a dARR of 0.6 indicates that each degree increase in developmental temperature increases the heat tolerance by 0.6°C. This effect size has the advantage of accounting for the magnitude of temperature difference between the temperature treatments compared (controlling for the “nuisance heterogeneity” sensu Noble et al., 2022).

We defined our effect size as:

(1) $$\text{dARR}=\frac{{\mathrm{HT}}_{\left[T2\right]}-{\mathrm{HT}}_{\left[T1\right]}}{T_{2}-T_{1}},$$

where *T* represents the developmental temperature in Celsius (with *T*_*2*_ > *T*_*1*_), and *HT* the heat tolerance estimates in Celsius. When data on >2 developmental temperatures were presented, we calculated dARR for each stepwise comparison (e.g., 20–22°C, 22–25°C, 25–27°C). The sampling variance for this effect size was derived as per Equation 2 (derived from Pottier, Burke, Drobniak et al., 2021):

(2) $$s^{2}\left(\text{dARR}\right)={\left(\frac{1}{T_{2}-T_{1}}\right)}^{2}\left(\frac{{{\mathrm{sd}}^{2}}_{\left[T1\right]}}{n_{\left[T1\right]}}+\frac{{{\mathrm{sd}}^{2}}_{\left[T2\right]}}{n_{\left[T2\right]}}\right),$$

where *s* ^*2*^ *(dARR)* is the sampling variance of *dARR*, *sd* is the standard deviation and *n* is the sample size (number of individuals). In cases where sample sizes were unknown and only standard errors were presented, the sampling variance of *dARR* was calculated as per Equation 3.

(3) $$s^{2}\left(\text{dARR}\right)={\left(\frac{1}{T_{2}-T_{1}}\right)}^{2}\left({{\mathrm{se}}^{2}}_{\left[T_{1}\right]}+{{\mathrm{se}}^{2}}_{\left[T_{2}\right]}\right)$$

Where *se* is the standard error.

We also included data where the same animals were measured at both *T*_*1*_ and *T*_*2*_. In this case, the sampling variance of *dARR* was calculated as Equation 4 when standard deviations were available, or Equation 5 when only standard errors were presented.

(4) $$s^{2}\left(\text{dARR}\right)={\left(\frac{1}{T_{2}-T_{1}}\right)}^{2}\left(\frac{{{\mathrm{sd}}^{2}}_{\left[T1\right]}+{{\mathrm{sd}}^{2}}_{\left[T2\right]}-2r_{\left[T1T2\right]}{\mathrm{sd}}_{\left[T1\right]}{\mathrm{sd}}_{\left[T2\right]}}{n_{\left[T1\right]}+n_{\left[T2\right]}}\right)$$

(5) $$s^{2}\left(\text{dARR}\right)={\left(\frac{1}{T_{2}-T_{1}}\right)}^{2}\left({{\mathrm{se}}^{2}}_{\left[T_{1}\right]}+{{\mathrm{se}}^{2}}_{\left[T_{2}\right]}-2r_{\left[T1T2\right]}{\mathrm{se}}_{\left[T1\right]}{\mathrm{se}}_{\left[T2\right]}\right)$$

Where *r*_*[T1T2]*_ was taken as 0.5 as a conservative measure (Noble et al., 2017).

The sampling variance for our effect size requires knowledge about the uncertainty around mean estimates (Equations (2), (3), (4), (5)). Therefore, for effect sizes missing standard deviations, we inferred standard deviations using within‐study imputation (Equation  6; Lajeunesse et al., 2013), where the standard deviation to mean ratio was deemed constant across studies. 
(6)

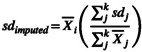

where *sd* is the standard deviation, ${\bar{X}}_{i}$ is the mean heat tolerance of the sample, *j* is the study, *k* is the total number of studies, and ${\bar{X}}_{j}$ is the mean heat tolerance of the study. Assessments of the accuracy of these imputations and their impact on our analyses are described in *Sensitivity Analyses*.

### Meta‐analysis and meta‐regressions

We performed all statistical analyses in R version 4.1.0 (R Core Team, 2019). We used multi‐level meta‐analytic models using the *rma.mv* function in the *metafor* package (Viechtbauer, 2010; version 3.0.2). Test statistics and confidence intervals for the fixed effects were computed using *t* distributions.

Our data had various sources of non‐independence (Noble et al., 2017). Multiple effect sizes were collected from the same studies (study ID), some species and populations were represented multiple times (species ID and population ID, respectively), species had different levels of phylogenetic relatedness (phylogeny), some animals in different treatments originated from the same parents (family ID), the same data were re‐used in stepwise comparisons when calculating effect sizes (e.g., dARR for groups acclimated to 20–22°C, 22–25°C, 25–27°C; shared treatment ID), and repeated measures were collected on the same group of animals (e.g. 24 h‐LT_50_ and 48 h‐LT_50_ measured on the same cohorts; cohort ID).

Family and population ID were confounded, as such, we only included population ID in our models. Similarly, species and study ID were not distinguishable given so few studies had multiple species. As such, we only kept species ID in the models to partition phylogenetic and non‐phylogenetic species effects (Cinar et al., 2022). We inferred phylogenetic relatedness from a phylogenetic tree constructed from the Open Tree of Life using the *rotl* package (Michonneau et al., 2016; version 3.0.11). We computed branch lengths using Grafen's method and modelled phylogeny as a correlation matrix using the *ape* package (Paradis & Schliep, 2019; version 5.5). Polytomies were resolved at random, and one species, *Villosa delumbis*, was manually added to the tree based on information from the *Integrated Taxonomic Information System* (https://itis.gov). Non‐independence arising from the same cohorts was controlled using Equations 4 and 5. Finally, sampling errors from treatments involved in multiple comparisons were correlated (using a conservative *r* = 0.5) with a variance covariance matrix using the *metaAidR* package (“github.com/daniel1noble/metaAidR”; version 0.0.0.9000). To decide on the random effect structure of the models, we first fitted all non‐overlapping random variables (species ID, population ID, and phylogeny) and an observation‐level random effect (effect size ID) in a meta‐analytic (intercept‐only) model. Because population ID explained virtually no variance, it was excluded from further models.

We then estimated the overall meta‐analytic mean and the total amount of heterogeneity (i.e., variation not explained by sampling error; Senior et al., 2016), and decomposed the heterogeneity explained by the different random effect terms. Single‐moderator models were performed with each of our a priori moderators (see *Introduction*) to address our hypotheses. More complex models with multiple moderators were also built to explain the remaining heterogeneity (see Supporting Information S1; supplementary methods).

For each meta‐regression, we visually assessed assumptions of homogeneity of residual variance and used a heteroscedastic compound symmetric structure with variance components estimated for each level of a categorical variable at the effect size level (*”HCS”* structure with zero covariance from the *rma.mv* function in the *metafor* package). AIC comparisons highlighted that this approach improved model fit (Table S20).

Statistical significance was assumed when 95% confidence intervals did not overlap with zero. We presented the estimates of each moderator category but note that differences between groups (i.e., contrasts) are also presented in Supporting Information S1 (Tables S9‐19).

### Sensitivity analyses and publication bias

Publication bias refers to a higher likelihood of statistically significant findings being published than that of non‐significant findings. This bottleneck generates unrepresentative study samples and may impact the robustness of meta‐analytic results (Nakagawa et al., 2022). Publication bias was assessed in four ways. First, we used visual inspections of the relationship between model residuals and the standard error using funnel plots. We note that this method assumes that data heterogeneity is null and may not be appropriate outside of a purely visual tool (see Nakagawa et al., 2022). Second, we performed multilevel meta‐regressions using standard error or sampling variance as moderator variables to detect a small study effect, where small‐sample‐sized studies tend to have larger effect sizes (sensu Nakagawa et al., 2022). Third, we compared whether the estimates obtained from peer‐reviewed publications differed from dissertations and theses in meta‐regressions. Fourth, we assessed the time‐lag bias in our data set using a meta‐regression with publication year. The time lag bias (also known as the ‘decline effect’) refers to cases where studies with larger effects tend to be published earlier than studies with smaller effects (Koricheva & Kulinskaya, 2019).

To assess the robustness of our results, we performed five types of sensitivity analyses. First, we performed leave‐one‐out‐analyses on the meta‐analytic intercept‐only model to determine how robust results were to the exclusion of one study or one species. Second, we performed separate analyses for studies investigating the initial or persistent effects of developmental temperatures. Each moderator variable outlined above (see *Introduction*) was fitted in single‐moderator models for both data subsets. Third, we fitted a meta‐analytic model without data deemed to be acquired using unusual methods (i.e., risk of bias analysis; Tables S6, S35). Fourth, we fitted a meta‐analytic model without the effect sizes for which sampling variances were imputed. Fifth, because previous syntheses excluded effect sizes under a certain magnitude of response (e.g., excluding effect sizes < −0.15 in Gunderson & Stillman, 2015; or quantifying negative responses as null in Morley et al., 2019), we fitted meta‐analytic models without effect sizes reaching different arbitrary cut‐offs.

### Deviations from registration

While we essentially followed our original plans and procedures, we acknowledge minor deviations (details in Supporting Information S1; supplementary methods). Notably, because the distribution of the data was skewed towards aquatic animals (85.7% of effect sizes), we estimated marginal mean estimates for models assessing habitat variation in developmental plasticity. We used the package *emmeans* (Lenth et al., 2019; version 1.6.2) to obtain marginal means, where data from different habitats were given equal weights (i.e., post‐stratification sensu Gelman et al., 2020). Following recommendations at the peer‐review stage, we examined whether developmental plasticity estimates varied with body mass, age at sexual maturity and the relative time at a common temperature after the initial acclimation, i.e., the proportion of days at a common temperature relative to the age at sexual maturity. We found no evidence that the age at sexual maturity is associated with levels of developmental plasticity in heat tolerance (Table S37). Furthermore, we found no evidence for a significant influence of the (relative) time at a common temperature after the initial acclimation on (i) the magnitude of developmental plasticity, or (ii) the persistence or ontogenetic variation in the reported effects (Tables S22, S23). We also examined two‐ and three‐way interactions between latitudinal origin, body mass, ramping rate, and acclimation duration (Supporting Information S1; supplementary *methods*; Tables S38–S44). Finally, because the temperature tolerated for 1 h is not a direct proxy for CT_max_, and in fact, is more analogous to the death temperature (T_KO_, cf. Rezende et al., 2014), we demonstrated that the inclusion of the temperature tolerated for 1 h did not influence our results (Table S35).

## RESULTS

### What is the current state of knowledge?

**FIGURE 2:**
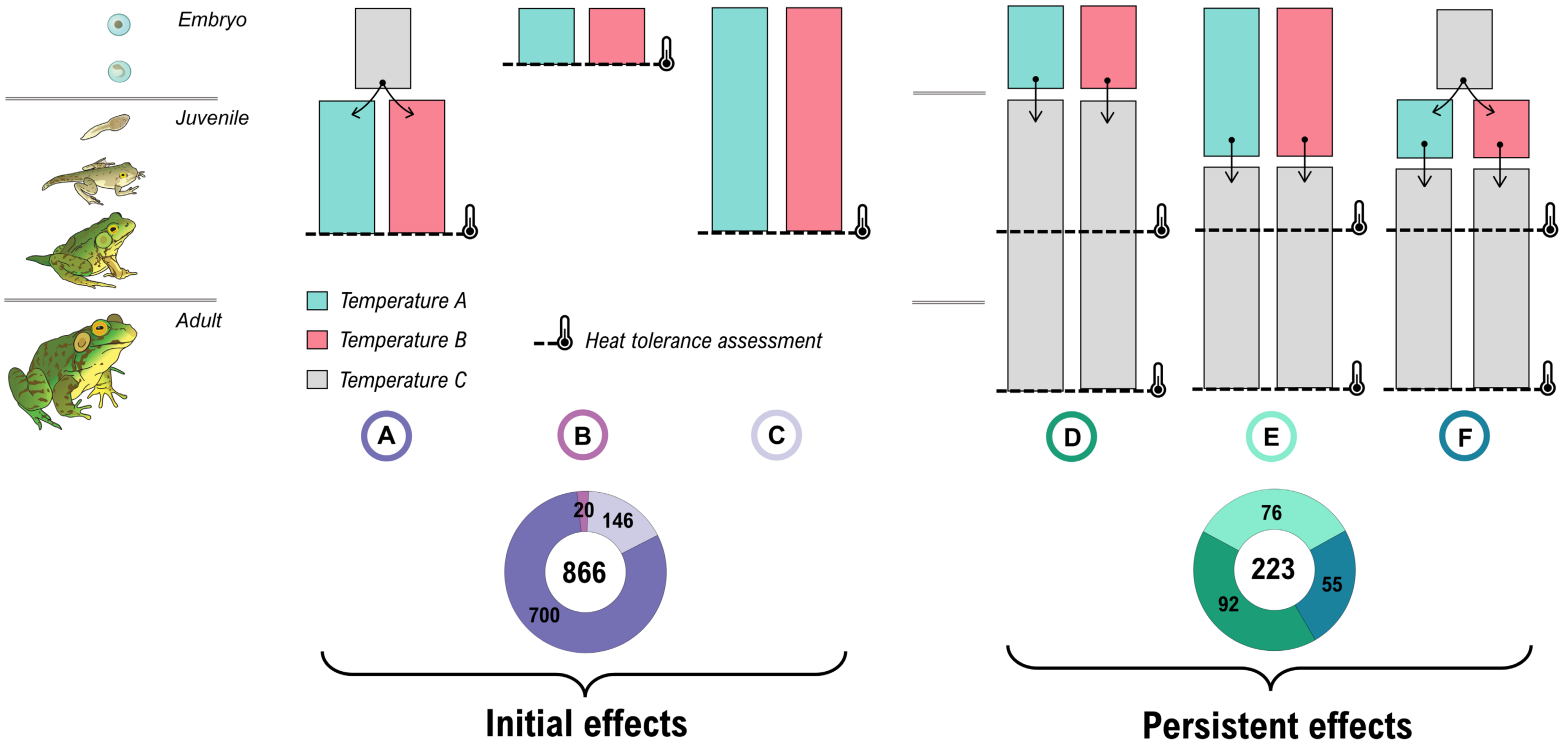
Experimental designs used to assess the developmental plasticity in heat tolerance in ectotherms. Experimental designs are grouped based on whether they assess the initial (a–c; without re‐acclimation to a common garden condition) or persistent (d–f; with re‐acclimation to a common garden condition after the initial acclimation) responses to developmental temperatures. Horizontal dashed lines represent when the heat tolerance was tested. The timing of heat tolerance measurement was positioned arbitrarily within a life history stage. In designs d, e and f, heat tolerance is assessed at either the juvenile or the adult stage following re‐acclimation, as denoted by the two heat tolerance symbols for each experimental design. Three temperatures (pink, green, grey) are presented here, but note that more temperatures can be used, and that the common temperature C can sometimes be identical to temperature a or b. Pie charts denote the number of effect sizes extracted for each type of experimental design.

**FIGURE 3:**
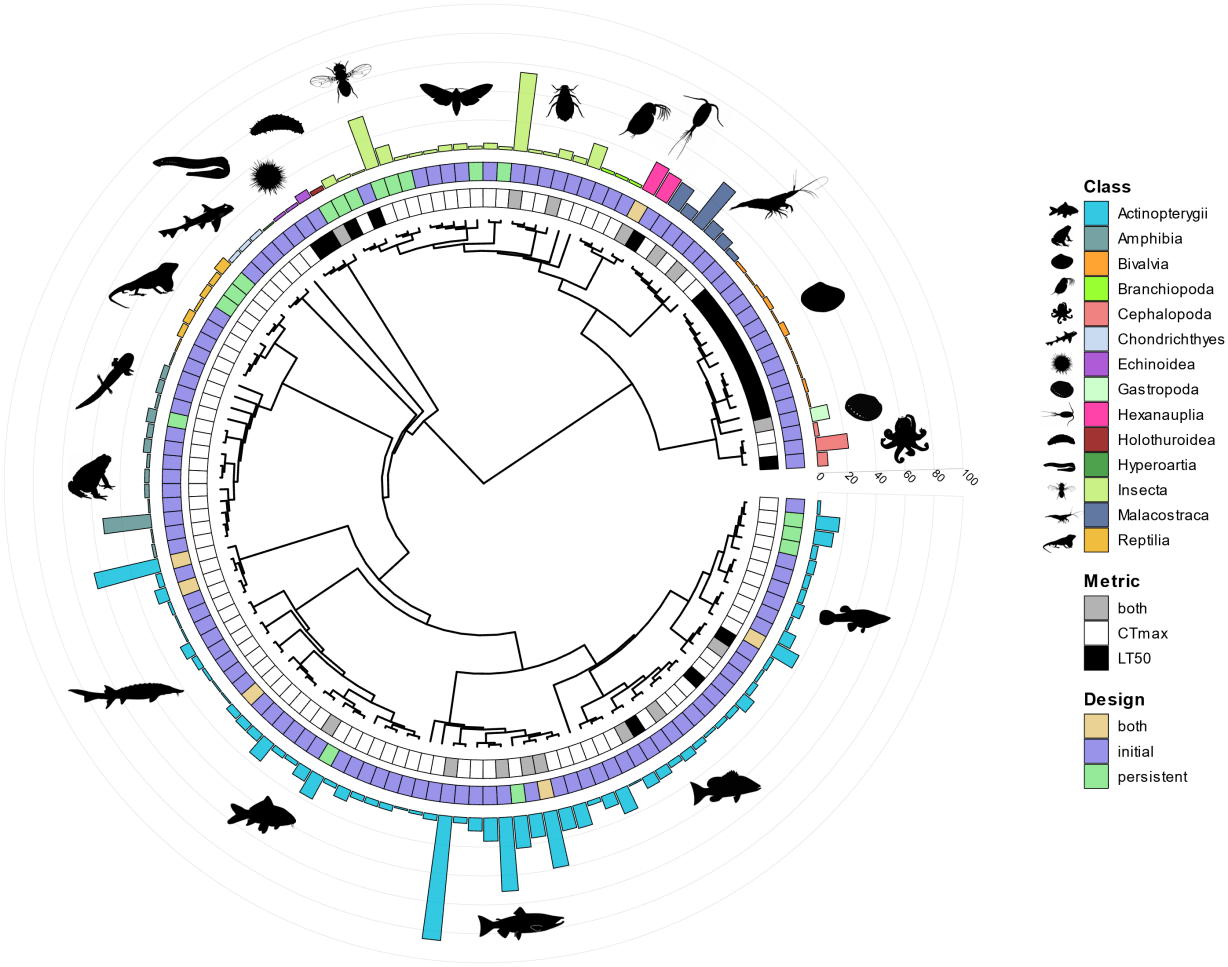
Distribution and characterization of the effect sizes across the phylogeny. The histograms represent the number of effect sizes extracted for each species. The outermost heatmap represent whether the initial or persistent effects of developmental temperatures (or both, cf. Figure 2) were assessed for this species. The innermost heatmap depicts whether the critical thermal maximum (i.e., CTmax), the median lethal temperature (i.e., LT50), or both metrics were assessed for this species. Phylogeny was constructed from the Open Tree of Life (Michonneau et al., 2016), and branch lengths were computed using Grafen's method. Silhouettes were taken from PhyloPic (www.phylopic.org).

We collected a total of 1089 effect sizes from 150 studies (1960–2021) and 138 ectothermic species. The mean (±SD) number of effect sizes per study was 7.26 ± 9.63, with a range of 1–80. Developmental plasticity in heat tolerance was tested with several experimental designs in the literature (Figure 2). We combined these designs into two broad categories: “initial” designs, where the heat tolerance was assessed immediately following the period of acclimation, and “persistent” designs, where the heat tolerance of different groups of animals was measured after a period of re‐acclimation to a common temperature after the initial acclimation (Figure 2). Overall, 79.5% of effect sizes represented “initial” effects whereas 20.5% of effect sizes represented “persistent” effects. In total, 57.2% of the effect sizes originated from fish species (Figure 3). Across papers, 79.2% of the effect sizes originated from CT_max_ data, whereas 20.8% originated from LT_50_ data (Figure 3). Further visualizations and explorations of the data are included in Supporting Information S2.

### How much do early thermal environments impact heat tolerance?

**FIGURE 4:**
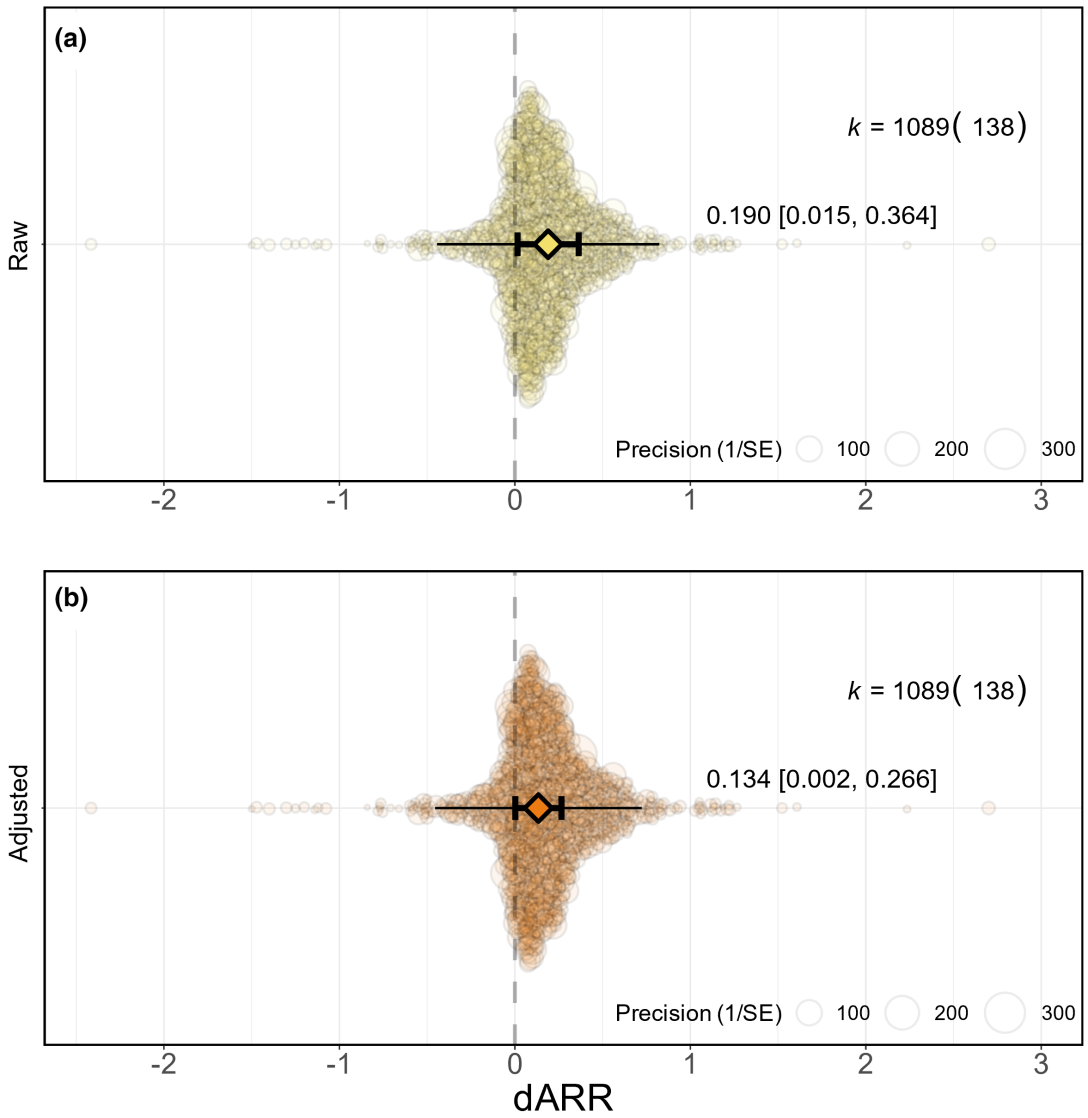
Overall level of developmental plasticity in heat tolerance. Mean meta‐analytic estimates (triangles) with their 95% confidence intervals (thicker bars with whiskers) and prediction intervals (thinner bars without whiskers) are depicted along with individual data points (coloured circles) scaled by precision (inverse of standard error). Results are presented before (a) and after (b) controlling for the over‐representation of data from aquatic vs. terrestrial animals. The graphs were constructed using the *orchaRd* package (Nakagawa et al., 2021; version 2.0). k: number of effect sizes (number of species). dARR: developmental acclimation response ratio.

Early thermal environments have a significant but weak overall effect on heat tolerance across ectotherms (dARR = 0.190; 95% confidence interval, CI = 0.015, 0.364; *n* = 1089; Figure 4). For each degree increase in developmental temperatures, heat tolerance increases by only 0.19°C. Prediction intervals (PI) suggest that 95% of the time, we expect future dARR estimates to fall between −0.444 and 0.823. Adjusting for the over‐representation of aquatic animals in our data set reduced the overall estimate even further (dARR = 0.134; 95% CI = 0.002, 0.266; 95% PI = −0.455, 0.723; *n* = 1089; Figure 4), pointing to a required 7.5°C shift in developmental temperatures to increase heat tolerance by 1°C. Despite this weak effect, heterogeneity was extremely high (*I* ^2^ = 99.5%). Overall, 26.1% of the variation was explained by shared evolutionary history, 10.0% explained by non‐phylogenetic species effects, and 63.4% of the heterogeneity associated with the residuals (i.e., within‐species heterogeneity).

### Are embryos more plastic than juveniles?

**FIGURE 5:**
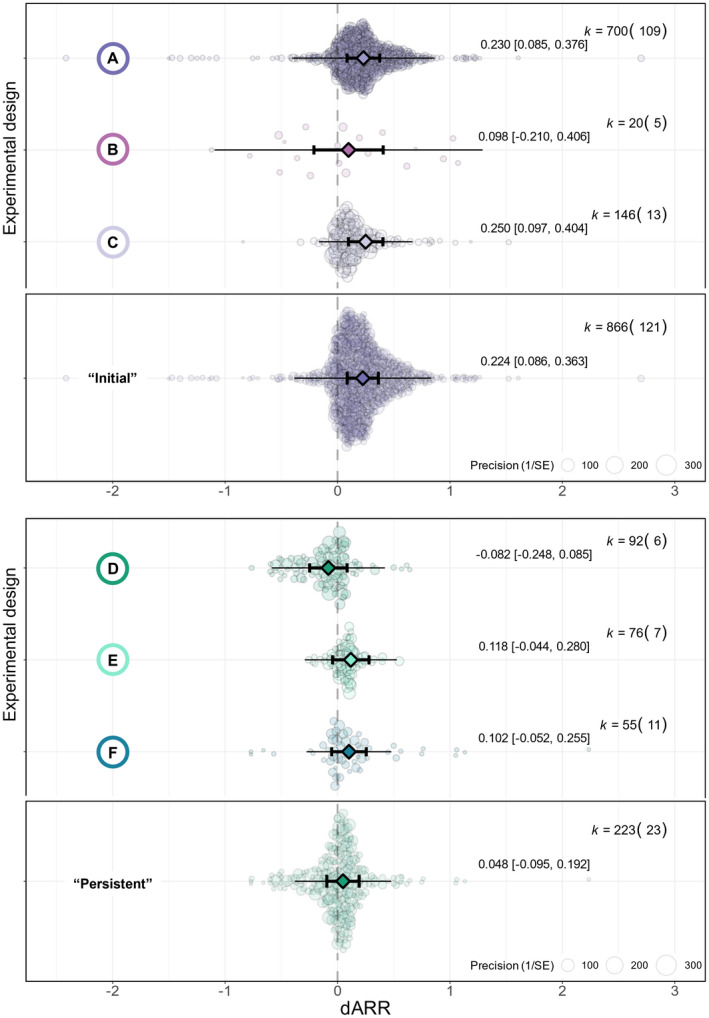
Life history variation and persistence of developmental plasticity. Mean estimates (triangles) with their 95% confidence intervals (thicker bars with whiskers) and prediction intervals (thinner bars without whiskers) are depicted along with individual data points (coloured circles) scaled by precision (inverse of standard error). k: number of effect sizes (number of species). dARR: developmental acclimation response ratio. Experimental design categorisations are presented in Figure 2.

Ectotherms were most plastic when tested immediately following temperature exposure during their juvenile (design A) or both their embryonic and juvenile development (design C) (dARR_design A_ = 0.230; 95% CI = 0.085, 0.376; 95% PI = −0.403, 0.864; *n* = 700; dARR_design C_ = 0.250; 95% CI = 0.097, 0.404; 95% PI = −0.166, 0.666; *n* = 146; *R* ^2^_marginal_ = 0.271; Figure 5). By contrast, embryos held at different temperatures (design B) barely differed in their heat tolerance levels and had highly heterogenous responses to temperature exposures (dARR_design B_ = 0.098; 95% CI = −0.210, 0.406; 95% PI = −1.093, 1.290; *n* = 20; Figure 5).

The magnitude and direction of persistent responses varied based on when in development the animals were acclimated before being re‐acclimated to a common temperature (Figure 5). Specifically, animals which experienced higher temperatures during their juvenile (design F) or both their embryonic and juvenile development (design E) and re‐acclimated to a common garden condition tended to be better at tolerating heat, albeit responses were not significantly different from zero (dARR_design E_ = 0.118; 95% CI = −0.044, 0.280; 95% PI = −0.292, 0.528; *n* = 76; dARR_design F_ = 0.102; 95% CI = −0.052, 0.255; 95% PI = −0.276, 0.479; *n* = 55). By contrast, animals incubated at different temperatures during their embryonic development and raised in a common garden condition after hatching (design D) tended to have relatively reduced heat tolerance levels, albeit not significantly (dARR_design D_ = −0.082; 95% CI = −0.248, 0.085; 95% PI = −0.585, 0.421; *n* = 92). However, we note that the distribution of those effect sizes was skewed towards negative dARR estimates—indicating that higher incubation temperatures persistently reduce the heat tolerance of ectotherms in most instances.

### Do early thermal environments have persistent impacts on thermal tolerance?

We found no overall signature for a persistent effect of early thermal environment on thermal tolerance. When animals had been returned to a common temperature after the initial developmental acclimation, the dARRs were not significantly different from zero on average, whereas animals tested immediately after acclimation to higher temperatures had higher thermal tolerance (dARR_initial_ = 0.224, 95% CI = 0.086, 0.363; 95% PI = −0.383, 0.832; *n* = 866; dARR_persistent_ = 0.049, 95% CI = −0.095, 0.192; 95% PI = −0.380, 0.477; *n* = 223; *R* ^2^_marginal_ = 0.191; Figure 5). However, note that the magnitude and direction of persistent responses varied based on the life‐history stage exposed to temperatures (see above).

Albeit non‐significant, we found a negative association between dARR and the time at a common temperature after the initial acclimation (intercept = 0.050; 95% CI = −0.134, 0.234; slope = −0.009; 95% CI = −0.034, −0.016; *n* = 204; *R* ^2^_marginal_ = 0.048; Figure S3).

### Are terrestrial animals more plastic than aquatic animals?

**FIGURE 6:**
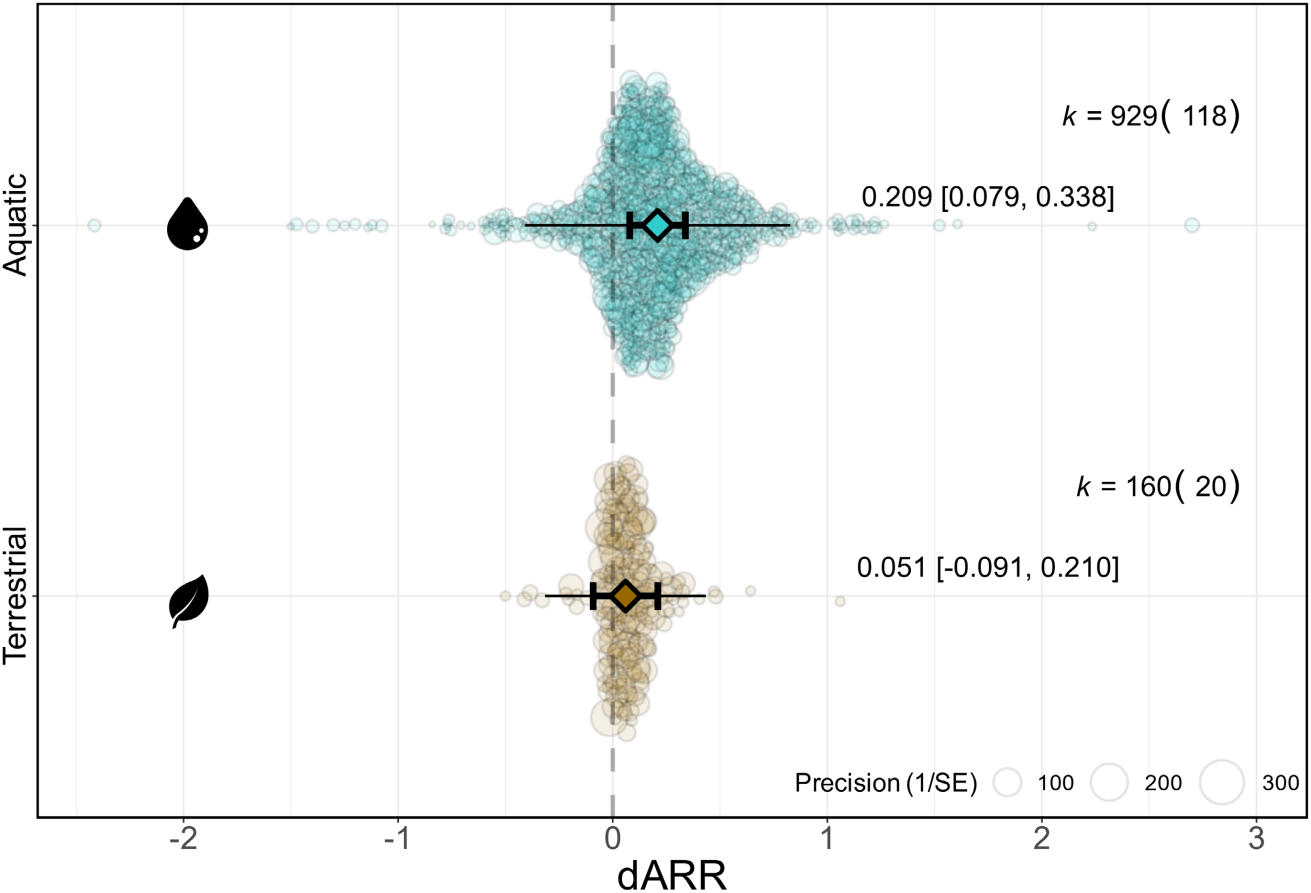
Habitat variation in developmental plasticity. Mean estimates (triangles) with their 95% confidence intervals (thicker bars with whiskers) and prediction intervals (thinner bars without whiskers) are depicted along with individual data points (coloured circles) scaled by precision (inverse of standard error). k: number of effect sizes (number of species). dARR: developmental acclimation response ratio.

**FIGURE 7:**
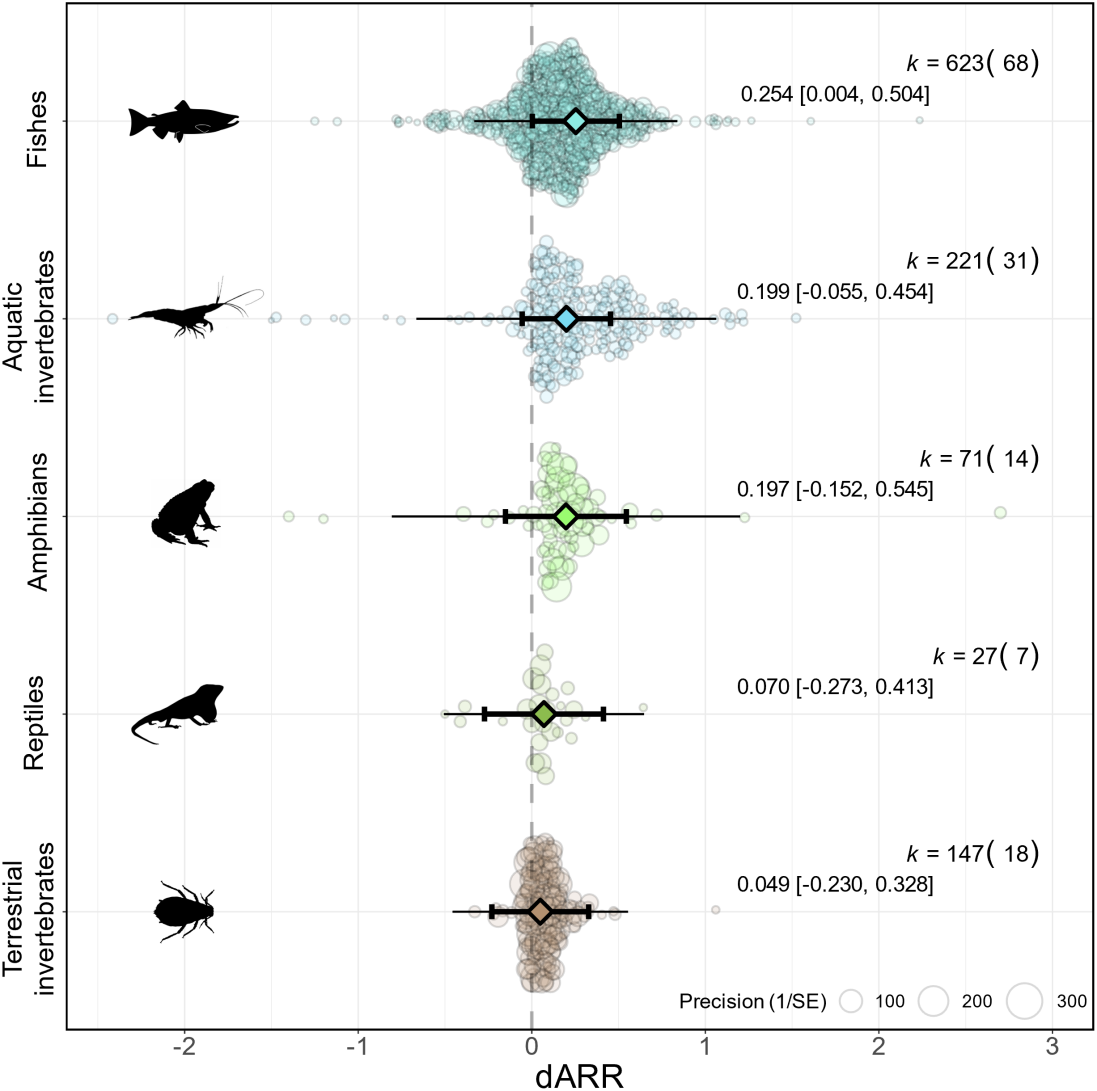
Taxonomic variation in developmental plasticity. Mean estimates (triangles) with their 95% confidence intervals (thicker bars with whiskers) and prediction intervals (thinner bars without whiskers) are depicted along with individual data points (coloured circles) scaled by precision (inverse of standard error). k: number of effect sizes (number of species). dARR: developmental acclimation response ratio. Taxonomic categorisations follow those of Morley et al., (2019). “Reptiles” refer to non‐avian reptiles.

We found that aquatic animals were more than three times as plastic as terrestrial animals (dARR_aquatic_ = 0.209; 95% CI = 0.079, 0.338; 95% PI = −0.410, 0.827; *n* = 929; dARR_terrestrial_ = 0.060; 95% CI = −0.091, 0.210; 95% PI = −0.315, 0.434; *n* = 160; *R* ^2^_marginal_ = 0.113; Figure 6). This variation aligned with differences between functional taxonomic groups (Figure 7). Fish, amphibians, and aquatic invertebrates expressed the largest plastic responses (dARR_fish_ = 0.254, 95% CI = 0.004, 0.504; 95% PI = −0.331, 0.839; *n* = 623; dARR_amphibians_ = 0.197, 95% CI = −0.152, 0.545; 95% PI = −0.807, 1.200; *n* = 71; dARR_aquatic invertebrates_ = 0.199, 95% CI = −0.055, 0.454; 95% PI = −0.665, 1.063; *n* = 221) whereas terrestrial animals had lower, and non‐statistically significant dARR estimates (dARR_reptiles_ = 0.070, 95% CI = −0.273, 0.413; 95% PI = −0.506, 0.647; *n* = 27; dARR_terrestrial invertebrates_ = 0.049, 95% CI = −0.230, 0.328; 95% PI = −0.457, 0.555; *n* = 147; *R* ^2^_marginal_ = 0.117).

### Is experimental methodology influential for estimating plasticity?

Neither the heat tolerance metric (dARR_CTmax_ = 0.195; 95% CI = 0.017, 0.372; 95% PI = −0.435, 0.829; *n* = 863; dARR_LT50_ = 0.162; 95% CI = −0.020, 0.343; 95% PI = −0.469, 0.798; *n* = 226; contrast = −0.033; 95%CI = −0.083, 0.017; *R* ^2^_marginal_ = 0.005) nor the heating rate (intercept = 0.212; 95% CI = 0.005, 0.419; slope = 0.019; 95% CI = −0.045, 0.084; *n* = 855; *R* ^2^_marginal_ <0.001) had statistically significant influence on developmental responses to temperatures. However, we found a positive association between heating rate and developmental responses to temperature after accounting for differences in body mass (Tables S39–S41). We also found evidence that developmental plasticity estimates were significantly influenced by the interaction between ramping rate and acclimation duration, as well as by a three‐way interaction between body mass, ramping rate, and acclimation duration (Tables S39–S41).

### Is there evidence for publication bias?

Visual inspections of the funnel plot of the model's residuals did not suggest evidence for publication bias (Figure S4). We also did not find evidence for publication bias (small‐study effect) when using robust multi‐level meta‐regressions (Table S26). Dissertations and theses provided qualitatively similar estimates to published findings (Table S27), and we found little evidence for a time‐lag bias (Table S28).

### How robust are our results?

Our results were robust to the iterative exclusion of one study or one species (Table S29). Investigating initial effects separately yielded higher estimates than previously presented, but generally qualitatively similar results (Tables S31, S32; Supporting Information S1; supplementary results). Analyses of persistent responses sometimes produced contrasting results to previously reported, but those analyses were deemed preliminary (Tables S33, S34; Supporting Information S1; supplementary results). Our results were also robust to the removal of (i) data acquired using uncommon methods, (ii) effect sizes for which sampling variance was imputed, and (iii) extreme negative effect sizes. However, removing extreme effect sizes tended to increase overall estimates (Table S35). Finally, the inclusion of body mass, heating rate, acclimation duration, and their interactions in models did not impact our main conclusions (Table S44).

## DISCUSSION

Understanding the extent to which ectotherms can acclimate to temperatures during their development is crucial to assess their vulnerability to rising temperatures. Here, we provide the first systematic review and quantitative synthesis to quantify the initial and persistent influence of developmental temperatures on heat tolerance across 138 ectothermic species.

### Early thermal environments have weak overall effects on thermal tolerance

Ectotherms raised at higher developmental temperatures tend to be slightly more tolerant to heat but the effects were weak (Figure 4). This pattern is akin to previous syntheses where data were mostly taken from adults (Gunderson & Stillman, 2015; Morley et al., 2019; Rohr et al., 2018) although early life stages seem to have a lower, and more variable, plasticity than adults. To increase heat tolerance by 1°C in developing ectotherms, it requires a 7.5°C shift in developmental temperatures (adjusted dARR ~0.13); whereas data from a previous synthesis on 278 adult ectothermic species (153 and 183 effect sizes from terrestrial and aquatic animals, respectively) points to a required shift of 4.2°C (ARR ~0.24; Morley et al., 2019). This discrepancy may be due to differences in study methodology and scope. First, previous syntheses often maximized positive ARR values by excluding effect sizes under a certain magnitude of response (e.g., excluding ARR below −0.15; or quantifying negative responses as null; Gunderson & Stillman, 2015; Morley et al., 2019). Such procedures may lead to an overestimation of the magnitude and direction of plastic responses by neglecting the possibility that ectotherms could express “non‐adaptive” (negative) responses to temperature exposures (Terblanche & Hoffmann, 2020). Unsurprisingly, excluding extremely negative effect sizes tended to increase our estimates. Negative responses have been argued to be biologically relevant and should be included in analyses to encompass the diversity of responses to temperatures organisms may exhibit (Terblanche & Hoffmann, 2020). Second, the low plasticity levels we observed may be due to biological and methodological variation. We observed an extremely high heterogeneity within and between species, which certainly contributed to the substantial width of our estimated confidence and prediction intervals. We aim to explain this variation in the next sections.

### Embryos respond differently to early thermal environments than juveniles

We found significant variation in degree of plastic responses based on the life‐history stage exposed to temperatures (Figure 5). Initial responses to acclimation during the embryonic stage are extremely heterogeneous. However, acclimation periods overlapping both the embryonic and juvenile stages tend to have similar impacts on heat tolerance compared to acclimation merely constrained to the juvenile stage. The analysis of long‐lasting impacts of developmental temperatures confirms this pattern (Figure 5). Embryonic temperatures differentially impact heat tolerance of later life stages, relative to juvenile developmental temperatures. While juveniles developing at higher temperatures tend to have slightly increased heat tolerance, animals incubated at higher temperatures as embryos and raised in standard conditions after hatching tend to have reduced thermal tolerance. These results suggest an important difference in the ability of embryos to adjust their heat tolerance relative to juvenile stages.

Our results are in favour of our alternative hypothesis that energy allocation trade‐offs may constrain the expression of plastic responses throughout ontogeny. Specifically, embryos, pupae, nymphs, and young larvae rely on endogenous energy reserves, whereas later life stages can resort to feeding to increase their energy intake. This reliance on energy reserves, combined with the important metabolic cost of growth (Marshall et al., 2020; Pettersen et al., 2018), may constrain energy allocation towards diverse functions, including plastic responses to temperatures. If energy allocation trade‐offs are major drivers of the ontogenetic variation in plasticity, then the low plasticity of embryos relative to juveniles may be due to the high energy demands of development and the limited capacity for embryos to increase their energy intake. Investigating whether limited access to nutrients constrain the expression of plastic responses in juveniles would be particularly interesting to confirm this hypothesis.

### Persistent responses to early thermal environments are common but not universal

Persistent responses of heat tolerance to developmental temperatures are not universal, which suggests that most of the responses recorded may represent reversible physiological acclimation rather than irreversible developmental thermal plasticity (sensu Beaman et al., 2016). Many ectotherms may successfully re‐acclimate to new environmental conditions, regardless of their early thermal history. However, we also note that only 26 studies investigated persistent responses, which is probably insufficient to reach adequate statistical power given the high heterogeneity in the data. In addition, we emphasize that the magnitude and direction of long‐lasting responses varied based on the life‐history stage exposed to temperatures (Figure 5). Therefore, we draw the reader's attention to the tendency for embryos to express negative responses to increased developmental temperatures, and the numerous cases where juvenile acclimation persistently impacts the heat tolerance of later life stages. We encourage additional research on the persistence of developmental plasticity to unravel whether those effects are robust and recommend prudence when assuming that laboratory acclimation erases the effects of early thermal history. The absence of evidence for a significant decrease in plasticity with re‐acclimation time may indicate that animals were already fully re‐acclimated to common garden conditions when assessed for thermal tolerance. Assessing the course of plasticity reversibility at various time scales is an important direction for future research.

### Shared evolutionary history and species ecology affect how species respond to early thermal environments

While we observed weak overall effects of early thermal environments on heat tolerance, effect size heterogeneity was high, suggesting that species exhibit diverse responses to early thermal environments. As predicted, a lot of this variation is due to species‐specific ecology and shared evolutionary history, with ~36% of the variation in effects driven by these two factors alone. Aquatic species were more plastic to thermal developmental environments than terrestrial species (Figures 6, 7). This observation confirms findings from previous syntheses focusing on later life stages (Gunderson & Stillman, 2015; Morley et al., 2019; Rohr et al., 2018) but contradicts our primary hypothesis that larger fluctuations in environmental temperatures may have selected for larger plastic responses in terrestrial animals. Instead, it provides support to our alternative hypothesis that body temperatures equilibrate faster in water, which may select for greater plasticity because of increased exposure to operative thermal fluctuations (Chevin & Hoffmann, 2017; Denny, 1993). Opportunities for behavioural thermoregulation were also hypothesised to be reduced in aquatic environments (Gunderson & Stillman, 2015), which may expose aquatic animals to even larger fluctuations in operative temperatures. In addition, greater selection for developmental plasticity may occur in aquatic environments as a response to limited oxygen availability (Pörtner et al., 2017; but see Jutfelt et al., 2018). On the other hand, terrestrial animals have more thermoregulatory opportunities and the selection for plastic physiological responses may be reduced (Muñoz, 2021). Because marine ectotherms are experiencing operative temperatures closer to their upper thermal limits (Pinsky et al., 2019), increased levels of plasticity seem imperative for their survival in a changing world. Assessing the extent to which plasticity compensates aquatic organisms for the increased exposure to extreme body temperatures is an interesting avenue for future research. While we might expect heavy and slow‐developing animals to be especially responsive to changes in thermal environments (Uno & Stillman, 2020), we found little evidence for a relationship between developmental plasticity in heat tolerance and body mass or age at sexual maturity. The reasons why animals with different life histories respond similarly to early thermal environments are unclear and require biological and methodological considerations (see next section).

### Methods for measuring heat tolerance can be influential

Although different metrics (i.e., CT_max_ or LT_50_) may yield different absolute levels of heat tolerance, the extent to which heat tolerance varies with developmental acclimation is relatively similar between metrics. While most quantitative syntheses on heat tolerance plasticity focused solely on CT_max_ (Barley et al., 2021; Gunderson & Stillman, 2015; Morley et al., 2019; Pottier, Burke, Drobniak et al., 2021; Rohr et al., 2018), we recommend, given statistical validation, the inclusion of LT_50_ in further syntheses. Slow heating rates result in extended time at high temperatures, which reduces thermal limits because of extended physiological stress (Rezende et al., 2011; Terblanche et al., 2007) and allow animals to acclimate during the experimental trials. Therefore, we predicted weak plasticity estimates at slow heating rates because extended heat stress and acclimation during assays reduce differences in thermal tolerance between cool‐ and warm‐acclimated animals. We found that, at equal body mass, animals tested at faster heating rates are usually more plastic, as predicted. Moreover, we detected previously described (Rohr et al., 2018) interactions between heating rate, body mass, and acclimation duration, but did not find evidence for interactions with latitudinal origin, probably due to a lack of statistical power. Our observations support that body size and methodological factors interact to shape the acclimation responses of ectotherms (Rohr et al., 2018).

### Limitations and future directions

While we aimed at performing a comprehensive systematic review, existing taxonomical and methodological biases in the literature (Figures 2, 3) constrain the generalisability of our findings. Notably, nearly 60% of the data eligible for our synthesis were on fish species, whereas we could only extract 27 relevant effect sizes in non‐avian reptiles. We encourage further research efforts on invertebrates and the herpetofauna for a more uniform distribution of data across the tree of life. We also observed a great disparity in the experimental designs employed to assess developmental plasticity in the literature (Figure 2). Most studies assessed the initial effects of developmental temperatures, with only 26 studies assessing whether those effects persist when animals are re‐acclimated to common garden conditions after the initial acclimation. Our synthesis also highlighted that only five studies tested for the initial plasticity of embryos. We stress the need for a greater standardization and unification of experimental approaches in the field, with a priority on the responses of embryos to varying temperatures. Importantly, we did not inspect whether there exist intrinsic differences in developmental plasticity within a life stage (e.g., between larval stages). However, basal thermal tolerance and plasticity may follow complex patterns throughout ontogeny (Klockmann et al., 2017; Pincebourde & Casas, 2015; Ruthsatz et al., 2022; Ruthsatz, Dausmann, et al., 2018) that need to be further investigated. We also encourage the use of state‐of‐the‐art meta‐analytic approaches to increase the reproducibility and comparability of evidence syntheses in comparative physiology (cf. Noble et al., 2022; Vetter et al., 2013). Particularly, a formal statistical comparison of the level of plasticity of adults relative to earlier life stages would represent an important advancement towards understanding and modelling how ectotherms will respond to rising temperatures.

### Implications for climate change impacts

Our study provides evidence that the capacity for ectotherms to adjust their heat tolerance is remarkably limited throughout their life cycle. Strikingly, nearly none of the 95% prediction intervals of the estimated effect sizes overlapped with unity. In other words, future changes in thermal phenotypes will rarely be expected to reach levels of perfect compensation, i.e., when heat tolerance perfectly tracks changes in environmental temperatures. We also observed numerous cases of reduced heat tolerance at higher developmental temperatures, particularly when acclimation occurred during the embryonic development (Figure 7). In fact, previous syntheses (Przeslawski et al., 2015; Collin et al., 2021; Dahlke et al., 2020; but see Pottier, Burke, Drobniak, et al., 2022 and Dahlke et al., 2022) and empirical work (e.g., Hall & Warner, 2019; Klockmann et al., 2017; Truebano et al., 2018; Turriago et al., 2015) suggest that embryos may have reduced thermal tolerance relative to other life stages. Non‐adaptive responses to developmental acclimation may represent a signature of physiological stress imposed upon embryos, possibly because of the inherent lower heat tolerance of this life stage. Low thermal tolerance combined with low, and sometimes non‐adaptive plasticity, brings embryos to the forefront of climate vulnerability. With rising temperatures, most animals may endure significant heat stress long before they reach the adult stage, although adults are often the focus of empirical studies and evidence syntheses. Assuming sufficient heritable variation, the strength of selection is expected to be stronger in embryos expressing non‐adaptive developmental plasticity. Investigating whether non‐adaptive plasticity may lead to rapid evolutionary change or extinction in a warming climate is thus a particularly interesting avenue for research (Gibert et al., 2019). We urge ecophysiologists to consider early life stages when assessing the vulnerability of ectotherms to changing temperatures.

Finally, although thermal tolerance limits are useful and intensively studied, evidence points to these metrics as not being perfect predictors of climate change vulnerability (Clusella‐Trullas et al., 2021). While thermal tolerance is relatively constrained, decreases in thermal sensitivity may help ectotherms tolerate heat waves for longer and ensure their survival (Seebacher et al., 2015; Rezende et al., 2020). Investigating how thermal tolerance and sensitivity are both impacted by early thermal environments within the same framework will represent a significant advancement towards understanding how ectotherms will navigate through changing environments. Thermal fertility limits, the temperatures at which animals lose fertility, may also represent better proxies (David et al., 2005; Parratt et al., 2021; van Heerwaarden & Sgrò, 2021; Walsh et al., 2019). In fact, fertility limits are much lower than standard thermal limits, and recent research suggest they may correlate better with global species distributions (Parratt et al., 2021; van Heerwaarden & Sgrò, 2021). Therefore, we may underestimate the impacts of rising temperatures by studying thermal tolerance limits. Notably, the development and maintenance of sexual organs and function may be sensitive to temperatures, and fertility loss may not be promptly reversible (Sales et al., 2021). Assessing the initial and persistent impacts of temperatures on fertility loss throughout ontogeny will be crucial to understand how ectotherms will navigate through changing environments.

## CONCLUSIONS

We found evidence for developing ectotherms to possess the ability to adjust their heat tolerance. Animals inhabiting aquatic environments tend to be more than three times as plastic as terrestrial animals, possibly because of their increased exposure to operative temperature fluctuations. Strikingly, we found evidence that embryos express a reduced, and more heterogenous plasticity than later life stages, with numerous responses appearing as non‐adaptive. Our study adds to the evidence that the embryonic stage may represent a critical window of vulnerability to changing temperatures. While we did not find universal evidence for developmental acclimation to have long‐lasting impacts on heat tolerance, persistent effects are common, and we call for increased consideration of those effects in future research. We also encourage a standardization of empirical studies and evidence syntheses, and we formally highlight important knowledge gaps in the literature. Overall, the capacity for developing ectotherms to adjust their thermal tolerance is limited and may provide minimal benefit in changing environments. Examining the combined impacts of developmental temperatures on thermal tolerance, sensitivity, and fertility will provide important insights into the future of most animals on the planet.

## AUTHORSHIP

Conceptualisation: PP, SB, RZ, DWAN, LES, SMD, SN. Methodology: PP, SB, RZ, DWAN, LES, SMD, SN. Software: PP, DWAN, SMD, SN. Formal Analysis: PP, DWAN, SMD, SN. Investigation: PP, SB, RZ. Data Curation: PP. Visualization: PP. Writing – Original Draft: PP. Writing – Review and Editing: PP, SB, RZ, DWAN, LES, SMD, SN. Project administration: PP. Supervision: SMD, SN. All authors gave final approval for publication.

## CONFLICT OF INTEREST

We declare no conflict of interest.

### PEER REVIEW

The peer review history for this article is available at https://publons.com/publon/10.1111/ele.14083.

### OPEN RESEARCH BADGES

This article has earned Open Data, Open Materials and Preregistered Research Design badges. Data, materials and the preregistered design and analysis plan are available at [https://osf.io/zkx6u].

## Supporting information


Appendix S1

Appendix S2

## Data Availability

Data and analysis code are available at https://github.com/p‐pottier/Dev_plasticity_thermal_tolerance and archived in Zenodo (https://doi.org/10.5281/zenodo.6818559; Pottier, Burke, Zhang, et al., 2022).
