# Supplementary material for: Developmental plasticity in thermal tolerance: Ontogenetic variation, persistence, and future directions
Source: Ecol Lett. 2022 Aug 25;25(10):2245–68. doi: 10.1111/ele.14083 (PMC9804923; doi:10.1111/ele.14083)
Supplement: Supplementary file 1 — Appendix S1 [file ELE-25-2245-s001.docx]

**Supporting Information S1**

**TABLE OF CONTENTS**

**Supplementary Methods3**

**Supplementary Results4**

**Supplementary Figures11s**

FIGURE S111

FIGURE S212

FIGURE S313

FIGURE S414

FIGURE S515

**Supplementary Tables16**

TABLE S117

TABLE S218

TABLE S320

TABLE S427

TABLE S534

TABLE S635

TABLE S736

TABLE S837

TABLE S939

TABLE S1040

TABLE S1142

TABLE S1243

TABLE S1345

TABLE S1446

TABLE S1547

TABLE S1648

TABLE S1750

TABLE S1851

TABLE S1953

TABLE S2054

TABLE S2155

TABLE S2256

TABLE S2357

TABLE S2458

TABLE S2559

TABLE S2662

TABLE S2763

TABLE S2864

TABLE S2965

TABLE S3066

TABLE S3167

TABLE S3268

TABLE S3369

TABLE S3470

TABLE S3571

TABLE S3672

TABLE S3773

TABLE S3874

TABLE S3976

TABLE S4079

TABLE S4181

TABLE S4284

TABLE S4386

TABLE S4487

TABLE S4590

**References91**

**Supplementary Methods**

***Literature searches and study selection***

We accessed Scopus, ISI Web of Science core collection, and ProQuest (Dissertation & Theses) on 05/03/2021. ProQuest was used as a source of grey literature.

We developed search strings to summarise studies manipulating the developmental temperature of ectothermic animals and subsequently measuring their heat tolerance. Search strings were adapted to the structure of each database, as follows:

Scopus:

TITLE-ABS-KEY("temperature*" OR "thermal" OR "cold*" OR "cool*" OR "heat*" OR "warm*" OR "acclimat*") AND TITLE-ABS-KEY("incubat*" OR "rear*" OR "development*" OR "ontogen*" OR "nest*" OR "embryo*" OR "egg*" OR "early*" OR "juvenile*" OR "hatchling*" OR "young*" OR "larv*" OR "fry" OR "alevin*" OR "fingerling*" OR "tadpole*" OR "froglet*" OR "pupa*" OR "nymph*" OR "*zoea*" OR "megalop*" OR "naupl*" OR "mysis" OR "life history stage*" OR "life stage*" OR "paralarv*" OR "neonat*" OR "neoten*" OR "subadult*" OR "metamorph" OR "metamorphic" OR "brood*" OR "split-brood*" OR "clutch*" OR "split-clutch*" OR "cyprid*" OR "veliger*" OR "bipinnaria*" OR "trochophore*" OR "immature*" OR "instar*" OR "copepodite*" OR "postlarva*" OR "post-larva*") AND TITLE-ABS-KEY("thermal tolerance*" OR "thermotolerance*" OR "heat tolerance*" OR "tolerance* to heat*" OR "tolerance* to temperature*" OR "temperature* tolerance*" OR "physiological tolerance*" OR "critical thermal m*" OR "critical temperature*" OR "thermal limit*" OR "thermal breadth*" OR "tolerance breadth*" OR "thermal range*" OR "performance breadth*" OR "thermal window*" OR "tolerance window*" OR "warming tolerance*" OR "tolerance* to warming" OR "CTmax" OR "CT max" OR "heat coma" OR "lethal temperature*" OR “temperature* lethal” OR "lethal limit*" OR "LTmax" OR "LT max" OR "heat stress tolerance*" OR "tolerance to heat stress" OR "thermal stress tolerance*" OR "tolerance* to thermal stress" OR "temperature stress tolerance*" OR "tolerance* to temperature stress" OR "tolerance* to heat shock " OR "heat shock tolerance*" OR "panting threshold*" OR "gaping threshold*" OR "loss of equilibrium" OR "onset of spasm*" OR "loss of righting response" OR "heat stupor" OR "death point*" OR "LT50" OR "LT100" OR “ULT50” OR “ULT100” OR "heat knockdown" OR "heat knock-down" OR "knockdown resistance" OR “knock-down resistance” OR "death time*" OR "knockdown time*" OR "knock-down time" OR "lethal time*" OR “heat resistance” OR “resistance to heat stress” OR “knockdown temperature*” OR “knock-down temperature*”) AND NOT TITLE-ABS-KEY("endotherm*" OR "bird*" OR "avia*" OR "aves" OR "mammal*" OR "rodent*" OR "mouse" OR "mice" OR "rat" OR "rats" OR "cattle*" OR "calves" OR "livestock*" OR "domesticated" OR "cow" OR "cows" OR “bovine” OR “beef” OR "pig" OR "pigs" OR "sheep*" OR "goat*" OR "horse*" OR "rabbit*" OR "chicken*" OR "duck*" OR "turkey*" OR "cat" OR "cats" OR "dog" OR "dogs" OR "chicks" OR "poultry" OR "dairy" OR "cultivar*" OR "germinat*" OR "panicle*" OR "tiller*" OR "sporophyt*" OR "gametophyt*" OR "*spore*" OR "vegetative" OR "seedling*" OR "stomat*" OR "photosynth*" OR "photochem*" OR "PSII" OR "chlorophyll*" OR "chloroplast*" OR "plant growth" OR "myceli*" OR "starch" OR "flower*" OR "shoot*" OR "germplasm*" OR "grain yield" OR "grain quality" OR "plant development" OR "arabidopsis" OR "bud" OR "fungi" OR "fungal" OR "yeast*" OR "alga" OR "algae" OR "algal" OR "seaweed*" OR "seagrass*" OR "cancer cell*" OR "tumor cell*" OR "lymphocyt*" OR "fungi" OR "fungal" OR "yeast*" OR "alga" OR "algae" OR "algal" OR "seaweed*" OR "seagrass*" OR "*bacteri*" OR "unicellular" OR "protist*" OR "archae*" OR "woman" OR "women" OR "man" OR "men" OR "volunteer*" OR "athlete*" OR "military" OR "patient*" OR "clothing" OR "sweat*") AND ( LIMIT-TO ( SUBJAREA,"AGRI" ) OR LIMIT-TO ( SUBJAREA,"BIOC" ) OR LIMIT-TO ( SUBJAREA,"ENVI" ) OR LIMIT-TO ( SUBJAREA,"EART" ) OR LIMIT-TO ( SUBJAREA,"MEDI" ) OR LIMIT-TO ( SUBJAREA,"MULT" ) OR EXCLUDE ( SUBJAREA,"ENGI" ) OR EXCLUDE ( SUBJAREA,"PHYS" ) OR EXCLUDE ( SUBJAREA,"CHEM" ) OR EXCLUDE ( SUBJAREA,"CENG" ) OR EXCLUDE ( SUBJAREA,"ENER" ) OR EXCLUDE ( SUBJAREA,"MATE" ) OR EXCLUDE ( SUBJAREA,"SOCI" ) OR EXCLUDE ( SUBJAREA,"COMP" ) OR EXCLUDE ( SUBJAREA,"BUSI" ) OR EXCLUDE ( SUBJAREA,"DECI" ) OR EXCLUDE ( SUBJAREA,"NURS" ) OR EXCLUDE ( SUBJAREA,"HEAL" ) OR EXCLUDE ( SUBJAREA,"ECON" ) OR EXCLUDE ( SUBJAREA,"ARTS" ) OR EXCLUDE ( SUBJAREA,"MATH" ) OR EXCLUDE ( SUBJAREA,"DENT" ) ) AND ( LIMIT-TO ( DOCTYPE , "ar" ) OR LIMIT-TO ( DOCTYPE , "re" ) OR LIMIT-TO ( DOCTYPE , "cp" ) )

ISI Web of Science (core collection):

TS=("temperature*" OR "thermal" OR "cold*" OR "cool*" OR "heat*" OR "warm*" OR "acclimat*") AND TS=("incubat*" OR "rear*" OR "development*" OR "ontogen*" OR "nest*" OR "embryo*" OR "egg*" OR "early*" OR "juvenile*" OR "hatchling*" OR "young*" OR "larv*" OR "fry" OR "alevin*" OR "fingerling*" OR "tadpole*" OR "froglet*" OR "pupa*" OR "nymph*" OR "*zoea*" OR "megalop*" OR "naupl*" OR "mysis" OR "life history stage*" OR "life stage*" OR "paralarv*" OR "neonat*" OR "neoten*" OR "subadult*" OR "metamorph" OR "metamorphic" OR "brood*" OR "split-brood*" OR "clutch*" OR "split-clutch*" OR "cyprid*" OR "veliger*" OR "bipinnaria*" OR "trochophore*" OR "immature*" OR "instar*" OR "copepodite*" OR "postlarva*" OR "post-larva*") AND TS=("thermal tolerance*" OR "thermotolerance*" OR "heat tolerance*" OR "tolerance* to heat*" OR "tolerance* to temperature*" OR "temperature* tolerance*" OR "physiological tolerance*" OR "critical thermal m*" OR "critical temperature*" OR "thermal limit*" OR "thermal breadth*" OR "tolerance breadth*" OR "thermal range*" OR "performance breadth*" OR "thermal window*" OR "tolerance window*" OR "warming tolerance*" OR "tolerance* to warming" OR "CTmax" OR "CT max" OR "heat coma" OR "lethal temperature*" OR “temperature* lethal” OR "lethal limit*" OR "LTmax" OR "LT max" OR "heat stress tolerance*" OR "tolerance to heat stress" OR "thermal stress tolerance*" OR "tolerance* to thermal stress" OR "temperature stress tolerance*" OR "tolerance* to temperature stress" OR "tolerance* to heat shock " OR "heat shock tolerance*" OR "panting threshold*" OR "gaping threshold*" OR "loss of equilibrium" OR "onset of spasm*" OR "loss of righting response" OR "heat stupor" OR "death point*" OR "LT50" OR "LT100" OR “ULT50” OR “ULT100” OR "heat knockdown" OR "heat knock-down" OR "knockdown resistance" OR “knock-down resistance” OR "death time*" OR "knockdown time*" OR "knock-down time" OR "lethal time*" OR “heat resistance” OR “resistance to heat stress” OR “knockdown temperature*” OR “knock-down temperature*”) NOT TS=("endotherm*" OR "bird*" OR "avia*" OR "aves" OR "mammal*" OR "rodent*" OR "mouse" OR "mice" OR "rat" OR "rats" OR "cattle*" OR "calves" OR "livestock*" OR "domesticated" OR "cow" OR "cows" OR “bovine” OR “beef” OR "pig" OR "pigs" OR "sheep*" OR "goat*" OR "horse*" OR "rabbit*" OR "chicken*" OR "duck*" OR "turkey*" OR "cat" OR "cats" OR "dog" OR "dogs" OR "chicks" OR "poultry" OR "dairy" OR "cultivar*" OR "germinat*" OR "panicle*" OR "tiller*" OR "sporophyt*" OR "gametophyt*" OR "*spore*" OR "vegetative" OR "seedling*" OR "stomat*" OR "photosynth*" OR "photochem*" OR "PSII" OR "chlorophyll*" OR "chloroplast*" OR "plant growth" OR "myceli*" OR "starch" OR "flower*" OR "shoot*" OR "germplasm*" OR "grain yield" OR "grain quality" OR "plant development" OR "arabidopsis" OR "bud" OR "fungi" OR "fungal" OR "yeast*" OR "alga" OR "algae" OR "algal" OR "seaweed*" OR "seagrass*" OR "cancer cell*" OR "tumor cell*" OR "lymphocyt*" OR "fungi" OR "fungal" OR "yeast*" OR "alga" OR "algae" OR "algal" OR "seaweed*" OR "seagrass*" OR "*bacteri*" OR "unicellular" OR "protist*" OR "archae*" OR "woman" OR "women" OR "man" OR "men" OR "volunteer*" OR "athlete*" OR "military" OR "patient*" OR "clothing" OR "sweat*") AND SU=( ECOLOGY OR ZOOLOGY OR MARINE FRESHWATER BIOLOGY OR ENTOMOLOGY OR BIOLOGY OR FISHERIES OR PHYSIOLOGY OR ENVIRONMENTAL SCIENCES OR BIOCHEMISTRY MOLECULAR BIOLOGY OR LIMNOLOGY OR EVOLUTIONARY BIOLOGY OR MULTIDISCIPLINARY SCIENCES OR OCEANOGRAPHY OR DEVELOPMENTAL BIOLOGY OR GENETICS HEREDITY OR BIODIVERSITY CONSERVATION) NOT SU=(BIOTECHNOLOGY APPLIED MICROBIOLOGY OR GEOGRAPHY PHYSICAL OR PLANT SCIENCES OR BIOPHYSICS ). Refined by: DOCUMENT TYPES: (ARTICLE OR REVIEW OR PROCEEDINGS PAPER)

ProQuest dissertations and theses:

(noft(development*) OR noft(incubat*) OR noft(acclimat*)) AND (noft(thermal tolerance*) OR noft(temperature* tolerance*) OR noft(thermal limit*) OR noft(heat tolerance*)) NOT noft(plants) NOT noft(mammal*) NOT noft(bird*)

Our searches found 5996 unique documents. Titles, abstracts, and keywords were screened by PP (90%), SB (5%) and RZ (5%) in Rayyan QCRI (Ouzzani et al., 2016). PP validated the screening of 120 bibliometric records (2%) screened by SB or RZ and 6 conflicts were observed. A total of 571 documents were further assessed for eligibility by PP. SB and RZ validated the full-text screening on 20 random documents (3.5%) and 5 conflicts were observed. Conflicts were due to misunderstandings about the inclusion criteria. All conflicts were discussed and resolved. Thirty-five documents were not accessible to the authors, and 32 studies were missing descriptive statistics for their direct inclusion in the meta-analysis (mean, sample size, and measure of dispersion). We contacted the authors of the original studies to request missing information if the study was published after 1995. We imputed missing standard deviations when authors did not respond but could not impute missing standard errors (see *Data extraction and effect size calculation*). One study (Cheung, 2019) was found to be eligible during pilot searches using Google Scholar (i.e., benchmarking, *sensu* Foo et al 2021), but was not captured by our search methods. Search methods are summarised in our PRISMA flowchart (Fig. 1), and the list of included studies is presented in the *Data sources* section.

***Deviations from registration:***

While essentially followed our original plans and procedures, we acknowledge minor deviations. We hypothesised that the magnitude of heat tolerance plasticity will vary based on which life stage was acclimated or tested. However, by treating the timing of acclimation and testing as separate predictors, we were not able to recover the experimental design information in sufficient detail (Fig. 2). For instance, animals tested at the juvenile stage can belong to any of the designs A, C, D, E or F. Therefore, we combined the information about the timing of acclimation and testing to assess the ontogenetic variation in plasticity more finely while keeping our original hypotheses. We also separated “initial” or “persistent” responses to developmental temperatures to assess the reversibility of developmental plasticity without fitting the time at a common temperature after the initial acclimation period, which was sometimes not reported in original studies, and difficult to generalize across taxa.

Because the distribution of the data was skewed towards aquatic animals (85.7% of effect sizes), the effect sizes from aquatic animals contributed more to the meta-analytic mean estimate than terrestrial ones. Therefore, we estimated marginal mean estimates for models assessing habitat variation in developmental plasticity. We used the package *emmeans* (Lenth, 2019; version 1.6.2) to obtain marginal means, where data from different habitats were given equal weights (i.e., post-stratification *sensu* Gelman et al., 2020).

Because our *a priori* moderators did not explain most of the data heterogeneity (see *Results*), we decided to fit a complex model with all moderators without missing values (i.e., habitat, taxonomic group, metric type, experimental design). We then used the *dredge* function in the *MuMIn* package (Barton, 2020; version 1.43.17) to fit models with all combinations of moderators to determine the ones explaining the most variation (models within two units of AICc from the best-fitting model), and the relative important of each moderator.

We also originally planned leave-one-out analyses on the meta-analytic model to determine the robustness of our results to the exclusion of one effect size or independent treatment (shared treatment ID). However, these models were computationally extensive and could not be ran. Note, however, that we performed leave-one-out analyses by iteratively removing one species or one study, which are more conservative.

Following recommendations at the peer-review stage, we fitted additional moderators to explain variation in developmental plasticity due to life history differences. Specifically, we first collected age at sexual maturity for 114 (82.6%) species by searching the AnAge and Animal Diversity Web databases. When data was not retrieved in those databases, we searched Google and Google Scholar with “species name” followed with a combination of either (age reproduct*), (age matur*), (sexual* matur*), or (development time). All searches were performed on 15/04/2022 and 21/04/2022. The list of data sources is presented in the Data sources section. We then fitted single-moderator models with the age at sexual maturity using the full data set, and subsets comprising either the initial or persistent responses to acclimation. We used a similar approach to examine the variation in developmental plasticity due to differences in body mass. We were also advised to examine variation in the data due to interactions between latitude, heating rate, acclimation duration, and body mass. Therefore, we fitted models with two- and three-way interactions between those variables using the full data set, and subsets comprising either the initial or persistent responses to acclimation. Finally, we also fitted models with habitat, taxonomic group, and the type of experimental design along with interactions between body mass, ramping rate and acclimation durations to examine whether our main conclusions are robust to controlling for body mass and methodological variation. We also examined the reversibility of plastic responses in greater detail. As such, we first calculated the ratio between the time animals were maintained in common garden conditions after developmental acclimation and their age at sexual maturity (a proxy for lifespan). We then fitted a single-moderator model with the relative time at a common garden temperature as a covariate. We also fitted the (relative) time at a common temperature together with the type of experimental design (i.e., D, E, F; cf. Figure 2) to examine whether our results were robust to controlling for differences in re-acclimation times after the initial acclimation.

**Supplementary Results**

Investigating initial effects separately yielded higher estimates than previously presented, but generally qualitatively similar results (Table S31-32). Yet, the initial dARR of reptiles was much higher than previously presented (dARR_reptiles_ = 0.143, 95% CI = -0.153, 0.439; 95% PI = -0.313, 0.599; n = 12); although it is relevant to note the low sample size for this taxonomic group. However, analyses of persistent responses produced contrasting results to previously reported (Table S33-34). We acknowledge, however, that most of these analyses had low sample sizes, and must be considered as preliminary. Terrestrial animals were as plastic as aquatic animals, and confidence intervals overlapped with zero in both cases (dARR_aquatic_ = 0.051; 95% CI = -0.157, 0.258; 95% PI = -0.493, 0.594; n = 148; dARR_terrestrial_ = 0.049; 95% CI = -0.173, 0.271; 95% PI = -0.401, 0.499; n = 75; R^2^_marginal_ < 0.001). Persistent developmental plasticity estimates were low and sometimes negative in vertebrates (dARR_fish_ = -0.029, 95% CI = -0.338, 0.279; 95% PI = -0.654, 0.592; n = 127; dARR_amphibians_ = 0.005, 95% CI =-0.444, 0.454; 95% PI= -0.786, 0.796; n = 6; dARR_reptiles_ = -0.047, 95% CI = -0.436, 0.341; 95% PI = -0.707, 0.613; n = 15; R^2^_marginal_ = 0.109), whereas higher temperatures tended to persistently increase heat tolerance in invertebrates (dARR_terrestrial invertebrates_ = 0.097, 95% CI = -0.210, 0.404; 95% PI = -0.416, 0.609; n = 62; dARR_aquatic invertebrates_ = 0.136, 95% CI = -0.148, 0.419; 95% PI = -0.378, 0.650; n = 13).

**Supplementary Figures**


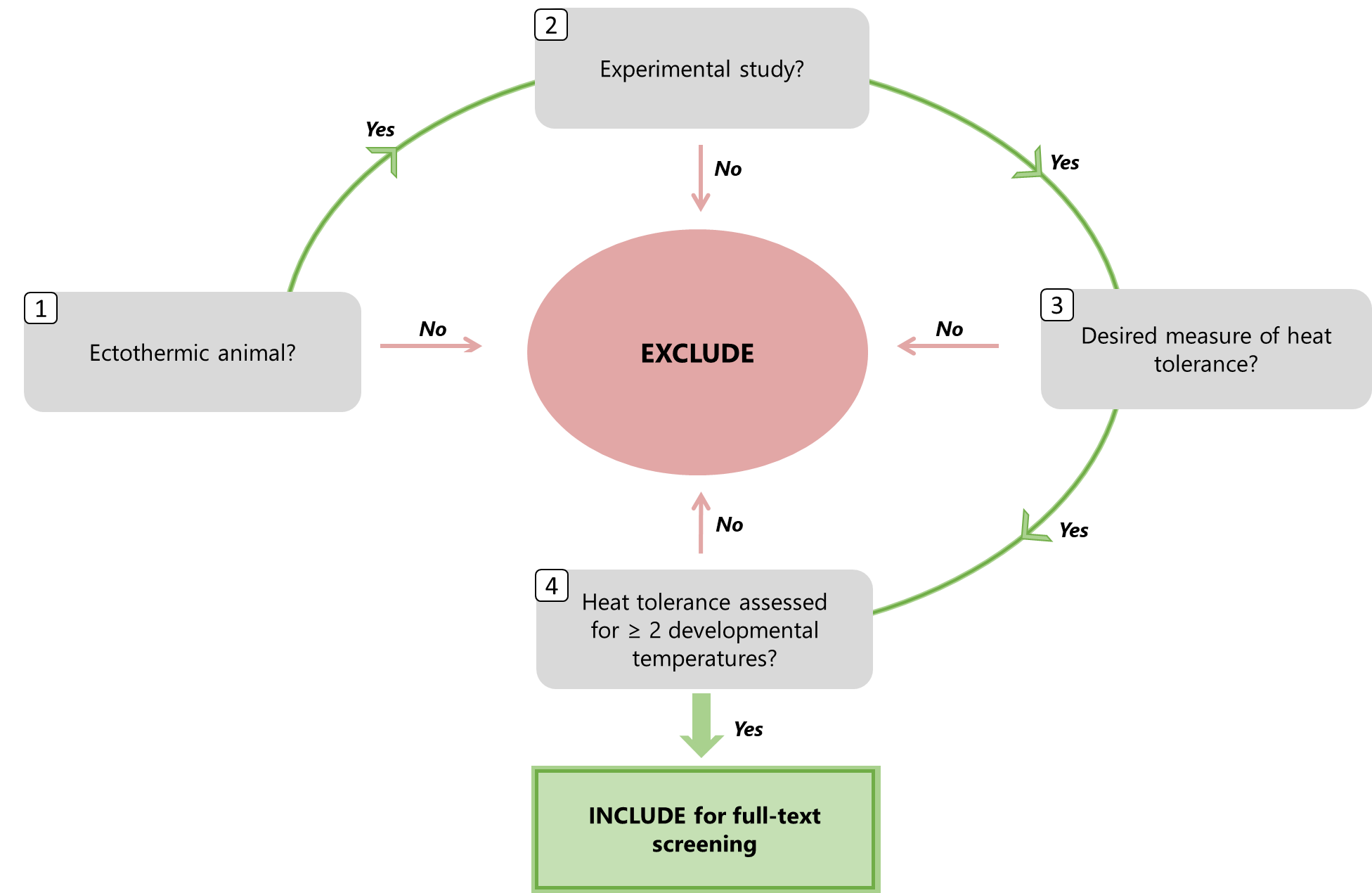


**FIGURE S1:** **Decision tree used for screening titles, abstracts, and keywords**. Details of each inclusion criteria can be found in Table S1.


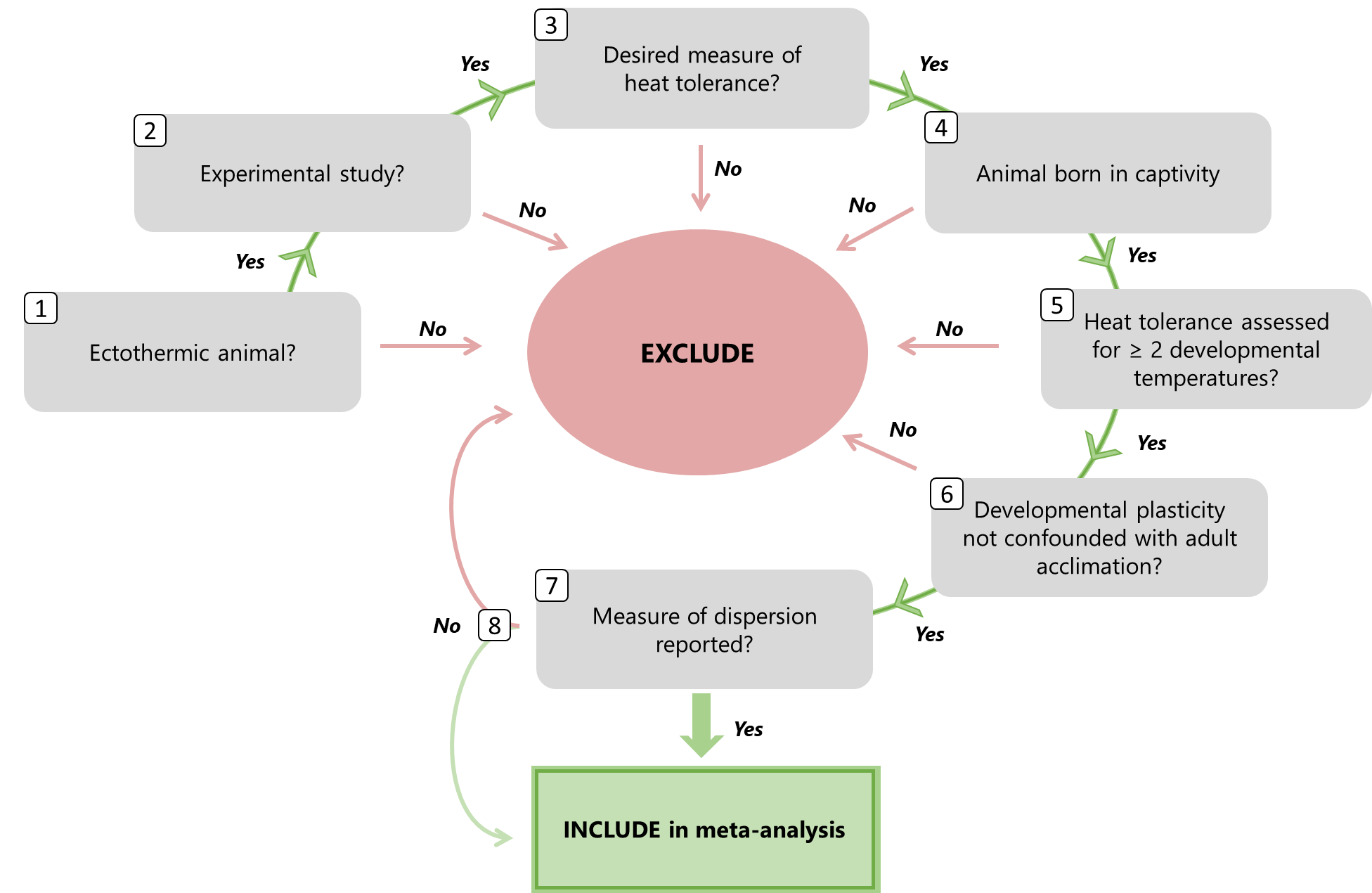


**FIGURE S2:** **Decision tree used to assess full articles for eligibility.** Details of each inclusion criteria can be found in Table S2.


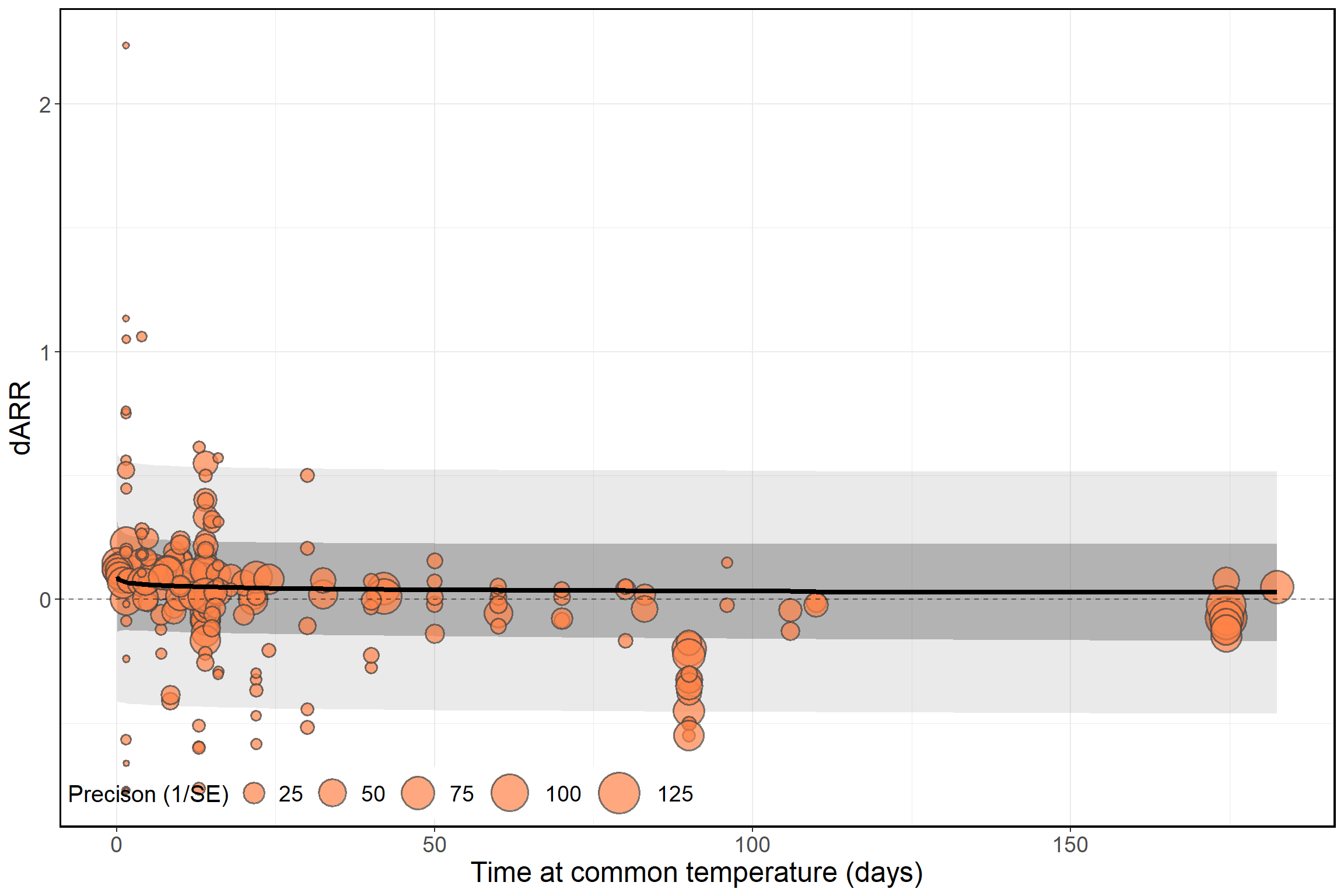


**FIGURE S3:** **Relationship between developmental acclimation capacity (dARR) and the time in a common garden condition after the initial acclimation.** The dark line represent predictions from the meta-regression with their associated 95% confidence (dark grey shaded area) and prediction (light grey shaded area) intervals Individual points (orange circles) were scaled by precision (1/SE). SE: standard error.


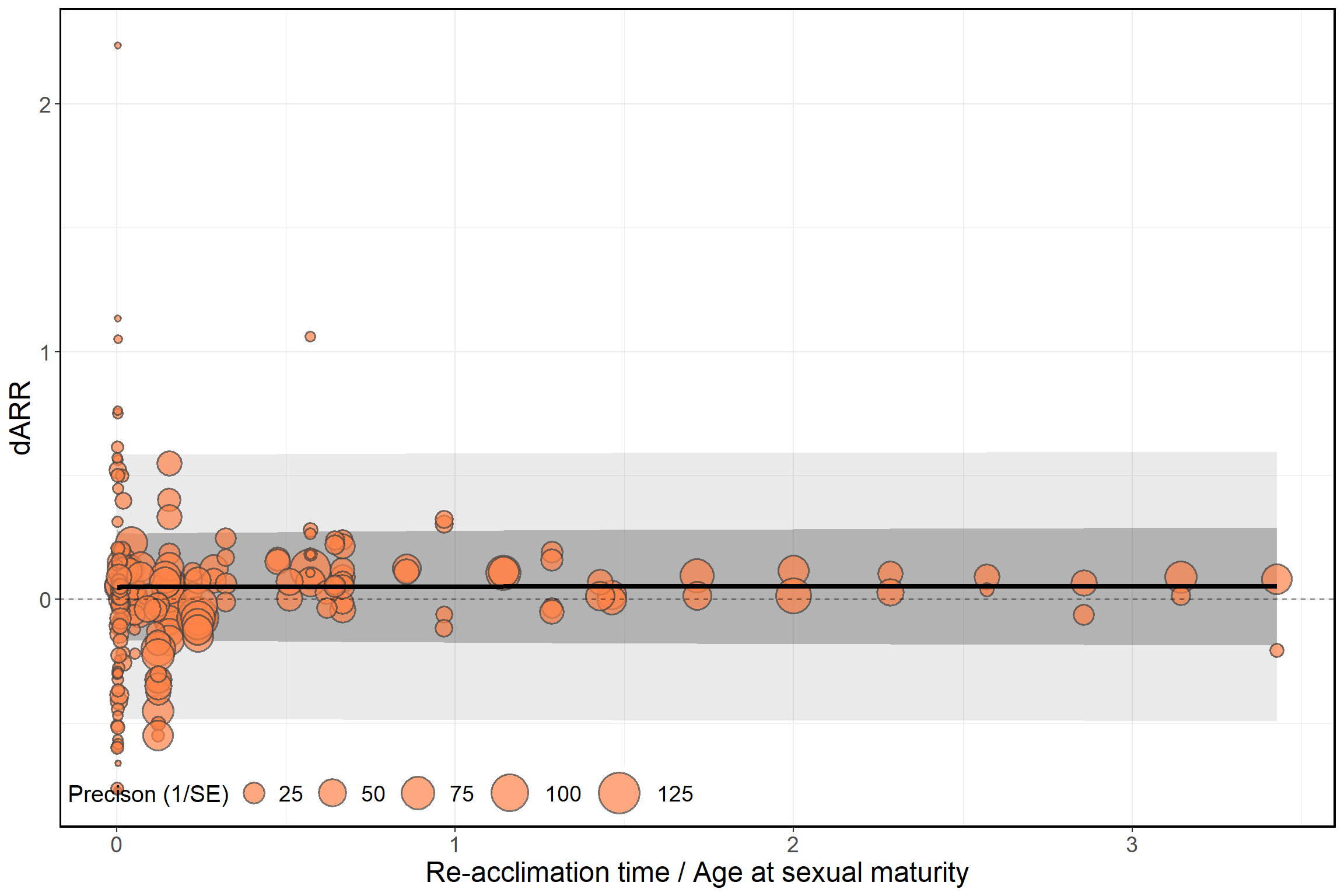


**FIGURE S4:** **Relationship between developmental acclimation capacity (dARR) and the relative time in a common garden condition after the initial acclimation.** The relative time in a common garden situation was calculated as the ratio between the time in a common garden condition (days) and the age at sexual maturity (days), which was used as a proxy for lifespan. The dark line represent predictions from the meta-regression with their associated 95% confidence (dark grey shaded area) and prediction (light grey shaded area) intervals Individual points (orange circles) were scaled by precision (1/SE). SE: standard error.


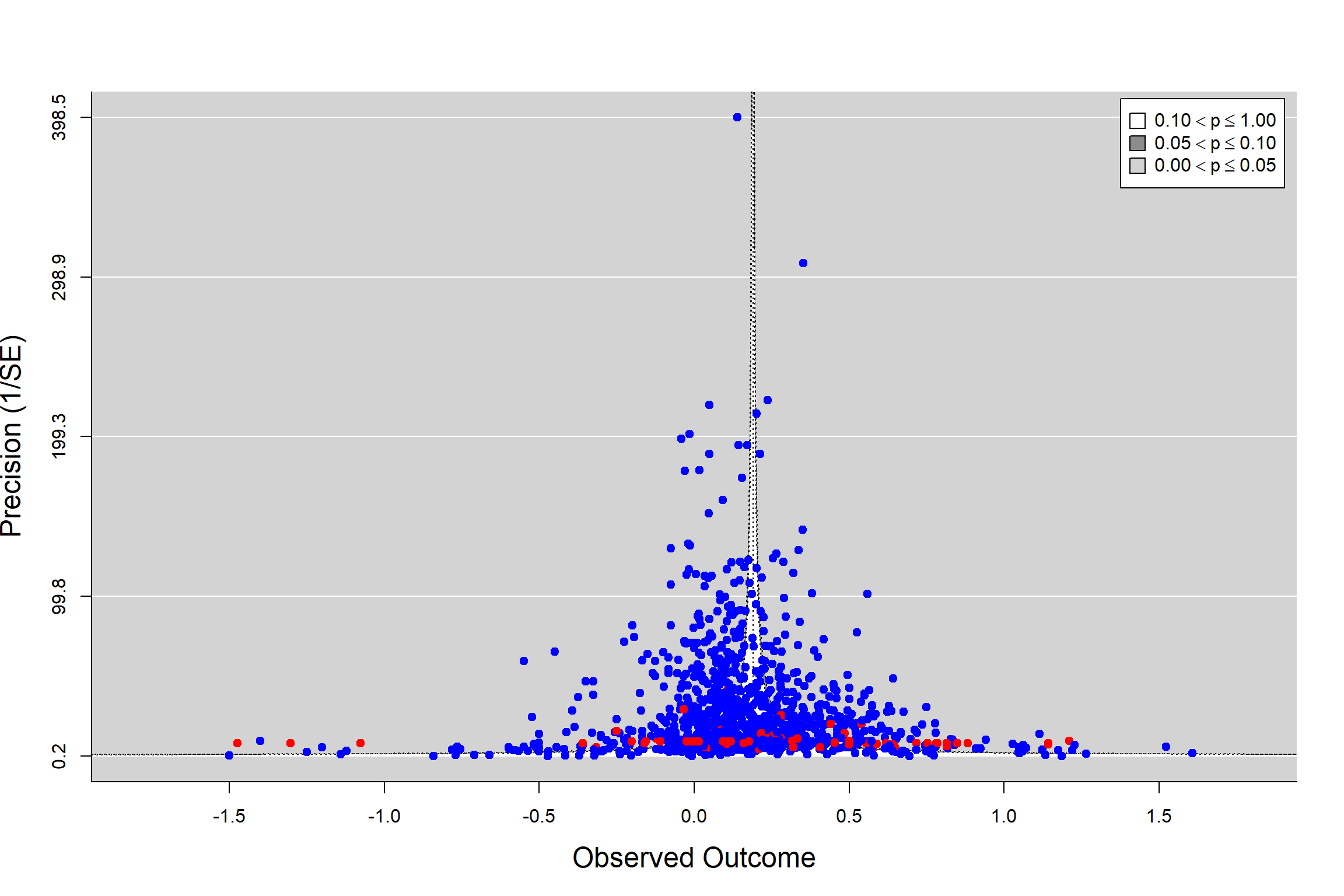


**FIGURE S5:** **Funnel plot showing the distribution of effect sizes based on their precision.** Blue points represent original values whereas red points represent effect sizes for which the standard deviation were imputed. SE: standard error.

**Supplementary Tables**

**TABLE S1*:*** **Description of the inclusion criteria used to screen titles, abstracts, and keywords.** Numbers match these used in in Figure S1 (decision tree).

|  | **Description** |
| --- | --- |
| **1** | "Ectothermic animals" referred to fish, amphibians, reptiles, and invertebrates. We only included studies performed on whole animals. Hence, we excluded studies on birds, mammals, plants, unicellular organisms, inanimate objects, and cells isolated from ectothermic animals. |
| **2** | “Experimental” study referred to manipulative laboratory experiments. Therefore, we excluded qualitative reviews and models (unless supplemented with original data), field experiments, theoretical studies, observational laboratory experiments, and conference abstracts. |
| **3** | Desired measures of upper thermal tolerance include the i) critical thermal maximum (CTmax), where animals are subject to incremental increases in temperature until an endpoint is reached; ii) the temperature lethal for 50% of the animals (LT50; sometimes referred to as the “incipient lethal temperature”), where survival is recorded after animals are abruptly transferred to a set of high temperatures for a given period of time (e.g. 96 hours) and LT50 is interpolated from the survival curve; and iii) the death time or heat knockdown time (HKT) where animals are abruptly transferred to elevated temperatures and the time needed for animals to reach an endpoint (e.g. immobilisation, death) is recorded as the response. With the latter measure, the thermal tolerance limit can be inferred from the relationship between HKT and the temperature of the knockdown assay. Therefore, heat knockdown times must have been measured at >2 temperatures.  We excluded alternative measures of upper thermal tolerance which cannot be converted to the temperature scale (e.g. heat coma recovery time) or CTmax extrapolated from physiological performance curves (e.g. critical temperature for ATPase activity). |
| **4** | We included studies in which independent groups of animals were exposed to >2 mean controlled (laboratory setting) temperatures during the embryonic or juvenile development, and subsequently assessed for thermal tolerance. Care was given to studies testing LT50 or survival at different temperatures (i.e. survival data is used to infer LT50). In such studies, LT50 must have been assessed for ≥ 2 developmental treatments (i.e. ≥ 2 independent survival curves). That is, studies manipulating incubation temperature of embryos and subsequently assessing the survival of each treatment at their respective incubation temperature were excluded (i.e. survival must have been measured at different temperatures for each treatment). Rapid heat or cold hardening (brief, <24h exposure to a temperature) was not considered a suitable manipulation of developmental temperature. Studies only comparing two temperature treatments with the same mean but differing by their variance were excluded.  If the life stage of the animals was not indicated, we included the record to the full text screening. If the abstract clearly mentioned that only adults were acclimated to different temperatures, we excluded the study. However, if the abstract mentioned that adults were tested for thermal tolerance, but did not mention when animals were acclimated, we included the study to the full-text screening (animals may have been acclimated as juveniles and tested as adults).  The term “developmental” has various terminologies and is sometimes referred to as, for instance, “acclimation” or “rearing”. Care was given regarding the terminology, and records were not excluded solely on a terminological basis.  When no mention was made regarding temperature manipulations, we exclude the study. For instance, if the study clearly stated that they investigated the effect of different diets on thermal tolerance (but did not mention that they tested whether the effect of diet interact with the temperature of incubation); we assume that no temperature manipulation was performed. We also excluded studies on animals recently collected from the wild (e.g. acclimation of wild-caught juveniles) because the embryo incubation temperature was not controlled. However, we included studies in which embryos were collected from the wild. |

**TABLE S2:** **Description of the inclusion criteria used to screen full texts of studies**. Numbers match these used in in Figure S2 (decision tree).

|  | **Description** |
| --- | --- |
| **1** | "Ectothermic animals" referred to fish, amphibians, reptiles, and invertebrates. We only included studies performed on whole animals. Hence, we excluded studies on birds, mammals, plants, unicellular organisms, inanimate objects, and cells isolated from ectothermic animals. |
| **2** | “Experimental” study referred to manipulative laboratory experiments. Therefore, we excluded qualitative reviews and models (unless supplemented with original data), field experiments, theoretical studies, observational laboratory experiments, and conference abstracts. |
| **3** | Desired measures of upper thermal tolerance included the i) critical thermal maximum (CTmax), where animals are subject to incremental increases in temperature until an endpoint is reached; ii) the temperature lethal for 50% of the animals (LT50; sometimes referred to as the “incipient lethal temperature”), where survival is recorded after animals are abruptly transferred to a set of high temperatures for a given period of time (e.g. 96 hours) and LT50 is interpolated from the survival curve; and iii) the death time or heat knockdown time (HKT) where animals are abruptly transferred to elevated temperatures and the time needed for animals to reach an endpoint (e.g. immobilisation, death) is recorded as the response. With the latter measure, the thermal tolerance limit can be inferred from the relationship between HKT and the temperature of the knockdown assay. Therefore, heat knockdown times must have been measured at >2 temperatures.  We excluded alternative measures of upper thermal tolerance which cannot be converted to the temperature scale (e.g. heat coma recovery time) or CTmax extrapolated from physiological performance curves (e.g. critical temperature for ATPase activity). |
| **4** | To be included, studies must have controlled for the incubation temperature of the embryos. Therefore, we included studies on animals born in captivity (e.g. laboratory, fish farms, pet stores) because these environments are generally controlled. We excluded studies where juveniles were collected from the wild because embryos may have experienced different thermal histories. However, we included studies in which embryos were collected from the wild. |
| **5** | We included studies in which independent groups of animals were exposed to >2 mean controlled (laboratory setting) temperatures during the embryonic or juvenile development and subsequently assessed for thermal tolerance. We also included studies manipulating the temperature fluctuation around the mean as long as the temperature variation was predictable (e.g. sinusoid, day/night changes) and could be compared to another treatment that differed by its mean, but not its fluctuation We excluded studies using unpredictable temperature variables (e.g. chaotic temperature variation, or heat spikes). Heat or cold hardening (brief, <24h exposure to a temperature) were not considered a suitable manipulation of developmental temperature.  If either the life stage, or a proxy for the life stage (e.g. body length, age) was not indicated, the study was excluded.  To be included, the developmental temperature must have been independently manipulated and not confounded with other known factors (i.e. *all else being equal*). |
| **6** | We excluded studies confounding developmental plasticity with adult acclimation. For instance, if the developmental temperature was prolonged after sexual maturity, early developmental plasticity is indistinguishable from recent adult acclimation. Therefore, to be included, adult measures of heat tolerance must have been performed on adults acclimated to the same temperature but differing by their developmental thermal history. For logistic reasons, the incubation from egg to adult may be continued for hours posterior the adult transition (e.g. emergence from pupa). We tolerated such overlap with adult acclimation when the time spent in adult acclimation is ≤ 24h. |
| **7** | A measure of dispersion (standard deviation, standard error, variance, range, coefficient of variation, confidence intervals) must have been provided for each group of animals. For studies reporting LT50 without a measure of dispersion, we included the studies presenting a survival curve (% of survival at each temperature, for each incubation temperature) along with sample sizes at each temperature tested, as it allowed the estimation of LT50 and its standard error.  If such data was not reported, we contacted authors for further information or imputed the data (see 8.) |
| **8** | Corresponding author(s) of studies not reporting descriptive summary statistics were contacted if the study was published after 1995. This cut was arbitrary, but we assumed that missing information was less likely to be retrieved from older studies. We imputed the missing standard deviations when authors did not address our requests but could not impute missing standard errors (see *Data extraction and effect size calculations*). |

**TABLE S3:** **Meta-data.** Full list of extracted and calculated data with data descriptions.

| **Data** | **Description** |
| --- | --- |
| initials | Initials of the researcher who performed the data extraction. |
| es_ID | Unique identifier for each effect size. |
| study_ID | Unique identifier for each study. |
| species_ID | Unique identifier for each species. |
| population_ID | Unique identifier for each population (collection site). In such case, we refer to distinctions between populations made by the authors; or consider animals taken from the same location or born from the same parents as the same population. |
| family_ID | Unique identifier for the family-level non-independence. Animals originating from the same clutch, or the same parents were assigned the same family_ID. When not presented, animals from the same population were considered the same family. |
| shared_trt_ID | Unique identifier for comparisons involving the same data (e.g., comparisons between 15-17°C, 17-20°C and 20-23°C acclimation treatments). |
| cohort_ID | Unique identifier for each cohort. By “cohort”, we designate independent groups of animals. In some cases, different data can be estimated on the same cohorts of animals (e.g., by using different endpoints). We, however, differentiate this identifier from shared_trt_ID, meaning that treatments re-used in multiple comparisons were not necessarily considered the same cohorts to reduce the overlap between these factors. |
| note_ID | General notes about the columns above. |
| data_source | Where the heat tolerance data is reported (e.g., text, figure, published data). |
| data_url | If the data was published, the url link to the repository containing the data. |
| fig_file_name | If the data is presented in a figure, the name of the file containing the figure. |
| data_type | Whether the data was published or obtained from authors. Factor with two levels: published or obtained. |
| data_file_name | Name of the published (or obtained) data file. |
| peer_reviewed | Whether the publication was peer-reviewed (i.e. published in a journal) or not (i.e. thesis). Factor with two levels: “peer-reviewed” or “not peer-reviewed”. |
| ref | Abbreviated reference for the study. |
| title | Title of the paper. |
| pub_year | Publication year of the paper. |
| journal | Journal the paper was published in. If the study is a thesis, indicate “thesis”. |
| thesis_chapter | If the study is a thesis, the chapter included. |
| doi | DOI of the paper. |
| citation | Citation for the paper. |
| phylum | Phylum. |
| class | Class. |
| order | Order. |
| family | Family. |
| genus | Genus. |
| species | Species. |
| genus_species | Species name. |
| age_maturity | Age at sexual maturity. ADW: Animal Diversity Web; AnAge: Animal Ageing and Longevity Database. |
| ref_age_maturity | Reference source for the age at sexual maturity. References are in the *Data sources* section of the manuscript. |
| habitat | The habitat. Factor with two levels: “terrestrial” or “aquatic”. For animals inhabiting different habitats during their life cycle, we used the habitat the life stage was inhabiting when tested for heat tolerance. |
| taxonomic_group | The taxonomic group of the species. Factor with five levels: “reptile”, “fish”, “amphibian”, “terrestrial invertebrate” and “aquatic invertebrate” (following Morley et al., 2019). |
| reproduction_mode | Whether the embryos were exposed to their thermal environment independently (oviparous), or through their parents (viviparous). Factor with two levels: “oviparous” or “viviparous”. External fertilizers were considered “oviparous”, mouth-breeders “viviparous”, and ovoviviparous animals were considered “viviparous”. |
| life_stage_manip | The life stage subjected to manipulations of developmental temperatures. Factor with three levels: “embryo”, “juvenile” or “embryo_and_juvenile”. |
| life_stage_tested | The life stage assessed for heat tolerance. Factor with three levels: “embryo”, “juvenile”, or “adult”. |
| brought_common_temp | Whether animals were brought to a common garden temperature after manipulating developmental temperature. Factor with two levels: “yes” or “no”. |
| mobility_life_stage_manip | The mobility status of the life stage subjected to manipulations of developmental temperatures. Factor with two levels: “mobile” or “immobile”. Embryos and pupae were considered “immobile”. |
| time_common_temp | If animals were brought to a common temperature, the time (days) animals were maintained at this common temperature until tested for heat tolerance. |
| common_temp | The mean temperature of the common garden temperature (°C). |
| exp_design | The type of experimental design, as described in Fig. XXX. |
| origin_hatching | Origin of the studied animals (where they hatched). |
| latitude | Latitude from which animals were collected (decimal degrees). If animals were collected at various latitudes and the data is not reported separately for each population (or the raw data is not published), we took the mean of the different latitudes reported. |
| longitude | Longitude from which animals were collected (decimal degrees). If animals were collected at various longitudes and the data is not reported separately for each population (or the raw data is not published), then we took the mean of the different longitude reported. |
| elevation | Elevation from which animals were collected (meters above sea level), as reported in the study. If not reported, it was estimated from Google Earth. |
| season | Season from which animals were collected. Factor with four levels: “spring”, “summer”, “fall” or “winter”. If the sampling spanned two seasons, we took the season spanning most of the sampling period. |
| year | Year from which the animals were collected. If the sampling spanned two years, we took the year spanning most of the sampling period. |
| body_length | Mean body length of the animals (mm). |
| body_mass | Mean body mass of the animals (g). |
| age_tested | The age (days-post-hatching) at which the animals were tested for heat tolerance. |
| sex | The sex of the animals. Factor with four levels: “male”, “female”, “mixed” or “unknown”. The “mixed” category was used when authors clearly stipulate that they mixed males and females. |
| housing_temp | The mean temperature of laboratory housing prior to acclimation (°C). |
| incubation_independent | Whether the temperatures of incubation compared are independent from housing_temp. |
| metric | The metric used to assess thermal tolerance. Factor with two levels: “CTmax” or “LT50”. |
| endpoint | The endpoint that was used for assessing heat tolerance (loss of righting response, loss of equilibrium, onset of spasms, death, other). Factor with four levels: “LRR”, “LOE”, “OS”, “death”, “other”. If “other”, indicate details in “notes_moderators”. |
| acc_temp_low | Mean temperature of acclimation of the experimental group acclimated to the lower temperature (°C). |
| acc_temp_high | Mean temperature of acclimation of the experimental group acclimated to the higher temperature (°C). |
| acc_temp_var | Variability in acclimation temperature expressed as the standard deviation around the mean acclimation temperature (°C). |
| is_acc_temp_fluctuating | Whether the acclimation temperature is highly variable (± > 0.5°C) or relatively constant (± ≤ 0.5°C). Factor with two levels: “fluctuating” or “constant”. When not reported, the temperature was considered “constant”. |
| acc_duration | Duration of acclimation (days). |
| ramping | If the metric was CTmax, the ramping (heating) rate applied to the animals (°C/min). |
| set_time | If the metric was LT50, the time the animals spent at the test temperature (the time after which the animals the survival was assessed, in hours). If the authors report e.g. 96h-LT50, then set_time would be 96. |
| n_test_temp | If the metric was LT50, the number of temperatures tested to assess heat tolerance. E.g., if authors measured survival at 36, 38, 39, and 41°C, n_test_temp = 4. |
| n_replicates_per_temp | If the metric was LT50, the number of replicates used at each test temperatures. E.g., if authors used 5 test temperatures and measured the survival of three independent cohorts of animals at each test temperature, then n_replicates_per_temp = 3. |
| n_animals_per_replicate | If the metric was LT50, the number of animals in each replicate. |
| humidity | Humidity at which animals were acclimated or tested (% relative humidity). If the humidity during the acclimation and the test were different, priority was given to the conditions of the test. |
| oxygen | Oxygen at which animals were acclimated or tested (mg.L^-1^ dissolved oxygen). If the oxygen concentration during the acclimation and the test were different, priority was given to the conditions of the test. |
| salinity | Salinity at which animals were acclimated or tested (parts per thousands). If the salinity during the acclimation and the test were different, priority was given to the conditions of the test. |
| pH | pH at which animals were acclimated. If the pH during the acclimation and the test were different, priority was given to the conditions of the test. |
| photoperiod | Photoperiod at which animals were acclimated (number of hours of light per day) |
| gravidity | Gravidity status of females. Factor with four levels: “non-gravid”, “gravid” (egg-bearing), “spent” (recently spawned), or “mixed”. |
| starved | Whether the animals were starved or fed before the heat tolerance assessment. Factor with two levels: “starved” or “fed”. |
| minor_concern | Potential minor concerns regarding the data (e.g. approximate latitude). A concern is considered “minor” when it may influence the moderator data or slightly influence the heat tolerance estimates. |
| major_concern | Potential major concerns about the data (e.g. heat tolerance measured in a peculiar way). A concern was considered major when it may significantly influence the heat tolerance estimate. |
| notes_moderators | Comments and details about the moderator variables. |
| mean_HT_low | Mean heat tolerance of the animals acclimated to the lower temperature (°C). |
| sd_HT_low | Standard deviation or standard error of mean_HT_low (see error_type). |
| n_HT_low | Sample size of mean_HT_low. For LT50 data, the sample size was taken as n_test_temp * n_replicates_per_temp. |
| mean_HT_high | Mean heat tolerance of the animals acclimated to the higher temperature (°C). |
| sd_HT_high | Standard deviation of mean_HT_high (see error type). |
| n_HT_high | Sample size of mean_HT_high. For LT50 data, the sample size was taken as n_test_temp * n_replicates_per_temp. |
| error_type | Whether the measure of dispersion of sd_HT_low and sd_HT_high is standard deviation (sd) or standard error (se). |
| notes_es | Comments and details about the heat tolerance data. |
| exclude | Whether the observation should be excluded. Were excluded only the observations for which the standard error was missing and could not be imputed. |
| n_trt | The number of cases the observation shares the same shared_trt_ID with other observations. |
| n_cohort | The number of cases the observation shares the same cohort_ID with other observations. |
| within_study_mean_low | The mean heat tolerance at the lowest acclimation temperature (mean_HT_low) for a given study. |
| within_study_mean_high | The mean heat tolerance at the highest acclimation temperature (mean_HT_high) for a given study. |
| within_study_sd_low | The mean standard deviation at the lowest acclimation temperature (sd_HT_low) for a given study. |
| within_study_sd_high | The mean standard deviation at the lowest acclimation temperature (sd_HT_high) for a given study. |
| between_study_mean_low | The mean heat tolerance at the lowest acclimation temperature (mean_HT_low) across studies. |
| between_study_mean_high | The mean heat tolerance at the highest acclimation temperature (mean_HT_high) across studies. |
| between_study_sd_low | The mean standard deviation at the lowest acclimation temperature (sd_HT_low) across studies. |
| between_study_sd_high | The mean standard deviation at the lowest acclimation temperature (sd_HT_high) across studies. |
| imputed | Whether the standard deviation was presented or calculated from information presented in the original study (“no”) or imputed (“yes”). |
| imputed_sd_low | Imputed or original standard deviation at the lowest acclimation temperature. |
| imputed_sd_high | Imputed or original standard deviation at the highest acclimation temperature. |
| dARR | Developmental acclimation response ratio. Calculated using Equation 1. |
| Var_dARR | Sampling variance of dARR. Calculated using Equations 2, 3, 4, or 5; depending on the nature and dependence of the data (see main text for details). |
| precision | Precision (inverse of the standard error) of dARR estimates. |
| is_concern | Whether the data has a risk of bias. Factor with two levels: “yes” or “no”. |

**Table S4: PRISMA-EcoEvo checklist.** For details about each item, see O’Dea et al. (2021).

| **Checklist Item** | **Sub-item Number** | **Sub-item** | **Reported by Authors?** | **Location** |
| --- | --- | --- | --- | --- |
| Title and abstract | 1.1 | Identify the review as a systematic review, meta-analysis, or both | Yes | Title; Abstract |
|  | 1.2 | Summarise the aims and scope of the review | Yes | Abstract |
|  | 1.3 | Describe the data set | Yes | Abstract |
|  | 1.4 | State the results of the primary outcome | Yes | Abstract |
|  | 1.5 | State conclusions | Yes | Abstract |
|  | 1.6 | State limitations | Yes | Abstract |
| Aims and questions | 2.1 | Provide a rationale for the review | Yes | Introduction |
|  | 2.2 | Reference any previous reviews or meta-analyses on the topic | Yes | Introduction |
|  | 2.3 | State the aims and scope of the review (including its generality) | Yes | Introduction |
|  | 2.4 | State the primary questions the review addresses (e.g. which moderators were tested) | Yes | Introduction; Materials and Methods: *Moderator variables and predictions* |
|  | 2.5 | Describe whether effect sizes were derived from experimental and/or observational comparisons | Yes | Introduction; Materials and Methods: *Eligibility criteria* |
| Review registration | 3.1 | Register review aims, hypotheses (if applicable), and methods in a time-stamped and publicly accessible archive and provide a link to the registration in the methods section of the manuscript. Ideally registration occurs before the search, but it can be done at any stage before data analysis. | Yes | <https://osf.io;zkx6u> |
|  | 3.2 | Describe deviations from the registered aims and methods | Yes | Materials and Methods: *Deviations from registration;* Supplementary methods |
|  | 3.3 | Justify deviations from the registered aims and methods | Yes | Materials and Methods: *Deviations from registration;* Supplementary methods |
| Eligibility criteria | 4.1 | Report the specific criteria used for including or excluding studies when screening titles and/or abstracts, and full texts, according to the aims of the systematic review (e.g. study design, taxa, data availability) | Yes | Materials and Methods: *Eligibility criteria*; Fig. 1; Fig. S1-2; Table S1-2. |
|  | 4.2 | Justify criteria, if necessary (i.e. not obvious from aims and scope) | Yes | Materials and Methods: *Eligibility criteria*; Table S1-2. |
| Finding studies | 5.1 | Define the type of search (e.g. comprehensive search, representative sample) | Yes | Materials and Methods: *Literature searches and study selection* |
|  | 5.2 | State what sources of information were sought (e.g. published and unpublished studies, personal communications) | Yes | Materials and Methods: *Literature searches and study selection* |
|  | 5.3 | Include, for each database searched, the exact search strings used, with keyword combinations and Boolean operators | Yes | Supplementary methods |
|  | 5.4 | Provide enough information to repeat the equivalent search (if possible), including the timespan covered (start and end dates) | Yes | Materials and Methods: *Literature searches and study selection ; Supplementary methods* |
| Study selection | 6.1 | Describe how studies were selected for inclusion at each stage of the screening process (e.g. use of decision trees, screening software) | Yes | Materials and Methods: *Literature searches and study selection; Eligibility criteria*; Fig. 1; Fig. S1-2; Table S1-2. |
|  | 6.2 | Report the number of people involved and how they contributed (e.g. independent parallel screening) | Yes | Materials and Methods: *Literature searches and study selection;* Supplementary methods |
| Data collection process | 7.1 | Describe where in the reports data were collected from (e.g. text or figures) | Yes | Materials and Methods: *Data extraction and effect size calculations* |
|  | 7.2 | Describe how data were collected (e.g. software used to digitize figures, external data sources) | Yes | Materials and Methods: *Data extraction and effect size calculations* |
|  | 7.3 | Describe moderator variables that were constructed from collected data (e.g. number of generations calculated from years and average generation time) | NA: no moderator variables were calculated from other values |  |
|  | 7.4 | Report how missing or ambiguous information was dealt with during data collection (e.g. authors of original studies were contacted for missing descriptive statistics, and/or effect sizes were calculated from test statistics) | Yes | Materials and Methods: *Literature searches and study selection* |
|  | 7.5 | Report who collected data | Yes | Materials and Methods: *Data extraction and effect size calculations* |
|  | 7.6 | State the number of extractions that were checked for accuracy by co-authors | Yes | Materials and Methods: *Data extraction and effect size calculations* |
| Data items | 8.1 | Describe the key data sought from each study | Yes | Materials and Methods: *Data extraction and effect size calculations* |
|  | 8.2 | Describe items that do not appear in the main results, or which could not be extracted due to insufficient information | Yes | Materials and Methods: *Data extraction and effect size calculations* |
|  | 8.3 | Describe main assumptions or simplifications that were made (e.g. categorising both ‘length’ and ‘mass’ as ‘morphology’) | NA: no assumptions or simplifications needed to be made |  |
|  | 8.4 | Describe the type of replication unit (e.g. individuals, broods, study sites) | Yes | Materials and Methods: *Data extraction and effect size calculations* |
| Assessment of individual study quality | 9.1 | Describe whether the quality of studies included in the systematic review or meta-analysis was assessed (e.g. blinded data collection, reporting quality, experimental vs. observational) | Yes | Materials and Methods: *Sensitivity analyses and publication bias* |
|  | 9.2 | Describe how information about study quality was incorporated into analyses (e.g. meta-regression and/or sensitivity analysis) | Yes | Materials and Methods: *Sensitivity analyses and publication bias* |
| Effect size measures | 10.1 | Describe effect size(s) used | Yes | Materials and Methods: *Data extraction and effect size calculations* |
|  | 10.2 | Provide a reference to the equation of each calculated effect size (e.g. standardised mean difference, log response ratio) and (if applicable) its sampling variance | Yes | Materials and Methods: *Data extraction and effect size calculations* |
|  | 10.3 | If no reference exists, derive the equations for each effect size and state the assumed sampling distribution(s) | Yes | Materials and Methods: *Data extraction and effect size calculations* |
| Missing data | 11.1 | Describe any steps taken to deal with missing data during analysis (e.g. imputation, complete case, subset analysis) | Yes | Materials and Methods: *Data extraction and effect size calculations* |
|  | 11.2 | Justify the decisions made to deal with missing data | Yes | Materials and Methods: *Data extraction and effect size calculations* |
| Meta-analytic model description | 12.1 | Describe the models used for synthesis of effect sizes | Yes | Materials and Methods: *Meta-analysis and meta-regressions* |
|  | 12.2 | The most common approach in ecology & evolution will be a random-effects model, often with a hierarchical/multilevel structure. If other types of models are chosen (e.g. common/fixed effects model, unweighted model), provide justification for this choice | NA: only (weighted) random-effects models were used |  |
| Software | 13.1 | Describe the statistical platform used for inference (e.g. *R*) | Yes | Materials and Methods: *Meta-analysis and meta-regressions* |
|  | 13.2 | Describe the packages used to run models | Yes | Materials and Methods: *Meta-analysis and meta-regressions;* Table S42. |
|  | 13.3 | Describe the functions used to run models | Yes | Materials and Methods: *Meta-analysis and meta-regressions* |
|  | 13.4 | Describe any arguments that differed from the default settings | Yes | Materials and Methods: *Meta-analysis and meta-regressions* |
|  | 13.5 | Describe the version numbers of all software used | Yes | Materials and Methods: *Meta-analysis and meta-regressions;* Table S35. |
| Non-independence | 14.1 | Describe the types of non-independence encountered (e.g. phylogenetic, spatial, multiple measurements over time) | Yes | Materials and Methods: *Meta-analysis and meta-regressions* |
|  | 14.2 | Describe how non-independence has been handled | Yes | Materials and Methods: *Meta-analysis and meta-regressions* |
|  | 14.3 | Justify decisions made | Yes | Materials and Methods: *Meta-analysis and meta-regressions* |
| Meta-regression and model selection | 15.1 | Provide a rationale for the inclusion of moderators (covariates) that were evaluated in meta-regression models | Yes | Materials and Methods: *Meta-analysis and meta-regressions* |
|  | 15.2 | Justify the number of parameters estimated in models, in relation to the number of effect sizes and studies (e.g. interaction terms were not included due to insufficient sample sizes) | Yes | Materials and Methods: *Meta-analysis and meta-regressions* |
|  | 15.3 | Describe any process of model selection | Yes | Materials and Methods: *Meta-analysis and meta-regressions; Deviations from registration* |
| Publication bias and sensitivity analyses | 16.1 | Describe assessments of the risk of bias due to missing results (e.g. publication, time-lag, and taxonomic biases) | Yes | Materials and Methods: *Sensitivity analyses and publication bias; Deviations from registration* |
|  | 16.2 | Describe any steps taken to investigate the effects of such biases (if present) | Yes | Materials and Methods: *Sensitivity analyses and publication bias; Deviations from registration* |
|  | 16.3 | Describe any other analyses of robustness of the results, e.g. due to effect size choice, weighting or analytical model assumptions, inclusion or exclusion of subsets of the data, or the inclusion of alternative moderator variables in meta-regressions | Yes | Materials and Methods: *Sensitivity analyses and publication bias* |
| Clarification of post hoc analyses | 17.1 | When hypotheses were formulated after data analysis, this should be acknowledged. | Yes | Materials and methods: *Deviations from registration* |
| Metadata, data, and code | 18.1 | Share metadata (i.e. data descriptions) | Yes | Table S3 |
|  | 18.2 | Share data required to reproduce the results presented in the manuscript | Yes | Archived upon acceptance |
|  | 18.3 | Share additional data, including information that was not presented in the manuscript (e.g. raw data used to calculate effect sizes, descriptions of where data were located in papers) | Yes | Archived upon acceptance |
|  | 18.4 | Share analysis scripts (or, if a software package with graphical user interface (GUI) was used, then describe full model specification and fully specify choices) | Yes | Archived upon acceptance |
| Results of study selection process | 19.1 | Report the number of studies screened | Yes | Materials and Methods: *Literature searches and study selection*; Fig. 1 |
|  | 19.2 | Report the number of studies excluded at each stage of screening | Yes | Materials and Methods: *Literature searches and study selection*; Fig. 1 |
|  | 19.3 | Report brief reasons for exclusion from the full text stage | Yes | Materials and Methods: *Literature searches and study selection*; Fig. 1 |
|  | 19.4 | Present a Preferred Reporting Items for Systematic Reviews and Meta-Analyses (PRISMA)-like flowchart (www.prisma-statement.org). | Yes | Fig. 1 |
| Sample sizes and study characteristics | 20.1 | Report the number of studies and effect sizes for data included in meta-analyses | Yes | Results: *Description of the data* set; Fig. 1 |
|  | 20.2 | Report the number of studies and effect sizes for subsets of data included in meta-regressions | Yes | Results; Table S5; Fig. 2-7 |
|  | 20.3 | Provide a summary of key characteristics for reported outcomes (either in text or figures; e.g. one quarter of effect sizes reported for vertebrates and the rest invertebrates) | Yes | Results: *Description of the data* set; Fig. 2-3 |
|  | 20.4 | Provide a summary of limitations of included moderators (e.g. collinearity and overlap between moderators) | Yes | Results: *Description of the data* set |
|  | 20.5 | Provide a summary of characteristics related to individual study quality (risk of bias) | Yes | Table S6. |
| Meta-analysis | 21.1 | Provide a quantitative synthesis of results across studies, including estimates for the mean effect size, with confidence/credible intervals | Yes | Results: *Overall level of developmental plasticity*; Fig. 4 |
| Heterogeneity | 22.1 | Report indicators of heterogeneity in the estimated effect (e.g. I2, tau2 and other variance components) | Yes | Results: *Overall level of developmental plasticity;* Table S7 |
| Meta-regression | 23.1 | Provide estimates of meta-regression slopes (i.e. regression coefficients) and confidence/credible intervals | Yes | Results; Fig. 4-7; Table S7-41. |
|  | 23.2 | Include estimates and confidence/credible intervals for all moderator variables that were assessed (i.e. complete reporting) | Yes | Results; Fig. 4-7; Table S7-41. |
|  | 23.3 | Report interactions, if they were included | NA: no interactions were included |  |
|  | 23.4 | Describe outcomes from model selection, if done (e.g. R2 and AIC) | Yes | Table S20, 24-26. |
| Outcomes of publication bias & sensitivity analyses | 24.1 | Provide results for the assessments of the risks of bias (e.g. Egger's regression, funnel plots) | Yes | Results: *Publication* bias; Fig. S4; Table S27-30, 35-36. |
|  | 24.2 | Provide results for the robustness of the review's results (e.g. subgroup analyses, meta-regression of study quality, results from alternative methods of analysis, and temporal trends) | Yes | Results: *Sensitivity analyses*; Table S28-30, 35-36. |
| Discussion | 25.1 | Summarise the main findings in terms of the magnitude of effect | Yes | Discussion |
|  | 25.2 | Summarise the main findings in terms of the precision of effects (e.g. size of confidence intervals, statistical significance) | Yes | Discussion |
|  | 25.3 | Summarise the main findings in terms of their heterogeneity | Yes | Discussion |
|  | 25.4 | Summarise the main findings in terms of their biological/practical relevance | Yes | Discussion |
|  | 25.5 | Compare results with previous reviews on the topic, if available | Yes | Discussion |
|  | 25.6 | Consider limitations and their influence on the generality of conclusions, such as gaps in the available evidence (e.g. taxonomic and geographical research biases) | Yes | Discussion |
| Contributions and funding | 26.1 | Provide names, affiliations, and funding sources of all co-authors | Yes | Title page |
|  | 26.2 | List the contributions of each co-author | Yes | Authorship |
|  | 26.3 | Provide contact details for the corresponding author | Yes | Title page |
|  | 26.4 | Disclose any conflicts of interest | Yes | Conflicts of interest |
| References | 27.1 | Provide a reference list of all studies included in the systematic review or meta-analysis | Yes | Data sources (References) |
|  | 27.2 | List included studies as referenced sources (e.g. rather than listing them in a table or supplement) | Yes | Data sources (References) |

**TABLE S5:** **Sample sizes for each categorical moderator**.

| **Moderator** | **Studies** | **Species** | **Effect sizes** |
| --- | --- | --- | --- |
| Aquatic | 128 | 118 | 929 |
| Terrestrial | 22 | 20 | 160 |
| Amphibians | 10 | 14 | 71 |
| Aquatic invertebrates | 29 | 31 | 221 |
| Fishes | 83 | 68 | 623 |
| Reptiles | 9 | 7 | 27 |
| Terrestrial invertebrates | 19 | 18 | 147 |
| Initial | 126 | 121 | 866 |
| Persistent | 26 | 23 | 223 |
| Design A | 104 | 109 | 700 |
| Design B | 5 | 5 | 20 |
| Design C | 21 | 13 | 146 |
| Design D | 7 | 6 | 92 |
| Design E | 8 | 7 | 76 |
| Design F | 12 | 11 | 55 |
| CTmax | 132 | 118 | 863 |
| LT50 | 30 | 35 | 226 |

**TABLE S6:** **Number of studies presenting potential risks of bias.** For the time spend at the time at the test temperature (set_time), refer to Table S3.

| **Risk of bias** | **Number of studies** |
| --- | --- |
| Elliott’s hybrid methodology | 4 |
| CTmax inferred from thermal tolerance landscape | 4 |
| Missing or estimated measure of dispersion (standard error or standard deviation) | 16 |
| Unclear sample size | 3 |
| Unusually high standard error | 1 |
| Data very similar to another study | 1 |
| Very short time spend at the test temperature (set_time) | 1 |

**TABLE S7:** **Results of the meta-analytic intercept model**. est: estimate; se: standard error; CI.lb: lower bound of the 95% confidence interval; CI.ub: upper bound of the 95% confidence interval; PI.lb: lower bound of the 95% prediction interval; PI.ub: upper bound of the 95% prediction interval. I^2^_total_: total amount of heterogeneity (%); I^2^_species_: percentage of heterogeneity explained by differences between species(%); I^2^_phylo_: percentage of heterogeneity explained by shared evolutionary history; I^2^_es_: heterogeneity associated with the residuals (within-species variation); k_species_: number of species included in the model; k_es_: number of effect sizes included in the model.

| Overall mean | **est** | **se** | **p** | **CI.lb** | **CI.ub** | **PI.lb** | **PI.ub** |
| --- | --- | --- | --- | --- | --- | --- | --- |
|  | 0.190 | 0.088 | 0.033 | 0.015 | 0.364 | -0.444 | 0.823 |
|  | **I^2^_total_** | **I^2^_species_** | **I^2^_phylo_** | **I^2^_es_** | **k_species_** | **k_es_** |  |
|  | 99.4 | 9.95 | 26.10 | 64.44 | 138 | 1089 |  |

**TABLE S8:** **Results of univariate meta-regressions fitted with categorical moderators.** Coefficients statistically different from zero were highlighted in bold. est: estimate; se: standard error; CI.lb: lower bound of the 95% confidence interval; CI.ub: upper bound of the 95% confidence interval; PI.lb: lower bound of the 95% prediction interval; PI.ub: upper bound of the 95% prediction interval; k_sp_: number of species included in the model; k_es_: number of effect sizes included in the model; Var_sp_: variance explained by variation between species; Var_phy_: variance explained by shared evolutionary history; Var_es_: variance associated with the residuals; A. inverts: aquatic invertebrates; T. inverts: terrestrial invertebrates; CTmax: critical thermal maximum; LT50: median lethal temperature. Designs are outlined in Fig. 2.

|  | **est** | **se** | **p** | **CI.lb** | **CI.ub** | **PI.lb** | **PI.ub** | **k_sp_** | **k_es_** | **Var_sp_** | **Var_phy_** | **Var_es_** |
| --- | --- | --- | --- | --- | --- | --- | --- | --- | --- | --- | --- | --- |
| **Aquatic** | **0.209** | **0.066** | **0.002** | **0.077** | **0.340** | -0.379 | 0.796 | 118 | 929 | 0.010 | 0.013 | 0.061 |
| Terrestrial | 0.067 | 0.082 | 0.413 | -0.095 | 0.229 | -0.528 | 0.662 | 20 | 160 |  |  |  |
|  | **est** | **se** | **p** | **CI.lb** | **CI.ub** | **PI.lb** | **PI.ub** | **k_sp_** | **k_es_** | **Var_sp_** | **Var_phy_** | **Var_es_** |
| Amphibians | 0.208 | 0.154 | 0.179 | -0.096 | 0.513 | -0.470 | 0.886 | 14 | 71 | 0.009 | 0.024 | 0.061 |
| A. inverts | 0.190 | 0.113 | 0.095 | -0.033 | 0.414 | -0.455 | 0.836 | 31 | 221 |  |  |  |
| **Fishes** | **0.241** | **0.116** | **0.040** | **0.012** | **0.471** | -0.407 | 0.889 | 68 | 623 |  |  |  |
| Reptiles | 0.067 | 0.163 | 0.684 | -0.256 | 0.340 | -0.620 | 0.753 | 7 | 27 |  |  |  |
| T. inverts | 0.054 | 0.129 | 0.678 | -0.201 | 0.308 | -0.604 | 0.711 | 18 | 147 |  |  |  |
|  | **est** | **se** | **p** | **CI.lb** | **CI.ub** | **PI.lb** | **PI.ub** | **k_sp_** | **k_es_** | **Var_sp_** | **Var_phy_** | **Var_es_** |
| **Initial** | **0.221** | **0.068** | **0.001** | **0.088** | **0.354** | -0.352 | 0.795 | 121 | 866 | 0.006 | 0.014 | 0.060 |
| Persistent | 0.039 | 0.073 | 0.588 | -0.103 | 0.182 | -0.536 | 0.615 | 23 | 223 |  |  |  |
|  | **est** | **se** | **p** | **CI.lb** | **CI.ub** | **PI.lb** | **PI.ub** | **k_sp_** | **k_es_** | **Var_sp_** | **Var_phy_** | **Var_es_** |
| **Design A** | **0.222** | **0.068** | **0.001** | **0.089** | **0.355** | -0.347 | 0.791 | 109 | 700 | 0.006 | 0.014 | 0.060 |
| Design B | 0.117 | 0.096 | 0.225 | -0.072 | 0.306 | -0.468 | 0.702 | 5 | 20 |  |  |  |
| **Design C** | **0.250** | **0.076** | **0.001** | **0.102** | **0.399** | -0.323 | 0.823 | 13 | 146 |  |  |  |
| Design D | -0.093 | 0.082 | 0.258 | -0.254 | 0.068 | -0.670 | 0.484 | 6 | 92 |  |  |  |
| Design E | 0.106 | 0.083 | 0.205 | -0.058 | 0.269 | -0.472 | 0.683 | 7 | 76 |  |  |  |
| Design F | 0.113 | 0.082 | 0.165 | -0.047 | 0.273 | -0.463 | 0.689 | 11 | 55 |  |  |  |
|  | **est** | **se** | **p** | **CI.lb** | **CI.ub** | **PI.lb** | **PI.ub** | **k_sp_** | **k_es_** | **Var_sp_** | **Var_phy_** | **Var_es_** |
| **CTmax** | **0.197** | **0.090** | **0.029** | **0.021** | **0.373** | -0.435 | 0.829 | 118 | 863 | 0.010 | 0.026 | 0.060 |
| LT50 | 0.165 | 0.092 | 0.074 | -0.016 | 0.345 | -0.469 | 0.798 | 35 | 226 |  |  |  |

**TABLE S9:** **Results of the univariate meta-regression used to assess the difference in developmental plasticity between aquatic and terrestrial animals**. Differences statistically different from zero were highlighted in bold. est: estimate; se: standard error; CI.lb: lower bound of the 95% confidence interval; CI.ub: upper bound of the 95% confidence interval; k_sp_: number of species included in the model; k_es_: number of effect sizes included in the model; Var_sp_: variance explained by variation between species; Var_phy_: variance explained by shared evolutionary history; Var_es_: variance associated with the residuals; Int: intercept (reference on which the comparison was made).

|  | **est** | **se** | **p** | **CI.lb** | **CI.ub** | **k_sp_** | **k_es_** | **Var_sp_** | **Var_phy_** | **Var_es_** |
| --- | --- | --- | --- | --- | --- | --- | --- | --- | --- | --- |
| Int (Aquatic) | 0.209 | 0.066 | 0.002 | 0.077 | 0.340 | 118 | 929 | 0.010 | 0.013 | 0.061 |
| **Terrestrial** | **-0.142** | **0.056** | **0.013** | **-0.253** | **-0.030** | 20 | 160 |  |  |  |

**TABLE S10:** **Results of the univariate meta-regression used to assess the difference in developmental plasticity estimates between taxonomic groups**. est: estimate; se: standard error; CI.lb: lower bound of the 95% confidence interval; CI.ub: upper bound of the 95% confidence interval; k_sp_: number of species included in the model; k_es_: number of effect sizes included in the model; Var_sp_: variance explained by variation between species; Var_phy_: variance explained by shared evolutionary history; Var_es_: variance associated with the residuals; Int: intercept (reference on which the comparison was made); A. inverts: aquatic invertebrates; T. inverts: terrestrial invertebrates.

|  | **est** | **se** | **p** | **CI.lb** | **CI.ub** | **k_sp_** | **k_es_** | **Var_sp_** | **Var_phy_** | **Var_es_** |
| --- | --- | --- | --- | --- | --- | --- | --- | --- | --- | --- |
| Int (Amphibians) | 0.208 | 0.154 | 0.179 | -0.096 | 0.513 | 14 | 71 | 0.009 | 0.024 | 0.061 |
| A. inverts | -0.018 | 0.176 | 0.919 | -0.365 | 0.329 | 31 | 221 |  |  |  |
| Fishes | 0.033 | 0.145 | 0.821 | -0.254 | 0.320 | 68 | 623 |  |  |  |
| Reptiles | -0.142 | 0.107 | 0.188 | -0.353 | 0.070 | 7 | 27 |  |  |  |
| T. inverts | -0.155 | 0.187 | 0.410 | -0.525 | 0.216 | 18 | 147 |  |  |  |
|  | **est** | **se** | **p** | **CI.lb** | **CI.ub** | **k_sp_** | **k_es_** | **Var_sp_** | **Var_phy_** | **Var_es_** |
| Int (A. inverts) | 0.190 | 0.113 | 0.095 | -0.033 | 0.414 | 31 | 221 |  |  |  |
| Amphibians | 0.018 | 0.176 | 0.919 | -0.329 | 0.365 | 14 | 71 |  |  |  |
| Fishes | 0.051 | 0.143 | 0.723 | -0.233 | 0.334 | 68 | 623 | 0.009 | 0.024 | 0.061 |
| Reptiles | -0.124 | 0.184 | 0.502 | -0.487 | 0.240 | 7 | 27 |  |  |  |
| T. inverts | -0.137 | 0.077 | 0.077 | -0.288 | 0.015 | 18 | 147 |  |  |  |
|  | **est** | **se** | **p** | **CI.lb** | **CI.ub** | **k_sp_** | **k_es_** | **Var_sp_** | **Var_phy_** | **Var_es_** |
| Int (Fishes) | 0.241 | 0.116 | 0.040 | 0.012 | 0.471 | 68 | 623 |  |  |  |
| Amphibians | -0.033 | 0.145 | 0.821 | -0.334 | 0.254 | 14 | 71 |  |  |  |
| A. inverts | -0.051 | 0.143 | 0.723 | -0.334 | 0.233 | 31 | 221 | 0.009 | 0.024 | 0.061 |
| Reptiles | -0.175 | 0.155 | 0.262 | -0.481 | 0.132 | 7 | 27 |  |  |  |
| T. inverts | -0.188 | 0.158 | 0.235 | -0.499 | 0.124 | 18 | 147 |  |  |  |
|  | **est** | **se** | **p** | **CI.lb** | **CI.ub** | **k_sp_** | **k_es_** | **Var_sp_** | **Var_phy_** | **Var_es_** |
| Int (Reptiles) | 0.067 | 0.163 | 0.684 | -0.256 | 0.340 | 7 | 27 |  |  |  |
| Amphibians | 0.142 | 0.107 | 0.188 | -0.070 | 0.353 | 14 | 71 |  |  |  |
| A. inverts | 0.124 | 0.184 | 0.502 | -0.240 | 0.487 | 31 | 221 | 0.009 | 0.024 | 0.061 |
| Fishes | 0.175 | 0.155 | 0.262 | -0.132 | 0.481 | 68 | 623 |  |  |  |
| T. inverts | -0.013 | 0.195 | 0.946 | -0.399 | 0.372 | 18 | 147 |  |  |  |
|  | **est** | **se** | **p** | **CI.lb** | **CI.ub** | **k_sp_** | **k_es_** | **Var_sp_** | **Var_phy_** | **Var_es_** |
| Int (T. inverts) | 0.054 | 0.129 | 0.678 | -0.201 | 0.308 | 18 | 147 |  |  |  |
| Amphibians | 0.155 | 0.187 | 0.410 | -0.216 | 0.525 | 14 | 71 |  |  |  |
| A. inverts | 0.137 | 0.077 | 0.077 | -0.015 | 0.288 | 31 | 221 | 0.009 | 0.024 | 0.061 |
| Fishes | 0.188 | 0.158 | 0.235 | -0.124 | 0.499 | 68 | 623 |  |  |  |
| Reptiles | 0.013 | 0.195 | 0.946 | -0.372 | 0.399 | 7 | 27 |  |  |  |

**TABLE S11:** **Results of the univariate meta-regression used to assess the difference in developmental plasticity estimates between initial and persistent designs.** Differences statistically different from zero were highlighted in bold. est: estimate; se: standard error; CI.lb: lower bound of the 95% confidence interval; CI.ub: upper bound of the 95% confidence interval; k_sp_: number of species included in the model; k_es_: number of effect sizes included in the model; Var_sp_: variance explained by variation between species; Var_phy_: variance explained by shared evolutionary history; Var_es_: variance associated with the residuals; Int: intercept (reference on which the comparison was made).

|  | **est** | **se** | **p** | **CI.lb** | **CI.ub** | **k_sp_** | **k_es_** | **Var_sp_** | **Var_phy_** | **Var_es_** |
| --- | --- | --- | --- | --- | --- | --- | --- | --- | --- | --- |
| Int (Initial) | 0.221 | 0.068 | 0.001 | 0.087 | 0.356 | 118 | 929 | 0.010 | 0.013 | 0.061 |
| **Persistent** | **-0.182** | **0.033** | **<0.0001** | **-0.246** | **-0.118** | 20 | 160 |  |  |  |

**TABLE S12:** **Results of the univariate meta-regression used to assess the difference in developmental plasticity estimates between the different types of experimental designs**. est: estimate; se: standard error; CI.lb: lower bound of the 95% confidence interval; CI.ub: upper bound of the 95% confidence interval; k_sp_: number of species included in the model; k_es_: number of effect sizes included in the model; Var_sp_: variance explained by variation between species; Var_phy_: variance explained by shared evolutionary history; Var_es_: variance associated with the residuals; Int: intercept (reference on which the comparison was made). Designs are outlined in Fig 2.

|  | **est** | **se** | **p** | **CI.lb** | **CI.ub** | **k_sp_** | **k_es_** | **Var_sp_** | **Var_phy_** | **Var_es_** |
| --- | --- | --- | --- | --- | --- | --- | --- | --- | --- | --- |
| Int (Design A) | 0.222 | 0.069 | 0.001 | 0.088 | 0.356 | 109 | 700 | 0.006 | 0.014 | 0.060 |
| Design B | -0.105 | 0.073 | 0.149 | -0.247 | 0.038 | 5 | 20 |  |  |  |
| Design C | 0.028 | 0.042 | 0.500 | -0.054 | 0.111 | 13 | 146 |  |  |  |
| **Design D** | **-0.315** | **0.051** | **<0.0001** | **-0.416** | **-0.214** | 6 | 92 |  |  |  |
| **Design E** | **-0.116** | **0.053** | **0.029** | **-0.220** | **-0.012** | 7 | 76 |  |  |  |
| **Design F** | **-0.109** | **0.050** | **0.031** | **-0.208** | **-0.010** | 11 | 55 |  |  |  |
|  | **est** | **se** | **p** | **CI.lb** | **CI.ub** | **k_sp_** | **k_es_** | **Var_sp_** | **Var_phy_** | **Var_es_** |
| Int (Design B) | 0.117 | 0.096 | 0.227 | -0.074 | 0.308 | 5 | 20 |  |  |  |
| Design A | 0.105 | 0.073 | 0.149 | -0.038 | 0.247 | 109 | 700 |  |  |  |
| Design C | 0.133 | 0.079 | 0.092 | -0.022 | 0.288 | 13 | 146 | 0.006 | 0.014 | 0.060 |
| **Design D** | **-0.210** | **0.087** | **0.016** | **-0.381** | **-0.039** | 6 | 92 |  |  |  |
| Design E | -0.011 | 0.089 | 0.898 | -0.185 | 0.162 | 7 | 76 |  |  |  |
| Design F | -0.004 | 0.086 | 0.964 | -0.173 | 0.165 | 11 | 55 |  |  |  |
|  | **est** | **se** | **p** | **CI.lb** | **CI.ub** | **k_sp_** | **k_es_** | **Var_sp_** | **Var_phy_** | **Var_es_** |
| Int (Design C) | 0.250 | 0.076 | 0.001 | 0.101 | 0.400 | 68 | 623 |  |  |  |
| Design A | -0.028 | 0.042 | 0.500 | -0.111 | 0.054 | 109 | 700 |  |  |  |
| Design B | -0.133 | 0.079 | 0.092 | -0.288 | 0.022 | 5 | 20 | 0.006 | 0.014 | 0.060 |
| **Design D** | **-0.343** | **0.064** | **<0.0001** | **-0.468** | **-0.219** | 6 | 92 |  |  |  |
| **Design E** | **-0.145** | **0.063** | **0.022** | **-0.268** | **-0.021** | 7 | 76 |  |  |  |
| **Design F** | **-0.137** | **0.062** | **0.027** | **-0.289** | **-0.016** | 11 | 55 |  |  |  |
|  | **est** | **se** | **p** | **CI.lb** | **CI.ub** | **k_sp_** | **k_es_** | **Var_sp_** | **Var_phy_** | **Var_es_** |
| Int (Design D) | -0.093 | 0.082 | -0.260 | -0.255 | 0.070 | 6 | 92 |  |  |  |
| **Design A** | **0.315** | **0.051** | **<0.0001** | **0.214** | **0.416** | 109 | 700 |  |  |  |
| **Design B** | **0.210** | **0.087** | **0.016** | **0.039** | **0.038** | 5 | 20 | 0.006 | 0.014 | 0.060 |
| **Design C** | **0.343** | **0.064** | **<0.0001** | **0.219** | **0.468** | 13 | 146 |  |  |  |
| **Design E** | **0.199** | **0.068** | **0.004** | **0.065** | **0.333** | 7 | 76 |  |  |  |
| **Design F** | **0.206** | **0.070** | **0.003** | **0.069** | **0.343** | 11 | 55 |  |  |  |
|  | **est** | **se** | **p** | **CI.lb** | **CI.ub** | **k_sp_** | **k_es_** | **Var_sp_** | **Var_phy_** | **Var_es_** |
| Int (Design E) | 0.106 | 0.083 | 0.207 | -0.059 | 0.270 | 7 | 76 |  |  |  |
| **Design A** | **0.116** | **0.053** | **0.029** | **0.012** | **0.220** | 109 | 700 |  |  |  |
| Design B | 0.011 | 0.089 | 0.898 | -0.162 | 0.185 | 5 | 20 | 0.006 | 0.014 | 0.060 |
| **Design C** | **0.145** | **0.063** | **0.022** | **0.021** | **0.268** | 13 | 146 |  |  |  |
| **Design D** | **-0.199** | **0.068** | **0.004** | **-0.333** | **-0.065** | 6 | 92 |  |  |  |
| Design F | 0.008 | 0.068 | 0.913 | -0.127 | 0.141 | 11 | 55 |  |  |  |
|  | **est** | **se** | **p** | **CI.lb** | **CI.ub** | **k_sp_** | **k_es_** | **Var_sp_** | **Var_phy_** | **Var_es_** |
| Int (Design F) | 0.113 | 0.082 | 0.168 | -0.048 | 0.274 | 11 | 55 |  |  |  |
| **Design A** | **0.109** | **0.050** | **0.031** | **0.010** | **0.208** | 109 | 700 |  |  |  |
| Design B | 0.004 | 0.086 | 0.964 | -0.165 | 0.173 | 5 | 20 | 0.006 | 0.014 | 0.060 |
| **Design C** | **0.137** | **0.062** | **0.027** | **0.016** | **0.259** | 13 | 146 |  |  |  |
| **Design D** | **-0.206** | **0.070** | **0.003** | **-0.343** | **-0.069** | 6 | 92 |  |  |  |
| Design E | -0.008 | 0.068 | 0.913 | -0.141 | 0.127 | 7 | 76 |  |  |  |

**TABLE S13:** **Results of the univariate meta-regression used to assess the difference in developmental plasticity estimates between metric types**. Differences statistically different from zero were highlighted in bold. est: estimate; se: standard error; CI.lb: lower bound of the 95% confidence interval; CI.ub: upper bound of the 95% confidence interval; k_sp_: number of species included in the model; k_es_: number of effect sizes included in the model; Var_sp_: variance explained by variation between species; Var_phy_: variance explained by shared evolutionary history; Var_es_: variance associated with the residuals; Int: intercept (reference on which the comparison was made); CTmax: critical thermal maximum. LT50: median lethal temperature.

|  | **est** | **se** | **p** | **CI.lb** | **CI.ub** | **k_sp_** | **k_es_** | **Var_sp_** | **Var_phy_** | **Var_es_** |
| --- | --- | --- | --- | --- | --- | --- | --- | --- | --- | --- |
| Int (CTmax) | 0.197 | 0.090 | 0.030 | 0.019 | 0.374 | 118 | 863 | 0.010 | 0.026 | 0.060 |
| LT50 | -0.032 | 0.028 | 0.246 | -0.086 | 0.022 | 35 | 226 |  |  |  |

**TABLE S14:** **Results of univariate categorical meta-regressions accounting for residual heteroscedasticity**. Heteroscedasticity was modelled at the effect size level. Coefficients statistically different from zero were highlighted in bold. est: estimate; se: standard error; CI.lb: lower bound of the 95% confidence interval; CI.ub: upper bound of the 95% confidence interval; PI.lb: lower bound of the 95% prediction interval; PI.ub: upper bound of the 95% prediction interval; k_sp_: number of species included in the model; k_es_: number of effect sizes included in the model; Var_sp_: variance explained by variation between species. Var_phy_: variance explained by shared evolutionary history. Var_es_: variance associated with the residuals. A. inverts: aquatic invertebrates; T. inverts: terrestrial invertebrates; CTmax: critical thermal maximum; LT50: median lethal temperature. Designs are outlined in Fig. 2.

|  | **est** | **se** | **p** | **CI.lb** | **CI.ub** | **PI.lb** | **PI.ub** | **k_sp_** | **k_es_** | **Var_sp_** | **Var_phy_** | **Var_es_** |
| --- | --- | --- | --- | --- | --- | --- | --- | --- | --- | --- | --- | --- |
| **Aquatic** | **0.209** | **0.066** | **0.002** | **0.079** | **0.338** | -0.410 | 0.827 | 118 | 929 | 0.009 | 0.013 | 0.072 |
| Terrestrial | 0.060 | 0.076 | 0.436 | -0.091 | 0.210 | -0.315 | 0.434 | 20 | 160 |  |  | 0.008 |
| Amphibians | 0.197 | 0.176 | 0.266 | -0.152 | 0.545 | -0.807 | 1.200 | 14 | 71 | 0.009 | 0.024 | 0.188 |
| A. inverts | 0.199 | 0.129 | 0.124 | -0.055 | 0.454 | -0.665 | 1.063 | 31 | 221 |  |  | 0.136 |
| **Fishes** | **0.254** | **0.127** | **0.047** | **0.004** | **0.504** | -0.331 | 0.839 | 68 | 623 |  |  | 0.033 |
| Reptiles | 0.070 | 0.173 | 0.686 | -0.273 | 0.413 | -0.506 | 0.647 | 7 | 27 |  |  | 0.016 |
| T. inverts | 0.049 | 0.141 | 0.730 | -0.230 | 0.328 | -0.457 | 0.555 | 18 | 147 |  |  | 0.007 |
| **Initial** | **0.224** | **0.071** | **0.002** | **0.086** | **0.363** | -0.383 | 0.832 | 121 | 866 | 0.006 | 0.016 | 0.070 |
| Persistent | 0.049 | 0.073 | 0.508 | -0.095 | 0.192 | -0.380 | 0.477 | 23 | 223 |  |  | 0.021 |
| **Design A** | **0.230** | **0.074** | **0.002** | **0.850** | **0.376** | -0.403 | 0.864 | 109 | 700 | 0.006 | 0.017 | 0.075 |
| Design B | 0.098 | 0.157 | 0.532 | -0.210 | 0.406 | -1.093 | 1.290 | 5 | 20 |  |  | 0.321 |
| **Design C** | **0.250** | **0.078** | **0.001** | **0.097** | **0.404** | -0.166 | 0.666 | 13 | 146 |  |  | 0.016 |
| Design D | -0.082 | 0.085 | 0.336 | -0.248 | 0.085 | -0.585 | 0.421 | 6 | 92 |  |  | 0.035 |
| Design E | 0.118 | 0.083 | 0.153 | -0.044 | 0.280 | -0.292 | 0.528 | 7 | 76 |  |  | 0.013 |
| Design F | 0.102 | 0.078 | 0.195 | -0.052 | 0.255 | -0.276 | 0.479 | 11 | 55 |  |  | 0.007 |
| **CTmax** | **0.195** | **0.091** | **0.032** | **0.017** | **0.372** | -0.435 | 0.829 | 118 | 863 | 0.010 | 0.026 | 0.065 |
| LT50 | 0.162 | 0.093 | 0.081 | -0.020 | 0.343 | -0.469 | 0.798 | 35 | 226 |  |  | 0.041 |

**TABLE S15:** **Results of the univariate meta-regression used to assess the difference in developmental plasticity between aquatic and terrestrial animals, including the modelling of residual heteroscedasticity**. Differences statistically different from zero were highlighted in bold. est: estimate; se: standard error; CI.lb: lower bound of the 95% confidence interval; CI.ub: upper bound of the 95% confidence interval; k_sp_: number of species included in the model; k_es_: number of effect sizes included in the model; Var_sp_: variance explained by variation between species; Var_phy_: variance explained by shared evolutionary history; Var_es_: variance associated with the residuals; Int: intercept (reference on which the comparison was made).

|  | **est** | **se** | **p** | **CI.lb** | **CI.ub** | **k_sp_** | **k_es_** | **Var_sp_** | **Var_phy_** | **Var_es_** |
| --- | --- | --- | --- | --- | --- | --- | --- | --- | --- | --- |
| Int (Aquatic) | 0.209 | 0.066 | 0.002 | 0.079 | 0.338 | 118 | 929 | 0.009 | 0.013 | 0.072 |
| **Terrestrial** | **-0.142** | **0.049** | **0.003** | **-0.246** | **-0.052** | 20 | 160 |  |  | 0.008 |

**TABLE S16:** **Results of the univariate meta-regression used to assess the difference in developmental plasticity estimates between taxonomic groups, including the modelling of residual heteroscedasticity**. est: estimate; se: standard error; CI.lb: lower bound of the 95% confidence interval; CI.ub: upper bound of the 95% confidence interval; k_sp_: number of species included in the model; k_es_: number of effect sizes included in the model; Var_sp_: variance explained by variation between species; Var_phy_: variance explained by shared evolutionary history; Var_es_: variance associated with the residuals; Int: intercept (reference on which the comparison was made); A. inverts: aquatic invertebrates; T. inverts: terrestrial invertebrates.

|  | **est** | **se** | **p** | **CI.lb** | **CI.ub** | **k_sp_** | **k_es_** | **Var_sp_** | **Var_phy_** | **Var_es_** |
| --- | --- | --- | --- | --- | --- | --- | --- | --- | --- | --- |
| Int (Amphibians) | 0.197 | 0.176 | 0.266 | -0.152 | 0.545 | 14 | 71 | 0.009 | 0.030 | 0.188 |
| A. inverts | 0.003 | 0.203 | 0.990 | -0.398 | 0.403 | 31 | 221 |  |  | 0.136 |
| Fishes | 0.057 | 0.165 | 0.729 | -0.268 | 0.383 | 68 | 623 |  |  | 0.033 |
| Reptiles | -0.126 | 0.114 | 0.269 | -0.352 | 0.099 | 7 | 27 |  |  | 0.016 |
| T. inverts | -0.148 | 0.212 | 0.487 | -0.567 | 0.272 | 18 | 147 |  |  | 0.007 |
|  | **est** | **se** | **p** | **CI.lb** | **CI.ub** | **k_sp_** | **k_es_** | **Var_sp_** | **Var_phy_** | **Var_es_** |
| Int (A. inverts) | 0.199 | 0.129 | 0.124 | -0.055 | 0.454 | 31 | 221 | 0.009 | 0.030 | 0.136 |
| Amphibians | -0.003 | 0.203 | 0.990 | -0.403 | 0.398 | 14 | 71 |  |  | 0.188 |
| Fishes | 0.055 | 0.161 | 0.736 | -0.265 | 0.374 | 68 | 623 |  |  | 0.033 |
| Reptiles | -0.129 | 0.200 | 0.521 | -0.525 | 0.267 | 7 | 27 |  |  | 0.016 |
| T. inverts | -0.150 | 0.079 | 0.060 | -0.307 | 0.007 | 18 | 147 |  |  | 0.007 |
|  | **est** | **se** | **p** | **CI.lb** | **CI.ub** | **k_sp_** | **k_es_** | **Var_sp_** | **Var_phy_** | **Var_es_** |
| Int (Fishes) | 0.254 | 0.167 | 0.047 | 0.004 | 0.504 | 68 | 623 | 0.009 | 0.030 | 0.033 |
| Amphibians | -0.057 | 0.165 | 0.729 | -0.383 | 0.268 | 14 | 71 |  |  | 0.188 |
| A. inverts | -0.055 | 0.161 | 0.736 | -0.374 | 0.265 | 31 | 221 |  |  | 0.136 |
| Reptiles | -0.184 | 0.162 | 0.258 | -0.503 | 0.136 | 7 | 27 |  |  | 0.016 |
| T. inverts | -0.206 | 0.173 | 0.239 | -0.547 | 0.138 | 18 | 147 |  |  | 0.007 |
|  | **est** | **se** | **p** | **CI.lb** | **CI.ub** | **k_sp_** | **k_es_** | **Var_sp_** | **Var_phy_** | **Var_es_** |
| Int (Reptiles) | 0.070 | 0.173 | 0.686 | -0.273 | 0.413 | 7 | 27 | 0.009 | 0.030 | 0.016 |
| Amphibians | 0.126 | 0.114 | 0.269 | -0.099 | 0.352 | 14 | 71 |  |  | 0.188 |
| A. inverts | 0.129 | 0.200 | 0.521 | -0.267 | 0.525 | 31 | 221 |  |  | 0.136 |
| Fishes | 0.184 | 0.162 | 0.258 | -0.136 | 0.503 | 68 | 623 |  |  | 0.033 |
| T. inverts | -0.21 | 0.210 | 0.919 | -0.436 | 0.394 | 18 | 147 |  |  | 0.007 |
|  | **est** | **se** | **p** | **CI.lb** | **CI.ub** | **k_sp_** | **k_es_** | **Var_sp_** | **Var_phy_** | **Var_es_** |
| Int (T. inverts) | 0.049 | 0.141 | 0.730 | -0.230 | 0.328 | 18 | 147 | 0.009 | 0.030 | 0.007 |
| Amphibians | 0.148 | 0.212 | 0.487 | -0.272 | 0.567 | 14 | 71 |  |  | 0.188 |
| A. inverts | 0.150 | 0.079 | 0.060 | -0.138 | 0.307 | 31 | 221 |  |  | 0.136 |
| Fishes | 0.205 | 0.173 | 0.239 | -0.138 | 0.547 | 68 | 623 |  |  | 0.033 |
| Reptiles | 0.021 | 0.210 | 0.919 | -0.394 | 0.436 | 7 | 27 |  |  | 0.016 |

**TABLE S17:** **Results of the univariate meta-regression used to assess the difference in developmental plasticity estimates between initial and persistent designs, including the modelling of residual heteroscedasticity.** Differences statistically different from zero were highlighted in bold. est: estimate; se: standard error; CI.lb: lower bound of the 95% confidence interval; CI.ub: upper bound of the 95% confidence interval; k_sp_: number of species included in the model; k_es_: number of effect sizes included in the model; Var_sp_: variance explained by variation between species; Var_phy_: variance explained by shared evolutionary history; Var_es_: variance associated with the residuals; Int: intercept (reference on which the comparison was made).

|  | **est** | **se** | **p** | **CI.lb** | **CI.ub** | **k_sp_** | **k_es_** | **Var_sp_** | **Var_phy_** | **Var_es_** |
| --- | --- | --- | --- | --- | --- | --- | --- | --- | --- | --- |
| Int (Initial) | 0.224 | 0.071 | 0.002 | 0.085 | 0.364 | 118 | 929 | 0.006 | 0.016 | 0.070 |
| **Persistent** | **-0.176** | **0.028** | **<0.0001** | **-0.231** | **-0.121** | 20 | 160 |  |  | 0.021 |

**TABLE S18:** **Results of the univariate meta-regression used to assess the difference in developmental plasticity estimates between the different types of experimental designs, including the modelling of residual heteroscedasticity**. est: estimate; se: standard error; CI.lb: lower bound of the 95% confidence interval; CI.ub: upper bound of the 95% confidence interval; k_sp_: number of species included in the model; k_es_: number of effect sizes included in the model; Var_sp_: variance explained by variation between species; Var_phy_: variance explained by shared evolutionary history; Var_es_: variance associated with the residuals; Int: intercept (reference on which the comparison was made). Designs are outlined in Fig 2.

|  | **est** | **se** | **p** | **CI.lb** | **CI.ub** | **k_sp_** | **k_es_** | **Var_sp_** | **Var_phy_** | **Var_es_** |
| --- | --- | --- | --- | --- | --- | --- | --- | --- | --- | --- |
| Int (Design A) | 0.230 | 0.074 | 0.002 | 0.084 | 0.377 | 109 | 700 | 0.006 | 0.017 | 0.075 |
| Design B | -0.132 | 0.141 | 0.348 | -0.408 | 0.144 | 5 | 20 |  |  | 0.321 |
| Design C | 0.020 | 0.037 | 0.589 | -0.052 | 0.092 | 13 | 146 |  |  | 0.016 |
| **Design D** | **-0.312** | **0.048** | **<0.0001** | **-0.406** | **-0.218** | 6 | 92 |  |  | 0.035 |
| **Design E** | **-0.112** | **0.043** | **0.009** | **-0.196** | **-0.028** | 7 | 76 |  |  | 0.013 |
| **Design F** | **-0.129** | **0.034** | **<0.001** | **-0.196** | **-0.062** | 11 | 55 |  |  | 0.007 |
|  | **est** | **se** | **p** | **CI.lb** | **CI.ub** | **k_sp_** | **k_es_** | **Var_sp_** | **Var_phy_** | **Var_es_** |
| Int (Design B) | 0.098 | 0.157 | 0.533 | -0.212 | 0.409 | 5 | 20 | 0.006 | 0.017 | 0.321 |
| Design A | 0.132 | 0.141 | 0.348 | -0.144 | 0.408 | 109 | 700 |  |  | 0.075 |
| Design C | 0.152 | 0.142 | 0.284 | -0.127 | 0.431 | 13 | 146 |  |  | 0.016 |
| Design D | -0.180 | 0.147 | 0.222 | -0.469 | 0.109 | 6 | 92 |  |  | 0.035 |
| Design E | 0.020 | 0.146 | 0.891 | -0.266 | 0.306 | 7 | 76 |  |  | 0.013 |
| Design F | 0.003 | 0.143 | 0.981 | -0.277 | 0.283 | 11 | 55 |  |  | 0.007 |
|  | **est** | **se** | **p** | **CI.lb** | **CI.ub** | **k_sp_** | **k_es_** | **Var_sp_** | **Var_phy_** | **Var_es_** |
| Int (Design C) | 0.250 | 0.078 | 0.002 | 0.096 | 0.405 | 68 | 623 | 0.006 | 0.017 | 0.016 |
| Design A | -0.020 | 0.037 | 0.589 | -0.092 | 0.052 | 109 | 700 |  |  | 0.075 |
| Design B | -0.152 | 0.142 | 0.284 | -0.431 | 0.127 | 5 | 20 |  |  | 0.321 |
| **Design D** | **-0.332** | **0.056** | **<0.0001** | **-0.442** | **-0.222** | 6 | 92 |  |  | 0.035 |
| **Design E** | **-0.132** | **0.051** | **0.010** | **-0.233** | **-0.032** | 7 | 76 |  |  | 0.013 |
| **Design F** | **-0.149** | **0.045** | **0.001** | **-0.237** | **-0.060** | 11 | 55 |  |  | 0.007 |
|  | **est** | **se** | **p** | **CI.lb** | **CI.ub** | **k_sp_** | **k_es_** | **Var_sp_** | **Var_phy_** | **Var_es_** |
| Int (Design D) | -0.082 | 0.085 | 0.337 | -0.250 | 0.086 | 6 | 92 | 0.006 | 0.017 | 0.035 |
| **Design A** | **0.312** | **0.048** | **<0.0001** | **0.218** | **0.406** | 109 | 700 |  |  | 0.075 |
| Design B | 0.180 | 0.147 | 0.222 | -0.109 | 0.469 | 5 | 20 |  |  | 0.321 |
| **Design C** | **0.332** | **0.056** | **<0.0001** | **0.222** | **0.442** | 13 | 146 |  |  | 0.016 |
| **Design E** | **0.200** | **0.054** | **<0.001** | **0.095** | **0.305** | 7 | 76 |  |  | 0.013 |
| **Design F** | **0.183** | **0.055** | **<0.001** | **0.075** | **0.291** | 11 | 55 |  |  | 0.007 |
|  | **est** | **se** | **p** | **CI.lb** | **CI.ub** | **k_sp_** | **k_es_** | **Var_sp_** | **Var_phy_** | **Var_es_** |
| Int (Design E) | 0.118 | 0.083 | 0.155 | -0.045 | 0.281 | 7 | 76 | 0.006 | 0.017 | 0.013 |
| **Design A** | **0.112** | **0.043** | **0.009** | **0.028** | **0.196** | 109 | 700 |  |  | 0.075 |
| Design B | -0.02 | 0.156 | 0.891 | -0.306 | 0.266 | 5 | 20 |  |  | 0.321 |
| **Design C** | **0.132** | **0.051** | **0.010** | **0.032** | **0.233** | 13 | 146 |  |  | 0.016 |
| **Design D** | **-0.200** | **0.054** | **<0.001** | **-0.305** | **-0.095** | 6 | 92 |  |  | 0.035 |
| Design F | -0.017 | 0.049 | 0.733 | -0.112 | 0.079 | 11 | 55 |  |  | 0.007 |
|  | **est** | **se** | **p** | **CI.lb** | **CI.ub** | **k_sp_** | **k_es_** | **Var_sp_** | **Var_phy_** | **Var_es_** |
| Int (Design F) | 0.102 | 0.078 | 0.197 | -0.054 | 0.257 | 11 | 55 | 0.006 | 0.017 | 0.007 |
| **Design A** | **0.189** | **0.034** | **<0.001** | **0.062** | **0.196** | 109 | 700 |  |  | 0.075 |
| Design B | -0.003 | 0.143 | 0.981 | -0.283 | 0.277 | 5 | 20 |  |  | 0.321 |
| **Design C** | **0.149** | **0.045** | **0.001** | **0.060** | **0.237** | 13 | 146 |  |  | 0.016 |
| **Design D** | **-0.183** | **0.055** | **<0.001** | **-0.291** | **-0.075** | 6 | 92 |  |  | 0.035 |
| Design E | 0.017 | 0.049 | 0.733 | -0.079 | 0.112 | 7 | 76 |  |  | 0.013 |

**TABLE S19:** **Results of the univariate meta-regression used to assess the difference in developmental plasticity estimates between metric types, including the modelling of residual heteroscedasticity**. Differences statistically different from zero were highlighted in bold. est: estimate; se: standard error; CI.lb: lower bound of the 95% confidence interval; CI.ub: upper bound of the 95% confidence interval; k_sp_: number of species included in the model; k_es_: number of effect sizes included in the model; Var_sp_: variance explained by variation between species; Var_phy_: variance explained by shared evolutionary history; Var_es_: variance associated with the residuals; Int: intercept (reference on which the comparison was made); CTmax: critical thermal maximum. LT50: median lethal temperature.

|  | **est** | **se** | **p** | **CI.lb** | **CI.ub** | **k_sp_** | **k_es_** | **Var_sp_** | **Var_phy_** | **Var_es_** |
| --- | --- | --- | --- | --- | --- | --- | --- | --- | --- | --- |
| Int (CTmax) | 0.195 | 0.091 | 0.034 | 0.015 | 0.374 | 118 | 863 | 0.010 | 0.026 | 0.065 |
| LT50 | -0.033 | 0.026 | 0.200 | -0.083 | 0.017 | 35 | 226 |  |  | 0.041 |

**TABLE S20:** **Comparisons of models modelling and ignoring residual heteroscedasticity.** AICc: Akaike information criterion corrected for small sample sizes. Comparisons are made between models modelling (with) and not modelling (without) residual heteroscedasticity for each moderator category.

|  | **AICc** |
| --- | --- |
| Habitat (without) | 456.1 |
| Habitat (with) | 319.6 |
| Taxonomy (without) | 456.1 |
| Taxonomy (with) | 133.3 |
| Persistence (without) | 435.9 |
| Persistence (with) | 380.4 |
| Design (without) | 430.0 |
| Design (with) | 274.2 |
| Metric (without) | 461.5 |
| Metric (with) | 454.5 |

**TABLE S21:** **Results of the meta-analytic intercept model adjusted for the over-representation of aquatic animals**. est: estimate; CI.lb: lower bound of the 95% confidence interval; CI.ub: upper bound of the 95% confidence interval; PI.lb: lower bound of the 95% prediction interval; PI.ub: upper bound of the 95% prediction interval; k_species_: number of species included in the model; k_es_: number of effect sizes included in the model.

|  | **est** | **CI.lb** | **CI.ub** | **PI.lb** | **PI.ub** | **k_sp_** | **k_es_** |
| --- | --- | --- | --- | --- | --- | --- | --- |
| Overall mean | 0.134 | 0.002 | 0.266 | -0.455 | 0.723 | 138 | 1089 |

**TABLE S22:** **Results of the univariate meta-regression with continuous moderators**. Moderators were centred to zero mean. est: estimate; se: standard error; CI.lb: lower bound of the 95% confidence interval; CI.ub: upper bound of the 95% confidence interval; k_sp_: number of species included in the model; k_es_: number of effect sizes included in the model; Var_sp_: variance explained by variation between species; Var_phy_: variance explained by shared evolutionary history; Var_es_: variance associated with the residuals; Time common temp: duration of re-acclimation to a common garden condition after the initial acclimation. Relative time common temp: duration of re-acclimation to a common garden condition relative to a proxy of life span (Time common temp / Age at sexual maturity).

|  | **est** | **se** | **p** | **CI.lb** | **CI.ub** | **k_sp_** | **k_es_** | **Var_sp_** | **Var_phy_** | **Var_es_** |
| --- | --- | --- | --- | --- | --- | --- | --- | --- | --- | --- |
| Intercept | 0.050 | 0.088 | 0.576 | -0.134 | 0.234 | 21 | 204 | 0.001 | 0.022 | 0.023 |
| Time common temp | -0.009 | 0.012 | 0.491 | -0.034 | 0.016 |  |  |  |  |  |
| Intercept | 0.050 | 0.103 | 0.631 | -0.165 | 0.266 | 20 | 202 | 0.001 | 0.031 | 0.023 |
| Relative time common temp | 0.001 | 0.0126 | 0.970 | -0.024 | 0.025 |  |  |  |  |  |
| Intercept | 0.212 | 0.105 | 0.045 | 0.005 | 0.419 | 114 | 855 | 0.007 | 0.035 | 0.064 |
| Heating rate | 0.019 | 0.033 | 0.553 | -0.045 | 0.084 |  |  |  |  |  |

**TABLE S23:** **Results of meta-regressions used to assess the difference in developmental plasticity estimates between different types of experimental designs**, **after controlling for re-acclimation times.** Moderators were right-skewed and were therefore ln-transformed. Moderators were also centred to zero mean. est: estimate; se: standard error; CI.lb: lower bound of the 95% confidence interval; CI.ub: upper bound of the 95% confidence interval; k_sp_: number of species included in the model; k_es_: number of effect sizes included in the model; Var_sp_: variance explained by variation between species; Var_phy_: variance explained by shared evolutionary history; Var_es_: variance associated with the residuals; Time common temp: duration of re-acclimation to a common garden condition after the initial acclimation; Relative time common temp: duration of re-acclimation to a common garden condition relative to a proxy of life span (Time common temp / Age at sexual maturity).

|  | **est** | **se** | **p** | **CI.lb** | **CI.ub** | **k_sp_** | **k_es_** | **Var_sp_** | **Var_phy_** | **Var_es_** |
| --- | --- | --- | --- | --- | --- | --- | --- | --- | --- | --- |
| **Intercept** | **0.075** | **0.035** | **0.049** | **0.001** | **0.150** | 21 | 204 | 0.004 | 0.001 | 0.022 |
| Time common temp | -0.010 | 0.012 | 0.415 | -0.034 | 0.014 |  |  |  |  |  |
| **Design D** | **-0.155** | **0.051** | **0.003** | **-0.025** | **-0.054** |  |  |  |  |  |
| Design E | 0.017 | 0.051 | 0.736 | -0.084 | 0.118 |  |  |  |  |  |
|  | **est** | **se** | **p** | **CI.lb** | **CI.ub** | **k_sp_** | **k_es_** | **Var_sp_** | **Var_phy_** | **Var_es_** |
| **I**ntercept | 0.076 | 0.037 | 0.058 | -0.003 | 0.156 | 20 | 202 | 0.005 | 0.001 | 0.022 |
| Relative time common temp | -0.002 | 0.010 | 0.883 | -0.022 | 0.019 |  |  |  |  |  |
| **Design D** | **-0.177** | **0.055** | **0.002** | **-0.285** | **-0.069** |  |  |  |  |  |
| Design E | 0.021 | 0.054 | 0.701 | -0.085 | 0.126 |  |  |  |  |  |

**TABLE S24:** **Characteristics of the best set of models identified (ΔAICc ≤ 2).** df: degrees of freedom; AICc: Aikaike Information Criterion corrected for small sample sizes; ΔAICc: difference in AICc from the best model; weight: AICc weight; R^2^: percentage of variation explained by the model.

|  | **Model rank** | | | | |
| --- | --- | --- | --- | --- | --- |
|  | 1 | 2 | 3 | 4 | 5 |
| df | 11 | 10 | 10 | 9 | 14 |
| AICc | 431.7 | 432.6 | 433.3 | 433.3 | 433.4 |
| ΔAICc | 0 | 0.89 | 1.53 | 1.56 | 1.68 |
| weight | 0.334 | 0.214 | 0.156 | 0.153 | 0.144 |
| R^2^ | 0.104 | 0.103 | 0.099 | 0.099 | 0.112 |

**TABLE S25:** **Importance of the moderator variables in the best set of models.**

|  | **Sum of weights** | **Number of models containing the variable** |
| --- | --- | --- |
| Design | 1 | 5 |
| Metric | 0.63 | 3 |
| Habitat | 0.55 | 2 |
| Taxonomy | 0.14 | 1 |

**TABLE S26:** **Results of the five best models**. Coefficients statistically different from zero were highlighted in bold. est: estimate; se: standard error; CI.lb: lower bound of the 95% confidence interval; CI.ub: upper bound of the 95% confidence interval; k_sp_: number of species included in the model; k_es_: number of effect sizes included in the model; Var_sp_: variance explained by variation between species; Var_phy_: variance explained by shared evolutionary history; Var_es_: variance associated with the residuals; Int: intercept (reference on which the comparison was made); LT50: median lethal temperature; A. inverts: aquatic invertebrates; T. inverts: terrestrial invertebrates. Designs are outlined in Fig 2.

|  | **Best model** | | | | | | | |
| --- | --- | --- | --- | --- | --- | --- | --- | --- |
|  | **est** | **se** | **p** | **CI.lb** | **CI.ub** | **Var_sp_** | **Var_phy_** | **Var_es_** |
| **Intercept** | **0.237** | **0.058** | **<0.0001** | **0.122** | **0.352** | 0.006 | 0.010 | 0.060 |
| Terrestrial | -0.063 | 0.053 | 0.238 | -0.169 | 0.042 |  |  |  |
| LT50 | -0.042 | 0.028 | 0.131 | -0.096 | 0.012 |  |  |  |
| Design B | -0.082 | 0.074 | 0.271 | -0.228 | 0.064 |  |  |  |
| Design C | 0.032 | 0.042 | 0.452 | -0.051 | 0.115 |  |  |  |
| **Design D** | **-0.304** | **0.053** | **<0.0001** | **-0.408** | **-0.199** |  |  |  |
| **Design E** | **-0.114** | **0.054** | **0.041** | **-0.218** | **-0.005** |  |  |  |
| **Design F** | **-0.115** | **0.051** | **0.023** | **-0.214** | **-0.016** |  |  |  |
|  | **Second best model** | | | | | | | |
|  | **est** | **se** | **p** | **CI.lb** | **CI.ub** | **Var_sp_** | **Var_phy_** | **Var_es_** |
| **Intercept** | **0.228** | **0.060** | **<0.001** | **0.111** | **0.346** | 0.006 | 0.010 | 0.060 |
| Terrestrial | 0.057 | 0.054 | 0.285 | -0.163 | 0.048 |  |  |  |
| Design B | -0.107 | 0.073 | 0.141 | -0.249 | 0.036 |  |  |  |
| Design C | 0.028 | 0.042 | 0.514 | -0.055 | 0.110 |  |  |  |
| **Design D** | **-0.301** | **0.053** | **<0.0001** | **-0.406** | **-0.197** |  |  |  |
| Design E | -0.106 | 0.054 | 0.051 | -0.212 | 0.001 |  |  |  |
| **Design F** | **-0.108** | **0.050** | **0.032** | **-0.206** | **-0.009** |  |  |  |
|  | **Third best model** | | | | | | | |
|  | **est** | **se** | **p** | **CI.lb** | **CI.ub** | **Var_sp_** | **Var_phy_** | **Var_es_** |
| **Intercept** | **0.230** | **0.069** | **0.001** | **0.093** | **0.366** | 0.006 | 0.014 | 0.060 |
| LT50 | -0.040 | 0.028 | 0.149 | -0.094 | 0.014 |  |  |  |
| Design B | -0.081 | 0.074 | 0.278 | -0.227 | 0.065 |  |  |  |
| Design C | 0.033 | 0.042 | 0.432 | -0.050 | 0.116 |  |  |  |
| **Design D** | **-0.318** | **0.051** | **<0.0001** | **-0.419** | **-0.217** |  |  |  |
| **Design E** | **-0.122** | **0.053** | **0.022** | **-0.226** | **-0.017** |  |  |  |
| **Design F** | **-0.116** | **0.051** | **0.022** | **-0.215** | **-0.017** |  |  |  |
|  | **Fourth best model** | | | | | | | |
|  | **est** | **se** | **p** | **CI.lb** | **CI.ub** | **Var_sp_** | **Var_phy_** | **Var_es_** |
| **Intercept** | **0.222** | **0.068** | **0.001** | **0.088** | **0.356** | 0.006 | 0.014 | 0.060 |
| Design B | -0.105 | 0.073 | 0.149 | -0.247 | 0.038 |  |  |  |
| Design C | 0.028 | 0.042 | 0.500 | -0.054 | 0.111 |  |  |  |
| **Design D** | **-0.315** | **0.051** | **<0.0001** | **-0.416** | **-0.214** |  |  |  |
| **Design E** | **-0.116** | **0.053** | **0.029** | **-0.220** | **-0.012** |  |  |  |
| **Design F** | **-0.109** | **0.050** | **0.031** | **-0.208** | **-0.010** |  |  |  |
|  | **Fifth best model** | | | | | | | |
|  | **est** | **se** | **p** | **CI.lb** | **CI.ub** | **Var_sp_** | **Var_phy_** | **Var_es_** |
| Intercept | 0.195 | 0.129 | 0.134 | -0.061 | 0.451 | 0.005 | 0.017 | 0.060 |
| Design B | -0.079 | 0.074 | 0.292 | -0.225 | 0.068 |  |  |  |
| Design C | 0.039 | 0.042 | 0.354 | -0.044 | 0.121 |  |  |  |
| **Design D** | **-0.316** | **0.053** | **<0.0001** | **-0.420** | **-0.213** |  |  |  |
| Design E | -0.098 | 0.054 | 0.071 | -0.204 | 0.009 |  |  |  |
| **Design F** | -0.110 | 0.051 | 0.031 | -0.209 | -0.010 |  |  |  |
| LT50 | -0.043 | 0.028 | 0.121 | -0.097 | 0.011 |  |  |  |
| A. inverts | 0.041 | 0.149 | 0.784 | -0.255 | 0.337 |  |  |  |
| Fishes | 0.069 | 0.122 | 0.575 | -0.173 | 0.311 |  |  |  |
| Reptiles | 0.020 | 0.098 | 0.842 | -0.174 | 0.214 |  |  |  |
| T. inverts | -0.084 | 0.159 | 0.600 | -0.399 | 0.231 |  |  |  |

**TABLE S27:** **Results of the univariate meta-regression fitted with standard error (Egger regression) or sampling variance.** Standard error was calculated as the squared root of sampling variance. se: standard error; CI.lb: lower bound of the 95% confidence interval; CI.ub: upper bound of the 95% confidence interval; k_sp_: number of species included in the model; k_es_: number of effect sizes included in the model; Var_sp_: variance explained by variation between species; Var_phy_: variance explained by shared evolutionary history; Var_es_: variance associated with the residuals

|  | **est** | **se** | **p** | **CI.lb** | **CI.ub** | **k_sp_** | **k_es_** | **Var_sp_** | **Var_phy_** | **Var_es_** |
| --- | --- | --- | --- | --- | --- | --- | --- | --- | --- | --- |
| Intercept | 0.193 | 0.089 | 0.031 | 0.018 | 0.369 | 138 | 1089 | 0.010 | 0.025 | 0.061 |
| $\sqrt{Sampling variance}$ | -0.040 | 0.136 | 0.770 | -0.306 | 0.226 |  |  |  |  |  |
| Intercept | 0.190 | 0.088 | 0.032 | 0.017 | 0.364 | 138 | 1089 | 0.010 | 0.025 | 0.061 |
| Sampling variance | -0.034 | 0.086 | 0.693 | -0.203 | 0.135 |  |  |  |  |  |

**TABLE S28:** **Results of the univariate meta-regression fitted with peer review history.** Contrasts are provided in the bottom rows. se: standard error; CI.lb: lower bound of the 95% confidence interval; CI.ub: upper bound of the 95% confidence interval; k_sp_: number of species included in the model; k_es_: number of effect sizes included in the model; Var_sp_: variance explained by variation between species; Var_phy_: variance explained by shared evolutionary history; Var_es_: variance associated with the residuals

|  | **est** | **se** | **p** | **CI.lb** | **CI.ub** | **k_sp_** | **k_es_** | **Var_sp_** | **Var_phy_** | **Var_es_** |
| --- | --- | --- | --- | --- | --- | --- | --- | --- | --- | --- |
| Dissertation | 0.190 | 0.088 | 0.032 | 0.017 | 0.364 | 138 | 1089 | 0.010 | 0.025 | 0.061 |
| Published article | -0.034 | 0.086 | 0.693 | -0.203 | 0.135 |  |  |  |  |  |
| Int. (Dissertation) | 0.133 | 0.094 | 0.158 | -0.052 | 0.319 | 138 | 1089 | 0.010 | 0.025 | 0.061 |
| Published article | 0.063 | 0.042 | 0.138 | -0.020 | 0.146 |  |  |  |  |  |

**TABLE S29:** **Results of the univariate meta-regression assessing a time lag bias.** Publication year was centred to zero mean. se: standard error; CI.lb: lower bound of the 95% confidence interval; CI.ub: upper bound of the 95% confidence interval; k_sp_: number of species included in the model; k_es_: number of effect sizes included in the model; Var_sp_: variance explained by variation between species; Var_phy_: variance explained by shared evolutionary history; Var_es_: variance associated with the residuals

|  | **est** | **se** | **p** | **CI.lb** | **CI.ub** | **k_sp_** | **k_es_** | **Var_sp_** | **Var_phy_** | **Var_es_** |
| --- | --- | --- | --- | --- | --- | --- | --- | --- | --- | --- |
| Intercept | 0.191 | 0.089 | 0.032 | 0.016 | 0.366 | 138 | 1089 | 0.010 | 0.025 | 0.061 |
| Publication year | -0.001 | 0.001 | 0.746 | -0.003 | 0.002 |  |  |  |  |  |

**TABLE S30:** **Results of leave-one-sensitivity analyses**. Mean meta-analytic estimates are presented after the iterative removal of one study or one species. est: estimate; se: standard error; CI.lb: lower bound of the 95% confidence interval; CI.ub: upper bound of the 95% confidence interval. Removing one study at a time produced estimated ranging from 0.180 and 0.199; whereas removing one species produced estimates ranging from 0.180 and 0.198.

|  | **est** | **se** | **p** | **CI.lb** | **CI.ub** |
| --- | --- | --- | --- | --- | --- |
| Leave one study out | 0.190 | 0.088 | 0.033 | 0.016 | 0.364 |
| Leave one species out | 0.190 | 0.088 | 0.033 | 0.016 | 0.364 |

**TABLE S31:** **Results of univariate meta-regressions only considering initial responses to developmental acclimation.** Coefficients statistically different from zero were highlighted in bold. est: estimate; se: standard error; CI.lb: lower bound of the 95% confidence interval; CI.ub: upper bound of the 95% confidence interval; PI.lb: lower bound of the 95% prediction interval; PI.ub: upper bound of the 95% prediction interval; k_sp_: number of species included in the model; k_es_: number of effect sizes included in the model; Var_sp_: variance explained by variation between species; Var_phy_: variance explained by shared evolutionary history; Var_es_: variance associated with the residuals; A. inverts: aquatic invertebrates; T. inverts: terrestrial invertebrates; CTmax: critical thermal maximum; LT50: median lethal temperature. Designs are outlined in Fig. 2.

|  | **est** | **se** | **p** | **CI.lb** | **CI.ub** | **PI.lb** | **PI.ub** | **k_sp_** | **k_es_** | **Var_sp_** | **Var_phy_** | **Var_es_** |
| --- | --- | --- | --- | --- | --- | --- | --- | --- | --- | --- | --- | --- |
| **Aquatic** | **0.233** | **0.051** | **<0.0001** | **0.132** | **0.335** | -0.344 | 0.811 | 110 | 781 | 0.006 | 0.007 | 0.069 |
| Terrestrial | 0.073 | 0.077 | 0.345 | -0.079 | 0.225 | -0.516 | 0.661 | 11 | 85 |  |  |  |
|  | **est** | **se** | **p** | **CI.lb** | **CI.ub** | **PI.lb** | **PI.ub** | **k_sp_** | **k_es_** | **Var_sp_** | **Var_phy_** | **Var_es_** |
| Amphibians | 0.225 | 0.122 | 0.067 | -0.016 | 0.465 | -0.411 | 0.860 | 13 | 65 | 0.005 | 0.014 | 0.069 |
| **A. inverts** | **0.195** | **0.093** | **0.038** | **0.011** | **0.379** | -0.422 | 0.811 | 30 | 208 |  |  |  |
| **Fishes** | **0.284** | **0.092** | **0.002** | **0.011** | **0.379** | -0.331 | 0.900 | 63 | 496 |  |  |  |
| Reptiles | 0.158 | 0.146 | 0.282 | -0.132 | 0.448 | -0.498 | 0.814 | 4 | 12 |  |  |  |
| T. inverts | 0.031 | 0.109 | 0.779 | -0.185 | 0.246 | -0.596 | 0.657 | 11 | 85 |  |  |  |
|  | **est** | **se** | **p** | **CI.lb** | **CI.ub** | **PI.lb** | **PI.ub** | **k_sp_** | **k_es_** | **Var_sp_** | **Var_phy_** | **Var_es_** |
| **CTmax** | **0.227** | **0.072** | **0.002** | **0.085** | **0.369** | -0.376 | 0.822 | 101 | 647 | 0.006 | 0.016 | 0.069 |
| **LT50** | **0.175** | **0.075** | **0.020** | **0.028** | **0.323** | -0.413 | 0.786 | 33 | 219 |  |  |  |
|  | **est** | **se** | **p** | **CI.lb** | **CI.ub** |  |  | **k_sp_** | **k_es_** | **Var_sp_** | **Var_phy_** | **Var_es_** |
| Intercept | 0.234 | 0.099 | 0.020 | 0.037 | 0.430 |  |  | 99 | 653 | 0.001 | 0.031 | 0.075 |
| Heating rate | 0.014 | 0.033 | 0.675 | -0.051 | 0.078 |  |  |  |  |  |  |  |

**TABLE S32:** **Results of univariate meta-regressions only considering initial responses to developmental acclimation and modelling residual heteroscedasticity.** Coefficients statistically different from zero were highlighted in bold. est: estimate; se: standard error; CI.lb: lower bound of the 95% confidence interval; CI.ub: upper bound of the 95% confidence interval; PI.lb: lower bound of the 95% prediction interval; PI.ub: upper bound of the 95% prediction interval; k_sp_: number of species included in the model; k_es_: number of effect sizes included in the model; Var_sp_: variance explained by variation between species; Var_phy_: variance explained by shared evolutionary history; Var_es_: variance associated with the residuals; A. inverts: aquatic invertebrates; T. inverts: terrestrial invertebrates; CTmax: critical thermal maximum; LT50: median lethal temperature. Designs are outlined in Fig. 2.

|  | **est** | **se** | **p** | **CI.lb** | **CI.ub** | **PI.lb** | **PI.ub** | **k_sp_** | **k_es_** | **Var_sp_** | **Var_phy_** | **Var_es_** |
| --- | --- | --- | --- | --- | --- | --- | --- | --- | --- | --- | --- | --- |
| **Aquatic** | **0.234** | **0.048** | **<0.0001** | **0.139** | **0.329** | -0.392 | 0.854 | 110 | 781 | 0.006 | 0.006 | 0.078 |
| Terrestrial | 0.074 | 0.065 | 0.255 | -0.054 | 0.202 | -0.320 | 0.464 | 11 | 85 |  |  | 0.009 |
|  | **est** | **se** | **p** | **CI.lb** | **CI.ub** | **PI.lb** | **PI.ub** | **k_sp_** | **k_es_** | **Var_sp_** | **Var_phy_** | **Var_es_** |
| Amphibians | 0.217 | 0.153 | 0.161 | -0.087 | 0.520 | -0.768 | 1.201 | 13 | 65 | 0.005 | 0.021 | 0.198 |
| A. inverts | 0.202 | 0.115 | 0.082 | -0.026 | 0.431 | -0.651 | 1.056 | 30 | 208 |  |  | 0.146 |
| **Fishes** | **0.296** | **0.108** | **0.007** | **0.081** | **0.511** | -0.227 | 0.819 | 63 | 496 |  |  | 0.032 |
| Reptiles | 0.143 | 0.150 | 0.340 | -0.153 | 0.439 | -0.313 | 0.599 | 4 | 12 |  |  | 0.005 |
| T. inverts | 0.031 | 0.125 | 0.806 | -0.217 | 0.279 | -0.413 | 0.475 | 11 | 85 |  |  | 0.009 |
|  | **est** | **se** | **p** | **CI.lb** | **CI.ub** | **PI.lb** | **PI.ub** | **k_sp_** | **k_es_** | **Var_sp_** | **Var_phy_** | **Var_es_** |
| **CTmax** | **0.225** | **0.072** | **0.002** | **0.084** | **0.366** | -0.406 | 0.856 | 101 | 647 | 0.006 | 0.016 | 0.076 |
| **LT50** | **0.172** | **0.074** | **0.021** | **0.026** | **0.317** | -0.343 | 0.687 | 33 | 219 |  |  | 0.041 |

**TABLE S33:** **Results of univariate meta-regressions only considering persistent responses to developmental acclimation.** Coefficients statistically different from zero were highlighted in bold. est: estimate; se: standard error; CI.lb: lower bound of the 95% confidence interval; CI.ub: upper bound of the 95% confidence interval; PI.lb: lower bound of the 95% prediction interval; PI.ub: upper bound of the 95% prediction interval; k_sp_: number of species included in the model; k_es_: number of effect sizes included in the model; Var_sp_: variance explained by variation between species; Var_phy_: variance explained by shared evolutionary history; Var_es_: variance associated with the residuals; A. inverts: aquatic invertebrates; T. inverts: terrestrial invertebrates; CTmax: critical thermal maximum; LT50: median lethal temperature. Designs are outlined in Fig. 2.

|  | **est** | **se** | **p** | **CI.lb** | **CI.ub** | **PI.lb** | **PI.ub** | **k_sp_** | **k_es_** | **Var_sp_** | **Var_phy_** | **Var_es_** |
| --- | --- | --- | --- | --- | --- | --- | --- | --- | --- | --- | --- | --- |
| Aquatic | 0.053 | 0.105 | 0.617 | -0.165 | 0.272 | -0.454 | 0.567 | 14 | 148 | 0.000 | 0.031 | 0.021 |
| Terrestrial | 0.052 | 0.114 | 0.654 | -0.185 | 0.289 | -0.450 | 0.588 | 9 | 75 |  |  |  |
|  | **est** | **se** | **p** | **CI.lb** | **CI.ub** | **PI.lb** | **PI.ub** | **k_sp_** | **k_es_** | **Var_sp_** | **Var_phy_** | **Var_es_** |
| Amphibians | 0.009 | 0.212 | 0.966 | -0.436 | 0.454 | -0.677 | 0.695 | 1 | 6 | 0.000 | 0.040 | 0.021 |
| A. inverts | 0.152 | 0.152 | 0.329 | -0.167 | 0.471 | -0.459 | 0.764 | 2 | 13 |  |  |  |
| Fishes | -0.028 | 0.161 | 0.864 | -0.366 | 0.310 | -0.649 | 0.594 | 10 | 127 |  |  |  |
| Reptiles | -0.051 | 0.200 | 0.801 | -0.471 | 0.369 | -0.721 | 0.619 | 3 | 15 |  |  |  |
| T. inverts | 0.119 | 0.165 | 0.479 | -0.227 | 0.465 | -0.507 | 0.745 | 7 | 62 |  |  |  |
|  | **est** | **se** | **p** | **CI.lb** | **CI.ub** | **PI.lb** | **PI.ub** | **k_sp_** | **k_es_** | **Var_sp_** | **Var_phy_** | **Var_es_** |
| CTmax | 0.044 | 0.096 | 0.646 | -0.145 | 0.233 | -0.429 | 0.521 | 22 | 216 | 0.000 | 0.027 | 0.022 |
| LT50 | 0.148 | 0.140 | 0.289 | -0.127 | 0.423 | -0.283 | 0.724 | 2 | 7 |  |  |  |
|  | **est** | **se** | **p** | **CI.lb** | **CI.ub** |  |  | **k_sp_** | **k_es_** | **Var_sp_** | **Var_phy_** | **Var_es_** |
| Intercept | 0.039 | 0.094 | 0.681 | -0.158 | 0.236 |  |  | 20 | 202 | 0.002 | 0.024 | 0.022 |
| Heating rate | 0.153 | 0.082 | 0.064 | -0.009 | 0.315 |  |  |  |  |  |  |  |

**TABLE S34:** **Results of univariate meta-regressions only considering persistent responses to developmental acclimation and modelling residual heteroscedasticity.** Coefficients statistically different from zero were highlighted in bold. est: estimate; se: standard error; CI.lb: lower bound of the 95% confidence interval; CI.ub: upper bound of the 95% confidence interval; PI.lb: lower bound of the 95% prediction interval; PI.ub: upper bound of the 95% prediction interval; k_sp_: number of species included in the model; k_es_: number of effect sizes included in the model; Var_sp_: variance explained by variation between species; Var_phy_: variance explained by shared evolutionary history; Var_es_: variance associated with the residuals; A. inverts: aquatic invertebrates; T. inverts: terrestrial invertebrates; CTmax: critical thermal maximum; LT50: median lethal temperature. Designs are outlined in Fig. 2.

|  | **est** | **se** | **p** | **CI.lb** | **CI.ub** | **PI.lb** | **PI.ub** | **k_sp_** | **k_es_** | **Var_sp_** | **Var_phy_** | **Var_es_** |
| --- | --- | --- | --- | --- | --- | --- | --- | --- | --- | --- | --- | --- |
| Aquatic | 0.051 | 0.100 | 0.617 | -0.157 | 0.258 | -0.493 | 0.594 | 14 | 148 | 0.003 | 0.027 |  |
| Terrestrial | 0.049 | 0.107 | 0.649 | -0.173 | 0.271 | -0.401 | 0.499 | 9 | 75 |  |  |  |
|  | **est** | **se** | **p** | **CI.lb** | **CI.ub** | **PI.lb** | **PI.ub** | **k_sp_** | **k_es_** | **Var_sp_** | **Var_phy_** | **Var_es_** |
| Amphibians | 0.005 | 0.214 | 0.982 | -0.444 | 0.454 | -0.786 | 0.796 | 1 | 6 | 0.001 | 0.033 | 0.063 |
| A. inverts | 0.136 | 0.135 | 0.328 | -0.148 | 0.419 | -0.378 | 0.650 | 2 | 13 |  |  | 0.008 |
| Fishes | -0.029 | 0.147 | 0.844 | -0.338 | 0.279 | -0.654 | 0.592 | 10 | 127 |  |  | 0.033 |
| Reptiles | -0.047 | 0.185 | 0.801 | -0.436 | 0.341 | -0.707 | 0.613 | 3 | 15 |  |  | 0.031 |
| T. inverts | 0.097 | 0.146 | 0.516 | -0.210 | 0.404 | -0.416 | 0.609 | 7 | 62 |  |  | 0.005 |
|  | **est** | **se** | **p** | **CI.lb** | **CI.ub** | **PI.lb** | **PI.ub** | **k_sp_** | **k_es_** | **Var_sp_** | **Var_phy_** | **Var_es_** |
| CTmax | 0.049 | 0.098 | 0.621 | -0.145 | 0.242 | -0.430 | 0.528 | 22 | 216 | 0.000 | 0.028 | 0.021 |
| LT50 | 0.188 | 0.166 | 0.259 | -0.139 | 0.514 | -0.460 | 0.835 | 2 | 7 |  |  | 0.052 |

**TABLE S35:** **Risk of bias analyses.** Provided are the meta-analytic estimates after the removal of imputed values, concerning data, or extreme effect sizes. est: estimate; se: standard error; CI.lb: lower bound of the 95% confidence interval; CI.ub: upper bound of the 95% confidence interval; k_sp_: number of species included in the model; k_es_: number of effect sizes included in the model; Var_sp_: variance explained by variation between species; Var_phy_: variance explained by shared evolutionary history; Var_es_: variance associated with the residuals; T_KO_: temperature tolerated for one hour.

|  | **est** | **se** | **p** | **CI.lb** | **CI.ub** | **PI.lb** | **PI.ub** | **k_sp_** | **k_es_** | **Var_sp_** | **Var_phy_** | **Var_es_** |
| --- | --- | --- | --- | --- | --- | --- | --- | --- | --- | --- | --- | --- |
| Without  imputed values | 0.187 | 0.083 | 0.027 | 0.022 | 0.352 | -0.372 | 0.743 | 133 | 1000 | 0.011 | 0.022 | 0.042 |
| Without  unusual data | 0.190 | 0.087 | 0.030 | 0.019 | 0.361 | -0.377 | 0.760 | 126 | 852 | 0.011 | 0.024 | 0.041 |
| Without T_KO_ | 0.194 | 0.091 | 0.036 | 0.013 | 0.374 | -0.448 | 0.835 | 136 | 1069 | 0.009 | 0.027 | 0.061 |
| -1.25<  dARR  <1.25 | 0.195 | 0.113 | 0.086 | -0.028 | 0.419 | -0.423 | 0.813 | 138 | 1079 | 0.010 | 0.042 | 0.033 |
| -0.5 < dARR | 0.218 | 0.198 | 0.273 | -0.174 | 0.610 | -0.695 | 1.131 | 137 | 1055 | 0.009 | 0.134 | 0.032 |
| -0.15 < dARR | 0.236 | 0.204 | 0.249 | -0.168 | 0.640 | -0.689 | 1.162 | 136 | 992 | 0.009 | 0.143 | 0.026 |
| 0 ≤ dARR | 0.226 | 0.117 | 0.057 | -0.006 | 0.458 | -0.383 | 0.834 | 138 | 138 | 0.008 | 0.046 | 0.027 |

**TABLE S36:** **Results of univariate meta-regressions testing for the association between developmental plasticity levels and body mass.** Note that these analyses were post-hoc (suggested at the peer-review stage). Body mass was right-skewed and was therefore ln-transformed. est: estimate; se: standard error; CI.lb: lower bound of the 95% confidence interval; CI.ub: upper bound of the 95% confidence interval; k_sp_: number of species included in the model; k_es_: number of effect sizes included in the model; Var_sp_: variance explained by variation between species; Var_phy_: variance explained by shared evolutionary history; Var_es_: variance associated with the residuals.

|  |  |  |  |  |  |  |  |  |  |  |
| --- | --- | --- | --- | --- | --- | --- | --- | --- | --- | --- |
|  | **All effect sizes** | | | | | | | | | |
|  | **est** | **se** | **p** | **CI.lb** | **CI.ub** | **k_sp_** | **k_es_** | **Var_sp_** | **Var_phy_** | **Var_es_** |
| Intercept | 0.214 | 0.030 | <0.0001 | 0.154 | 0.275 | 67 | 494 | 0.009 | 0.001 | 0.056 |
| Body mass | 0.012 | 0.006 | 0.053 | -0.001 | 0.025 |  |  |  |  |  |
|  | **Initial responses only** | | | | | | | | | |
|  | **est** | **se** | **p** | **CI.lb** | **CI.ub** | **k_sp_** | **k_es_** | **Var_sp_** | **Var_phy_** | **Var_es_** |
| Intercept | 0.258 | 0.035 | <0.0001 | 0.188 | 0.327 | 59 | 411 | 0.004 | 0.002 | 0.060 |
| Body mass | 0.004 | 0.006 | 0.493 | -0.008 | 0.017 |  |  |  |  |  |
|  | **Persistent responses only** | | | | | | | | | |
|  | **est** | **se** | **p** | **CI.lb** | **CI.ub** | **k_sp_** | **k_es_** | **Var_sp_** | **Var_phy_** | **Var_es_** |
| Intercept | 0.037 | 0.055 | 0.510 | -0.086 | 0.161 | 11 | 83 | 0.000 | 0.005 | 0.019 |
| Body mass | 0.019 | 0.013 | 0.132 | -0.006 | 0.044 |  |  |  |  |  |

**TABLE S37:** **Results of univariate meta-regressions testing for the association between developmental plasticity levels and the age at sexual maturity.** Note that these analyses were post-hoc (suggested at the peer-review stage). Age at sexual maturity was right-skewed and was therefore ln-transformed. est: estimate; se: standard error; CI.lb: lower bound of the 95% confidence interval; CI.ub: upper bound of the 95% confidence interval; k_sp_: number of species included in the model; k_es_: number of effect sizes included in the model; Var_sp_: variance explained by variation between species; Var_phy_: variance explained by shared evolutionary history; Var_es_: variance associated with the residuals

|  |  |  |  |  |  |  |  |  |  |  |
| --- | --- | --- | --- | --- | --- | --- | --- | --- | --- | --- |
|  | **All effect sizes** | | | | | | | | | |
|  | **est** | **se** | **p** | **CI.lb** | **CI.ub** | **k_sp_** | **k_es_** | **Var_sp_** | **Var_phy_** | **Var_es_** |
| Intercept | 0.194 | 0.070 | 0.006 | 0.056 | 0.332 | 114 | 971 | 0.013 | 0.015 | 0.039 |
| Age maturity | 0.011 | 0.014 | 0.425 | -0.016 | 0.039 |  |  |  |  |  |
|  | **Initial responses only** | | | | | | | | | |
|  | **est** | **se** | **p** | **CI.lb** | **CI.ub** | **k_sp_** | **k_es_** | **Var_sp_** | **Var_phy_** | **Var_es_** |
| Intercept | 0.222 | 0.046 | <0.0001 | 0.131 | 0.313 | 98 | 752 | 0.009 | 0.006 | 0.043 |
| Age maturity | 0.003 | 0.013 | 0.805 | -0.023 | 0.029 |  |  |  |  |  |
|  | **Persistent responses only** | | | | | | | | | |
|  | **est** | **se** | **p** | **CI.lb** | **CI.ub** | **k_sp_** | **k_es_** | **Var_sp_** | **Var_phy_** | **Var_es_** |
| Intercept | 0.033 | 0.090 | 0.713 | -0.154 | 0.220 | 22 | 219 | 0.001 | 0.023 | 0.022 |
| Age maturity | -0.020 | 0.019 | 0.313 | -0.060 | 0.020 |  |  |  |  |  |

**TABLE S38:** **Results of meta-regressions testing for two-way interactive effects of latitude, body mass, and methodology on measured developmental plasticity.** Note that these analyses were post-hoc (suggested at the peer-review stage). Body mass, heating rate, and acclimation duration were right-skewed and were therefore ln-transformed. Latitude was taken as absolute values. All predictors were standardised. est: estimate; se: standard error; CI.lb: lower bound of the 95% confidence interval; CI.ub: upper bound of the 95% confidence interval; k_sp_: number of species included in the model; k_es_: number of effect sizes included in the model; Var_sp_: variance explained by variation between species; Var_phy_: variance explained by shared evolutionary history; Var_es_: variance associated with the residuals; x: interaction.

|  | **est** | **se** | **p** | **CI.lb** | **CI.ub** | **k_sp_** | **k_es_** | | **Var_sp_** | | **Var_phy_** | | **Var_es_** | |
| --- | --- | --- | --- | --- | --- | --- | --- | --- | --- | --- | --- | --- | --- | --- |
| Intercept | 0.231 | 0.147 | 0.137 | -0.082 | 0.544 | 19 | 80 | | 0.009 | | 0.039 | | 0.034 | |
| Body mass | 0.046 | 0.060 | 0.448 | -0.074 | 0.166 |  |  |  |  |  |  |  |  |  |
| Latitude | -0.004 | 0.028 | 0.901 | -0.059 | 0.052 |  |  |  |  |  |  |  |  |  |
| Body mass x  Latitude | 0.036 | 0.037 | 0.341 | -0.039 | 0.110 |  |  |  |  |  |  |  |  |  |
|  | **est** | **se** | **p** | **CI.lb** | **CI.ub** | **k_sp_** | **k_es_** | | **Var_sp_** | | **Var_phy_** | | **Var_es_** | |
| **Intercept** | **0.142** | **0.039** | **0.001** | **0.062** | **0.062** | 38 | 243 | | 0.016 | | 0.001 | | 0.107 | |
| Latitude | -0.005 | 0.031 | 0.865 | -0.066 | 0.056 |  |  |  |  |  |  |  |  |  |
| Heating rate | 0.020 | 0.043 | 0.644 | -0.064 | 0.104 |  |  |  |  |  |  |  |  |  |
| Latitude x  Heating rate | -0.003 | 0.033 | 0.925 | -0.068 | 0.062 |  |  |  |  |  |  |  |  |  |
|  | **est** | **se** | **p** | **CI.lb** | **CI.ub** | **k_sp_** | **k_es_** | | **Var_sp_** | | **Var_phy_** | | **Var_es_** | |
| **Intercept** | **0.220** | **0.031** | **<0.001** | **0.158** | **0.282** | 65 | 399 | | 0.008 | | 0.001 | | 0.062 | |
| **Body mass** | **0.039** | **0.019** | **0.037** | **0.002** | **0.075** |  |  |  |  |  |  |  |  |  |
| Heating rate | 0.033 | 0.020 | 0.094 | -0.006 | 0.072 |  |  |  |  |  |  |  |  |  |
| Body mass x  Heating rate | 0.021 | 0.017 | 0.219 | -0.012 | 0.054 |  |  |  |  |  |  |  |  |  |
|  | **est** | **se** | **p** | **CI.lb** | **CI.ub** | **k_sp_** | **k_es_** | | **Var_sp_** | | **Var_phy_** | | **Var_es_** | |
| **Intercept** | **0.193** | **0.035** | **<0.001** | **0.122** | **0.264** | 33 | 156 | | 0.000 | | 0.000 | | 0.160 | |
| Latitude | 0.005 | 0.029 | 0.877 | -0.053 | 0.062 |  |  |  |  |  |  |  |  |  |
| Acclimation time | 0.039 | 0.031 | 0.219 | -0.023 | 0.100 |  |  |  |  |  |  |  |  |  |
| Latitude x Acclimation time | 0.012 | 0.024 | 0.615 | -0.035 | 0.059 |  |  |  |  |  |  |  |  |  |
|  | **est** | **se** | **p** | **CI.lb** | **CI.ub** | **k_sp_** | **k_es_** | | **Var_sp_** | | **Var_phy_** | | **Var_es_** | |
| **Intercept** | **0.207** | **0.028** | **<0.001** | **0.151** | **0.262** | 61 | | 456 | | 0.007 | | 0.001 | | 0.060 |
| Body mass | 0.017 | 0.019 | 0.368 | -0.020 | 0.054 |  |  |  |  |  |  |  |  |  |
| Acclimation time | 0.039 | 0.024 | 0.105 | -0.008 | 0.085 |  |  |  |  |  |  |  |  |  |
| Body mass x Acclimation time | -0.005 | 0.024 | 0.847 | -0.052 | 0.043 |  |  |  |  |  |  |  |  |  |
|  | **est** | **se** | **p** | **CI.lb** | **CI.ub** | **k_sp_** | **k_es_** | | **Var_sp_** | | **Var_phy_** | | **Var_es_** | |
| **Intercept** | **0.204** | **0.031** | **<0.001** | **0.143** | **0.265** | 99 | | 614 | | 0.006 | | 0.002 | | 0.087 |
| Heating rate | 0.029 | 0.016 | 0.073 | -0.003 | 0.060 |  |  |  |  |  |  |  |  |  |
| **Acclimation time** | **0.056** | **0.017** | **0.001** | **0.022** | **0.090** |  |  |  |  |  |  |  |  |  |
| Heating rate x Acclimation time | 0.026 | 0.014 | 0.062 | -0.001 | 0.054 |  |  |  |  |  |  |  |  |  |

**TABLE S39:** **Results of meta-regressions testing for three-way interactive effects of latitude, body mass, and methodology on measured developmental plasticity.** Note that these analyses were post-hoc (suggested at the peer-review stage). Body mass, heating rate, and acclimation time were right-skewed and were therefore ln-transformed. Latitude was taken as absolute values. All predictors were standardised. est: estimate; se: standard error; CI.lb: lower bound of the 95% confidence interval; CI.ub: upper bound of the 95% confidence interval; k_sp_: number of species included in the model; k_es_: number of effect sizes included in the model; Var_sp_: variance explained by variation between species; Var_phy_: variance explained by shared evolutionary history; Var_es_: variance associated with the residuals; x: interaction.

|  | **est** | **se** | **p** | **CI.lb** | **CI.ub** | **k_sp_** | **k_es_** | **Var_sp_** | **Var_phy_** | **Var_es_** |
| --- | --- | --- | --- | --- | --- | --- | --- | --- | --- | --- |
| Intercept | 0.257 | 0.201 | 0.230 | -0.191 | 0.704 | 18 | 76 | 0.009 | 0.077 | 0.032 |
| Body mass | 0.060 | 0.0851 | 0.398 | -0.081 | 0.201 |  |  |  |  |  |
| Heating rate | 0.048 | 0.077 | 0.533 | -0.105 | 0.202 |  |  |  |  |  |
| Latitude | 0.014 | 0.044 | 0.747 | -0.074 | 0.102 |  |  |  |  |  |
| Body mass x  Heating rate | -0.172 | 0.142 | 0.228 | -0.455 | 0.110 |  |  |  |  |  |
| Body mass x  Latitude | 0.035 | 0.052 | 0.506 | -0.069 | 0.139 |  |  |  |  |  |
| Heating rate x  Latitude | 0.076 | 0.062 | 0.224 | -0.048 | 0.200 |  |  |  |  |  |
| Body mass x  Heating rate x  Latitude | -0.020 | 0.098 | 0.841 | -0.216 | 0.176 |  |  |  |  |  |
|  | **est** | **se** | **p** | **CI.lb** | **CI.ub** | **k_sp_** | **k_es_** | **Var_sp_** | **Var_phy_** | **Var_es_** |
| Intercept | **0.200** | **0.025** | **<0.001** | **0.149** | **0.250** | 60 | 365 | 0.007 | 0.000 | 0.067 |
| Body mass | 0.021 | 0.020 | 0.285 | -0.018 | 0.061 |  |  |  |  |  |
| Heating rate | 0.035 | 0.022 | 0.114 | -0.009 | 0.079 |  |  |  |  |  |
| Acclimation time | 0.045 | 0.026 | 0.091 | -0.007 | 0.097 |  |  |  |  |  |
| Body mass x Heating rate | 0.015 | 0.018 | 0.392 | -0.020 | 0.051 |  |  |  |  |  |
| Body mass x Acclimation time | -0.018 | 0.027 | 0.499 | -0.071 | 0.035 |  |  |  |  |  |
| Heating rate x Acclimation time | 0.027 | 0.019 | 0.156 | -0.011 | 0.065 |  |  |  |  |  |
| Body mass x  Heating rate x  Acclimation time | -0.047 | 0.024 | 0.050 | -0.095 | 0.000 |  |  |  |  |  |
|  | **est** | **se** | **p** | **CI.lb** | **CI.ub** | **k_sp_** | **k_es_** | **Var_sp_** | **Var_phy_** | **Var_es_** |
| **Intercept** | **0.167** | **0.056** | **0.006** | **0.052** | **0.283** | 32 | 140 | 0.000 | 0.000 | 0.185 |
| Acclimation time | 0.093 | 0.060 | 0.124 | -0.026 | 0.212 |  |  |  |  |  |
| Heating rate | -0.012 | 0.064 | 0.857 | -0.138 | 0.115 |  |  |  |  |  |
| Latitude | 0.022 | 0.039 | 0.574 | -0.055 | 0.099 |  |  |  |  |  |
| Acclimation time x Heating rate | -0.089 | 0.065 | 0.176 | -0.218 | 0.040 |  |  |  |  |  |
| Acclimation time x  Latitude | -0.034 | 0.044 | 0.441 | -0.121 | 0.053 |  |  |  |  |  |
| Heating rate x Latitude | -0.045 | 0.053 | 0.394 | -0.150 | 0.059 |  |  |  |  |  |
| Acclimation time x Heating rate x Latitude | 0.014 | 0.046 | 0.766 | -0.077 | 0.105 |  |  |  |  |  |
|  | **est** | **se** | **p** | **CI.lb** | **CI.ub** | **k_sp_** | **k_es_** | **Var_sp_** | **Var_phy_** | **Var_es_** |
| Intercept | 0.249 | 0.167 | 0.181 | -0.147 | 0.644 | 15 | 56 | 0.000 | 0.028 | 0.047 |
| Body mass | -0.042 | 0.154 | 0.784 | -0.352 | 0.267 |  |  |  |  |  |
| Acclimation time | -0.006 | 0.084 | 0.946 | -0.175 | 0.163 |  |  |  |  |  |
| Latitude | -0.018 | 0.063 | 0.781 | -0.145 | 0.108 |  |  |  |  |  |
| Body mass x Acclimation time | 0.013 | 0.116 | 0.912 | -0.221 | 0.247 |  |  |  |  |  |
| Body mass x Latitude | -0.037 | 0.084 | 0.660 | -0.205 | 0.131 |  |  |  |  |  |
| Acclimation time x Latitude | 0.043 | 0.057 | 0.456 | -0.071 | 0.156 |  |  |  |  |  |
| Body mass x  Acclimation time x Latitude | 0.039 | 0.073 | 0.589 | -0.106 | 0.185 |  |  |  |  |  |

**TABLE S40:** **Results of meta-regressions testing for two-way interactive effects of latitude, body mass, and methodology on measured initial developmental plasticity.** Note that these analyses were post-hoc (suggested at the peer-review stage). Body mass, heating rate, and acclimation time were right-skewed and were therefore ln-transformed. Latitude was taken as absolute values. All predictors were standardised. est: estimate; se: standard error; CI.lb: lower bound of the 95% confidence interval; CI.ub: upper bound of the 95% confidence interval; k_sp_: number of species included in the model; k_es_: number of effect sizes included in the model; Var_sp_: variance explained by variation between species; Var_phy_: variance explained by shared evolutionary history; Var_es_: variance associated with the residuals; x: interaction.

|  | **est** | **se** | **p** | **CI.lb** | **CI.ub** | **k_sp_** | **k_es_** | | **Var_sp_** | | **Var_phy_** | | **Var_es_** | |
| --- | --- | --- | --- | --- | --- | --- | --- | --- | --- | --- | --- | --- | --- | --- |
| Intercept | **0.276** | **0.076** | **0.004** | **0.108** | **0.443** | 15 | 59 | | 0.001 | | 0.008 | | 0.048 | |
| Body mass | -0.023 | 0.047 | 0.623 | -0.118 | 0.071 |  |  |  |  |  |  |  |  |  |
| Latitude | 0.005 | 0.030 | 0.865 | -0.054 | 0.064 |  |  |  |  |  |  |  |  |  |
| Body mass x  Latitude | -0.001 | 0.037 | 0.979 | -0.076 | 0.074 |  |  |  |  |  |  |  |  |  |
|  | **est** | **se** | **p** | **CI.lb** | **CI.ub** | **k_sp_** | **k_es_** | | **Var_sp_** | | **Var_phy_** | | **Var_es_** | |
| **Intercept** | **0.216** | **0.05** | **0.001** | **0.111** | **0.321** | 31 | 139 | | 0.001 | | 0.002 | | 0.159 | |
| Latitude | -0.002 | 0.037 | 0.958 | -0.076 | 0.072 |  |  |  |  |  |  |  |  |  |
| Heating rate | -0.020 | 0.052 | 0.708 | -0.122 | 0.083 |  |  |  |  |  |  |  |  |  |
| Latitude x  Heating rate | 0.003 | 0.042 | 0.940 | -0.080 | 0.087 |  |  |  |  |  |  |  |  |  |
|  | **est** | **se** | **p** | **CI.lb** | **CI.ub** | **k_sp_** | **k_es_** | | **Var_sp_** | | **Var_phy_** | | **Var_es_** | |
| **Intercept** | **0.270** | **0.035** | **<0.001** | **0.199** | **0.340** | 58 | 321 | | 0.001 | | 0.002 | | 0.069 | |
| Body mass | 0.010 | 0.018 | 0.582 | -0.025 | 0.045 |  |  |  |  |  |  |  |  |  |
| Heating rate | 0.039 | 0.020 | 0.050 | -0.001 | 0.078 |  |  |  |  |  |  |  |  |  |
| Body mass x  Heating rate | 0.007 | 0.017 | 0.685 | -0.027 | 0.040 |  |  |  |  |  |  |  |  |  |
|  | **est** | **se** | **p** | **CI.lb** | **CI.ub** | **k_sp_** | **k_es_** | | **Var_sp_** | | **Var_phy_** | | **Var_es_** | |
| **Intercept** | **0.207** | **0.038** | **<0.001** | **0.130** | **0.284** | 30 | 128 | | 0.000 | | 0.000 | | 0.168 | |
| Latitude | 0.002 | 0.033 | 0.960 | -0.063 | 0.067 |  |  |  |  |  |  |  |  |  |
| Acclimation time | 0.052 | 0.034 | 0.130 | -0.015 | 0.119 |  |  |  |  |  |  |  |  |  |
| Latitude x Acclimation time | 0.012 | 0.027 | 0.656 | -0.042 | 0.066 |  |  |  |  |  |  |  |  |  |
|  | **est** | **se** | **p** | **CI.lb** | **CI.ub** | **k_sp_** | **k_es_** | | **Var_sp_** | | **Var_phy_** | | **Var_es_** | |
| **Intercept** | **0.239** | **0.038** | **<0.001** | **0.163** | **0.315** | 58 | | 396 | | 0.003 | | 0.002 | | 0.062 |
| Body mass | 0.001 | 0.019 | 0.983 | -0.037 | 0.038 |  |  |  |  |  |  |  |  |  |
| Acclimation time | 0.035 | 0.026 | 0.176 | -0.016 | 0.086 |  |  |  |  |  |  |  |  |  |
| Body mass x Acclimation time | -0.004 | 0.025 | 0.887 | -0.052 | 0.045 |  |  |  |  |  |  |  |  |  |
|  | **est** | **se** | **p** | **CI.lb** | **CI.ub** | **k_sp_** | **k_es_** | | **Var_sp_** | | **Var_phy_** | | **Var_es_** | |
| **Intercept** | **0.230** | **0.028** | **<0.001** | **0.173** | **0.286** | 92 | | 526 | | 0.002 | | 0.002 | | 0.092 |
| Heating rate | 0.031 | 0.016 | 0.060 | -0.001 | 0.063 |  |  |  |  |  |  |  |  |  |
| **Acclimation time** | **0.056** | **0.018** | **0.001** | **0.022** | **0.091** |  |  |  |  |  |  |  |  |  |
| Heating rate x Acclimation time | 0.043 | 0.017 | 0.011 | 0.010 | 0.077 |  |  |  |  |  |  |  |  |  |

**TABLE S41:** **Results of meta-regressions testing for three-way interactive effects of latitude, body mass, and methodology on measured initial developmental plasticity.** Note that these analyses were post-hoc (suggested at the peer-review stage). Body mass, heating rate, and acclimation time were right-skewed and were therefore ln-transformed. Latitude was taken as absolute values. All predictors were standardised. est: estimate; se: standard error; CI.lb: lower bound of the 95% confidence interval; CI.ub: upper bound of the 95% confidence interval; k_sp_: number of species included in the model; k_es_: number of effect sizes included in the model; Var_sp_: variance explained by variation between species; Var_phy_: variance explained by shared evolutionary history; Var_es_: variance associated with the residuals; x: interaction.

|  | **est** | **se** | **p** | **CI.lb** | **CI.ub** | **k_sp_** | **k_es_** | **Var_sp_** | **Var_phy_** | **Var_es_** |
| --- | --- | --- | --- | --- | --- | --- | --- | --- | --- | --- |
| Intercept | 0.276 | 0.117 | 0.051 | -0.001 | 0.553 | 15 | 59 | 0.001 | 0.023 | 0.046 |
| Body mass | -0.029 | 0.057 | 0.614 | -0.143 | 0.085 |  |  |  |  |  |
| Heating rate | -0.047 | 0.109 | 0.669 | -0.267 | 0.173 |  |  |  |  |  |
| Latitude | 0.021 | 0.039 | 0.598 | -0.057 | 0.098 |  |  |  |  |  |
| Body mass x  Heating rate | -0.167 | 0.124 | 0.186 | -0.416 | 0.083 |  |  |  |  |  |
| Body mass x  Latitude | -0.026 | 0.051 | 0.620 | -0.128 | 0.077 |  |  |  |  |  |
| Heating rate x  Latitude | 0.011 | 0.081 | 0.888 | -0.150 | 0.173 |  |  |  |  |  |
| Body mass x  Heating rate x  Latitude | -0.010 | 0.107 | 0.926 | -0.205 | 0.225 |  |  |  |  |  |
|  | **est** | **se** | **p** | **CI.lb** | **CI.ub** | **k_sp_** | **k_es_** | **Var_sp_** | **Var_phy_** | **Var_es_** |
| Intercept | **0.235** | **0.028** | **<0.001** | **0.180** | **0.291** | 57 | 306 | 0.001 | 0.001 | 0.071 |
| Body mass | 0.003 | 0.019 | 0.871 | -0.034 | 0.041 |  |  |  |  |  |
| Heating rate | 0.038 | 0.033 | 0.090 | -0.006 | 0.082 |  |  |  |  |  |
| Acclimation time | 0.036 | 0.028 | 0.196 | -0.019 | 0.091 |  |  |  |  |  |
| Body mass x Heating rate | -0.001 | 0.018 | 0.966 | -0.036 | 0.035 |  |  |  |  |  |
| Body mass x Acclimation time | -0.032 | 0.027 | 0.236 | -0.086 | 0.021 |  |  |  |  |  |
| **Heating rate x Acclimation time** | **0.051** | **0.025** | **0.041** | **0.002** | **0.099** |  |  |  |  |  |
| **Body mass x  Heating rate x  Acclimation time** | **-0.066** | **0.027** | **0.014** | **-0.118** | **-0.013** |  |  |  |  |  |
|  | **est** | **se** | **p** | **CI.lb** | **CI.ub** | **k_sp_** | **k_es_** | **Var_sp_** | **Var_phy_** | **Var_es_** |
| **Intercept** | **0.178** | **0.060** | **0.008** | **0.053** | **0.304** | 29 | 112 | 0.000 | 0.000 | 0.200 |
| Acclimation time | 0.102 | 0.065 | 0.121 | -0.027 | 0.232 |  |  |  |  |  |
| Heating rate | 0.015 | 0.084 | 0.863 | -0.153 | 0.182 |  |  |  |  |  |
| Latitude | 0.020 | 0.044 | 0.645 | -0.067 | 0.107 |  |  |  |  |  |
| Acclimation time x Heating rate | -0.066 | 0.090 | 0.464 | -0.245 | 0.113 |  |  |  |  |  |
| Acclimation time x  Latitude | -0.028 | 0.052 | 0.594 | -0.131 | 0.076 |  |  |  |  |  |
| Heating rate x Latitude | -0.032 | 0.070 | 0.645 | -0.170 | 0.106 |  |  |  |  |  |
| Acclimation time x Heating rate x Latitude | 0.030 | 0.060 | 0.626 | -0.091 | 0.150 |  |  |  |  |  |
|  | **est** | **se** | **p** | **CI.lb** | **CI.ub** | **k_sp_** | **k_es_** | **Var_sp_** | **Var_phy_** | **Var_es_** |
| **Intercept** | **0.257** | **0.094** | **0.035** | **0.026** | **0.488** | 14 | 52 | 0.000 | 0.000 | 0.053 |
| Body mass | -0.039 | 0.115 | 0.734 | -0.270 | 0.192 |  |  |  |  |  |
| Acclimation time | 0.013 | 0.069 | 0.852 | -0.126 | 0.152 |  |  |  |  |  |
| Latitude | -0.016 | 0.059 | 0.794 | -0.135 | 0.104 |  |  |  |  |  |
| Body mass x Acclimation time | -0.030 | 0.108 | 0.786 | -0.248 | 0.189 |  |  |  |  |  |
| Body mass x Latitude | -0.037 | 0.079 | 0.639 | -0.197 | 0.122 |  |  |  |  |  |
| Acclimation time x Latitude | 0.028 | 0.052 | 0.587 | -0.076 | 0.133 |  |  |  |  |  |
| Body mass x  Acclimation time x Latitude | 0.058 | 0.072 | 0.427 | -0.088 | 0.203 |  |  |  |  |  |

**TABLE S42:** **Results of meta-regressions testing for the two-way interactive effects of latitude, body mass, and methodology on measured persistent developmental plasticity.** Note that these analyses were post-hoc (suggested at the peer-review stage). Body mass, heating rate, and acclimation time were right-skewed and were therefore ln-transformed. Latitude was taken as absolute values. All predictors were standardised. The model testing the three-way interaction between latitude, body mass and heating rate failed to converge. est: estimate; se: standard error; CI.lb: lower bound of the 95% confidence interval; CI.ub: upper bound of the 95% confidence interval; k_sp_: number of species included in the model; k_es_: number of effect sizes included in the model; Var_sp_: variance explained by variation between species; Var_phy_: variance explained by shared evolutionary history; Var_es_: variance associated with the residuals; x: interaction.

|  | **est** | **se** | **p** | **CI.lb** | **CI.ub** | **k_sp_** | **k_es_** | | **Var_sp_** | | **Var_phy_** | | **Var_es_** | |
| --- | --- | --- | --- | --- | --- | --- | --- | --- | --- | --- | --- | --- | --- | --- |
| Intercept | 0.136 | 0.078 | 0.330 | -0.849 | 1.121 | 4 | 21 | | 0.001 | | 0.001 | | 0.003 | |
| **Body mass** | **-0.222** | **0.096** | **0.033** | **-0.424** | **-0.021** |  |  |  |  |  |  |  |  |  |
| Latitude | 0.039 | 0.050 | 0.448 | -0.067 | 0.145 |  |  |  |  |  |  |  |  |  |
| Body mass x  Latitude | -0.072 | 0.058 | 0.235 | -0.195 | 0.051 |  |  |  |  |  |  |  |  |  |
|  | **est** | **se** | **p** | **CI.lb** | **CI.ub** | **k_sp_** | **k_es_** | | **Var_sp_** | | **Var_phy_** | | **Var_es_** | |
| Intercept | -0.062 | 0.111 | 0.615 | -0.414 | 0.290 | 7 | 104 | | 0.045 | | 0.001 | | 0.020 | |
| Latitude | 0.174 | 0.09 | 0.069 | -0.014 | 0.361 |  |  |  |  |  |  |  |  |  |
| **Heating rate** | **0.278** | **0.104** | **0.009** | **0.072** | **0.484** |  |  |  |  |  |  |  |  |  |
| Latitude x  Heating rate | 0.018 | 0.099 | 0.861 | -0.179 | 0.214 |  |  |  |  |  |  |  |  |  |
|  | **est** | **se** | **p** | **CI.lb** | **CI.ub** | **k_sp_** | **k_es_** | | **Var_sp_** | | **Var_phy_** | | **Var_es_** | |
| Intercept | 0.011 | 0.063 | 0.869 | -0.144 | 0.166 | 10 | 78 | | 0.001 | | 0.009 | | 0.018 | |
| **Body mass** | **0.066** | **0.031** | **0.036** | **0.004** | **0.127** |  |  |  |  |  |  |  |  |  |
| Heating rate | 0.036 | 0.024 | 0.186 | -0.023 | 0.096 |  |  |  |  |  |  |  |  |  |
| Body mass x  Heating rate | -0.021 | 0.019 | 0.285 | -0.059 | 0.018 |  |  |  |  |  |  |  |  |  |
|  | **est** | **se** | **p** | **CI.lb** | **CI.ub** | **k_sp_** | **k_es_** | | **Var_sp_** | | **Var_phy_** | | **Var_es_** | |
| Intercept | -2.297 | 20.378 | 0.929 | -261.229 | 256.635 | 3 | 28 | | 0.322 | | 0.092 | | 0.060 | |
| Latitude | -3.487 | 25.863 | 0.894 | -56.866 | 49.892 |  |  |  |  |  |  |  |  |  |
| Acclimation time | 1.212 | 9.115 | 0.895 | -17.601 | 20.025 |  |  |  |  |  |  |  |  |  |
| Latitude x Acclimation time | 2.009 | 11.723 | 0.865 | -22.186 | 26.204 |  |  |  |  |  |  |  |  |  |
|  | **est** | **se** | **p** | **CI.lb** | **CI.ub** | **k_sp_** | **k_es_** | | **Var_sp_** | | **Var_phy_** | | **Var_es_** | |
| Intercept | 0.021 | 0.078 | 0.818 | -0.315 | 0.356 | 6 | | 60 | | 0.000 | | 0.010 | | 0.035 |
| Body mass | 0.054 | 0.038 | 0.160 | -0.022 | 0.129 |  |  |  |  |  |  |  |  |  |
| Acclimation time | 0.025 | 0.059 | 0.680 | -0.094 | 0.143 |  |  |  |  |  |  |  |  |  |
| Body mass x Acclimation time | -0.003 | 0.053 | 0.952 | -0.110 | 0.103 |  |  |  |  |  |  |  |  |  |
|  | **est** | **se** | **p** | **CI.lb** | **CI.ub** | **k_sp_** | **k_es_** | | **Var_sp_** | | **Var_phy_** | | **Var_es_** | |
| Intercept | 0.029 | 0.042 | 0.504 | -0.069 | 0.128 | 11 | | 88 | | 0.005 | | 0.000 | | 0.043 |
| Heating rate | 0.037 | 0.034 | 0.321 | -0.044 | 0.117 |  |  |  |  |  |  |  |  |  |
| Acclimation time | 0.021 | 0.033 | 0.535 | -0.452 | 0.086 |  |  |  |  |  |  |  |  |  |
| Heating rate x Acclimation time | -0.006 | 0.016 | 0.694 | -0.038 | 0.026 |  |  |  |  |  |  |  |  |  |

**TABLE S43:** **Results of meta-regressions testing for three-way interactive effects of latitude, body mass, and methodology on measured initial developmental plasticity.** Note that these analyses were post-hoc (suggested at the peer-review stage). Body mass, heating rate, and acclimation time were right-skewed and were therefore ln-transformed. Latitude was taken as absolute values. All predictors were standardised. est: estimate; se: standard error; CI.lb: lower bound of the 95% confidence interval; CI.ub: upper bound of the 95% confidence interval; k_sp_: number of species included in the model; k_es_: number of effect sizes included in the model; Var_sp_: variance explained by variation between species; Var_phy_: variance explained by shared evolutionary history; Var_es_: variance associated with the residuals; x: interaction.

|  | **est** | **se** | **p** | **CI.lb** | **CI.ub** | **k_sp_** | **k_es_** | **Var_sp_** | **Var_phy_** | **Var_es_** |
| --- | --- | --- | --- | --- | --- | --- | --- | --- | --- | --- |
| Intercept | 0.002 | 0.081 | 0.983 | -1.030 | 1.034 | 6 | 59 | 0.000 | 0.000 | 0.037 |
| Body mass | 0.117 | 0.074 | 0.071 | -0.010 | 0.245 |  |  |  |  |  |
| Heating rate | 0.137 | 0.080 | 0.360 | -0.896 | 1.149 |  |  |  |  |  |
| Acclimation time | -0.024 | 0.125 | 0.850 | -0.275 | 0.228 |  |  |  |  |  |
| Body mass x Heating rate | -0.054 | 0.046 | 0.238 | -0.146 | 0.037 |  |  |  |  |  |
| Body mass x Acclimation time | 0.022 | 0.094 | 0.816 | -0.167 | 0.211 |  |  |  |  |  |
| Heating rate x Acclimation time | -0.054 | 0.075 | 0.472 | -0.204 | 0.096 |  |  |  |  |  |
| Body mass x  Heating rate x  Acclimation time | 0.037 | 0.062 | 0.555 | -0.087 | 0.161 |  |  |  |  |  |

**TABLE S44:** **Robustness of our results to the inclusion of body mass and methodological variation in statistical models.** Note that these analyses were post-hoc (suggested at the peer-review stage). Body mass, heating rate, and acclimation time were right-skewed and were therefore ln-transformed. All continuous predictors were standardised. est: estimate; se: standard error; CI.lb: lower bound of the 95% confidence interval; CI.ub: upper bound of the 95% confidence interval; k_sp_: number of species included in the model; k_es_: number of effect sizes included in the model; Var_sp_: variance explained by variation between species; Var_phy_: variance explained by shared evolutionary history; Var_es_: variance associated with the residuals; A. inverts: aquatic invertebrates; T. inverts: terrestrial invertebrates; x: interaction.

|  | **est** | **se** | **p** | **CI.lb** | **CI.ub** | **k_sp_** | **k_es_** | | | | **Var_sp_** | | | | **Var_phy_** | | | | **Var_es_** | | | |
| --- | --- | --- | --- | --- | --- | --- | --- | --- | --- | --- | --- | --- | --- | --- | --- | --- | --- | --- | --- | --- | --- | --- |
| Body mass | 0.003 | 0.021 | 0.906 | -0.039 | 0.044 | 60 | | 365 | | | | 0.004 | | | | 0.003 | | | | 0.066 | | |
| **Heating rate** | **0.043** | **0.022** | **0.047** | **0.001** | **0.085** |  |  |  |  |  |  |  |  |  |  |  |  |  |  |  |  |  |
| **Acclimation time** | **0.057** | **0.036** | **0.031** | **0.005** | **0.108** |  |  |  |  |  |  |  |  |  |  |  |  |  |  |  |  |  |
| **Aquatic** | **0.227** | **0.042** | **<0.001** | **0.143** | **0.312** |  |  |  |  |  |  |  |  |  |  |  |  |  |  |  |  |  |
| Terrestrial | 0.062 | 0.113 | 0.583 | -0.288 | 0.164 |  |  |  |  |  |  |  |  |  |  |  |  |  |  |  |  |  |
| Body mass x Heating rate | 0.001 | 0.018 | 0.951 | -0.035 | 0.037 |  |  |  |  |  |  |  |  |  |  |  |  |  |  |  |  |  |
| Body mass x  Acclimation time | -0.031 | 0.026 | 0.239 | -0.083 | 0.021 |  |  |  |  |  |  |  |  |  |  |  |  |  |  |  |  |  |
| Heating rate x Acclimation time | 0.033 | 0.019 | 0.087 | -0.005 | 0.071 |  |  |  |  |  |  |  |  |  |  |  |  |  |  |  |  |  |
| **Body mass x  Heating rate x Acclimation time** | **-0.057** | **0.024** | **0.019** | **-0.104** | **-0.009** |  |  |  |  |  |  |  |  |  |  |  |  |  |  |  |  |  |
|  | **est** | **se** | **p** | **CI.lb** | **CI.ub** | **k_sp_** | **k_es_** | | | | **Var_sp_** | | | | **Var_phy_** | | | | **Var_es_** | | | |
| Body mass | 0.021 | 0.021 | 0.319 | -0.021 | 0.063 | 60 | | | 365 | | | | 0.005 | | | | 0.000 | | | | 0.067 | |
| Heating rate | 0.036 | 0.022 | 0.093 | -0.006 | 0.079 |  |  |  |  |  |  |  |  |  |  |  |  |  |  |  |  |  |
| Acclimation time | 0.049 | 0.026 | 0.063 | -0.003 | 0.100 |  |  |  |  |  |  |  |  |  |  |  |  |  |  |  |  |  |
| **Amphibians** | **0.304** | **0.098** | **0.003** | **0.106** | **0.501** |  |  |  |  |  |  |  |  |  |  |  |  |  |  |  |  |  |
| **A. inverts** | **0.309** | **0.064** | **<0.001** | **0.180** | **0.438** |  |  |  |  |  |  |  |  |  |  |  |  |  |  |  |  |  |
| **Fishes** | **0.193** | **0.027** | **<0.001** | **0.140** | **0.247** |  |  |  |  |  |  |  |  |  |  |  |  |  |  |  |  |  |
| Reptiles | 0.021 | 0.087 | 0.815 | -0.155 | 0.196 |  |  |  |  |  |  |  |  |  |  |  |  |  |  |  |  |  |
| T. inverts | 0.108 | 0.137 | 0.436 | -0.168 | 0.384 |  |  |  |  |  |  |  |  |  |  |  |  |  |  |  |  |  |
| Body mass x Heating rate | 0.007 | 0.019 | 0.716 | -0.030 | 0.044 |  |  |  |  |  |  |  |  |  |  |  |  |  |  |  |  |  |
| Body mass x  Acclimation time | -0.021 | 0.027 | 0.434 | -0.073 | 0.032 |  |  |  |  |  |  |  |  |  |  |  |  |  |  |  |  |  |
| Heating rate x Acclimation time | 0.029 | 0.020 | 0.132 | -0.009 | 0.068 |  |  |  |  |  |  |  |  |  |  |  |  |  |  |  |  |  |
| **Body mass x  Heating rate x Acclimation time** | **-0.055** | **0.024** | **0.024** | **-0.102** | **-0.007** |  |  |  |  |  |  |  |  |  |  |  |  |  |  |  |  |  |
|  | **est** | **se** | **p** | **CI.lb** | **CI.ub** | **k_sp_** | **k_es_** | | | | **Var_sp_** | | | | **Var_phy_** | | | | **Var_es_** | | | |
| Body mass | 0.008 | 0.018 | 0.673 | -0.028 | 0.043 | 60 | | | | 365 | | | | 0.001 | | | | 0.001 | | | | 0.067 |
| Heating rate | 0.036 | 0.020 | 0.071 | -0.003 | 0.075 |  |  |  |  |  |  |  |  |  |  |  |  |  |  |  |  |  |
| Acclimation time | 0.035 | 0.025 | 0.158 | -0.014 | 0.084 |  |  |  |  |  |  |  |  |  |  |  |  |  |  |  |  |  |
| Design A | 0.235 | 0.029 | <0.001 | 0.179 | 0.084 |  |  |  |  |  |  |  |  |  |  |  |  |  |  |  |  |  |
| Design C | 0.119 | 0.272 | 0.665 | -0.427 | 0.664 |  |  |  |  |  |  |  |  |  |  |  |  |  |  |  |  |  |
| Design D | -0.076 | 0.061 | 0.214 | -0.195 | 0.044 |  |  |  |  |  |  |  |  |  |  |  |  |  |  |  |  |  |
| Design F | 0.122 | 0.088 | 0.165 | -0.050 | 0.294 |  |  |  |  |  |  |  |  |  |  |  |  |  |  |  |  |  |
| Body mass x Acclimation time | 0.005 | 0.017 | 0.753 | -0.028 | 0.038 |  |  |  |  |  |  |  |  |  |  |  |  |  |  |  |  |  |
| Body mass x  Acclimation time | -0.023 | 0.024 | 0.350 | -0.071 | 0.025 |  |  |  |  |  |  |  |  |  |  |  |  |  |  |  |  |  |
| Heating rate x Acclimation time | 0.035 | 0.018 | 0.057 | -0.001 | 0.071 |  |  |  |  |  |  |  |  |  |  |  |  |  |  |  |  |  |
| **Body mass x  Heating rate x Acclimation time** | **-0.053** | **0.023** | **0.022** | **-0.098** | **-0.008** |  |  |  |  |  |  |  |  |  |  |  |  |  |  |  |  |  |

**TABLE S45:** **Main packages and functions used for statistical analyses and figures.** For further details about how these functions were implemented, refer to Supporting information S2.

| **Package** | **Functions** | **Use** |
| --- | --- | --- |
| **rotl** v. 3.0.11 (Michonneau et al., 2016) | tnrs_match_names, tol_induced_subtree, | Match taxonomic names to the Open Tree Taxonomy, build phylogenetic tree based on nodes in the Open Tree Taxonomy |
| **ape** v. 5.5  (Paradis & Schliep, 2019) | multi2di, compute.brlen,  vcv | Resolve polytomies at random in phylogenetic trees, compute branch lengths of a tree, generate a correlation matrix between species in a tree |
| **metafor**  v. 3.0-2  (Viechtbauer, 2010) | rma.mv, funnel, | Fit a multi-level meta-analytic model, create funnel plot |
| **metaAidR**  v. 0.0.0.9000  (github.com/daniel1noble/metaAidR) | make_VCV_matrix | Generate a variance covariance matrix between sampling variances |
| **orchaRd**  v. 2.0  (Nakagawa et al., 2020) | i2_ml,  r2_ml,  orchard_plot,  mod_results  marginal_means | Calculate heterogeneity, calculate the percentage of variance explained by fixed and random effects, create and orchard_plot, generate table of model results with mean estimates, confidence, and prediction intervals, estimate marginal means using some properties of emmeans. |
| **emmeans**  v. 1.7.2-9000003  (Length, 2019) | emmeans | Compute estimated marginal means. |
| **MuMIn**  v. 1.43.17  (Barton, 2015) | dredge,  sw | Generate a model selection of models with combinations of moderators, generate the sum of model weights over all models including each explanatory variable. |

**References**

Gelman, A., Hill, J., & Vehtari, A. (2020). Poststratification and missing-data imputation. In: Regression and other stories. Cambridge University Press, Cambridge, United Kingdom, pp. 313-336.

Barton, K. (2020). MuMIn: Multi-model inference. R package version 1.43.17. Retrieved from https://cran.r-project.org/web/packages/MuMIn/.

Cheung, K. (2019). The effects of embryonic incubation temperature on subsequent development, growth, and thermal tolerance through early ontogeny of White Sturgeon. PhD Thesis. University of British Columbia.

Foo, Y. Z., O'Dea, R. E., Koricheva, J., Nakagawa, S., & Lagisz, M. (2021). A practical guide to question formation, systematic searching and study screening for literature reviews in ecology and evolution. Methods in Ecology and Evolution, 12, 1705-1720.

Lenth, R., Singmann, H., Love, J., Buerkner, P., & Herve, M. (2019). emmeans: Estimated marginal means, aka least-squares means. R package version 1.7.2-9000003.

Michonneau, F., Brown, J.W. & Winter, D.J. (2016). rotl: an R package to interact with the Open Tree of Life data. Methods in Ecology and Evolution, 7, 1476–1481.

Nakagawa, S., Lagisz, M., O'Dea, R. E., Rutkowska, J., Yang, Y., Noble, D. W., & Senior, A. M. (2021). The orchard plot: cultivating a forest plot for use in ecology, evolution, and beyond. Research Synthesis Methods, 12, 4-12.

Ouzzani, M., Hammady, H., Fedorowicz, Z. & Elmagarmid, A. (2016). Rayyan—a web and mobile app for systematic reviews. Syst Rev, 5, 210.

Paradis, E. & Schliep, K. (2019). ape 5.0: an environment for modern phylogenetics and evolutionary analyses in R. Bioinformatics, 35, 526–528.

Viechtbauer, W. (2010). Conducting meta-analyses in R with the metafor package. Journal of Statistical Software, 36, 1–48.
