# Supplementary material for: Developmental plasticity in thermal tolerance: Ontogenetic variation, persistence, and future directions
Source: Ecol Lett. 2022 Aug 25;25(10):2245–68. doi: 10.1111/ele.14083 (PMC9804923; doi:10.1111/ele.14083)
Supplement: Supplementary file 2 — Appendix S2 [file ELE-25-2245-s002.html]

 
 
   
     
     
     

         
    
    
     Developmental plasticity in thermal tolerance: ontogenetic variation, persistence and future directions 

         
         
         
         
         
         
         
         
         
         
         
         
         
         
         
         
         
    
    
         
     
    
    
     
     

     
         
    
     
         
    
     

     
    
 

 

   	
             
    
      
           
      
         
       
       Code     
      
	    	      Show All Code  
	      Hide All Code  
	    	      
	    	    	    	      Download Rmd  
	          
    
   
        
       Developmental plasticity in thermal tolerance:
ontogenetic variation, persistence and future directions 
          Supporting Information S2 
   
          
    
      Developmental plasticity in thermal
tolerance: ontogenetic variation, persistence and future
directions  
     
       
         Load packages and
      data  
       
        Load packages  
        Load data  
        
         Data
      processing   
         Calculate effect
      sizes  
       
        Impute missing
      standard deviations  
        Calculate
      effect sizes and sampling variances  
        Merge
      designs E and F into the same design and rename design G  
        
         Data
      exploration  
       
        Check for
      extreme values and/or studies  
        Missing data
      patterns  
        Frequency of
      the different variables  
        Map
      effect sizes against all extracted moderators 
       
        Habitat type  
        Taxonomic group  
        Time in a common
      garden condition  
        Experimental design  
        initial
      vs. persistent effects  
        Metric type  
        Heating rate  
        Viviparous or
      Oviparous exposure  
        Sex  
        Age tested  
        Heat tolerance
      endpoint  
        Hatching origin  
        Body mass  
        Whether
      the acclimation temperatures used are independent from housing
      temperatures prior to acclimation  
        The
      time after which survival was assessed in static assays  
        Concerns with data
      quality  
        Imputed vs original
      data  
          
        
        
         Phylogeny  
       
        Plot the tree  
        
         Account
      for treatments re-used in multiple comparisons   
         Sample sizes   
         Save processed
      data   
         Choose
      random effect structure   
         Custom
      functions  
       
        Run models  
        Plot orchaRd plots  
        Plot continuous
      moderator  
        
         Intercept
      Meta-analytic model   
         Single
      moderator metaregressions  
       
         Habitat  
       
        Individual
      coefficients  
        Contrasts  
        Heteroscedasticity
      check  
        Individual
      coefficients (with heteroscedasticity)  
        Contrasts (with
      heteroscedasticity)  
          
        
         Taxonomic groups  
       
        Individual
      coefficients  
        Contrasts  
        Heteroscedasticity
      check  
        Individual
      coefficients (with heteroscedasticity)  
        Contrasts (with
      heteroscedasticity)  
          
        
         Persistence of
      developmental effects  
       
        Individual
      coefficients  
        Contrasts  
        Heteroscedasticity
      check  
        Individual
      coefficients (with heteroscedasticity)  
        Contrasts (with
      heteroscedasticity)  
          
        
         Life
      history variation and persistence  
       
        Individual
      coefficients  
        Contrasts  
        Heteroscedasticity
      check  
        Individual
      coefficients (with heteroscedasticity)  
        Contrasts (with
      heteroscedasticity)  
          
        
         Time in a
      common garden condition  
       
        Run model and plot
      results  
        Heteroscedasticity
      check  
          
        
         Type of metric  
       
        Individual
      coefficients  
        Contrasts  
        Heteroscedasticity
      check  
        Individual
      coefficients (with heteroscedasticity)  
        Contrasts (with
      heteroscedasticity)  
          
        
         Heating rate  
       
        Run model and plot
      results  
        Heteroscedasticity
      check  
          
        
        
         Mean
      adjusted for habitat differences  
       
        Overall
      mean with habitat weighted equally  
        
         Multi-model
      inference  
       
        Best models 
       
        Best model  
        Second best model  
        Third best model  
        Fourth best model  
        Fifth best model  
          
        
        
         Publication
      bias  
       
        Funnel plot  
        Data type 
       
        Run model and plot
      results  
        Heteroscedasticity
      check  
        Contrasts  
        
        Publication type 
       
        Run model and plot
      results  
        Heteroscedasticity
      check  
        Contrasts  
        
        Publication year 
       
        Run model and plot
      results  
        Heteroscedasticity
      check  
        
        Egger’s regression  
        Sampling
      variance fitted as a moderator  
        
         Sensitivity
      analyses  
       
         Leave-one-out
      analyses  
       
        Leave one study out  
        Leave one species
      out  
          
        
         Analyses
      with initial designs only  
       
        Habitat 
       
        Individual
      coefficients  
        Heteroscedasticity
      check  
        Individual
      coefficients (with heteroscedasticity)  
        Mean
      initial effects with habitat weighted equally  
        
        Taxonomic groups 
       
        Individual
      coefficients  
        Heteroscedasticity
      check  
        Individual
      coefficients (with heteroscedasticity)  
        
        Type of metric 
       
        Individual
      coefficients  
        Heteroscedasticity
      check  
        Individual
      coefficients (with heteroscedasticity)  
        
        Heating rate 
       
        Run model and plot
      results  
        
          
        
         Analyses
      with persistent designs only  
       
        Habitat 
       
        Individual
      coefficients  
        Heteroscedasticity
      check  
        Individual
      coefficients (with heteroscedasticity)  
        Mean
      persistent effects with habitat weighted equally  
        
        Taxonomic groups 
       
        Individual
      coefficients  
        Heteroscedasticity
      check  
        Individual
      coefficients (with heteroscedasticity)  
        
        Type of metric 
       
        Individual
      coefficients  
        Heteroscedasticity
      check  
        Individual
      coefficients (with heteroscedasticity)  
        
        Heating rate 
       
        Run model and plot
      results  
        
          
        
         Risk of bias
      analysis  
       
        Analysis without
      imputed values  
        Analysis without
      concerning data  
        Analyses
      without extreme values of dARR  
          
        
         Comparisons
      with the data from Morley et al. 2019.
      10.1111/geb.12911   
        
         Post-hoc
      analyses  
       
        Body mass 
       
        Full dataset  
        Initial effects  
        Persistent effects  
        
        Age at sexual maturity 
       
        Full dataset  
        Initial effects  
        Persistent effects  
        
        Time in a
      common garden condition 
       
        Accounting
      for differences in life span  
        Life
      history variation after accounting for different re-acclimation
      times  
        
        Interactions 
       
        Full dataset 
       
        Latitude x Body mass  
        Latitude x Heating
      rate  
        Latitude x
      Acclimation duration  
        Body mass x Heating
      rate  
        Body mass x
      Acclimation duration  
        Heating rate x
      Acclimation duration  
        Latitude x Body
      mass x Heating rate  
        Latitude x
      Body mass x Acclimation duration  
        Latitude x
      Heating rate x Acclimation duration  
        Body size
      x Heating rate x Acclimation duration  
        Habitat
      variation after accounting for methodological and body mass
      variation  
        Taxonomic
      variation after accounting for methodological and body mass
      variation  
        Life
      history variation after accounting for methodological and body
      mass variation  
        
        Initial effects 
       
        Latitude x Body
      mass  
        Latitude x Heating
      rate  
        Latitude x
      Acclimation duration  
        Body mass x Heating
      rate  
        Body mass x
      Acclimation duration  
        Heating rate
      x Acclimation duration  
        Latitude x
      Body mass x Heating rate  
        Latitude x
      Body mass x Acclimation duration  
        Latitude
      x Heating rate x Acclimation duration  
        Body
      size x Heating rate x Acclimation duration  
        
        Persistent effects 
       
        Latitude x Body
      mass  
        Latitude x Heating
      rate  
        Latitude x
      Acclimation duration  
        Body mass x Heating
      rate  
        Body mass x
      Acclimation duration  
        Heating rate
      x Acclimation duration  
        Latitude x
      Body mass x Heating rate  
        Latitude x
      Body mass x Acclimation duration  
        Latitude
      x Heating rate x Acclimation duration  
        Body
      size x Heating rate x Acclimation duration  
        
        
        Exclude
      temperature tolerated for one hour  
        
         Figures for
      publication  
       
        Figure 2 (Design
      representation)  
        Figure 3 (Phylogeny)  
        Figure 4 (Meta-analytic
      mean)  
        Figure 5
      (Life history variation and persistence)  
        Figure 6 (Habitat
      variation)  
        Figure 7 (Taxonomic
      variation)  
        
         Software and
      packages versions   
       
     
     
                      Patrice
Pottier, Samantha Burke, Rose Y. Zhang, Daniel W.A. Noble, Lisa E.
Schwanz, Szymon M. Drobniak, Shinichi Nakagawa 
                            latest
update: 22 June 2022 
           
    
     

   
      
   
  
 
 
  Load packages and data  
 
 Load packages 
      pacman ::  p_load (tidyverse, 
                  kableExtra, 
                  DataExplorer, 
                  viridis, 
                  viridisLite, 
                  maps, 
                  rotl, 
                  ape, 
                  patchwork, 
                  R.utils, 
                  ggtree,  # devtools::install_github("YuLab-SMU/ggtree")  
                  ggtreeExtra,  # devtools::install_github("xiangpin/ggtreeExtra")  
                  phytools, 
                  tidytree, 
                  ggnewscale,  
                  RColorBrewer, 
                  metafor, 
                  metaAidR,  # devtools::install_github("daniel1noble/metaAidR")  
                  orchaRd,  # devtools::install_github("daniel1noble/orchaRd")  
                  ggdist, 
                  ggstatsplot, 
                  here, 
                  ggExtra,  
                  MuMIn)  
    
    library ( "groundhog" ) 
    groundhog.library ( "emmeans" ,  "2022-04-23" )     
 
 
 Load data 
      d.raw  <-   read_csv ( "data/Raw_data.csv" ) 
    
    # Reformat ID variables as characters  
   d.raw $ species_ID  =   as.character (d.raw $ species_ID) 
   d.raw $ study_ID  =   as.character (d.raw $ study_ID) 
   d.raw $ population_ID  =   as.character (d.raw $ population_ID) 
   d.raw $ family_ID  =   as.character (d.raw $ family_ID) 
   d.raw $ shared_trt_ID  =   as.character (d.raw $ shared_trt_ID) 
   d.raw $ cohort_ID  =   as.character (d.raw $ cohort_ID) 
   d.raw $ es_ID  =   as.character (d.raw $ es_ID) 
    
    # Quick summary of the data set  
    kable ( summary (d.raw),  "html" )  %>%  
        kable_styling ( "striped" ,  position =   "left" )  %>%  
        scroll_box ( width =   "100%" ,  height =   "600px" )    
 
 
 
 
 
 
 
initials
 
 
es_ID
 
 
study_ID
 
 
species_ID
 
 
population_ID
 
 
family_ID
 
 
shared_trt_ID
 
 
cohort_ID
 
 
note_ID
 
 
data_source
 
 
data_url
 
 
fig_file_name
 
 
data_type
 
 
data_file_name
 
 
peer-reviewed
 
 
ref
 
 
title
 
 
pub_year
 
 
journal
 
 
thesis_chapter
 
 
doi
 
 
citation
 
 
phylum
 
 
class
 
 
order
 
 
family
 
 
genus
 
 
species
 
 
genus_species
 
 
age_maturity
 
 
ref_age_maturity
 
 
habitat
 
 
taxonomic_group
 
 
reproduction_mode
 
 
life_stage_manip
 
 
life_stage_tested
 
 
brought_common_temp
 
 
mobility_life_stage_manip
 
 
time_common_temp
 
 
common_temp
 
 
exp_design
 
 
origin_hatching
 
 
latitude
 
 
longitude
 
 
elevation
 
 
season
 
 
year
 
 
body_length
 
 
body_mass
 
 
age_tested
 
 
sex
 
 
housing_temp
 
 
incubation_independent
 
 
metric
 
 
endpoint
 
 
acc_temp_low
 
 
acc_temp_high
 
 
acc_temp_var
 
 
is_acc_temp_fluctuating
 
 
acc_duration
 
 
ramping
 
 
set_time
 
 
n_test_temp
 
 
n_replicates_per_temp
 
 
n_animals_per_replicate
 
 
humidity
 
 
oxygen
 
 
salinity
 
 
pH
 
 
photoperiod
 
 
gravidity
 
 
starved
 
 
minor_concerns
 
 
major_concerns
 
 
notes_moderators
 
 
mean_HT_low
 
 
sd_HT_low
 
 
n_HT_low
 
 
mean_HT_high
 
 
sd_HT_high
 
 
n_HT_high
 
 
error_type
 
 
notes_es
 
 
 
 
 
 
 
 
Length:1131
 
 
Length:1131
 
 
Length:1131
 
 
Length:1131
 
 
Length:1131
 
 
Length:1131
 
 
Length:1131
 
 
Length:1131
 
 
Length:1131
 
 
Length:1131
 
 
Length:1131
 
 
Length:1131
 
 
Length:1131
 
 
Length:1131
 
 
Length:1131
 
 
Length:1131
 
 
Length:1131
 
 
Min. :1960
 
 
Length:1131
 
 
Min. :1.000
 
 
Length:1131
 
 
Length:1131
 
 
Length:1131
 
 
Length:1131
 
 
Length:1131
 
 
Length:1131
 
 
Length:1131
 
 
Length:1131
 
 
Length:1131
 
 
Min. : 0.00821
 
 
Length:1131
 
 
Length:1131
 
 
Length:1131
 
 
Length:1131
 
 
Length:1131
 
 
Length:1131
 
 
Length:1131
 
 
Length:1131
 
 
Min. : 0.125
 
 
Min. : 6.0
 
 
Length:1131
 
 
Length:1131
 
 
Min. :-41.87
 
 
Min. :-128.23
 
 
Min. : -14.0
 
 
Length:1131
 
 
Min. :1957
 
 
Min. : 0.1782
 
 
Min. : 0.000
 
 
Min. : 0.00
 
 
Length:1131
 
 
Min. : 2.00
 
 
Length:1131
 
 
Length:1131
 
 
Length:1131
 
 
Min. : 0.50
 
 
Min. : 5.00
 
 
Min. : 0.0850
 
 
Length:1131
 
 
Min. : 0.9167
 
 
Min. :0.00069
 
 
Min. : 0.0028
 
 
Min. : 2.000
 
 
Min. : 1.000
 
 
Min. : 1.00
 
 
Min. :60.00
 
 
Min. : 1.308
 
 
Min. : 0.30
 
 
Min. :6.800
 
 
Min. : 8.00
 
 
Length:1131
 
 
Length:1131
 
 
Length:1131
 
 
Length:1131
 
 
Length:1131
 
 
Min. :14.19
 
 
Min. : 0.02362
 
 
Min. : 2.00
 
 
Min. :15.38
 
 
Min. : 0.0000
 
 
Min. : 2.0
 
 
Length:1131
 
 
Length:1131
 
 
 
 
 
 
Class :character
 
 
Class :character
 
 
Class :character
 
 
Class :character
 
 
Class :character
 
 
Class :character
 
 
Class :character
 
 
Class :character
 
 
Class :character
 
 
Class :character
 
 
Class :character
 
 
Class :character
 
 
Class :character
 
 
Class :character
 
 
Class :character
 
 
Class :character
 
 
Class :character
 
 
1st Qu.:1998
 
 
Class :character
 
 
1st Qu.:3.000
 
 
Class :character
 
 
Class :character
 
 
Class :character
 
 
Class :character
 
 
Class :character
 
 
Class :character
 
 
Class :character
 
 
Class :character
 
 
Class :character
 
 
1st Qu.: 0.50000
 
 
Class :character
 
 
Class :character
 
 
Class :character
 
 
Class :character
 
 
Class :character
 
 
Class :character
 
 
Class :character
 
 
Class :character
 
 
1st Qu.: 8.000
 
 
1st Qu.:14.9
 
 
Class :character
 
 
Class :character
 
 
1st Qu.: 26.83
 
 
1st Qu.: -96.91
 
 
1st Qu.: 0.0
 
 
Class :character
 
 
1st Qu.:2009
 
 
1st Qu.: 14.0000
 
 
1st Qu.: 0.310
 
 
1st Qu.: 5.00
 
 
Class :character
 
 
1st Qu.:17.00
 
 
Class :character
 
 
Class :character
 
 
Class :character
 
 
1st Qu.:13.00
 
 
1st Qu.:18.00
 
 
1st Qu.: 0.2000
 
 
Class :character
 
 
1st Qu.: 7.0000
 
 
1st Qu.:0.10000
 
 
1st Qu.: 1.0000
 
 
1st Qu.: 5.000
 
 
1st Qu.: 1.000
 
 
1st Qu.: 1.00
 
 
1st Qu.:65.00
 
 
1st Qu.: 5.500
 
 
1st Qu.:30.00
 
 
1st Qu.:7.550
 
 
1st Qu.:12.00
 
 
Class :character
 
 
Class :character
 
 
Class :character
 
 
Class :character
 
 
Class :character
 
 
1st Qu.:28.58
 
 
1st Qu.: 0.32097
 
 
1st Qu.: 8.00
 
 
1st Qu.:29.57
 
 
1st Qu.: 0.2901
 
 
1st Qu.: 7.0
 
 
Class :character
 
 
Class :character
 
 
 
 
 
 
Mode :character
 
 
Mode :character
 
 
Mode :character
 
 
Mode :character
 
 
Mode :character
 
 
Mode :character
 
 
Mode :character
 
 
Mode :character
 
 
Mode :character
 
 
Mode :character
 
 
Mode :character
 
 
Mode :character
 
 
Mode :character
 
 
Mode :character
 
 
Mode :character
 
 
Mode :character
 
 
Mode :character
 
 
Median :2012
 
 
Mode :character
 
 
Median :4.000
 
 
Mode :character
 
 
Mode :character
 
 
Mode :character
 
 
Mode :character
 
 
Mode :character
 
 
Mode :character
 
 
Mode :character
 
 
Mode :character
 
 
Mode :character
 
 
Median : 1.79000
 
 
Mode :character
 
 
Mode :character
 
 
Mode :character
 
 
Mode :character
 
 
Mode :character
 
 
Mode :character
 
 
Mode :character
 
 
Mode :character
 
 
Median : 14.000
 
 
Median :23.0
 
 
Mode :character
 
 
Mode :character
 
 
Median : 37.26
 
 
Median : -73.85
 
 
Median : 30.0
 
 
Mode :character
 
 
Median :2012
 
 
Median : 48.0000
 
 
Median : 1.900
 
 
Median : 20.50
 
 
Mode :character
 
 
Median :20.00
 
 
Mode :character
 
 
Mode :character
 
 
Mode :character
 
 
Median :20.00
 
 
Median :25.00
 
 
Median : 0.3000
 
 
Mode :character
 
 
Median : 14.0000
 
 
Median :0.25000
 
 
Median : 16.6667
 
 
Median : 7.000
 
 
Median : 3.000
 
 
Median : 8.00
 
 
Median :70.00
 
 
Median : 6.600
 
 
Median :30.00
 
 
Median :7.963
 
 
Median :12.00
 
 
Mode :character
 
 
Mode :character
 
 
Mode :character
 
 
Mode :character
 
 
Mode :character
 
 
Median :35.00
 
 
Median : 0.54000
 
 
Median : 12.00
 
 
Median :36.10
 
 
Median : 0.5201
 
 
Median : 11.0
 
 
Mode :character
 
 
Mode :character
 
 
 
 
 
 
NA
 
 
NA
 
 
NA
 
 
NA
 
 
NA
 
 
NA
 
 
NA
 
 
NA
 
 
NA
 
 
NA
 
 
NA
 
 
NA
 
 
NA
 
 
NA
 
 
NA
 
 
NA
 
 
NA
 
 
Mean :2006
 
 
NA
 
 
Mean :3.707
 
 
NA
 
 
NA
 
 
NA
 
 
NA
 
 
NA
 
 
NA
 
 
NA
 
 
NA
 
 
NA
 
 
Mean : 3.28359
 
 
NA
 
 
NA
 
 
NA
 
 
NA
 
 
NA
 
 
NA
 
 
NA
 
 
NA
 
 
Mean : 33.233
 
 
Mean :20.1
 
 
NA
 
 
NA
 
 
Mean : 27.35
 
 
Mean : -35.99
 
 
Mean : 288.1
 
 
NA
 
 
Mean :2008
 
 
Mean : 57.9212
 
 
Mean : 8.204
 
 
Mean : 67.92
 
 
NA
 
 
Mean :20.44
 
 
NA
 
 
NA
 
 
NA
 
 
Mean :18.39
 
 
Mean :23.16
 
 
Mean : 0.5805
 
 
NA
 
 
Mean : 21.9179
 
 
Mean :0.38546
 
 
Mean : 50.0755
 
 
Mean : 7.761
 
 
Mean : 3.453
 
 
Mean : 13.07
 
 
Mean :68.55
 
 
Mean : 6.691
 
 
Mean :29.81
 
 
Mean :7.878
 
 
Mean :12.89
 
 
NA
 
 
NA
 
 
NA
 
 
NA
 
 
NA
 
 
Mean :34.21
 
 
Mean : 0.82568
 
 
Mean : 18.39
 
 
Mean :35.01
 
 
Mean : 0.7719
 
 
Mean : 18.2
 
 
NA
 
 
NA
 
 
 
 
 
 
NA
 
 
NA
 
 
NA
 
 
NA
 
 
NA
 
 
NA
 
 
NA
 
 
NA
 
 
NA
 
 
NA
 
 
NA
 
 
NA
 
 
NA
 
 
NA
 
 
NA
 
 
NA
 
 
NA
 
 
3rd Qu.:2018
 
 
NA
 
 
3rd Qu.:5.000
 
 
NA
 
 
NA
 
 
NA
 
 
NA
 
 
NA
 
 
NA
 
 
NA
 
 
NA
 
 
NA
 
 
3rd Qu.: 3.00000
 
 
NA
 
 
NA
 
 
NA
 
 
NA
 
 
NA
 
 
NA
 
 
NA
 
 
NA
 
 
3rd Qu.: 40.000
 
 
3rd Qu.:25.0
 
 
NA
 
 
NA
 
 
3rd Qu.: 49.85
 
 
3rd Qu.: 10.21
 
 
3rd Qu.: 285.0
 
 
NA
 
 
3rd Qu.:2016
 
 
3rd Qu.:100.0000
 
 
3rd Qu.:11.000
 
 
3rd Qu.: 60.00
 
 
NA
 
 
3rd Qu.:26.00
 
 
NA
 
 
NA
 
 
NA
 
 
3rd Qu.:24.00
 
 
3rd Qu.:28.00
 
 
3rd Qu.: 1.0000
 
 
NA
 
 
3rd Qu.: 30.0000
 
 
3rd Qu.:0.50000
 
 
3rd Qu.: 96.0000
 
 
3rd Qu.: 8.000
 
 
3rd Qu.: 5.000
 
 
3rd Qu.: 17.50
 
 
3rd Qu.:70.00
 
 
3rd Qu.: 7.300
 
 
3rd Qu.:35.00
 
 
3rd Qu.:8.130
 
 
3rd Qu.:14.00
 
 
NA
 
 
NA
 
 
NA
 
 
NA
 
 
NA
 
 
3rd Qu.:39.91
 
 
3rd Qu.: 0.99437
 
 
3rd Qu.: 20.00
 
 
3rd Qu.:40.73
 
 
3rd Qu.: 0.9305
 
 
3rd Qu.: 20.0
 
 
NA
 
 
NA
 
 
 
 
 
 
NA
 
 
NA
 
 
NA
 
 
NA
 
 
NA
 
 
NA
 
 
NA
 
 
NA
 
 
NA
 
 
NA
 
 
NA
 
 
NA
 
 
NA
 
 
NA
 
 
NA
 
 
NA
 
 
NA
 
 
Max. :2021
 
 
NA
 
 
Max. :5.000
 
 
NA
 
 
NA
 
 
NA
 
 
NA
 
 
NA
 
 
NA
 
 
NA
 
 
NA
 
 
NA
 
 
Max. :19.48528
 
 
NA
 
 
NA
 
 
NA
 
 
NA
 
 
NA
 
 
NA
 
 
NA
 
 
NA
 
 
Max. :182.500
 
 
Max. :36.0
 
 
NA
 
 
NA
 
 
Max. :110.32
 
 
Max. : 174.80
 
 
Max. :3324.0
 
 
NA
 
 
Max. :2019
 
 
Max. :344.0000
 
 
Max. :88.345
 
 
Max. :758.00
 
 
NA
 
 
Max. :30.00
 
 
NA
 
 
NA
 
 
NA
 
 
Max. :37.00
 
 
Max. :38.00
 
 
Max. :10.0000
 
 
NA
 
 
Max. :426.0000
 
 
Max. :3.00000
 
 
Max. :168.0000
 
 
Max. :20.000
 
 
Max. :24.000
 
 
Max. :100.00
 
 
Max. :76.00
 
 
Max. :10.450
 
 
Max. :40.00
 
 
Max. :8.600
 
 
Max. :16.00
 
 
NA
 
 
NA
 
 
NA
 
 
NA
 
 
NA
 
 
Max. :53.90
 
 
Max. :15.51098
 
 
Max. :480.00
 
 
Max. :58.70
 
 
Max. :24.9888
 
 
Max. :480.0
 
 
NA
 
 
NA
 
 
 
 
 
 
NA
 
 
NA
 
 
NA
 
 
NA
 
 
NA
 
 
NA
 
 
NA
 
 
NA
 
 
NA
 
 
NA
 
 
NA
 
 
NA
 
 
NA
 
 
NA
 
 
NA
 
 
NA
 
 
NA
 
 
NA
 
 
NA
 
 
NA’s :1056
 
 
NA
 
 
NA
 
 
NA
 
 
NA
 
 
NA
 
 
NA
 
 
NA
 
 
NA
 
 
NA
 
 
NA’s :146
 
 
NA
 
 
NA
 
 
NA
 
 
NA
 
 
NA
 
 
NA
 
 
NA
 
 
NA
 
 
NA’s :926
 
 
NA’s :908
 
 
NA
 
 
NA
 
 
NA’s :823
 
 
NA’s :823
 
 
NA’s :828
 
 
NA
 
 
NA’s :812
 
 
NA’s :708
 
 
NA’s :623
 
 
NA’s :733
 
 
NA
 
 
NA’s :384
 
 
NA
 
 
NA
 
 
NA
 
 
NA
 
 
NA
 
 
NA’s :538
 
 
NA
 
 
NA’s :305
 
 
NA’s :272
 
 
NA’s :858
 
 
NA’s :880
 
 
NA’s :878
 
 
NA’s :901
 
 
NA’s :1091
 
 
NA’s :974
 
 
NA’s :915
 
 
NA’s :969
 
 
NA’s :582
 
 
NA
 
 
NA
 
 
NA
 
 
NA
 
 
NA
 
 
NA
 
 
NA’s :107
 
 
NA’s :21
 
 
NA
 
 
NA’s :113
 
 
NA’s :21
 
 
NA
 
 
NA
 
 
 
 
 
       # 1131 effect sizes 158 studies 147 species 251 populations     
 
 
 
  Data processing  
 On 2022/01/13, we noticed few mistakes in the extracted data. We also
had concerns about one study. Specifically, two different species_ID
were assigned to  Ischura elegans  and  Litopenaeus
vannamei . We also found that  Lampsilis abrupta ,
 Lampsilis radiata  and  Lampsilis cariosa  were
mistakenly each assigned the same species_ID as  Myxocyprinus
asiaticus ,  Procypris rabaudi  and  Schizopygopsis
younghusbandi , respectively. 
 The study from Buckley and Nufio (2014) Conservation Physiology, was
also found not to be eligible to our inclusion criteria. The main author
of the study indicated to us that  “The acclimation to 25C was short
term (1-2 hours) immediately before CTmax measurements. Animals were
raised at either 24 or 27C until adulthood (and CTmax
measurements)”  which suggests an overlap between juvenile and adult
acclimation. In additon, the re-acclimation period turned out to be
short to be considered relevant in our study. 
 On 2022/01/21, we also realised that the equation 6 (imputation of
missing standard deviations) is not valid to impute missing standard
errors. Therefore, we had to exclude all observations for which the
standard error was missing (studies from: Rosa_et_al_2014;
Campbell_et_al_2020; Blaxter 1960; Hokanson & Koenst 1986;
Brown_1969; Edsall_and_Colby_1970; Reynolds_1976;
Threader_and_Houston_1983). These observations were all LT50
measurements, for which sample standard deviation does not make
particular sense because the measure of dispersion (standard error) is
inferred from regression-based interpolations. 
 There was also one effect size (es_ID 1009 from Re_et_al_2006) for
which the standard deviation was taken as “0”. This value was replaced
as “NA”. 
       # Identify different species IDs used from the same species  
   d.raw $ species_ID[d.raw $ genus_species  ==   "Ischnura elegans" ]    
  ##  [1] "15" "15" "15" "15" "15" "15" "15" "15" "15" "15" "15" "16" "15" "15" "15"
## [16] "15"  
      d.raw $ species_ID[d.raw $ genus_species  ==   "Litopenaeus vannamei" ]    
  ##  [1] "79" "79" "79" "79" "79" "79" "79" "79" "79" "79" "79" "79" "50" "50" "50"
## [16] "50" "50" "50"  
       # Identify different species with the same species ID  
   d.raw $ genus_species[d.raw $ species_ID  ==   "109" ]    
  ## [1] "Myxocyprinus asiaticus" "Lampsilis abrupta"      "Lampsilis abrupta"     
## [4] "Lampsilis abrupta"  
      d.raw $ genus_species[d.raw $ species_ID  ==   "110" ]    
  ## [1] "Procypris rabaudi" "Lampsilis radiata"  
      d.raw $ genus_species[d.raw $ species_ID  ==   "111" ]    
  ## [1] "Lampsilis cariosa"            "Lampsilis cariosa"           
## [3] "Schizopygopsis younghusbandi" "Schizopygopsis younghusbandi"
## [5] "Schizopygopsis younghusbandi" "Schizopygopsis younghusbandi"  
       # Correct mistakes  
   d.raw $ species_ID[d.raw $ genus_species  ==   "Ischnura elegans" ]  <-   "15"    # Same species_ID for this species  
   d.raw $ species_ID[d.raw $ genus_species  ==   "Litopenaeus vannamei" ]  <-   "50"    # Same species_ID for this species  
    
   d.raw $ species_ID[d.raw $ genus_species  ==   "Lampsilis abrupta" ]  <-   "148"    # Use number not used previously  
   d.raw $ species_ID[d.raw $ genus_species  ==   "Lampsilis radiata" ]  <-   "149"    # Use number not used previously  
   d.raw $ species_ID[d.raw $ genus_species  ==   "Lampsilis cariosa" ]  <-   "150"    # Use number not used previously  
    
    
    
    # Exclude the study from Buckley and Nufio (2014)  
   d.raw  <-   filter (d.raw, ref  !=   "Buckley_and_Nufio_2014" ) 
    
   d.raw $ sd_HT_high[d.raw $ sd_HT_high  ==   "0" ]  <-   NA    # Replace the null SD value by NA  
    
    # Identify effect sizes where the measure of dispersion is standard error, and  
    # sample size is missing  
   d.raw $ ref[d.raw $ error_type  ==   "se"   &   is.na (d.raw $ sd_HT_high)  ==   "TRUE" ]    
  ##  [1] "Rosa_et_al_2014"           "Campbell_et_al_2020"      
##  [3] "Blaxter_1960"              "Blaxter_1960"             
##  [5] "Blaxter_1960"              "Brown_1969"               
##  [7] "Brown_1969"                "Brown_1969"               
##  [9] "Brown_1969"                "Brown_1969"               
## [11] "Brown_1969"                "Brown_1969"               
## [13] "Brown_1969"                "Brown_1969"               
## [15] "Brown_1969"                "Brown_1969"               
## [17] "Brown_1969"                "Brown_1969"               
## [19] "Brown_1969"                "Brown_1969"               
## [21] "Edsall_and_Colby_1970"     "Edsall_and_Colby_1970"    
## [23] "Edsall_and_Colby_1970"     "Edsall_and_Colby_1970"    
## [25] "Hokanson_and_Koenst_1986"  "Hokanson_and_Koenst_1986" 
## [27] "Reynolds_1976"             "Reynolds_1976"            
## [29] "Threader_and_Houston_1983" "Threader_and_Houston_1983"
## [31] "Threader_and_Houston_1983" "Threader_and_Houston_1983"
## [33] "Threader_and_Houston_1983" "Threader_and_Houston_1983"
## [35] "Threader_and_Houston_1983" "Threader_and_Houston_1983"
## [37] "Threader_and_Houston_1983" "Threader_and_Houston_1983"
## [39] "Threader_and_Houston_1983" "Threader_and_Houston_1983"  
      d.raw  <-   mutate (d.raw,  exclude =   ifelse (error_type  ==   "se"   &   is.na (sd_HT_high)  ==  
        "TRUE"   |  error_type  ==   "se"   &   is.na (sd_HT_low)  ==   "TRUE" ,  "yes" ,  "no" ))   # If the standard error is missing for either the low or high acclimation temperature, indicate exclude = 'yes', otherwise 'no'  
    
   d.raw  <-   filter (d.raw, exclude  !=   "yes" )   # Exclude all observations where the standard error is missing     
 
 
  Calculate effect sizes  
 
 Impute missing standard deviations 
 Missing standard deviation was estimated using formula 13.1 (p. 199)
of the Handbook of Meta-anlysis in Ecology and Evolution (Koricheva,
Gurevitch and Mengersen, 2013) 
 Because treatments are repeated in stepwise multiple comparisons
(i.e., the same data is used twice to calculate different effect sizes),
we made sure the imputed standard deviations (or standard error) were
the same for the repeated data. 
 First, only keep data with SD (because we can only impute SD), then
do the within/between study means, then impute. 
       # First, only take observations with standard deviations because they are the only ones we can impute  
   d.sd <-  filter (d.raw, error_type ==  "sd" ) 
    
    # Check assumption that SD/mean ratio is relatively constant between studies  
    
   d.sd   %>%    group_by (study_ID)  %>%          # Group each study together  
               mutate ( within_study_mean_low =   mean (mean_HT_low,  na.rm =  T),  # Calculate the within study heat tolerance at the lowest acclimation temperature  
                      within_study_mean_high =   mean (mean_HT_high,  na.rm =  T),   # Calculate the within study heat tolerance at the highest acclimation temperature  
                                             
                      within_study_sd_low =   mean (sd_HT_low,  na.rm= T),  # Calculate the within study standard deviation at the lowest acclimation temperature  
                      within_study_sd_high  =  mean (sd_HT_high,  na.rm= T),  # Calculate the within study standard deviation at the lowest acclimation temperature  
      
                      sd_mean_ratio_low =  within_study_sd_low  /  within_study_mean_low,  # Calculate the mean to SD ratio for lowest acclimation temperatures  
                      sd_mean_ratio_high =  within_study_sd_high  /  within_study_mean_high)  %>%    # Calculate the mean to SD ratio for highest acclimation temperatures  
                      ggplot ()  +   geom_histogram ( aes ( x= sd_mean_ratio_low),  fill=  "red" ,  alpha=  0.2 )  +   # Plot the results  
                                 geom_histogram ( aes ( x= sd_mean_ratio_high),  fill=  "blue" ,  alpha=  0.2 )    
   
       # Indicate which treatment is re-used in >1 comparisons  
   d.trt  <-   group_by (d.sd,shared_trt_ID)  %>%   summarise ( n_trt=  n ()) 
   d.sd  <-  left_join (d.sd, d.trt,  by=  "shared_trt_ID" ) 
    
    # Indicate which cohort is repeated >1 times to calculate sampling variances of non-independent observations differently.   
   d.cohort  <-   group_by (d.sd,cohort_ID)  %>%   summarise ( n_cohort=  n ()) 
   d.sd <-  left_join (d.sd, d.cohort,  by=  "cohort_ID" ) 
    
    
    # Calculate within and between study heat tolerance and standard deviation  
   d.sd   <-  d.sd  %>%   
                    group_by (study_ID)  %>%    # Group by study   
                                      mutate ( within_study_mean_low =   mean (mean_HT_low,  na.rm =  T),  # Calculate within study heat tolerance at the lowest acclimation temperature  
                                             within_study_mean_high =   mean (mean_HT_high,  na.rm =  T),  # Calculate within study heat tolerance at the highest acclimation temperature  
                                             
                                             within_study_sd_low =   mean (sd_HT_low,  na.rm= T),   # Calculate within study standard deviation at the lowest acclimation temperature  
                                             within_study_sd_high =   mean (sd_HT_high,  na.rm= T))  %>%   # Calculate within study standard deviation at the highest acclimation temperature  
                                             ungroup ()  %>%    # Ungroup observations to calculate between study heat tolerance and SD  
                                      mutate ( 
                                             
                                             between_study_mean_low=  mean (within_study_mean_low,  na.rm= T),   # Calculate between study heat tolerance at the lowest acclimation temperature  
                                             between_study_mean_high=  mean (within_study_mean_high,  na.rm= T),  # Calculate between study heat tolerance at the highest acclimation temperature  
                                             
                                             between_study_sd_low=  mean (within_study_sd_low,  na.rm= T),  # Calculate between study standard deviation at the lowest acclimation temperature  
                                             between_study_sd_high=  mean (within_study_sd_high,  na.rm= T)  # Calculate between study standard deviation at the highest acclimation temperature  
                                            )  
    
    
   d.sd $ sd_HT_high[ is.na (d.sd $ sd_HT_high)] <-  "missing"   # Problem detecting NA so replaced NA with "missing"  
   d.sd $ sd_HT_low[ is.na (d.sd $ sd_HT_low)] <-  "missing"  
      
   d.sd  <-  d.sd  %>%    mutate ( imputed=  ifelse (sd_HT_low ==  "missing"  | sd_HT_high ==  "missing" ,  "yes" ,  "no" ))  # Add a column `imputed`, indicated whether or not the standard deviation is missing and need to be imputed  
    
    
    # Impute missing standard deviations  
   d.sd  <-   mutate (d.sd,  
                          imputed_sd_low =   ifelse (sd_HT_low ==  "missing"  & n_trt !=  "1" ,  
                                                (within_study_mean_high * (between_study_sd_high / between_study_mean_high)),  
                                           ifelse (sd_HT_low ==  "missing"  & n_trt ==  "1" ,  
                                                (within_study_mean_low * (between_study_sd_low / between_study_mean_low)), sd_HT_low)),   # If the treatments are repeated, take the sd of the high acclimation group (because we made stepwise comparisons), otherwise estimate sd normally.   
                 
                          imputed_sd_high =   ifelse (sd_HT_high ==  "missing" ,  
                                                 (within_study_mean_high * (between_study_sd_high / between_study_mean_high)), sd_HT_high)) 
    
   d.sd $ imputed_sd_low  =   as.numeric (d.sd $ imputed_sd_low)  # Force sd to be considered as numeric values  
   d.sd $ imputed_sd_high  =   as.numeric (d.sd $ imputed_sd_high)  # Force sd to be considered as numeric values  
   d.sd $ sd_HT_low  =   as.numeric (d.sd $ sd_HT_low)  # Force sd to be considered as numeric values  
   d.sd $ sd_HT_high  =   as.numeric (d.sd $ sd_HT_high)  # Force sd to be considered as numeric values  
     
    
   d.se <-  filter (d.raw, error_type ==  "se" )  # Filter the data where the measure of dispersion is SE  
   d.se $ imputed <-   "no"   # Assign the value "no" for the column "imputed"  
    
    # Indicate which treatment is re-used in >1 comparisons  
   d.trt.se  <-   group_by (d.se,shared_trt_ID)  %>%   summarise ( n_trt=  n ()) 
   d.se  <-  left_join (d.se, d.trt.se,  by=  "shared_trt_ID" )  # Join this information to d.se  
    
    # Indicate which cohort is repeated >1 times to calculate sampling variances of non-independent observations differently.   
   d.cohort.se  <-   group_by (d.se,cohort_ID)  %>%   summarise ( n_cohort=  n ()) 
   d.se <-  left_join (d.se, d.cohort.se,  by=  "cohort_ID" )  # Join this information to d.se  
    
   d <-  full_join (d.sd, d.se)  # Join the data with imputed standard deviations  
    
    
   d $ es_ID =  as.numeric (d $ es_ID)  # Convert effect size ID to numeric to order the data  
   d <-  arrange (d, es_ID)  # Order the data by effect size ID  
   d $ es_ID =  as.factor (d $ es_ID)  # Convert effect size ID back to a factor  
    
    
    
    # General diagnostic plots   
    ggplot (d,  aes ( x= study_ID,  y=  log10 (imputed_sd_low),  col= imputed,  shape= metric),  show.legend= F) +  geom_point ( size=  2 ,  alpha=  0.5 )    
   
       ggplot (d,  aes ( x= study_ID,  y=  log10 (imputed_sd_high),  col= imputed,  shape= metric),  show.legend= F) +  geom_point ( size=  2 ,  alpha=  0.5 )    
   
 
 
 Calculate effect sizes and sampling variances 
       # Calculate effect sizes (formulas are presented in the manuscript)  
   d  <-  d  %>%    mutate ( dARR =  ((mean_HT_high - mean_HT_low) / (acc_temp_high - acc_temp_low)), 
                       
                       Var_dARR =   ifelse (error_type ==  "sd"  & n_cohort ==  "1" ,  
                                       (( 1  / (acc_temp_high  -  acc_temp_low)) ^  2  * (imputed_sd_low ^  2  / n_HT_low + imputed_sd_high ^  2  / n_HT_high)),  # If SD and independent, use this formula   
                                  ifelse (error_type ==  "sd"  & n_cohort !=  "1" ,  # If SD and non-independent, use this formula  
                                       (( 1  / (acc_temp_high  -  acc_temp_low)) ^  2  * ((imputed_sd_low ^  2   +  imputed_sd_high ^  2   -   2  *  0.5  * imputed_sd_low * imputed_sd_high) / (n_HT_low  +  n_HT_high))),  
                             
                                  ifelse (error_type ==  "se"  & n_cohort ==  "1" ,  # If SE and independent, use this formula  
                                       (( 1  / (acc_temp_high  -  acc_temp_low)) ^  2  * (sd_HT_low ^  2  + sd_HT_high ^  2 )), 
                            
                                       (( 1  / (acc_temp_high  -  acc_temp_low)) ^  2  * (sd_HT_low ^  2  +  sd_HT_high ^  2   -   2  *  0.5  * sd_HT_low * sd_HT_high))))),  # Otherwise (if SE and non-independent, use this last formula)  
             
                       precision = ( 1  /  sqrt (Var_dARR)))  # Calculate precision (1/SE)  
    
    summary (d $ dARR)  # Summary of the effect sizes     
  ##     Min.  1st Qu.   Median     Mean  3rd Qu.     Max. 
## -2.41514  0.02227  0.14704  0.16981  0.30649  2.70000  
       summary (d $ Var_dARR)  # Summary of the sampling variance of dARR; seems fine except for the maximum     
  ##     Min.  1st Qu.   Median     Mean  3rd Qu.     Max. 
##  0.00001  0.00074  0.00319  0.17200  0.01446 34.73580  
       summary (d $ precision)  # Summary of the precision; seems fine except for the maximum     
  ##     Min.  1st Qu.   Median     Mean  3rd Qu.     Max. 
##   0.1697   8.3163  17.7139  29.0091  36.7072 398.5101  
       ggplot (d,  aes ( x= study_ID,  y=  log10 (Var_dARR),  col= imputed,  shape= metric),  show.legend= F) +  geom_point ( size=  2 ,  alpha=  0.5 )     
   
       ggplot (d,  aes ( x= study_ID,  y= precision,  col= imputed,  shape= metric),  show.legend= F) +  geom_point ( size=  2 ,  alpha=  0.5 )    
   
 
 
 Merge designs E and F into the same design and rename design G 
 Originally, the design presented as design E in Figure 2 of the
manuscript was separated into two designs “E” and “F”, with the current
design “F” being classified as “G”. The difference between the former
designs “E” and “F” was on the life stage tested (i.e., former design
“E” only comprised data from animals tested at the juveniles stage,
whereas former design “F” only comprised data from animals tested right
after the adult transition; although both designs shared similar
acclimation periods) 
 Because designs E and F are very similar, and the sample size for
design F was very low (2 studies), we decided to merge these designs.
Design G was renamed to design “F”. 
      d $ exp_design  <-   str_replace (d $ exp_design,  "F" ,  "E" )   # Merge designs 'E' and 'F'  
   d $ exp_design  <-   str_replace (d $ exp_design,  "G" ,  "F" )   # Rename 'G' as 'F'     
 
 
 
  Data exploration  
 
 Check for extreme values and/or studies 
 Here, we checked the mean, minimum, and maximum values of dARR and
Var_dARR for each study to see if the extreme values are particularly
condensed in certain studies. 
 We also plotted the distribution of dARR to see if the response
variable is skewed. 
       kable (d  %>%  
        group_by (ref)  %>%   # Group by study  
        summarise ( mean_dARR =   mean (dARR),  
                  var_dARR =   mean (Var_dARR),  
                  prec =   mean (precision),  
                  max_dARR =   max (dARR),  
                  min_dARR =   min (dARR),  
                  n =   n ()))  %>%   # Calculate descriptive statistics  
        kable_styling ( "striped" ,  position =   "left" )  %>%   # Stylize the table  
        scroll_box ( width =   "100%" ,  height =   "500px" )    
 
 
 
 
 
ref
 
 
mean_dARR
 
 
var_dARR
 
 
prec
 
 
max_dARR
 
 
min_dARR
 
 
n
 
 
 
 
 
 
Abayarathna_et_al_2019
 
 
0.0422826
 
 
0.0001814
 
 
75.3091248
 
 
0.0540130
 
 
0.0115391
 
 
5
 
 
 
 
Akhtar_et_al_2013
 
 
0.4549679
 
 
0.0084695
 
 
13.0618648
 
 
0.6982367
 
 
0.2525533
 
 
8
 
 
 
 
Alford_2010
 
 
0.0644712
 
 
0.0001677
 
 
102.4163159
 
 
0.1886680
 
 
-0.0404940
 
 
36
 
 
 
 
Alford_et_al_2012
 
 
0.0194444
 
 
0.0005000
 
 
53.0330086
 
 
0.1200000
 
 
-0.0300000
 
 
18
 
 
 
 
Archambault_et_al_2014a
 
 
0.2500000
 
 
0.0312370
 
 
5.8724124
 
 
0.2800000
 
 
0.2200000
 
 
2
 
 
 
 
Archambault_et_al_2014b
 
 
0.0485714
 
 
0.0153061
 
 
8.3890929
 
 
0.5400000
 
 
-0.2600000
 
 
7
 
 
 
 
Ashaf-Ud-Doulah_et_al_2020
 
 
-0.3333333
 
 
0.0061033
 
 
12.8904219
 
 
-0.1666667
 
 
-0.5000000
 
 
2
 
 
 
 
Asheim_et_al_2020
 
 
0.2452538
 
 
0.0000084
 
 
352.9905278
 
 
0.3515975
 
 
0.1389100
 
 
2
 
 
 
 
Azra_et_al_2018
 
 
0.3176913
 
 
0.0048178
 
 
14.4070170
 
 
0.4858800
 
 
0.2616275
 
 
4
 
 
 
 
Bai_et_al_2019
 
 
-0.0979187
 
 
0.0047536
 
 
14.9844055
 
 
0.0706650
 
 
-0.3286250
 
 
4
 
 
 
 
Baroudy_and_Elliott_1994
 
 
0.0764341
 
 
0.0056489
 
 
21.4770868
 
 
0.4680000
 
 
-0.3000000
 
 
43
 
 
 
 
Becker_and_Genoway_1979
 
 
0.2663292
 
 
0.0001102
 
 
102.4939799
 
 
0.3810130
 
 
0.1873420
 
 
10
 
 
 
 
Benedict_et_al_1991
 
 
0.1135920
 
 
0.0004995
 
 
44.7442857
 
 
0.1135920
 
 
0.1135920
 
 
1
 
 
 
 
Billman_et_al_2008
 
 
0.4135680
 
 
2.7801338
 
 
13.6085427
 
 
0.8333333
 
 
-0.0072440
 
 
9
 
 
 
 
Bishai_1960
 
 
0.2787259
 
 
0.0350696
 
 
6.7536342
 
 
0.7180689
 
 
0.0773267
 
 
6
 
 
 
 
Blair_and_Glover_2019
 
 
-0.0547942
 
 
0.0003733
 
 
51.7583539
 
 
-0.0547942
 
 
-0.0547942
 
 
1
 
 
 
 
Bowden_et_al_2018
 
 
0.3560112
 
 
0.0292445
 
 
5.8630533
 
 
0.4881225
 
 
0.1866100
 
 
4
 
 
 
 
Britton_2005
 
 
0.0697917
 
 
0.0005755
 
 
52.3497327
 
 
0.5500000
 
 
-0.1666667
 
 
16
 
 
 
 
Bugg_et_al_2020
 
 
0.3592606
 
 
0.0025333
 
 
21.8863872
 
 
0.5742775
 
 
0.1861925
 
 
4
 
 
 
 
Cai_and_Chen_2005
 
 
0.0455578
 
 
0.0169639
 
 
11.8866227
 
 
0.2866667
 
 
-0.7535900
 
 
18
 
 
 
 
Carbonell_and_Stoks_2020
 
 
0.1721478
 
 
0.0087696
 
 
13.4077909
 
 
0.4118775
 
 
0.0095800
 
 
9
 
 
 
 
Chaterjee_et_al_2004
 
 
0.2650000
 
 
0.0005862
 
 
46.5140866
 
 
0.4600000
 
 
0.1200000
 
 
8
 
 
 
 
Chen_and_Chen_1999
 
 
0.1843035
 
 
0.0023786
 
 
20.6360961
 
 
0.5400000
 
 
0.0489440
 
 
12
 
 
 
 
Chen_et_al_2013
 
 
-0.2500000
 
 
0.0039815
 
 
55.9404777
 
 
0.0750000
 
 
-0.5500000
 
 
22
 
 
 
 
Cheung_2019
 
 
-0.1015827
 
 
0.0308685
 
 
9.2756558
 
 
0.6157962
 
 
-0.7636286
 
 
43
 
 
 
 
Chidawanyika_and_Terblanche_2010
 
 
0.1146790
 
 
0.0002147
 
 
68.2552997
 
 
0.2293580
 
 
0.0000000
 
 
2
 
 
 
 
Cook_et_al_2006
 
 
0.3565646
 
 
0.0003226
 
 
68.8543839
 
 
0.5600000
 
 
0.1166667
 
 
7
 
 
 
 
Currie_et_al_1998
 
 
0.2933333
 
 
0.0012290
 
 
30.7886049
 
 
0.4600000
 
 
0.1400000
 
 
6
 
 
 
 
Dang_et_al_2019
 
 
0.0853788
 
 
0.0003908
 
 
51.3033640
 
 
0.1097410
 
 
0.0658450
 
 
4
 
 
 
 
Das_et_al_2004
 
 
0.2032407
 
 
0.0082969
 
 
28.8623767
 
 
0.6300000
 
 
0.0333333
 
 
18
 
 
 
 
Das_et_al_2005
 
 
0.2831111
 
 
0.0010057
 
 
38.9740108
 
 
0.4960000
 
 
0.0833333
 
 
3
 
 
 
 
Dayanada_et_al_2017
 
 
-0.3985789
 
 
0.0037211
 
 
16.6430034
 
 
-0.3851684
 
 
-0.4119895
 
 
2
 
 
 
 
de-Beek_et_al_2017
 
 
0.0826088
 
 
0.0023948
 
 
20.4422841
 
 
0.0852850
 
 
0.0799325
 
 
2
 
 
 
 
de-Beek_et_al_2018a
 
 
0.0698550
 
 
0.0017264
 
 
24.0675422
 
 
0.0698550
 
 
0.0698550
 
 
1
 
 
 
 
de-Beek_et_al_2018b
 
 
0.0992363
 
 
0.0020452
 
 
22.1610679
 
 
0.1547075
 
 
0.0447825
 
 
4
 
 
 
 
Debnath_et_al_2006
 
 
0.1843750
 
 
0.0003500
 
 
87.9325151
 
 
0.3500000
 
 
0.0450000
 
 
4
 
 
 
 
Del-Rio_et_al_2019
 
 
-0.0168266
 
 
0.0045805
 
 
19.0698223
 
 
0.2670950
 
 
-0.5235050
 
 
8
 
 
 
 
Delorme-Juri_2017
 
 
0.1444700
 
 
15.4177909
 
 
1.3235837
 
 
1.1854700
 
 
-0.8413000
 
 
6
 
 
 
 
Deslauriers_et_al_2016
 
 
0.2104328
 
 
0.0043057
 
 
19.4854456
 
 
0.2791660
 
 
0.1495499
 
 
3
 
 
 
 
Diaz-Herrera_et_al_1998
 
 
0.4375000
 
 
0.0205716
 
 
11.5153366
 
 
0.7666667
 
 
0.1666667
 
 
8
 
 
 
 
Donelson_et_al_2015
 
 
0.0592972
 
 
0.0014255
 
 
26.6957898
 
 
0.2898975
 
 
-0.1713031
 
 
2
 
 
 
 
Dulger_et_al_2012
 
 
0.2710000
 
 
0.0011877
 
 
30.1827669
 
 
0.3220000
 
 
0.2200000
 
 
2
 
 
 
 
Ebel_et_al_1971
 
 
-0.0357366
 
 
3.3237193
 
 
0.6891243
 
 
0.7739900
 
 
-0.7083880
 
 
10
 
 
 
 
Elliott_1991
 
 
0.1255392
 
 
0.0201177
 
 
9.6820608
 
 
0.5781991
 
 
-0.5781990
 
 
80
 
 
 
 
Elliott_and_Klemetsen_2002
 
 
0.1438095
 
 
0.0028514
 
 
22.9464408
 
 
0.3479953
 
 
0.0253548
 
 
8
 
 
 
 
Esquer-Mendez_et_al_2010
 
 
0.1041667
 
 
0.0126217
 
 
20.9382859
 
 
0.3125000
 
 
0.0000000
 
 
3
 
 
 
 
Faleiro_et_al_2016
 
 
0.4285714
 
 
0.6356765
 
 
1.2542437
 
 
0.4285714
 
 
0.4285714
 
 
1
 
 
 
 
Fields_et_al_1987
 
 
0.4760417
 
 
0.0014669
 
 
28.9182905
 
 
0.5750000
 
 
0.3625000
 
 
12
 
 
 
 
Floyd_1983
 
 
0.1980737
 
 
0.0007774
 
 
63.7249643
 
 
0.3875354
 
 
-0.1314448
 
 
20
 
 
 
 
Floyd_1985
 
 
0.1647468
 
 
0.0005790
 
 
90.0607240
 
 
0.2375000
 
 
0.0692641
 
 
13
 
 
 
 
Gervais_et_al_2020
 
 
0.5077778
 
 
0.0038111
 
 
17.3971720
 
 
0.6466667
 
 
0.3700000
 
 
3
 
 
 
 
Gibson_et_al_2015
 
 
-0.0908345
 
 
0.0335600
 
 
14.3738981
 
 
0.0412700
 
 
-0.2187500
 
 
4
 
 
 
 
Gomez-Isaza_et_al_2020
 
 
0.2348710
 
 
0.0010724
 
 
30.5372505
 
 
0.2348710
 
 
0.2348710
 
 
1
 
 
 
 
Gray_2013
 
 
0.0386719
 
 
0.0005119
 
 
44.4515464
 
 
0.0719863
 
 
0.0053575
 
 
2
 
 
 
 
Gunderson_et_al_2020
 
 
0.0357143
 
 
0.0842502
 
 
3.6202384
 
 
0.6428571
 
 
-0.5000000
 
 
4
 
 
 
 
He_et_al_2014
 
 
0.1681250
 
 
0.0000594
 
 
154.0989112
 
 
0.2550000
 
 
0.0500000
 
 
4
 
 
 
 
Healy_et_al_2019
 
 
0.1048000
 
 
0.0009741
 
 
33.9768257
 
 
0.2412500
 
 
-0.0425000
 
 
10
 
 
 
 
Illing_et_al_2020
 
 
0.2311021
 
 
0.1328116
 
 
7.4400875
 
 
1.6074000
 
 
-0.3640778
 
 
27
 
 
 
 
Ineno_et_al_2005
 
 
0.0902312
 
 
0.0208194
 
 
42.7221821
 
 
1.0714375
 
 
-1.1207650
 
 
13
 
 
 
 
Kelly_et_al_2014
 
 
0.2495770
 
 
0.0041549
 
 
18.0829434
 
 
0.5074299
 
 
0.0269977
 
 
12
 
 
 
 
Kerfoot_2012
 
 
0.0000000
 
 
0.0147221
 
 
8.2416799
 
 
0.1600000
 
 
-0.1600000
 
 
2
 
 
 
 
Kern_et_al_2015
 
 
0.2465955
 
 
0.0003993
 
 
50.0752545
 
 
0.2817210
 
 
0.2114700
 
 
2
 
 
 
 
Kingsolver_et_al_2016
 
 
0.2283242
 
 
0.0193378
 
 
9.4239779
 
 
0.4798000
 
 
-0.2197000
 
 
4
 
 
 
 
Kir_2020
 
 
0.2560000
 
 
0.0123074
 
 
18.4841046
 
 
0.7460000
 
 
0.0720000
 
 
6
 
 
 
 
Kir_and_Demirci_2018
 
 
0.3096667
 
 
0.0120644
 
 
18.6234218
 
 
0.5780000
 
 
0.1800000
 
 
6
 
 
 
 
Kir_et_al_2017
 
 
0.2300000
 
 
0.0007922
 
 
48.2226547
 
 
0.2675000
 
 
0.1650000
 
 
3
 
 
 
 
Komoroske_et_al_2014
 
 
0.2434211
 
 
0.0022442
 
 
21.3182700
 
 
0.7368421
 
 
-0.2500000
 
 
2
 
 
 
 
Kumlu_et_al_2010
 
 
0.4203333
 
 
0.0015146
 
 
34.5051064
 
 
0.5420000
 
 
0.3300000
 
 
6
 
 
 
 
Larios-Soriano_et_al_2019
 
 
0.3785242
 
 
0.0032903
 
 
18.4470864
 
 
0.4742933
 
 
0.3101167
 
 
4
 
 
 
 
Leon-Palomino_et_al_2017
 
 
0.4899107
 
 
0.0121333
 
 
11.3917243
 
 
1.0609733
 
 
-0.0811520
 
 
2
 
 
 
 
Li_et_al_2015
 
 
0.1781333
 
 
0.0087457
 
 
15.3922944
 
 
0.4950000
 
 
-0.0780000
 
 
5
 
 
 
 
Linton_et_al_1998
 
 
0.2111598
 
 
0.0144344
 
 
8.7693821
 
 
0.2482579
 
 
0.1572316
 
 
3
 
 
 
 
Llewelyn_et_al_2018
 
 
0.0177900
 
 
0.0003837
 
 
54.0875299
 
 
0.0753533
 
 
-0.0258900
 
 
4
 
 
 
 
Lohr_et_al_1996
 
 
0.1847002
 
 
0.0102181
 
 
21.6696135
 
 
0.2763158
 
 
0.0963850
 
 
4
 
 
 
 
Lu_et_al_2020
 
 
0.3100000
 
 
0.1027122
 
 
3.1202464
 
 
0.3100000
 
 
0.3100000
 
 
1
 
 
 
 
Ma_and_Ma_2012
 
 
0.0677063
 
 
0.0013982
 
 
27.0361051
 
 
0.1603567
 
 
-0.0249440
 
 
2
 
 
 
 
Manriquez_et_al_2019
 
 
0.1229922
 
 
0.0169003
 
 
13.9849521
 
 
0.1976285
 
 
0.0483559
 
 
2
 
 
 
 
Mascaro_et_al_2016
 
 
0.6416667
 
 
0.0004225
 
 
48.6530912
 
 
0.6416667
 
 
0.6416667
 
 
1
 
 
 
 
Mascaro_et_al_2017
 
 
0.3916667
 
 
0.0015799
 
 
25.1588361
 
 
0.3916667
 
 
0.3916667
 
 
1
 
 
 
 
Mascaro_et_al_2018
 
 
0.2583333
 
 
0.0009954
 
 
31.6962326
 
 
0.2583333
 
 
0.2583333
 
 
1
 
 
 
 
McCauley_1963
 
 
0.0310620
 
 
0.0246195
 
 
6.3732458
 
 
0.0310620
 
 
0.0310620
 
 
1
 
 
 
 
McDermid_et_al_2013
 
 
0.3145695
 
 
0.0004557
 
 
47.2930566
 
 
0.3791380
 
 
0.2781460
 
 
4
 
 
 
 
McDonnell_et_al_2019
 
 
0.3054676
 
 
0.0005401
 
 
43.0861315
 
 
0.4515736
 
 
-0.0977970
 
 
4
 
 
 
 
Medina-Romo_et_al_2018
 
 
0.3580833
 
 
0.0171857
 
 
7.6280912
 
 
0.6319133
 
 
0.1053167
 
 
4
 
 
 
 
Meng_et_al_2009
 
 
0.0900000
 
 
0.0006508
 
 
39.2000063
 
 
0.0900000
 
 
0.0900000
 
 
1
 
 
 
 
Mitchell_et_al_1993
 
 
0.1072580
 
 
0.0002899
 
 
61.8369460
 
 
0.3322580
 
 
-0.1935480
 
 
4
 
 
 
 
Moyano_et_al_2017
 
 
0.5984217
 
 
0.0404443
 
 
6.6869073
 
 
1.1739923
 
 
0.2091346
 
 
7
 
 
 
 
Mueller_et_al_2019
 
 
0.0086520
 
 
0.0102290
 
 
10.8981737
 
 
0.3987168
 
 
-0.2554760
 
 
6
 
 
 
 
Munoz_et_al_2018
 
 
0.2504176
 
 
0.0019224
 
 
24.2865463
 
 
0.3494455
 
 
0.0910614
 
 
4
 
 
 
 
Mutamiswa_et_al_2018a
 
 
0.0234410
 
 
0.0011717
 
 
29.2183745
 
 
0.1253600
 
 
-0.0445360
 
 
6
 
 
 
 
Mutamiswa_et_al_2018b
 
 
0.0499230
 
 
0.0010794
 
 
30.4388650
 
 
0.1248080
 
 
-0.0249620
 
 
2
 
 
 
 
Myrick_1998
 
 
0.1443156
 
 
0.1622640
 
 
17.8326161
 
 
0.2692308
 
 
0.0126697
 
 
8
 
 
 
 
Myrick_and_Cech_2000
 
 
0.1333333
 
 
0.0093889
 
 
10.3203137
 
 
0.1333333
 
 
0.1333333
 
 
1
 
 
 
 
Myrick_and_Cech_2002
 
 
0.3333333
 
 
0.0080222
 
 
11.1648439
 
 
0.3333333
 
 
0.3333333
 
 
1
 
 
 
 
Myrick_and_Cech_2003
 
 
0.2175000
 
 
0.0067300
 
 
14.8718114
 
 
0.2666667
 
 
0.1750000
 
 
6
 
 
 
 
Myrick_and_Cech_2005
 
 
0.2632875
 
 
0.0114797
 
 
9.5079355
 
 
0.3001975
 
 
0.2263775
 
 
2
 
 
 
 
Noyola_et_al_2013
 
 
0.3916667
 
 
0.0224516
 
 
7.1393644
 
 
0.5250000
 
 
0.2250000
 
 
3
 
 
 
 
Opuszynski_et_al_1989
 
 
0.3451461
 
 
0.0327028
 
 
5.5582393
 
 
0.5683000
 
 
0.1900767
 
 
3
 
 
 
 
Orille_et_al_2020
 
 
0.2517306
 
 
0.0003014
 
 
57.6012050
 
 
0.2517306
 
 
0.2517306
 
 
1
 
 
 
 
Oyamaguchi_et_al_2018
 
 
0.2316729
 
 
0.0229617
 
 
10.1583883
 
 
0.4608950
 
 
0.0942571
 
 
3
 
 
 
 
Pandolfo_et_al_2010a
 
 
-0.1628571
 
 
0.2628369
 
 
2.3280499
 
 
0.5200000
 
 
-1.1400000
 
 
7
 
 
 
 
Pandolfo_et_al_2010b
 
 
-0.6866667
 
 
3.0780230
 
 
5.7405113
 
 
-0.1400000
 
 
-1.5000000
 
 
3
 
 
 
 
Peng_et_al_2014
 
 
0.5122810
 
 
0.0012702
 
 
28.2565280
 
 
0.6363620
 
 
0.2747940
 
 
4
 
 
 
 
Pereira_et_al_2017
 
 
0.1857273
 
 
0.0009366
 
 
34.1994500
 
 
0.2682000
 
 
0.0792000
 
 
11
 
 
 
 
Perez_et_al_2003
 
 
0.4750000
 
 
0.0075652
 
 
11.4971338
 
 
0.4750000
 
 
0.4750000
 
 
1
 
 
 
 
Perez_et_al_2004
 
 
0.6750000
 
 
0.0013015
 
 
27.7185858
 
 
0.6750000
 
 
0.6750000
 
 
1
 
 
 
 
Perez_et_al_2006
 
 
0.0500000
 
 
0.0061823
 
 
12.7181594
 
 
0.0500000
 
 
0.0500000
 
 
1
 
 
 
 
Perez_et_al_2007
 
 
0.1750000
 
 
0.0005694
 
 
41.9065627
 
 
0.1750000
 
 
0.1750000
 
 
1
 
 
 
 
Perez_et_al_2010
 
 
0.7500000
 
 
0.0010575
 
 
30.7510100
 
 
0.7500000
 
 
0.7500000
 
 
1
 
 
 
 
Perez_et_al_2012
 
 
0.0500000
 
 
0.0013015
 
 
27.7185858
 
 
0.0500000
 
 
0.0500000
 
 
1
 
 
 
 
Pimentel_et_al_2014
 
 
0.3153800
 
 
0.0027002
 
 
20.3116342
 
 
0.4723500
 
 
0.1497700
 
 
4
 
 
 
 
Piyaphongkul_et_al_2014
 
 
0.1970238
 
 
0.0211766
 
 
15.3007733
 
 
0.4714286
 
 
0.0125000
 
 
6
 
 
 
 
Piyaphongkul_et_al_2018
 
 
0.2050000
 
 
0.0008500
 
 
35.0419485
 
 
0.2400000
 
 
0.1700000
 
 
2
 
 
 
 
Porter_2016
 
 
0.2750000
 
 
0.1053125
 
 
3.0814849
 
 
0.2750000
 
 
0.2750000
 
 
1
 
 
 
 
Porter_2019
 
 
-0.0250000
 
 
0.0428125
 
 
4.8329767
 
 
-0.0250000
 
 
-0.0250000
 
 
1
 
 
 
 
Porter_2021
 
 
0.6500000
 
 
0.0365625
 
 
5.2297636
 
 
0.6500000
 
 
0.6500000
 
 
1
 
 
 
 
Procarione_and_King_1993
 
 
0.3980294
 
 
0.0102526
 
 
10.2374962
 
 
0.5555556
 
 
0.3076923
 
 
4
 
 
 
 
Re_et_al_2006
 
 
0.4767143
 
 
0.0240879
 
 
8.2254399
 
 
1.2200000
 
 
-0.0900000
 
 
35
 
 
 
 
Re_et_al_2012
 
 
0.4250972
 
 
0.0020867
 
 
22.9887521
 
 
0.7766800
 
 
0.1290733
 
 
12
 
 
 
 
Reyes_et_al_2011
 
 
0.2935600
 
 
0.0032982
 
 
17.5455199
 
 
0.3490233
 
 
0.1931133
 
 
4
 
 
 
 
Rodgers_et_al_2019
 
 
0.3800000
 
 
0.0039071
 
 
16.1261792
 
 
0.4614286
 
 
0.2985714
 
 
2
 
 
 
 
Rosa_et_al_2014
 
 
0.2467058
 
 
0.0344412
 
 
9.5418595
 
 
0.8278632
 
 
-0.0604048
 
 
7
 
 
 
 
Ruthsatz_et_al_2018
 
 
0.0333333
 
 
0.0152778
 
 
9.6199463
 
 
2.7000000
 
 
-1.4000000
 
 
4
 
 
 
 
Ruthsatz_et_al_2020
 
 
0.4927083
 
 
0.0081047
 
 
21.6126127
 
 
1.2266667
 
 
-0.3933333
 
 
4
 
 
 
 
Sakurai_et_al_2021
 
 
0.1557799
 
 
0.0005417
 
 
44.9398975
 
 
0.2081410
 
 
0.1034188
 
 
2
 
 
 
 
Salachan_and_Sorensen_2017
 
 
0.0662500
 
 
0.0014432
 
 
27.7374492
 
 
0.1926675
 
 
-0.0515000
 
 
4
 
 
 
 
Salinas_et_al_2019
 
 
0.6607434
 
 
0.0639706
 
 
11.8925715
 
 
1.0468267
 
 
0.3064920
 
 
3
 
 
 
 
Sasaki_and_Dam_2019
 
 
0.7621810
 
 
0.0251199
 
 
7.2983358
 
 
1.5226225
 
 
0.3175750
 
 
10
 
 
 
 
Sasaki_and_Dam_2020
 
 
0.3297967
 
 
0.0029592
 
 
19.7639473
 
 
0.5723633
 
 
-0.1087100
 
 
5
 
 
 
 
Sasaki_et_al_2019
 
 
0.1908150
 
 
0.0162258
 
 
8.0619543
 
 
0.3579550
 
 
0.0359850
 
 
4
 
 
 
 
Sasmita_et_al_2019
 
 
0.3458333
 
 
0.0895791
 
 
4.6069208
 
 
1.0600000
 
 
0.1066667
 
 
6
 
 
 
 
Schaefer_and_Ryan_2006
 
 
0.0748408
 
 
0.0027237
 
 
19.1827253
 
 
0.1110675
 
 
0.0322450
 
 
3
 
 
 
 
Shinner_et_al_2020
 
 
0.0058965
 
 
0.0003282
 
 
55.2042580
 
 
0.0168204
 
 
-0.0050275
 
 
2
 
 
 
 
Shrode_1975
 
 
0.0102783
 
 
0.0117458
 
 
9.2269509
 
 
0.1767225
 
 
-0.2011500
 
 
13
 
 
 
 
Simon_et_al_2015
 
 
0.1287012
 
 
0.0010855
 
 
44.2932737
 
 
0.2966670
 
 
0.0115390
 
 
15
 
 
 
 
Slotsbo_et_al_2016
 
 
0.0719253
 
 
0.0015568
 
 
53.4677447
 
 
0.1457140
 
 
-0.2067710
 
 
32
 
 
 
 
Spinks_et_al_2019
 
 
-0.0296650
 
 
0.0014100
 
 
29.8094881
 
 
0.0202000
 
 
-0.1284100
 
 
8
 
 
 
 
Stitt_2012
 
 
0.1727788
 
 
0.0008875
 
 
34.8665097
 
 
0.3130041
 
 
0.0692397
 
 
9
 
 
 
 
Stoler_2012
 
 
-0.1868946
 
 
0.0450393
 
 
10.5843418
 
 
0.7115385
 
 
-1.2500000
 
 
3
 
 
 
 
Tatum_2018
 
 
0.1928571
 
 
0.0063454
 
 
13.2625824
 
 
0.2285714
 
 
0.1500000
 
 
3
 
 
 
 
Terblanche_and_Chown_2006
 
 
-0.0028871
 
 
0.0014489
 
 
27.8831569
 
 
0.0279070
 
 
-0.0336812
 
 
2
 
 
 
 
Troia_et_al_2015
 
 
0.2790809
 
 
0.5391901
 
 
3.1993095
 
 
2.2348500
 
 
-0.7703100
 
 
17
 
 
 
 
Tsuchida_and_Setoguma_1997
 
 
0.2208561
 
 
0.0012408
 
 
42.0244152
 
 
0.3177400
 
 
0.1255080
 
 
9
 
 
 
 
Underwood_et_al_2012
 
 
0.1937075
 
 
0.0025186
 
 
23.1729833
 
 
0.3333333
 
 
0.1224490
 
 
5
 
 
 
 
Uriarte_et_al_2018
 
 
0.1914155
 
 
0.0148079
 
 
8.2177450
 
 
1.1422950
 
 
-2.4151450
 
 
22
 
 
 
 
Vanvelk_et_al_2021
 
 
0.1032731
 
 
0.0019630
 
 
23.1124143
 
 
0.2226817
 
 
-0.0636233
 
 
6
 
 
 
 
Wagner_et_al_2001
 
 
0.2261674
 
 
0.0006738
 
 
49.5270258
 
 
0.4090909
 
 
-0.0195682
 
 
11
 
 
 
 
Walsh_et_al_1998
 
 
0.2230000
 
 
0.0091460
 
 
10.4564533
 
 
0.2230000
 
 
0.2230000
 
 
1
 
 
 
 
Wang_2014
 
 
0.3933333
 
 
0.0032844
 
 
18.0464599
 
 
0.7200000
 
 
0.1800000
 
 
3
 
 
 
 
Wang_et_al_2013
 
 
0.3503160
 
 
0.0131409
 
 
10.1368526
 
 
0.4993660
 
 
0.2012660
 
 
2
 
 
 
 
Warriner_et_al_2020
 
 
0.2000000
 
 
0.0006444
 
 
39.3919299
 
 
0.2000000
 
 
0.2000000
 
 
1
 
 
 
 
White_and_Wahl_2020
 
 
0.6053272
 
 
0.0030467
 
 
20.0036686
 
 
0.6222767
 
 
0.5859567
 
 
3
 
 
 
 
Wong_and_Hofmann_2020
 
 
0.0618902
 
 
0.0012199
 
 
28.6378246
 
 
0.0750000
 
 
0.0487805
 
 
2
 
 
 
 
Wu_et_al_2013
 
 
0.1433880
 
 
0.0004693
 
 
46.2016834
 
 
0.2426450
 
 
0.0441310
 
 
2
 
 
 
 
Xu_et_al_2015
 
 
0.1631094
 
 
0.0024202
 
 
20.3312628
 
 
0.2073162
 
 
0.1189025
 
 
2
 
 
 
 
Xue_and_Ma_2020
 
 
0.1237757
 
 
0.0031886
 
 
19.1650647
 
 
0.3240480
 
 
-0.1172380
 
 
12
 
 
 
 
Yoon_et_al_2019
 
 
-0.1225325
 
 
0.0448945
 
 
6.1378861
 
 
0.1492600
 
 
-0.5357700
 
 
4
 
 
 
 
Zhang_and_Kieffer_2014
 
 
0.5200000
 
 
0.0078690
 
 
12.0230393
 
 
0.7800000
 
 
0.2600000
 
 
2
 
 
 
 
Zhou_et_al_2019
 
 
0.4148405
 
 
0.0023819
 
 
22.7363794
 
 
0.4392430
 
 
0.3904380
 
 
2
 
 
 
 
Zhu_et_al_2019
 
 
0.0790000
 
 
0.0001253
 
 
92.1683669
 
 
0.1480000
 
 
0.0340000
 
 
4
 
 
 
 
Ziegeweid_et_al_2008
 
 
0.2934783
 
 
0.0009170
 
 
45.0360055
 
 
0.3043478
 
 
0.2826087
 
 
2
 
 
 
 
 
      d  %>%  
        group_by (ref)  %>%  
        summarise ( mean_dARR =   mean (dARR),  
                  var_dARR =   mean (Var_dARR),  
                  prec =   mean (precision), 
                  max_dARR =   max (dARR), 
                  min_dARR =   min (dARR),  
                  n =   n ())  %>%   
        ggplot ()  +   
        stat_dots ( aes ( x= mean_dARR),  col=  "darkcyan" )  # Plot the results     
   
       ggplot (d,  aes (dARR))  +   
      geom_histogram ( fill =   "darkcyan" ,  col =   "black" ,  binwidth =   0.02 )  +  
      theme_classic ()  # Plot the distribution of dARR     
   
       ggplot (d)  +  
      stat_slab ( aes ( x= dARR,  fill_ramp=  stat ( cut_cdf_qi (cdf,  .width=  c (. 5 , . 8 , . 95 ),  labels= scales ::  percent_format ()))),  side=  "bottom" ,  scale=  0.5 ,  show.legend= F,  col=  "darkcyan" )  +  
      stat_dots ( aes ( x= dARR),  alpha=  0.8 ,  quantiles=  1000 ,  dotsize=  1.5 ,  shape=  16 ,  show.legend= F,  col=  "darkcyan" )  # More stylised version of the distribution     
   
 
 
 Missing data patterns 
 The package  DataExplorer  has some useful resources for
data exploration 
       plot_missing (d)   # Missing data patterns     
   
 
 
 Frequency of the different variables 
       plot_bar (d)   # Distribution of categorical variables     
      
       plot_histogram (d)   # Distribution of continuous variables     
     
 
 
 Map effect sizes against all
extracted moderators 
 
 Habitat type 
       # Plot habitat variation in dARR, with data points scaled by precision  
    ggplot ( data= d,  aes ( y= dARR,  x= habitat,  col= habitat, size= precision)) +  
                         geom_hline ( yintercept =   0 , linetype =   2 ,  colour =   "black" , alpha=  0.5 ) +   
                         geom_jitter ( alpha=  0.5 ) +   # display data points with a jitter, so they don't overlap  
                         scale_size_continuous ( range =   c ( 1 ,  7 )) +   # change scaling of data points  
                         labs ( x=  "Experimental design" , y=  "dARR" )  +   # rename axis labels  
                         theme_classic ()  # Classic theme     
   
       ggbetweenstats ( data= d,  y= dARR,  x= habitat)  # The ggbetweenstats function provides a neat visualisation of the data along with descriptive statistics     
   
       # Plot habitat variation in precision  
    ggplot ( data= d,  aes ( y= precision,  x= habitat,  col= habitat)) +  
                         geom_hline ( yintercept =   0 , linetype =   2 ,  colour =   "black" , alpha=  0.5 ) +   
                         geom_jitter ( alpha=  0.5 ) +   
                         scale_size_continuous ( range =   c ( 1 ,  7 )) +   
                         labs ( x=  "Experimental design" , y=  "Precision (1/SE)" )  +   
                         theme_classic ()     
   
       ggbetweenstats ( data= d,  y= precision,  x= habitat)     
   
 
 
 Taxonomic group 
       # Plot taxonomic variation in dARR, with data points scaled by precision  
    ggplot ( data =  d,  aes ( y =  dARR,  x =  taxonomic_group,  col =  taxonomic_group,  size =  precision))  +  
        geom_hline ( yintercept =   0 ,  linetype =   2 ,  colour =   "black" ,  alpha =   0.5 )  +   geom_jitter ( alpha =   0.5 )  +  
        scale_size_continuous ( range =   c ( 1 ,  7 ))  +   labs ( x =   "Experimental design" ,  y =   "dARR" )  +  
        theme_classic ()    
   
       ggbetweenstats ( data =  d,  y =  dARR,  x =  taxonomic_group)    
   
       # Plot taxonomic variation in precision, with data points scaled by precision  
    ggplot ( data =  d,  aes ( y =  precision,  x =  taxonomic_group,  col =  taxonomic_group))  +  
        geom_hline ( yintercept =   0 ,  linetype =   2 ,  colour =   "black" ,  alpha =   0.5 )  +   geom_jitter ( alpha =   0.5 )  +  
        scale_size_continuous ( range =   c ( 1 ,  7 ))  +   labs ( x =   "Experimental design" ,  y =   "Precision (1/SE)" )  +  
        theme_classic ()    
   
       ggbetweenstats ( data =  d,  y =  precision,  x =  taxonomic_group)    
   
 
 
 Time in a common garden condition 
       # Plot variation in dARR with re-acclimation time, with data points scaled by  
    # precision  
    ggplot ( data =  d,  aes ( y =  dARR,  x =  time_common_temp,  size =  precision),  alpha =   0.5 )  +  
        geom_hline ( yintercept =   0 ,  linetype =   2 ,  colour =   "black" ,  alpha =   0.5 )  +   geom_jitter ( alpha =   0.5 )  +  
        scale_size_continuous ( range =   c ( 1 ,  7 ))  +   labs ( x =   "age_tested" ,  y =   "dARR" )  +  
        theme_classic ()  +   geom_smooth ( method =   "lm" ) 
    
    ggscatterstats ( data =  d,  x =  time_common_temp,  y =  dARR)    
   
       # Plot variation in precision with re-acclimation time.  
    ggplot ( data =  d,  aes ( y =  precision,  x =  time_common_temp),  alpha =   0.5 )  +   geom_hline ( yintercept =   0 , 
        linetype =   2 ,  colour =   "black" ,  alpha =   0.5 )  +   geom_jitter ( alpha =   0.5 )  +   scale_size_continuous ( range =   c ( 1 , 
        7 ))  +   labs ( x =   "age_tested" ,  y =   "Precision (1/SE)" )  +   theme_classic ()  +   geom_smooth ( method =   "lm" ) 
    
    ggscatterstats ( data =  d,  x =  time_common_temp,  y =  precision)    
   
 
 
 Experimental design 
       # Plot variation in dARR between experimental designs (life history variation  
    # and persistence)  
   p_design  <-   ggplot ( data =  d,  aes ( y =  dARR,  x =  exp_design,  col =  exp_design,  size =  precision))  +  
        geom_hline ( yintercept =   0 ,  linetype =   2 ,  colour =   "black" ,  alpha =   0.5 )  +   geom_jitter ( alpha =   0.5 )  +  
        scale_size_continuous ( range =   c ( 1 ,  7 ))  +   labs ( x =   "Experimental design" ,  y =   "dARR" )  +  
        theme_classic () 
    
    # Plot variation in precision between experimental designs (life history  
    # variation and persistence)  
   p_design_var  <-   ggplot ( data =  d,  aes ( y =  precision,  x =  exp_design,  col =  exp_design))  +  
        geom_hline ( yintercept =   0 ,  linetype =   2 ,  colour =   "black" ,  alpha =   0.5 )  +   geom_jitter ( alpha =   0.5 )  +  
        scale_size_continuous ( range =   c ( 1 ,  7 ))  +   labs ( x =   "Experimental design" ,  y =   "Precision (1/SE)" )  +  
        theme_classic () 
    
    # Plot variation in dARR between experimental designs (life history variation  
    # and persistence), with points coloured for each taxonomic group  
   p_design_taxa  <-   ggplot ( data =  d,  aes ( y =  dARR,  x =  exp_design,  col =  taxonomic_group, 
        size =  precision))  +   geom_hline ( yintercept =   0 ,  linetype =   2 ,  colour =   "black" , 
        alpha =   0.5 )  +   geom_jitter ( alpha =   0.5 )  +   scale_size_continuous ( range =   c ( 1 , 
        7 ))  +   labs ( x =   "Experimental design" ,  y =   "dARR" )  +   theme_classic () 
    
    # Plot variation in dARR between experimental designs (life history variation  
    # and persistence), with points coloured for each metric  
   p_design_metric  <-   ggplot ( data =  d,  aes ( y =  dARR,  x =  exp_design,  col =  metric,  size =  precision))  +  
        geom_hline ( yintercept =   0 ,  linetype =   2 ,  colour =   "black" ,  alpha =   0.5 )  +   geom_jitter ( alpha =   0.5 )  +  
        scale_size_continuous ( range =   c ( 1 ,  7 ))  +   labs ( x =   "Experimental design" ,  y =   "dARR" )  +  
        theme_classic () 
    
   (p_design  +  p_design_var) / (p_design_taxa  +  p_design_metric)   # Combine plots     
   
       ggbetweenstats ( data =  d,  y =  dARR,  x =  exp_design)   # dARR variation between designs     
   
       ggbetweenstats ( data =  d,  y =  precision,  x =  exp_design)   # Precision variation between designs     
   
 
 
 initial vs. persistent effects 
       # Plot variation in dARR between initial and persistent effect sizes  
    ggplot ( data =  d,  aes ( y =  dARR,  x =  brought_common_temp,  col =  brought_common_temp, 
        size =  precision))  +   geom_hline ( yintercept =   0 ,  linetype =   2 ,  colour =   "black" , 
        alpha =   0.5 )  +   geom_jitter ( alpha =   0.5 )  +   scale_size_continuous ( range =   c ( 1 , 
        7 ))  +   labs ( x =   "Experimental design" ,  y =   "dARR" )  +   theme_classic ()    
   
       ggbetweenstats ( data =  d,  y =  dARR,  x =  brought_common_temp)    
   
       # Plot variation in precision between initial and persistent effect sizes  
    ggplot ( data =  d,  aes ( y =  precision,  x =  brought_common_temp,  col =  brought_common_temp))  +  
        geom_hline ( yintercept =   0 ,  linetype =   2 ,  colour =   "black" ,  alpha =   0.5 )  +   geom_jitter ( alpha =   0.5 )  +  
        scale_size_continuous ( range =   c ( 1 ,  7 ))  +   labs ( x =   "Experimental design" ,  y =   "Precision (1/SE)" )  +  
        theme_classic ()    
   
       ggbetweenstats ( data =  d,  y =  precision,  x =  brought_common_temp)    
   
 
 
 Metric type 
       # Plot variation in dARR between metric types  
   p_metric  <-   ggplot ( data =  d,  aes ( y =  dARR,  x =  metric,  col =  metric,  size =  precision))  +  
        geom_hline ( yintercept =   0 ,  linetype =   2 ,  colour =   "black" ,  alpha =   0.5 )  +   geom_jitter ( alpha =   0.5 )  +  
        scale_size_continuous ( range =   c ( 1 ,  7 ))  +   labs ( x =   "Metric type" ,  y =   "dARR" )  +  
        theme_classic () 
    
    # Plot variation in precision between metric types  
   p_metric_prec  <-   ggplot ( data =  d,  aes ( y =  precision,  x =  metric,  col =  metric))  +  
        geom_hline ( yintercept =   0 ,  linetype =   2 ,  colour =   "black" ,  alpha =   0.5 )  +   geom_jitter ( alpha =   0.5 )  +  
        scale_size_continuous ( range =   c ( 1 ,  7 ))  +   labs ( x =   "Metric type" ,  y =   "Precision (1/SE)" )  +  
        theme_classic () 
    
    # Plot variation in dARR between metric types, with different colours for each  
    # taxomic groups  
   p_metric_taxa  <-   ggplot ( data =  d,  aes ( y =  dARR,  x =  metric,  col =  taxonomic_group, 
        size =  precision))  +   geom_hline ( yintercept =   0 ,  linetype =   2 ,  colour =   "black" , 
        alpha =   0.5 )  +   geom_jitter ( alpha =   0.5 )  +   scale_size_continuous ( range =   c ( 1 , 
        7 ))  +   labs ( x =   "Metric type" ,  y =   "dARR" )  +   theme_classic () 
    
    # Plot variation in precision between metric types, with different colours for  
    # each taxomic groups  
   p_metric_design  <-   ggplot ( data =  d,  aes ( y =  dARR,  x =  metric,  col =  exp_design,  size =  precision))  +  
        geom_hline ( yintercept =   0 ,  linetype =   2 ,  colour =   "black" ,  alpha =   0.5 )  +   geom_jitter ( alpha =   0.5 )  +  
        scale_size_continuous ( range =   c ( 1 ,  7 ))  +   labs ( x =   "Metric type" ,  y =   "dARR" )  +  
        theme_classic () 
    
   (p_metric  +  p_metric_prec) / (p_metric_taxa  +  p_metric_design)   # Combine plots     
   
       ggbetweenstats ( data =  d,  y =  dARR,  x =  metric)   # dARR variation between metrics     
   
       ggbetweenstats ( data =  d,  y =  precision,  x =  metric)   # precision variation between metrics     
   
 
 
 Heating rate 
       # Plot variation in dARR with heating rate  
    ggplot ( data =  d,  aes ( y =  dARR,  x =  ramping,  size =  precision),  alpha =   0.5 )  +   geom_hline ( yintercept =   0 , 
        linetype =   2 ,  colour =   "black" ,  alpha =   0.5 )  +   geom_jitter ( alpha =   0.5 )  +   scale_size_continuous ( range =   c ( 1 , 
        7 ))  +   labs ( x =   "age_tested" ,  y =   "dARR" )  +   theme_classic ()  +   geom_smooth ( method =   "lm" ) 
    
    ggscatterstats ( data =  d,  x =  ramping,  y =  dARR)    
   
       # Plot variation in precision with heating rate  
    ggplot ( data =  d,  aes ( y =  precision,  x =  ramping),  alpha =   0.5 )  +   geom_hline ( yintercept =   0 , 
        linetype =   2 ,  colour =   "black" ,  alpha =   0.5 )  +   geom_jitter ( alpha =   0.5 )  +   scale_size_continuous ( range =   c ( 1 , 
        7 ))  +   labs ( x =   "age_tested" ,  y =   "Precision (1/SE)" )  +   theme_classic ()  +   geom_smooth ( method =   "lm" ) 
    
    ggscatterstats ( data =  d,  x =  ramping,  y =  precision)    
   
 
 
 Viviparous or Oviparous exposure 
 Note that external fertilizers were considered as “oviparous” because
the temperature treatment was experienced through the egg, instead of
through the mothers’ body. 
       # Plot variation in dARR between viviparous and oviparous animals  
    ggplot ( data =  d,  aes ( y =  dARR,  x =  reproduction_mode,  col =  reproduction_mode,  size =  precision))  +  
        geom_hline ( yintercept =   0 ,  linetype =   2 ,  colour =   "black" ,  alpha =   0.5 )  +   geom_jitter ( alpha =   0.5 )  +  
        scale_size_continuous ( range =   c ( 1 ,  7 ))  +   labs ( x =   "Experimental design" ,  y =   "dARR" )  +  
        theme_classic ()    
   
       ggbetweenstats ( data =  d,  y =  dARR,  x =  reproduction_mode)    
   
       # Plot variation in precision between viviparous and oviparous animals  
    ggplot ( data =  d,  aes ( y =  precision,  x =  reproduction_mode,  col =  reproduction_mode))  +  
        geom_hline ( yintercept =   0 ,  linetype =   2 ,  colour =   "black" ,  alpha =   0.5 )  +   geom_jitter ( alpha =   0.5 )  +  
        scale_size_continuous ( range =   c ( 1 ,  7 ))  +   labs ( x =   "Experimental design" ,  y =   "Precision (1/SE)" )  +  
        theme_classic ()    
   
       ggbetweenstats ( data =  d,  y =  precision,  x =  reproduction_mode)    
   
 
 
 Sex 
       # Plot variation in dARR between sexes  
    ggplot ( data =  d,  aes ( y =  dARR,  x =  sex,  col =  sex,  size =  precision))  +   geom_hline ( yintercept =   0 , 
        linetype =   2 ,  colour =   "black" ,  alpha =   0.5 )  +   geom_jitter ( alpha =   0.5 )  +   scale_size_continuous ( range =   c ( 1 , 
        7 ))  +   labs ( x =   "Experimental design" ,  y =   "dARR" )  +   theme_classic ()    
   
       ggbetweenstats ( data =  d,  y =  dARR,  x =  sex)    
   
       # Plot variation in precision between sexes  
    ggplot ( data =  d,  aes ( y =  precision,  x =  sex,  col =  sex))  +   geom_hline ( yintercept =   0 , 
        linetype =   2 ,  colour =   "black" ,  alpha =   0.5 )  +   geom_jitter ( alpha =   0.5 )  +   scale_size_continuous ( range =   c ( 1 , 
        7 ))  +   labs ( x =   "Experimental design" ,  y =   "Precision (1/SE)" )  +   theme_classic ()    
   
       ggbetweenstats ( data =  d,  y =  precision,  x =  sex)    
   
 
 
 Age tested 
       # Plot variation in dARR with the age of testing  
    ggplot ( data =  d,  aes ( y =  dARR,  x =  age_tested,  size =  precision),  alpha =   0.5 )  +  
        geom_hline ( yintercept =   0 ,  linetype =   2 ,  colour =   "black" ,  alpha =   0.5 )  +   geom_jitter ( alpha =   0.5 )  +  
        scale_size_continuous ( range =   c ( 1 ,  7 ))  +   labs ( x =   "age_tested" ,  y =   "dARR" )  +  
        theme_classic ()  +   geom_smooth ( method =   "lm" ) 
    
    ggscatterstats ( data =  d,  x =  age_tested,  y =  dARR)    
   
       # Plot variation in precision with the age of testing  
    ggplot ( data =  d,  aes ( y =  precision,  x =  age_tested),  alpha =   0.5 )  +   geom_hline ( yintercept =   0 , 
        linetype =   2 ,  colour =   "black" ,  alpha =   0.5 )  +   geom_jitter ( alpha =   0.5 )  +   scale_size_continuous ( range =   c ( 1 , 
        7 ))  +   labs ( x =   "age_tested" ,  y =   "Precision (1/SE)" )  +   theme_classic ()  +   geom_smooth ( method =   "lm" ) 
    
    ggscatterstats ( data =  d,  x =  precision,  y =  dARR)    
   
 
 
 Heat tolerance endpoint 
       # Plot variation in dARR between heat tolerance endpoints  
    ggplot ( data =  d,  aes ( y =  dARR,  x =  endpoint,  col =  endpoint,  size =  precision))  +  
        geom_hline ( yintercept =   0 ,  linetype =   2 ,  colour =   "black" ,  alpha =   0.5 )  +   geom_jitter ( alpha =   0.5 )  +  
        scale_size_continuous ( range =   c ( 1 ,  7 ))  +   labs ( x =   "Experimental design" ,  y =   "dARR" )  +  
        theme_classic ()    
   
       ggbetweenstats ( data =  d,  y =  dARR,  x =  endpoint)    
   
       # Plot variation in precision between heat tolerance endpoints  
    ggplot ( data =  d,  aes ( y =  precision,  x =  endpoint,  col =  endpoint))  +   geom_hline ( yintercept =   0 , 
        linetype =   2 ,  colour =   "black" ,  alpha =   0.5 )  +   geom_jitter ( alpha =   0.5 )  +   scale_size_continuous ( range =   c ( 1 , 
        7 ))  +   labs ( x =   "Experimental design" ,  y =   "Precision (1/SE)" )  +   theme_classic ()    
   
       ggbetweenstats ( data =  d,  y =  precision,  x =  endpoint)    
   
 
 
 Hatching origin 
       # Plot variation in dARR between locations where animals hatched  
    ggplot ( data =  d,  aes ( y =  dARR,  x =  origin_hatching,  col =  origin_hatching,  size =  precision))  +  
        geom_hline ( yintercept =   0 ,  linetype =   2 ,  colour =   "black" ,  alpha =   0.5 )  +   geom_jitter ( alpha =   0.5 )  +  
        scale_size_continuous ( range =   c ( 1 ,  7 ))  +   labs ( x =   "Origin" ,  y =   "dARR" )  +   theme_classic ()    
   
       ggbetweenstats ( data =  d,  y =  dARR,  x =  origin_hatching)    
   
       # Plot variation in precision between locations where animals hatched  
    ggplot ( data =  d,  aes ( y =  precision,  x =  origin_hatching,  col =  origin_hatching))  +  
        geom_hline ( yintercept =   0 ,  linetype =   2 ,  colour =   "black" ,  alpha =   0.5 )  +   geom_jitter ( alpha =   0.5 )  +  
        scale_size_continuous ( range =   c ( 1 ,  7 ))  +   labs ( x =   "Origin" ,  y =   "Precision (1/SE)" )  +  
        theme_classic ()    
   
       ggbetweenstats ( data =  d,  y =  precision,  x =  origin_hatching)    
   
 
 
 Body mass 
       # Plot variation in dARR with body mass  
    ggplot ( data =  d,  aes ( y =  dARR,  x =  body_mass,  size =  precision),  alpha =   0.5 )  +   geom_hline ( yintercept =   0 , 
        linetype =   2 ,  colour =   "black" ,  alpha =   0.5 )  +   geom_jitter ( alpha =   0.5 )  +   scale_size_continuous ( range =   c ( 1 , 
        7 ))  +   labs ( x =   "Body mass" ,  y =   "dARR" )  +   theme_classic ()  +   geom_smooth ( method =   "lm" ) 
    
    ggscatterstats ( data =  d,  x =  body_mass,  y =  dARR)    
   
       # Plot variation in precision with body mass  
    ggplot ( data =  d,  aes ( y =  precision,  x =  body_mass),  alpha =   0.5 )  +   geom_hline ( yintercept =   0 , 
        linetype =   2 ,  colour =   "black" ,  alpha =   0.5 )  +   geom_jitter ( alpha =   0.5 )  +   scale_size_continuous ( range =   c ( 1 , 
        7 ))  +   labs ( x =   "Body mass" ,  y =   "Precision (1/SE)" )  +   theme_classic ()  +   geom_smooth ( method =   "lm" ) 
    
    ggscatterstats ( data =  d,  x =  body_mass,  y =  precision)    
   
 
 
 Whether the acclimation temperatures used are independent from
housing temperatures prior to acclimation 
       # Plot variation in dARR depending on whether all temperature acclimation  
    # treatments were independent from housing temperatures prior to acclimation  
    ggplot ( data =  d,  aes ( y =  dARR,  x =  incubation_independent,  col =  incubation_independent, 
        size =  precision))  +   geom_hline ( yintercept =   0 ,  linetype =   2 ,  colour =   "black" , 
        alpha =   0.5 )  +   geom_jitter ( alpha =   0.5 )  +   scale_size_continuous ( range =   c ( 1 , 
        7 ))  +   labs ( x =   "Origin" ,  y =   "dARR" )  +   theme_classic ()    
   
       ggbetweenstats ( data =  d,  y =  dARR,  x =  incubation_independent)    
   
       # Plot variation in precision depending on whether all temperature acclimation  
    # treatments were independent from housing temperatures prior to acclimation  
    ggplot ( data =  d,  aes ( y =  precision,  x =  incubation_independent,  col =  incubation_independent))  +  
        geom_hline ( yintercept =   0 ,  linetype =   2 ,  colour =   "black" ,  alpha =   0.5 )  +   geom_jitter ( alpha =   0.5 )  +  
        scale_size_continuous ( range =   c ( 1 ,  7 ))  +   labs ( x =   "Origin" ,  y =   "Precision (1/SE)" )  +  
        theme_classic ()    
   
       ggbetweenstats ( data =  d,  y =  precision,  x =  incubation_independent)    
   
 
 
 The time after which survival was assessed in static assays 
       # Plot variation in dARR depending on experiment duration in static assays  
    ggplot ( data =  d,  aes ( y =  dARR,  x =  set_time,  size =  precision),  alpha =   0.5 )  +   geom_hline ( yintercept =   0 , 
        linetype =   2 ,  colour =   "black" ,  alpha =   0.5 )  +   geom_jitter ( alpha =   0.5 )  +   scale_size_continuous ( range =   c ( 1 , 
        7 ))  +   labs ( x =   "Body mass" ,  y =   "dARR" )  +   theme_classic ()  +   geom_smooth ( method =   "lm" ) 
    
    ggscatterstats ( data =  d,  x =  set_time,  y =  dARR)    
   
       # Plot variation in precision depending on experiment duration in static assays  
    ggplot ( data =  d,  aes ( y =  precision,  x =  set_time),  alpha =   0.5 )  +   geom_hline ( yintercept =   0 , 
        linetype =   2 ,  colour =   "black" ,  alpha =   0.5 )  +   geom_jitter ( alpha =   0.5 )  +   scale_size_continuous ( range =   c ( 1 , 
        7 ))  +   labs ( x =   "Body mass" ,  y =   "Precision (1/SE)" )  +   theme_classic ()  +   geom_smooth ( method =   "lm" ) 
    
    ggscatterstats ( data =  d,  x =  set_time,  y =  precision)    
   
 
 
 Concerns with data quality 
      d  <-  d  %>%  
        mutate ( is_concern =   ifelse ( is.na (major_concerns),  "no" ,  "yes" ))   # Create a new column to indicate whether there are concerns with the data or not  
    
    # Plot variation in dARR depending on whether the study has a risk of bias  
    ggplot ( data =  d,  aes ( y =  dARR,  x =  is_concern,  col =  is_concern,  size =  precision))  +  
        geom_hline ( yintercept =   0 ,  linetype =   2 ,  colour =   "black" ,  alpha =   0.5 )  +   geom_jitter ( alpha =   0.5 )  +  
        scale_size_continuous ( range =   c ( 1 ,  7 ))  +   labs ( x =   "Major data concerns" ,  y =   "dARR" )  +  
        theme_classic ()    
   
       ggbetweenstats ( data =  d,  x =  is_concern,  y =  dARR)    
   
       # Plot variation in precision depending on whether the study has a risk of bias  
    ggplot ( data =  d,  aes ( y =  precision,  x =  is_concern,  col =  is_concern))  +   geom_hline ( yintercept =   0 , 
        linetype =   2 ,  colour =   "black" ,  alpha =   0.5 )  +   geom_jitter ( alpha =   0.5 )  +   scale_size_continuous ( range =   c ( 1 , 
        7 ))  +   labs ( x =   "Major data concerns" ,  y =   "Precision (1/SE)" )  +   theme_classic ()    
   
       ggbetweenstats ( data =  d,  x =  is_concern,  y =  precision)    
   
 
 
 Imputed vs original data 
       # Plot variation in dARR depending on whether the sampling variance was imputed  
    ggplot ( data =  d,  aes ( y =  dARR,  x =  imputed,  col =  imputed,  size =  precision))  +   geom_hline ( yintercept =   0 , 
        linetype =   2 ,  colour =   "black" ,  alpha =   0.5 )  +   geom_jitter ( alpha =   0.5 )  +   scale_size_continuous ( range =   c ( 1 , 
        7 ))  +   labs ( x =   "SD imputation" ,  y =   "dARR" )  +   theme_classic ()    
   
       ggbetweenstats ( data =  d,  y =  dARR,  x =  imputed)    
   
       # Plot variation in precision depending on whether the sampling variance was  
    # imputed  
    ggplot ( data =  d,  aes ( y =  precision,  x =  imputed,  col =  Var_dARR,  size =  precision))  +  
        geom_hline ( yintercept =   0 ,  linetype =   2 ,  colour =   "black" ,  alpha =   0.5 )  +   geom_jitter ( alpha =   0.5 )  +  
        scale_size_continuous ( range =   c ( 1 ,  7 ))  +   labs ( x =   "SD imputation" ,  y =   "Precision (1/SE)" )  +  
        theme_classic ()    
   
       ggbetweenstats ( data =  d,  y =  precision,  x =  imputed)    
   
 
 
  
 
 
 
 
  Phylogeny  
  Potamilus alatus  was not classified in the  Open Tree of
Life taxonomy . Quick searches in the  Integrated Taxonomic
Information System  for all bivalve species included in our study
indicate that  Potamilus alatus  belongs to the
 Lampsilini  tribe. Species of the genus  Villosa ,
 Ligumia ,  Lampsilis  and  Ellipsaria  all belong
to this tribe. 
 Therefore, this species was arbitrarily added next to  Villosa
delumbis  and polytomies were resolved at random 
      d  <-   as.data.frame (d) 
    
   d $ genus_species  =   as.factor (d $ genus_species)   # Convert species name as factor  
   taxa  <-   tnrs_match_names ( names =   levels (d $ genus_species),  context =   "Animals" )   # Match species name to the Open Tree of Life taxonomy  
    kable (taxa)   #  Some species are synonyms, but they don't have >1 match so it's all good.     
 
 
 
 
search_string
 
 
unique_name
 
 
approximate_match
 
 
ott_id
 
 
is_synonym
 
 
flags
 
 
number_matches
 
 
 
 
 
 
acanthochromis polyacanthus
 
 
Acanthochromis polyacanthus
 
 
FALSE
 
 
100410
 
 
FALSE
 
 
incertae_sedis_inherited
 
 
1
 
 
 
 
acartia tonsa
 
 
Acartia tonsa
 
 
FALSE
 
 
265973
 
 
FALSE
 
 
sibling_higher
 
 
1
 
 
 
 
acipenser brevirostrum
 
 
Acipenser brevirostrum
 
 
FALSE
 
 
82746
 
 
FALSE
 
 
 
 
1
 
 
 
 
acipenser fulvescens
 
 
Acipenser fulvescens
 
 
FALSE
 
 
698270
 
 
FALSE
 
 
 
 
1
 
 
 
 
acipenser transmontanus
 
 
Acipenser transmontanus
 
 
FALSE
 
 
378925
 
 
FALSE
 
 
 
 
1
 
 
 
 
aedes aegypti
 
 
Aedes aegypti
 
 
FALSE
 
 
269666
 
 
FALSE
 
 
 
 
1
 
 
 
 
agosia chrysogaster
 
 
Agosia chrysogaster
 
 
FALSE
 
 
308519
 
 
FALSE
 
 
 
 
1
 
 
 
 
alasmidonta varicosa
 
 
Alasmidonta varicosa
 
 
FALSE
 
 
381439
 
 
FALSE
 
 
sibling_higher
 
 
1
 
 
 
 
amalosia lesueurii
 
 
Amalosia lesueurii
 
 
FALSE
 
 
295655
 
 
FALSE
 
 
 
 
2
 
 
 
 
amblema plicata
 
 
Amblema plicata
 
 
FALSE
 
 
88263
 
 
FALSE
 
 
sibling_higher
 
 
1
 
 
 
 
ambystoma mexicanum
 
 
Ambystoma mexicanum
 
 
FALSE
 
 
984726
 
 
FALSE
 
 
 
 
1
 
 
 
 
amphiprion melanopus
 
 
Amphiprion melanopus
 
 
FALSE
 
 
45635
 
 
FALSE
 
 
incertae_sedis_inherited
 
 
1
 
 
 
 
anisotremus scapularis
 
 
Anisotremus scapularis
 
 
FALSE
 
 
115727
 
 
FALSE
 
 
 
 
1
 
 
 
 
anolis sagrei
 
 
Anolis sagrei
 
 
FALSE
 
 
970153
 
 
FALSE
 
 
 
 
1
 
 
 
 
anopheles albimanus
 
 
Anopheles albimanus
 
 
FALSE
 
 
793189
 
 
FALSE
 
 
 
 
1
 
 
 
 
apostichopus japonicus
 
 
Apostichopus japonicus
 
 
FALSE
 
 
721060
 
 
FALSE
 
 
 
 
1
 
 
 
 
argyrosomus regius
 
 
Argyrosomus regius
 
 
FALSE
 
 
204035
 
 
FALSE
 
 
 
 
1
 
 
 
 
belonesox belizanus
 
 
Belonesox belizanus
 
 
FALSE
 
 
913297
 
 
FALSE
 
 
 
 
1
 
 
 
 
bidyanus bidyanus
 
 
Bidyanus bidyanus
 
 
FALSE
 
 
600523
 
 
FALSE
 
 
 
 
1
 
 
 
 
bufo gargarizans
 
 
Bufo gargarizans
 
 
FALSE
 
 
279549
 
 
FALSE
 
 
 
 
1
 
 
 
 
busseola fusca
 
 
Busseola fusca
 
 
FALSE
 
 
535636
 
 
FALSE
 
 
 
 
1
 
 
 
 
catla catla
 
 
Labeo catla
 
 
FALSE
 
 
719907
 
 
TRUE
 
 
sibling_higher
 
 
1
 
 
 
 
chilo partellus
 
 
Chilo partellus
 
 
FALSE
 
 
1062112
 
 
FALSE
 
 
 
 
1
 
 
 
 
chiloscyllium plagiosum
 
 
Chiloscyllium plagiosum
 
 
FALSE
 
 
962561
 
 
FALSE
 
 
 
 
1
 
 
 
 
cirrhinus mrigala
 
 
Cirrhinus mrigala
 
 
FALSE
 
 
10821
 
 
FALSE
 
 
 
 
2
 
 
 
 
clupea harengus
 
 
Clupea harengus
 
 
FALSE
 
 
1005932
 
 
FALSE
 
 
 
 
1
 
 
 
 
culex pipiens
 
 
Culex pipiens
 
 
FALSE
 
 
218684
 
 
FALSE
 
 
 
 
1
 
 
 
 
cydia pomonella
 
 
Cydia pomonella
 
 
FALSE
 
 
819146
 
 
FALSE
 
 
 
 
1
 
 
 
 
cyprinodon nevadensis
 
 
Cyprinodon nevadensis
 
 
FALSE
 
 
617460
 
 
FALSE
 
 
 
 
1
 
 
 
 
cyprinus carpio
 
 
Cyprinus carpio
 
 
FALSE
 
 
429083
 
 
FALSE
 
 
 
 
1
 
 
 
 
danio rerio
 
 
Danio rerio
 
 
FALSE
 
 
1005914
 
 
FALSE
 
 
 
 
1
 
 
 
 
daphnia galeata
 
 
Daphnia galeata
 
 
FALSE
 
 
46786
 
 
FALSE
 
 
sibling_higher
 
 
1
 
 
 
 
daphnia magna
 
 
Daphnia magna
 
 
FALSE
 
 
668392
 
 
FALSE
 
 
sibling_higher
 
 
1
 
 
 
 
daphnia pulicaria
 
 
Daphnia pulicaria
 
 
FALSE
 
 
668394
 
 
FALSE
 
 
sibling_higher
 
 
1
 
 
 
 
dicentrarchus labrax
 
 
Dicentrarchus labrax
 
 
FALSE
 
 
3549
 
 
FALSE
 
 
 
 
1
 
 
 
 
drosophila melanogaster
 
 
Drosophila melanogaster
 
 
FALSE
 
 
505714
 
 
FALSE
 
 
 
 
1
 
 
 
 
drosophila suzukii
 
 
Drosophila suzukii
 
 
FALSE
 
 
227752
 
 
FALSE
 
 
 
 
1
 
 
 
 
ellipsaria lineolata
 
 
Ellipsaria lineolata
 
 
FALSE
 
 
190876
 
 
FALSE
 
 
 
 
1
 
 
 
 
engystomops pustulosus
 
 
Engystomops pustulosus
 
 
FALSE
 
 
262675
 
 
FALSE
 
 
 
 
1
 
 
 
 
enteroctopus megalocyathus
 
 
Enteroctopus megalocyathus
 
 
FALSE
 
 
746557
 
 
FALSE
 
 
 
 
1
 
 
 
 
evechinus chloroticus
 
 
Evechinus chloroticus
 
 
FALSE
 
 
924047
 
 
FALSE
 
 
 
 
1
 
 
 
 
gambusia affinis
 
 
Gambusia affinis
 
 
FALSE
 
 
617445
 
 
FALSE
 
 
 
 
1
 
 
 
 
glossina pallidipes
 
 
Glossina pallidipes
 
 
FALSE
 
 
753729
 
 
FALSE
 
 
 
 
1
 
 
 
 
haliotis diversicolor
 
 
Haliotis diversicolor
 
 
FALSE
 
 
780542
 
 
FALSE
 
 
 
 
1
 
 
 
 
harmonia axyridis
 
 
Harmonia axyridis
 
 
FALSE
 
 
258281
 
 
FALSE
 
 
 
 
1
 
 
 
 
heterodontus portusjacksoni
 
 
Heterodontus portusjacksoni
 
 
FALSE
 
 
1019118
 
 
FALSE
 
 
 
 
1
 
 
 
 
hippocampus erectus
 
 
Hippocampus erectus
 
 
FALSE
 
 
630161
 
 
FALSE
 
 
 
 
1
 
 
 
 
hodotermes mossambicus
 
 
Hodotermes mossambicus
 
 
FALSE
 
 
973344
 
 
FALSE
 
 
 
 
1
 
 
 
 
hypomesus transpacificus
 
 
Hypomesus transpacificus
 
 
FALSE
 
 
348839
 
 
FALSE
 
 
 
 
1
 
 
 
 
hypophthalmichthys molitrix
 
 
Hypophthalmichthys molitrix
 
 
FALSE
 
 
35786
 
 
FALSE
 
 
 
 
1
 
 
 
 
hypsiboas faber
 
 
Boana faber
 
 
FALSE
 
 
500272
 
 
TRUE
 
 
 
 
1
 
 
 
 
ictalurus punctatus
 
 
Ictalurus punctatus
 
 
FALSE
 
 
701523
 
 
FALSE
 
 
 
 
1
 
 
 
 
ischnura elegans
 
 
Ischnura elegans
 
 
FALSE
 
 
253311
 
 
FALSE
 
 
 
 
1
 
 
 
 
kryptolebias marmoratus
 
 
Kryptolebias marmoratus
 
 
FALSE
 
 
229709
 
 
FALSE
 
 
 
 
1
 
 
 
 
labeo rohita
 
 
Labeo rohita
 
 
FALSE
 
 
160616
 
 
FALSE
 
 
sibling_higher
 
 
1
 
 
 
 
lampropholis coggeri
 
 
Lampropholis coggeri
 
 
FALSE
 
 
1073190
 
 
FALSE
 
 
 
 
1
 
 
 
 
lampsilis abrupta
 
 
Lampsilis abrupta
 
 
FALSE
 
 
1080021
 
 
FALSE
 
 
sibling_higher
 
 
1
 
 
 
 
lampsilis cariosa
 
 
Lampsilis cariosa
 
 
FALSE
 
 
788597
 
 
FALSE
 
 
sibling_higher
 
 
1
 
 
 
 
lampsilis radiata
 
 
Lampsilis radiata
 
 
FALSE
 
 
281722
 
 
FALSE
 
 
sibling_higher
 
 
1
 
 
 
 
lampsilis siliquoidea
 
 
Lampsilis siliquoidea
 
 
FALSE
 
 
765106
 
 
FALSE
 
 
sibling_higher
 
 
1
 
 
 
 
lates calcarifer
 
 
Lates calcarifer
 
 
FALSE
 
 
437601
 
 
FALSE
 
 
incertae_sedis_inherited
 
 
1
 
 
 
 
lepdomeda copei
 
 
Lepidomeda copei
 
 
TRUE
 
 
1073687
 
 
FALSE
 
 
 
 
1
 
 
 
 
leucoraja erinacea
 
 
Leucoraja erinacea
 
 
FALSE
 
 
493418
 
 
FALSE
 
 
 
 
1
 
 
 
 
ligumia recta
 
 
Ligumia recta
 
 
FALSE
 
 
2851633
 
 
FALSE
 
 
sibling_higher
 
 
1
 
 
 
 
limnodynastes peronii
 
 
Limnodynastes peronii
 
 
FALSE
 
 
276279
 
 
FALSE
 
 
sibling_higher
 
 
1
 
 
 
 
litopenaeus stylirostris
 
 
Penaeus stylirostris
 
 
FALSE
 
 
957079
 
 
TRUE
 
 
 
 
1
 
 
 
 
litopenaeus vannamei
 
 
Penaeus vannamei
 
 
FALSE
 
 
169168
 
 
TRUE
 
 
 
 
1
 
 
 
 
loligo vulgaris
 
 
Loligo vulgaris
 
 
FALSE
 
 
1054848
 
 
FALSE
 
 
 
 
1
 
 
 
 
loxechinus albus
 
 
Loxechinus albus
 
 
FALSE
 
 
555689
 
 
FALSE
 
 
 
 
1
 
 
 
 
lutjanus guttatus
 
 
Lutjanus guttatus
 
 
FALSE
 
 
445054
 
 
FALSE
 
 
 
 
1
 
 
 
 
macrobrachium rosenbergii
 
 
Macrobrachium rosenbergii
 
 
FALSE
 
 
708755
 
 
FALSE
 
 
 
 
1
 
 
 
 
manduca sexta
 
 
Manduca sexta
 
 
FALSE
 
 
116647
 
 
FALSE
 
 
 
 
1
 
 
 
 
mauremys mutica
 
 
Mauremys mutica
 
 
FALSE
 
 
679467
 
 
FALSE
 
 
 
 
1
 
 
 
 
mauremys reevesii
 
 
Mauremys reevesii
 
 
FALSE
 
 
829098
 
 
FALSE
 
 
 
 
1
 
 
 
 
megalonaias nervosa
 
 
Megalonaias nervosa
 
 
FALSE
 
 
609673
 
 
FALSE
 
 
 
 
1
 
 
 
 
mesocentrotus franciscanus
 
 
Mesocentrotus franciscanus
 
 
FALSE
 
 
564712
 
 
FALSE
 
 
 
 
1
 
 
 
 
metapenaeus affinis
 
 
Metapenaeus affinis
 
 
FALSE
 
 
675009
 
 
FALSE
 
 
 
 
1
 
 
 
 
micropterus salmoides
 
 
Micropterus salmoides
 
 
FALSE
 
 
230493
 
 
FALSE
 
 
 
 
1
 
 
 
 
morone saxatilis
 
 
Morone saxatilis
 
 
FALSE
 
 
223669
 
 
FALSE
 
 
 
 
1
 
 
 
 
moxostoma robustum
 
 
Moxostoma robustum
 
 
FALSE
 
 
300757
 
 
FALSE
 
 
 
 
1
 
 
 
 
myxocyprinus asiaticus
 
 
Myxocyprinus asiaticus
 
 
FALSE
 
 
141409
 
 
FALSE
 
 
 
 
1
 
 
 
 
myzus persicae
 
 
Myzus persicae
 
 
FALSE
 
 
785522
 
 
FALSE
 
 
 
 
2
 
 
 
 
nilaparvata lugens
 
 
Nilaparvata lugens
 
 
FALSE
 
 
876508
 
 
FALSE
 
 
 
 
1
 
 
 
 
octopus maya
 
 
Octopus maya
 
 
FALSE
 
 
237487
 
 
FALSE
 
 
 
 
1
 
 
 
 
oncorhynchus clarkii
 
 
Oncorhynchus clarkii
 
 
FALSE
 
 
800432
 
 
FALSE
 
 
 
 
1
 
 
 
 
oncorhynchus kisutch
 
 
Oncorhynchus kisutch
 
 
FALSE
 
 
739917
 
 
FALSE
 
 
 
 
1
 
 
 
 
oncorhynchus mykiss
 
 
Oncorhynchus mykiss (species in domain Eukaryota)
 
 
FALSE
 
 
165368
 
 
FALSE
 
 
 
 
1
 
 
 
 
oncorhynchus nerka
 
 
Oncorhynchus nerka
 
 
FALSE
 
 
165375
 
 
FALSE
 
 
 
 
1
 
 
 
 
oncorhynchus tshawytscha
 
 
Oncorhynchus tshawytscha
 
 
FALSE
 
 
730762
 
 
FALSE
 
 
 
 
1
 
 
 
 
oreochromis niloticus
 
 
Oreochromis niloticus
 
 
FALSE
 
 
288063
 
 
FALSE
 
 
sibling_higher
 
 
1
 
 
 
 
oryzias melastigma
 
 
Oryzias melastigma
 
 
FALSE
 
 
179491
 
 
FALSE
 
 
 
 
2
 
 
 
 
pangasius pangasius
 
 
Pangasius pangasius
 
 
FALSE
 
 
216174
 
 
FALSE
 
 
 
 
1
 
 
 
 
parabramis pekinensis
 
 
Parabramis pekinensis
 
 
FALSE
 
 
837024
 
 
FALSE
 
 
 
 
1
 
 
 
 
paralichthys californicus
 
 
Paralichthys californicus
 
 
FALSE
 
 
301358
 
 
FALSE
 
 
 
 
1
 
 
 
 
pelodiscus sinensis
 
 
Pelodiscus sinensis
 
 
FALSE
 
 
143391
 
 
FALSE
 
 
 
 
1
 
 
 
 
penaeus monodon
 
 
Penaeus monodon
 
 
FALSE
 
 
212713
 
 
FALSE
 
 
 
 
1
 
 
 
 
petromyzon marinus
 
 
Petromyzon marinus
 
 
FALSE
 
 
227494
 
 
FALSE
 
 
 
 
1
 
 
 
 
physalaemus cuvieri
 
 
Physalaemus cuvieri
 
 
FALSE
 
 
642784
 
 
FALSE
 
 
 
 
1
 
 
 
 
pimephales promelas
 
 
Pimephales promelas
 
 
FALSE
 
 
564459
 
 
FALSE
 
 
 
 
1
 
 
 
 
pleurodema diplolistris
 
 
Pleurodema diplolister
 
 
FALSE
 
 
806505
 
 
TRUE
 
 
sibling_higher
 
 
1
 
 
 
 
portunus pelagicus
 
 
Portunus pelagicus
 
 
FALSE
 
 
599991
 
 
FALSE
 
 
 
 
1
 
 
 
 
potamilus alatus
 
 
Potamilus alatus
 
 
FALSE
 
 
732215
 
 
FALSE
 
 
unplaced_inherited
 
 
1
 
 
 
 
premnas biaculeatus
 
 
Premnas biaculeatus
 
 
FALSE
 
 
519096
 
 
FALSE
 
 
incertae_sedis_inherited
 
 
1
 
 
 
 
procypris rabaudi
 
 
Procypris rabaudi
 
 
FALSE
 
 
328803
 
 
FALSE
 
 
 
 
1
 
 
 
 
propylea japonica
 
 
Propylea japonica
 
 
FALSE
 
 
790672
 
 
FALSE
 
 
 
 
1
 
 
 
 
pseudacris hypochondriaca
 
 
Pseudacris hypochondriaca
 
 
FALSE
 
 
3619952
 
 
FALSE
 
 
 
 
1
 
 
 
 
pseudococcus jackbeardsleyi
 
 
Pseudococcus jackbeardsleyi
 
 
FALSE
 
 
135880
 
 
FALSE
 
 
 
 
1
 
 
 
 
pseudocrenilabrus multicolor
 
 
Pseudocrenilabrus multicolor
 
 
FALSE
 
 
338070
 
 
FALSE
 
 
 
 
1
 
 
 
 
pseudopleuronectes yokohamae
 
 
Pseudopleuronectes yokohamae
 
 
FALSE
 
 
608831
 
 
FALSE
 
 
 
 
1
 
 
 
 
pterophyllum scalare
 
 
Pterophyllum scalare
 
 
FALSE
 
 
220929
 
 
FALSE
 
 
 
 
1
 
 
 
 
rana temporaria
 
 
Rana temporaria
 
 
FALSE
 
 
14718
 
 
FALSE
 
 
 
 
1
 
 
 
 
rhinella granulosa
 
 
Rhinella granulosa
 
 
FALSE
 
 
962298
 
 
FALSE
 
 
 
 
1
 
 
 
 
rhinella icterica
 
 
Rhinella icterica
 
 
FALSE
 
 
745776
 
 
FALSE
 
 
 
 
1
 
 
 
 
rhinella marina
 
 
Rhinella marina
 
 
FALSE
 
 
889340
 
 
FALSE
 
 
 
 
1
 
 
 
 
rhinella ornata
 
 
Rhinella ornata
 
 
FALSE
 
 
721631
 
 
FALSE
 
 
 
 
1
 
 
 
 
salmo salar
 
 
Salmo salar
 
 
FALSE
 
 
688328
 
 
FALSE
 
 
 
 
1
 
 
 
 
salmo trutta
 
 
Salmo trutta
 
 
FALSE
 
 
688332
 
 
FALSE
 
 
 
 
1
 
 
 
 
salvelinus alpinus
 
 
Salvelinus alpinus
 
 
FALSE
 
 
688324
 
 
FALSE
 
 
 
 
1
 
 
 
 
salvelinus fontinalis
 
 
Salvelinus fontinalis
 
 
FALSE
 
 
688339
 
 
FALSE
 
 
 
 
1
 
 
 
 
salvelinus namaycush
 
 
Salvelinus namaycush
 
 
FALSE
 
 
114152
 
 
FALSE
 
 
 
 
1
 
 
 
 
sardina pilchardus
 
 
Sardina pilchardus
 
 
FALSE
 
 
1027727
 
 
FALSE
 
 
 
 
1
 
 
 
 
scaphirhynchus albus
 
 
Scaphirhynchus albus
 
 
FALSE
 
 
962544
 
 
FALSE
 
 
 
 
1
 
 
 
 
scaphirhynchus platorynchus
 
 
Scaphirhynchus platorynchus
 
 
FALSE
 
 
904110
 
 
FALSE
 
 
 
 
1
 
 
 
 
schizopygopsis younghusbandi
 
 
Schizopygopsis younghusbandi
 
 
FALSE
 
 
203753
 
 
FALSE
 
 
 
 
1
 
 
 
 
schizothorax kozlovi
 
 
Schizothorax kozlovi
 
 
FALSE
 
 
709859
 
 
FALSE
 
 
 
 
1
 
 
 
 
sciaenops ocellatus
 
 
Sciaenops ocellatus
 
 
FALSE
 
 
433079
 
 
FALSE
 
 
 
 
1
 
 
 
 
sebastes schlegeli
 
 
Sebastes schlegelii
 
 
FALSE
 
 
206127
 
 
TRUE
 
 
sibling_higher
 
 
1
 
 
 
 
sesamia calamistis
 
 
Sesamia calamistis
 
 
FALSE
 
 
112903
 
 
FALSE
 
 
 
 
1
 
 
 
 
sitobion avenae
 
 
Sitobion avenae
 
 
FALSE
 
 
1004324
 
 
FALSE
 
 
 
 
1
 
 
 
 
solea senegalensis
 
 
Solea senegalensis
 
 
FALSE
 
 
366998
 
 
FALSE
 
 
 
 
1
 
 
 
 
sparus aurata
 
 
Sparus aurata
 
 
FALSE
 
 
760723
 
 
FALSE
 
 
 
 
1
 
 
 
 
sphoeroides annulatus
 
 
Sphoeroides annulatus
 
 
FALSE
 
 
6406
 
 
FALSE
 
 
 
 
1
 
 
 
 
thymallus arcticus
 
 
Thymallus arcticus
 
 
FALSE
 
 
713375
 
 
FALSE
 
 
 
 
1
 
 
 
 
tigriopus californicus
 
 
Tigriopus californicus
 
 
FALSE
 
 
461524
 
 
FALSE
 
 
 
 
1
 
 
 
 
tor putitora
 
 
Tor putitora
 
 
FALSE
 
 
720179
 
 
FALSE
 
 
 
 
1
 
 
 
 
trachemys scripta
 
 
Trachemys scripta
 
 
FALSE
 
 
725741
 
 
FALSE
 
 
 
 
1
 
 
 
 
villosa delumbis
 
 
Villosa delumbis
 
 
FALSE
 
 
15452
 
 
FALSE
 
 
sibling_higher
 
 
1
 
 
 
 
xenopus laevis
 
 
Xenopus laevis
 
 
FALSE
 
 
465096
 
 
FALSE
 
 
 
 
1
 
 
 
 
      taxa $ unique_name  <-   gsub ( " " ,  "_" , taxa $ unique_name)   # Replace spaces between species name by an underscore  
    
    # phylo_tree <- tol_induced_subtree(ott_ids = taxa$ott_id, label_format =  
    # 'name') # This is a comment because this code cannot run without the  
    # additional processing below.  
    
    # Potamilus alatus (ott732215) is not found in the Open tree taxonomy  
    
   d  %>%  
        filter (class  ==   "Bivalvia" )  %>%  
        select (order, family, genus_species)  %>%  
        arrange (genus_species)   # check all species of bivalves to see where Potamilus alatus sits     
  ##       order    family         genus_species
## 1  Unionida Unionidae  Alasmidonta varicosa
## 2  Unionida Unionidae       Amblema plicata
## 3  Unionida Unionidae  Ellipsaria lineolata
## 4  Unionida Unionidae     Lampsilis abrupta
## 5  Unionida Unionidae     Lampsilis abrupta
## 6  Unionida Unionidae     Lampsilis abrupta
## 7  Unionida Unionidae     Lampsilis cariosa
## 8  Unionida Unionidae     Lampsilis cariosa
## 9  Unionida Unionidae     Lampsilis radiata
## 10 Unionida Unionidae Lampsilis siliquoidea
## 11 Unionida Unionidae Lampsilis siliquoidea
## 12 Unionida Unionidae Lampsilis siliquoidea
## 13 Unionida Unionidae Lampsilis siliquoidea
## 14 Unionida Unionidae         Ligumia recta
## 15 Unionida Unionidae         Ligumia recta
## 16 Unionida Unionidae   Megalonaias nervosa
## 17 Unionida Unionidae      Potamilus alatus
## 18 Unionida Unionidae      Potamilus alatus
## 19 Unionida Unionidae      Villosa delumbis  
      taxa.tree  <-   filter (taxa, ott_id  !=   "732215" )   # remove Potamilus alatus, as it will be manually added later  
   phylo_tree  <-   tol_induced_subtree ( ott_ids =  taxa.tree $ ott_id,  label_format =   "name" )   # Generate phylogenetic tree based on the Open Tree of Life taxonomy     
      ott_in_tree  <-   ott_id (taxa.tree)[ is_in_tree ( ott_id (taxa.tree))]   # Make sure that all identifiers are in the taxonomy  
    
   phylo_tree  <-   tol_induced_subtree ( ott_ids =  ott_in_tree)   # Generate tree with all species found in the taxonomy     
       is.binary (phylo_tree)   # Check if tree is binary      
  ## [1] FALSE  
       set.seed ( 1 )   # Set a seed to resolve politomies at random, and obtain similar results  
    
   binary.tree  <-   multi2di (phylo_tree,  random =  T)   #resolve polytomies at random  
   binary.tree  <-   bind.tip (binary.tree,  tip.label =   "Potamilus_alatus" ,  where =   95 )   # Add Potamilus alatus to the tree  
    
   phylo_branched  <-   compute.brlen (binary.tree,  method =   "Grafen" ,  power =   1 )   # compute branch lengths using Grafen's method  
    
    
   phylo_branched $ tip.label  <-   strip_ott_ids (phylo_branched $ tip.label,  remove_underscores =   FALSE )   # remove ott ID from species name to match it to the data set  
   phylo_matrix  <-   vcv (phylo_branched,  cor =  T)   # Generate variance covariance matrix to correlate species relatedness   
    
   d  <-   as.data.frame (d) 
   d  <-   mutate (d,  search_string =   decapitalize (genus_species))   # Decapitalise species name to match it to the species name presented in 'taxa'  
   d  <-   left_join (d,  select (taxa, search_string, unique_name, ott_id),  by =   "search_string" )   # Join data sets  
    
   d  <-  d[d $ unique_name  %in%  phylo_branched $ tip.label, ]   # Check that species names are well matched with the phylogenetic tree     
 
 Plot the tree 
 Note that the tree presented in Figure 3 of the manuscript was
generated using the code in  Figures for publication  
      d.tree  <-   mutate (d,  tip.label =  unique_name)   # For the correspondence between tree and data  
   d.tree $ tip.label  =   as.factor (d.tree $ tip.label)   # Convert to factor  
   d.tree $ tip.label  <-   gsub ( "_" ,  " " , d.tree $ tip.label)   # remove underscores from data  
   phylo_branched $ tip.label  <-   strip_ott_ids (phylo_branched $ tip.label,  remove_underscores =   TRUE )   # remove underscores from tree  
    
    
    
    summary (d.tree $ tip.label)   # Need to remove duplicate species for building the tree     
  ##    Length     Class      Mode 
##      1089 character character  
      d.tree  <-  d.tree  %>%  
        group_by (tip.label)  %>%  
        mutate ( n_es =   sum ( n =   n ()))  %>%  
        ungroup ()   # Count the number of effect sizes for each species  
   d.tree  <-   mutate (d.tree,  log_es =   log10 (n_es))   # Log the number of effect sizes, if needed for visualisations  
   d.tree_plot  <-   distinct (d.tree, tip.label,  .keep_all =   TRUE )   # Only keep one row per species  
   d.tree_plot  <-   as.data.frame (d.tree_plot)   # Convert tibble to data frame  
   d.tree_plot $ phylum  <-   as.character (d.tree_plot $ phylum)   # Convert phylum to character  
   d.tree_plot  <-   select (d.tree_plot, tip.label, phylum, class, family, exp_design, 
       metric, n_es, log_es, dARR)   # important: select only the columns you need, otherwise this does not run  
    
   tree_for_plot  <-   drop.tip (phylo_branched,  setdiff (phylo_branched $ tip.label, phylo_branched $ tip.label))   # Make sure to keep all matching observations  
    
    
    
   design_dat  <-  d.tree  %>%  
        group_by (tip.label)  %>%  
        summarise ( initial =  brought_common_temp  ==   "no" ,  persistent =  brought_common_temp  ==  
            "yes" ,  CTmax =  metric  ==   "CTmax" ,  LT50 =  metric  ==   "LT50" )   # Summarise, for each species, whether they tested initial effects, persistent effects, CTmax, or LT50  
    
   design_dat  <-   distinct (design_dat)   # Only keep unique rows  
   design_dat $ initial  =   as.numeric (design_dat $ initial)   # convert TRUE/FALSE to binary values  
   design_dat $ persistent  =   as.numeric (design_dat $ persistent)   # convert TRUE/FALSE to binary values  
   design_dat $ CTmax  =   as.numeric (design_dat $ CTmax)   #convert TRUE/FALSE to binary values  
   design_dat $ LT50  =   as.numeric (design_dat $ LT50)   #convert TRUE/FALSE to binary values  
    
   design_dat  <-  design_dat  %>%  
        group_by (tip.label)  %>%  
        summarise ( initial =   sum (initial),  persistent =   sum (persistent),  CTmax =   sum (CTmax), 
            LT50 =   sum (LT50))   # calculate the sum for each species (i.e. if 1, the species has the given design or metric)     
    
    # In some cases, values were repeated, so need to replace '2' values by '1'  
   design_dat $ initial[design_dat $ initial  ==   "2" ]  <-   "1"  
   design_dat $ persistent[design_dat $ persistent  ==   "2" ]  <-   "1"  
   design_dat $ CTmax[design_dat $ CTmax  ==   "2" ]  <-   "1"  
    
   design_dat $ initial  =   as.factor (design_dat $ initial)   # convert back to factor for the plot  
   design_dat $ persistent  =   as.factor (design_dat $ persistent) 
   design_dat $ CTmax  =   as.factor (design_dat $ CTmax) 
   design_dat $ LT50  =   as.factor (design_dat $ LT50) 
    
    
   design_dat  <-   mutate (design_dat,  persistence =   ifelse (initial  ==   "1"   &  persistent  ==  
        "1" ,  "both" ,  ifelse (initial  ==   "1"   &  persistent  ==   "0" ,  "initial" ,  "persistent" )))   # If both initial and persistent, indicate 'both', if only initial, indicate 'initial', otherwise indicate 'persistent'  
    
   design_dat  <-   mutate (design_dat,  metrics =   ifelse (CTmax  ==   "1"   &  LT50  ==   "1" ,  "both" , 
        ifelse (CTmax  ==   "1"   &  LT50  ==   "0" ,  "CTmax" ,  "LT50" )))   # If both CTmax and LT50, indicate 'both', if only CTmax, indicate 'CTmax', otherwise indicate 'LT50'  
    
   d.tree_plot  <-   left_join (d.tree_plot, design_dat,  by =   "tip.label" )   # Join this information to the rest of the data we want to plot  
    
    
    
   p  <-   ggtree (tree_for_plot,  layout =   "circular" ,  lwd =   0.75 )   # Circular tree  
   p  <-  p  %<+%  d.tree_plot   # link plot to data  
   p2  <-  p  +   geom_fruit ( geom =  geom_tile,  mapping =   aes ( fill =  metrics),  width =   0.07 , 
        offset =   0.085 ,  col =   "gray30" )  +   scale_fill_manual ( values =   c ( "gray70" ,  "white" , 
        "black" ))   # Create tiles to indicate which metric was used for this species  
    
   p3  <-  p2  +   new_scale_fill ()  +   geom_fruit ( geom =  geom_tile,  mapping =   aes ( fill =  persistence), 
        offset =   0.1 ,  width =   0.07 ,  col =   "gray30" )  +   scale_fill_manual ( values =   c ( "#D95F02" , 
        "#7570B3" ,  "#1B9E77" ))   # Create tiles to indicate whether initial or persistent effects were assessed  
    
   d.tree_plot $ n_es  =   as.numeric (d.tree_plot $ n_es)   # Convert to numeric   
    
   p4  <-  p3  +   new_scale_fill ()  +   geom_fruit ( geom =  geom_bar,  mapping =   aes ( x =  n_es, 
        fill =  class),  stat =   "identity" ,  col =   "gray1" ,  orientation =   "y" ,  axis.params =   list ( axis =   "x" , 
        text.angle =   -  45 ,  hjust =   0 ,  text.size =   3 ),  border =   1.2 ,  grid.params =   list ( alpha =   0.35 ), 
        offset =   0.085 ,  pwidth =   0.55 ,  alpha =   0.8 )  +   scale_fill_manual ( values =   c ( "#00BBDB" , 
        "darkslategray4" ,  "darkorange" ,  "chartreuse" ,  "indianred2" ,  "lightsteelblue2" , 
        "darkorchid" ,  "darkseagreen1" ,  "deeppink" ,  "darkred" ,  "forestgreen" ,  "darkolivegreen2" , 
        "#39568CFF" ,  "darkgoldenrod2" ))   # Display number of effect sizes  
    
   p4    
   
 
 
 
  Account for treatments re-used in multiple
comparisons  
 In many cases, authors used multiple temperature treatments that can
be compared. In this case, we calculated our effect sizes on stepwise
comparisons (e.g., 15C-17C; 17C-19C, 19C-22C). Doing such creates a
source of non-independence that needs to be accounted for. In our case,
sampling errors from treatments involved in multiple comparisons were
correlated (using a conservative r = 0.5) with a variance covariance
matrix using the make_VCV_matrix function from the metaAidR package. 
       names (d)  <-   make.names ( names (d)) 
   d  <-   as.data.frame (d)   # Important: only works with data frames!   
   VCV_dARR  <-   make_VCV_matrix (d,  V =   "Var_dARR" ,  cluster =   "shared_trt_ID" ,  obs =   "es_ID" )    
 
 
  Sample sizes  
      d  %>%  
      summarise ( 
        
    # Number of studies, species and effect sizes  
        `  Studies  `   =   n_distinct (study_ID), 
        `  Species  `   =   n_distinct (genus_species),  
        `  Effect sizes  `   =   n_distinct (es_ID), 
        
    # Number of studies, species, and effect sizes for initial and persistent designs  
        `  Studies (initial)  `   =   n_distinct (study_ID[brought_common_temp ==  "no" ]), 
        `  Studies (Persistent)  `   =   n_distinct (study_ID[brought_common_temp ==  "yes" ]), 
        
        `  Species (initial)  `   =   n_distinct (genus_species[brought_common_temp ==  "no" ]), 
        `  Species (Persistent)  `   =   n_distinct (genus_species[brought_common_temp ==  "yes" ]),    
        
        `  Effect sizes (initial)  `   =   n_distinct (es_ID[brought_common_temp ==  "no" ]),   
        `  Effect sizes (Persistent)  `   =   n_distinct (es_ID[brought_common_temp ==  "yes" ]),   
       
    # Number of studies, species, and effect sizes for each habitat  
        `  Studies (Aquatic)  `   =   n_distinct (study_ID[habitat ==  "aquatic" ]), 
        `  Studies (Terrestrial)  `   =   n_distinct (study_ID[habitat ==  "terrestrial" ]), 
        
        `  Species (Aquatic)  `   =   n_distinct (genus_species[habitat ==  "aquatic" ]), 
        `  Species (Terrestrial)  `   =   n_distinct (genus_species[habitat ==  "terrestrial" ]),    
        
        `  Effect sizes (Aquatic)  `   =   n_distinct (es_ID[habitat ==  "aquatic" ]),   
        `  Effect sizes (Terrestrial)  `   =   n_distinct (es_ID[habitat ==  "terrestrial" ]),   
        
    # Number of studies, species, and effect sizes for each taxonomic group  
        `  Studies (Fishes)  `   =   n_distinct (study_ID[taxonomic_group ==  "fish" ]), 
        `  Studies (Reptiles)  `   =   n_distinct (study_ID[taxonomic_group ==  "reptile" ]), 
        `  Studies (Amphibians)  `   =   n_distinct (study_ID[taxonomic_group ==  "amphibian" ]), 
        `  Studies (Aquatic invertebrates)  `   =   n_distinct (study_ID[taxonomic_group ==  "aquatic invertebrate" ]), 
        `  Studies (Terrestrial invertebrates)  `   =   n_distinct (study_ID[taxonomic_group ==  "terrestrial invertebrate" ]), 
        `  Studies (Arthropods)  `   =   n_distinct (study_ID[phylum ==  "Arthropoda" ]), 
        `  Studies (Echinoderms)  `   =   n_distinct (study_ID[phylum ==  "Echinodermata" ]), 
        `  Studies (Molluscs)  `   =   n_distinct (study_ID[phylum ==  "Mollusca" ]), 
        
        `  Species (Fishes)  `   =   n_distinct (genus_species[taxonomic_group ==  "fish" ]), 
        `  Species (Reptiles)  `   =   n_distinct (genus_species[taxonomic_group ==  "reptile" ]), 
        `  Species (Amphibians)  `   =   n_distinct (genus_species[taxonomic_group ==  "amphibian" ]), 
        `  Species (Aquatic invertebrates)  `   =   n_distinct (genus_species[taxonomic_group ==  "aquatic invertebrate" ]), 
        `  Species (Terrestrial invertebrates)  `   =   n_distinct (genus_species[taxonomic_group ==  "terrestrial invertebrate" ]), 
        `  Species (Arthropods)  `   =   n_distinct (genus_species[phylum ==  "Arthropoda" ]), 
        `  Species (Echinoderms)  `   =   n_distinct (genus_species[phylum ==  "Echinodermata" ]), 
        `  Species (Molluscs)  `   =   n_distinct (genus_species[phylum ==  "Mollusca" ]),     
        
        `  Effect sizes (Fishes)  `   =   n_distinct (es_ID[taxonomic_group ==  "fish" ]), 
        `  Effect sizes (Reptiles)  `   =   n_distinct (es_ID[taxonomic_group ==  "reptile" ]), 
        `  Effect sizes (Amphibians)  `   =   n_distinct (es_ID[taxonomic_group ==  "amphibian" ]), 
        `  Effect sizes (Aquatic invertebrates)  `   =   n_distinct (es_ID[taxonomic_group ==  "aquatic invertebrate" ]), 
        `  Effect sizes (Terrestrial invertebrates)  `   =   n_distinct (es_ID[taxonomic_group ==  "terrestrial invertebrate" ]), 
        `  Effect sizes (Arthropods)  `   =   n_distinct (es_ID[phylum ==  "Arthropoda" ]), 
        `  Effect sizes (Echinoderms)  `   =   n_distinct (es_ID[phylum ==  "Echinodermata" ]), 
        `  Effect sizes (Molluscs)  `   =   n_distinct (es_ID[phylum ==  "Mollusca" ]), 
       
    # Number of studies, species and effect sizes for each experimental design  
        `  Studies (Design A)  `   =   n_distinct (study_ID[exp_design ==  "A" ]), 
        `  Studies (Design B)  `   =   n_distinct (study_ID[exp_design ==  "B" ]), 
        `  Studies (Design C)  `   =   n_distinct (study_ID[exp_design ==  "C" ]), 
        `  Studies (Design D)  `   =   n_distinct (study_ID[exp_design ==  "D" ]), 
        `  Studies (Design E)  `   =   n_distinct (study_ID[exp_design ==  "E" ]), 
        `  Studies (Design F)  `   =   n_distinct (study_ID[exp_design ==  "F" ]),   
        
        `  Species (Design A)  `   =   n_distinct (genus_species[exp_design ==  "A" ]), 
        `  Species (Design B)  `   =   n_distinct (genus_species[exp_design ==  "B" ]), 
        `  Species (Design C)  `   =   n_distinct (genus_species[exp_design ==  "C" ]), 
        `  Species (Design D)  `   =   n_distinct (genus_species[exp_design ==  "D" ]), 
        `  Species (Design E)  `   =   n_distinct (genus_species[exp_design ==  "E" ]), 
        `  Species (Design F)  `   =   n_distinct (genus_species[exp_design ==  "F" ]),       
        
        `  Effect sizes (Design A)  `   =   n_distinct (es_ID[exp_design ==  "A" ]), 
        `  Effect sizes (Design B)  `   =   n_distinct (es_ID[exp_design ==  "B" ]), 
        `  Effect sizes (Design C)  `   =   n_distinct (es_ID[exp_design ==  "C" ]), 
        `  Effect sizes (Design D)  `   =   n_distinct (es_ID[exp_design ==  "D" ]), 
        `  Effect sizes (Design E)  `   =   n_distinct (es_ID[exp_design ==  "E" ]), 
        `  Effect sizes (Design F)  `   =   n_distinct (es_ID[exp_design ==  "F" ]),       
        
    # Number of studies, species and effect sizes for each metric type  
        `  Studies (CTmax)  `   =   n_distinct (study_ID[metric ==  "CTmax" ]), 
        `  Studies (LT50)  `   =   n_distinct (study_ID[metric ==  "LT50" ]), 
    
        `  Species (CTmax)  `   =   n_distinct (genus_species[metric ==  "CTmax" ]), 
        `  Species (LT50)  `   =   n_distinct (genus_species[metric ==  "LT50" ]),    
        
        `  Effect sizes (CTmax)  `   =   n_distinct (es_ID[metric ==  "CTmax" ]), 
        `  Effect sizes (LT50)  `   =   n_distinct (es_ID[metric ==  "LT50" ]), 
    
        )  ->  table_sample_sizes 
    
   table_sample_sizes <-  t (table_sample_sizes) 
    colnames (table_sample_sizes) <-  "n (sample size)"  
    kable (table_sample_sizes)  %>%   kable_styling ( "striped" ,  position=  "center" )   # Stylise table      
 
 
 
 
 
 
n (sample size)
 
 
 
 
 
 
Studies
 
 
150
 
 
 
 
Species
 
 
138
 
 
 
 
Effect sizes
 
 
1089
 
 
 
 
Studies (initial)
 
 
126
 
 
 
 
Studies (Persistent)
 
 
26
 
 
 
 
Species (initial)
 
 
121
 
 
 
 
Species (Persistent)
 
 
23
 
 
 
 
Effect sizes (initial)
 
 
866
 
 
 
 
Effect sizes (Persistent)
 
 
223
 
 
 
 
Studies (Aquatic)
 
 
128
 
 
 
 
Studies (Terrestrial)
 
 
22
 
 
 
 
Species (Aquatic)
 
 
118
 
 
 
 
Species (Terrestrial)
 
 
20
 
 
 
 
Effect sizes (Aquatic)
 
 
929
 
 
 
 
Effect sizes (Terrestrial)
 
 
160
 
 
 
 
Studies (Fishes)
 
 
83
 
 
 
 
Studies (Reptiles)
 
 
9
 
 
 
 
Studies (Amphibians)
 
 
10
 
 
 
 
Studies (Aquatic invertebrates)
 
 
29
 
 
 
 
Studies (Terrestrial invertebrates)
 
 
19
 
 
 
 
Studies (Arthropods)
 
 
35
 
 
 
 
Studies (Echinoderms)
 
 
5
 
 
 
 
Studies (Molluscs)
 
 
8
 
 
 
 
Species (Fishes)
 
 
68
 
 
 
 
Species (Reptiles)
 
 
7
 
 
 
 
Species (Amphibians)
 
 
14
 
 
 
 
Species (Aquatic invertebrates)
 
 
31
 
 
 
 
Species (Terrestrial invertebrates)
 
 
18
 
 
 
 
Species (Arthropods)
 
 
30
 
 
 
 
Species (Echinoderms)
 
 
4
 
 
 
 
Species (Molluscs)
 
 
15
 
 
 
 
Effect sizes (Fishes)
 
 
623
 
 
 
 
Effect sizes (Reptiles)
 
 
27
 
 
 
 
Effect sizes (Amphibians)
 
 
71
 
 
 
 
Effect sizes (Aquatic invertebrates)
 
 
221
 
 
 
 
Effect sizes (Terrestrial invertebrates)
 
 
147
 
 
 
 
Effect sizes (Arthropods)
 
 
292
 
 
 
 
Effect sizes (Echinoderms)
 
 
13
 
 
 
 
Effect sizes (Molluscs)
 
 
63
 
 
 
 
Studies (Design A)
 
 
104
 
 
 
 
Studies (Design B)
 
 
5
 
 
 
 
Studies (Design C)
 
 
21
 
 
 
 
Studies (Design D)
 
 
7
 
 
 
 
Studies (Design E)
 
 
8
 
 
 
 
Studies (Design F)
 
 
12
 
 
 
 
Species (Design A)
 
 
109
 
 
 
 
Species (Design B)
 
 
5
 
 
 
 
Species (Design C)
 
 
13
 
 
 
 
Species (Design D)
 
 
6
 
 
 
 
Species (Design E)
 
 
7
 
 
 
 
Species (Design F)
 
 
11
 
 
 
 
Effect sizes (Design A)
 
 
700
 
 
 
 
Effect sizes (Design B)
 
 
20
 
 
 
 
Effect sizes (Design C)
 
 
146
 
 
 
 
Effect sizes (Design D)
 
 
92
 
 
 
 
Effect sizes (Design E)
 
 
76
 
 
 
 
Effect sizes (Design F)
 
 
55
 
 
 
 
Studies (CTmax)
 
 
132
 
 
 
 
Studies (LT50)
 
 
30
 
 
 
 
Species (CTmax)
 
 
118
 
 
 
 
Species (LT50)
 
 
35
 
 
 
 
Effect sizes (CTmax)
 
 
863
 
 
 
 
Effect sizes (LT50)
 
 
226
 
 
 
 
       kable (d  %>%   group_by (study_ID)  %>%   # Calculate the number of effect sizes per study  
                summarise ( n =   n_distinct (es_ID))  %>%    
                ungroup ()  %>%   
                summarise ( `  n effect sizes per study  `   =   mean (n),  
                           `  sd effect sizes per study  `  =   sd  (n),  
                           `  min effect sizes per study  `   =   min (n), 
                           `  max effect sizes per study  `  =   max (n)))  %>%   
    kable_styling ( "striped" ,  position=  "center" )     
 
 
 
 
n effect sizes per study
 
 
sd effect sizes per study
 
 
min effect sizes per study
 
 
max effect sizes per study
 
 
 
 
 
 
7.26
 
 
9.627582
 
 
1
 
 
80
 
 
 
 
       # Risk of bias   
    
   d.concern <-  mutate (d, 
                      Elliott=  ifelse (major_concerns ==  "Elliot's hybrid methodology. Sample size unclear."  | major_concerns ==  "Elliott's hybrid methodology"  | major_concerns ==  "Elliott's hybrid methodology."  | major_concerns ==  "Elliott's hybrid methodology.  SD missing for animals acclimated to 25C"  | major_concerns ==  "Elliott's hybrid methodology. SD missing for animals acclimated to 10C and 15C."  |  major_concerns ==  "Elliott's hybrid methodology. SD missing for animals acclimated to 15C"  |  major_concerns ==  "Elliott's hybrid methodology. SD missing for animals acclimated to 15C."  | major_concerns ==  "Elliott's hybrid methodology. SD missing for animals acclimated to 25C."  | major_concerns ==  "Elliott's hybrid methodology. SD missing for animals acclimated to 27C."  | major_concerns ==  "Elliott's hybrid methodology. SD missing for animals acclimated to 5C."  | major_concerns ==  "Elliott's hybrid methodology. SD missing."  | major_concerns ==  "hybrid between LT50 and CTmax" , study_ID,  NA ), 
                      
                      Missing_est_SD=  ifelse (major_concerns ==  "Elliott's hybrid methodology.  SD missing for animals acclimated to 25C"  | major_concerns ==  "Elliott's hybrid methodology. SD missing for animals acclimated to 10C and 15C."  |  major_concerns ==  "Elliott's hybrid methodology. SD missing for animals acclimated to 15C"  |  major_concerns ==  "Elliott's hybrid methodology. SD missing for animals acclimated to 15C."  | major_concerns ==  "Elliott's hybrid methodology. SD missing for animals acclimated to 25C."  | major_concerns ==  "Elliott's hybrid methodology. SD missing for animals acclimated to 27C."  | major_concerns ==  "Elliott's hybrid methodology. SD missing for animals acclimated to 5C."  | major_concerns ==  "Elliott's hybrid methodology. SD missing."  | major_concerns ==  "Missing sd."  | major_concerns ==  "SD missing"  | major_concerns ==  "SD missing."  | major_concerns ==  "SD possibly under-estimated."  | major_concerns ==  "SE was taken instead of the SD reported."  | major_concerns ==  "SE was taken instead of the SD reported."  | major_concerns ==  "Standard deviation calculated as 0 so it was assigned as   \"  NA  \"  ."  | major_concerns ==  "Standard deviation was reported as 0 so it was assigned as   \"  NA  \"  "  | major_concerns ==  "Standard error missing."  | major_concerns ==  "Standard error probably under- or over-estimated because the number of animals at each test temperatures was not clearly stated and is used for calculating LT50."  | imputed ==  "yes" , study_ID,  NA ),  
                      Unclear_N=  ifelse (major_concerns ==  "Elliot's hybrid methodology. Sample size unclear."  | major_concerns ==  "N missing" , study_ID,  NA ), 
                      Tolerance_landscape=  ifelse (major_concerns ==  "The CTmax was estimated from death times at different temperatures (thermal tolerance landscapes)."  | major_concerns ==  "The CTmax was estimated from death times at different temperatures (thermal tolerance landscapes).  \\  " , study_ID,  NA ), 
                      Data_similar=  ifelse (major_concerns ==  "Data very similar to study_ID 69." , study_ID,  NA ), 
                      High_SE=  ifelse (major_concerns ==  "Very high standard error because low sample size (4 test temperatures) in the 28C acclimated group." , study_ID,  NA ), 
                      Short_test=  ifelse (major_concerns ==  "Very short set_time at the test temperatures." , study_ID,  NA ) 
                      
                     ) 
    
    
      
      
   table_bias <-  d.concern  %>%   
      summarise ( 
      `  Studies (data similar)  `   =   n_distinct (Data_similar,  na.rm= T), 
      `  Studies (Elliott's hybrid)  `   =   n_distinct (Elliott,  na.rm= T), 
      `  Studies (Missing or estimated SD)  `  =   n_distinct (Missing_est_SD,  na.rm= T), 
      `  Studies (Unclear sample size)  `  =   n_distinct (Unclear_N,  na.rm= T), 
      `  Studies (Tolerance landscape)  `  =   n_distinct (Tolerance_landscape,  na.rm= T), 
      `  Studies (High standard error)  `  =   n_distinct (High_SE,  na.rm= T), 
      `  Studies (Short test time)  `  =   n_distinct (Short_test,  na.rm= T) 
   ) 
    
   table_bias <-  t (table_bias) 
    colnames (table_bias) <-  "n (sample size)"  
    kable (table_bias)  %>%   kable_styling ( "striped" ,  position=  "center" )     
 
 
 
 
 
 
n (sample size)
 
 
 
 
 
 
Studies (data similar)
 
 
1
 
 
 
 
Studies (Elliott’s hybrid)
 
 
4
 
 
 
 
Studies (Missing or estimated SD)
 
 
16
 
 
 
 
Studies (Unclear sample size)
 
 
3
 
 
 
 
Studies (Tolerance landscape)
 
 
4
 
 
 
 
Studies (High standard error)
 
 
1
 
 
 
 
Studies (Short test time)
 
 
1
 
 
 
 
 
 
  Save processed data  
      d $ phylogeny  <-  d $ unique_name   # Rename 'unique_name' to 'phylogeny' for the models  
    save (VCV_dARR, phylo_matrix,  file =   here ( "RData" ,  "Var_and_phylo_matrices.RData" )) 
    
    write_csv (d,  file =   here ( "data" ,  "Processed_data.csv" )) 
    
    
    load ( here ( "Rdata" ,  "Var_and_phylo_matrices.RData" )) 
   d  <-   read_csv ( "data/Processed_data.csv" )    
 
 
  Choose random effect structure  
 Study ID and species ID were not included in the same model because
their levels overlapped too much. We kept “species ID” because it allows
to decompose phylogenetic and non-phylogenetic species effects, as
recommended in Cinar et al. 2021 
 Because population_ID was explaining virtually no variance, they were
removed from further models. 
 Cohort_ID and shared_treatment_ID were not included as random effects
because the non-independence arising from shared measurements and
treatments reused in multiple comparisons were already accounted for in
the calculation of effect sizes (see equation 4 and 5) and associated
sampling variances (see calculation of VCV_dARR). 
 Note that all models are relatively long to run, but they are saved
in the folder  models  and code for loading each model is
presented at the end of each section in the .Rmd file. 
      d <-  as.data.frame (d)  #   
   random_effect_mod <-   rma.mv (dARR ~  1 ,  # Intercept only  
                               V= VCV_dARR,   
                               method=  "REML" ,  # Restricted maximum likelihood  
                               test=  "t" ,  # Test statistics using a t distribution  
                               dfs=  "contain" ,  # Contain degrees of freedom (recommended)  
                               random=  list ( ~  1  | species_ID, 
                                           ~  1  | population_ID,  
                                           ~  1  | phylogeny, 
                                           ~  1  | es_ID), 
                               R =   list ( phylogeny =  phylo_matrix),  # Assign the phylogenetic relatedness matrix to "phylogeny"  
                               data= d) 
    
    summary (random_effect_mod)  # Summary of the model     
  ## 
## Multivariate Meta-Analysis Model (k = 1089; method: REML)
## 
##    logLik   Deviance        AIC        BIC       AICc 
## -226.3154   452.6309   462.6309   487.5913   462.6863   
## 
## Variance Components:
## 
##             estim    sqrt  nlvls  fixed         factor    R 
## sigma^2.1  0.0095  0.0974    138     no     species_ID   no 
## sigma^2.2  0.0000  0.0000    239     no  population_ID   no 
## sigma^2.3  0.0249  0.1578    138     no      phylogeny  yes 
## sigma^2.4  0.0605  0.2460   1089     no          es_ID   no 
## 
## Test for Heterogeneity:
## Q(df = 1088) = 63442.0311, p-val < .0001
## 
## Model Results:
## 
## estimate      se    tval   df    pval   ci.lb   ci.ub 
##   0.1898  0.0882  2.1525  137  0.0331  0.0154  0.3641  * 
## 
## ---
## Signif. codes:  0 '***' 0.001 '**' 0.01 '*' 0.05 '.' 0.1 ' ' 1  
 
 
  Custom functions  
 
 Run models 
      run.model <-  function (data,formula){ 
     data <-  as.data.frame (data)  # convert data set into a data frame to calculate VCV matrix   
     VCV <-  make_VCV_matrix (data,  V=  "Var_dARR" ,  cluster=  "shared_trt_ID" ,  obs=  "es_ID" )  # create VCV matrix for the specified data  
      
   mod <-    rma.mv ( yi= dARR,  
             V= VCV,  # run the model, as described earlier  
             mods=  formula, 
             method=  "REML" , 
             test=  "t" , 
             dfs=  "contain" , 
             random=  list ( ~  1  | species_ID, 
                         ~  1  | phylogeny, 
                         ~  1  | es_ID), 
             R =   list ( phylogeny =  phylo_matrix), 
             data= data, 
             sparse=  TRUE )  # To reduce model run time  
    return (mod) 
   }    
 
 
 Plot orchaRd plots 
      my.orchard <-   function  (object,  mod =   "1" , group, data, xlab,  N =   "none" ,  
        alpha =   0.5 ,  angle =   90 ,  cb =   FALSE ,  k =   TRUE ,  g =   TRUE ,  
        trunk.size =   7 ,  branch.size =   2 ,  twig.size =   0.8 , whisker,  transfm =   c ( "none" ,  # increased point size, branch size, and added a whisker argument  
            "tanh" ),  condition.lab =   "Condition" ,  legend.pos =   "bottom.right" ,  k.pos =   c ( "right" ,  
            "left" ))  
   { 
       transfm  <-   match.arg (transfm) 
        if  ( any ( class (object)  %in%   c ( "rma.mv" ,  "rma" ))) { 
            if  (mod  !=   "1" ) { 
               results  <-  orchaRd ::  mod_results (object, mod, group,  
                   data) 
           } 
            else  { 
               results  <-  orchaRd ::  mod_results (object,  mod =   "1" ,  
                   group, data) 
           } 
       } 
        if  ( any ( class (object)  %in%   c ( "orchard" ))) { 
           results  <-  object 
       } 
       mod_table  <-  results $ mod_table 
       data  <-  results $ data 
       data $ moderator  <-   factor (data $ moderator,  levels =  mod_table $ name,  
            labels =  mod_table $ name) 
       data $ scale  <-  ( 1  /  sqrt (data[,  "vi" ])) 
       legend  <-   "Precision (1/SE)"  
        if  ( any (N  !=   "none" )) { 
           data $ scale  <-  N 
           legend  <-   "Sample Size (N)"  
       } 
        if  (transfm  ==   "tanh" ) { 
           cols  <-   sapply (mod_table, is.numeric) 
           mod_table[, cols]  <-   Zr_to_r (mod_table[, cols]) 
           data $ yi  <-   Zr_to_r (data $ yi) 
           label  <-  xlab 
       } 
        else  { 
           label  <-  xlab 
       } 
       mod_table $ K  <-   as.vector ( by (data, data[,  "moderator" ],  
            function (x)  length (x[,  "yi" ]))) 
       mod_table $ g  <-   as.vector ( num_studies (data, moderator, stdy)[,  
            2 ]) 
       group_no  <-   length ( unique (mod_table[,  "name" ])) 
       cbpl  <-   c ( "#88CCEE" ,  "#CC6677" ,  "#DDCC77" ,  
            "#117733" ,  "#332288" ,  "#AA4499" ,  "#44AA99" ,  
            "#999933" ,  "#882255" ,  "#661100" ,  "#6699CC" ,  
            "#888888" ,  "#E69F00" ,  "#56B4E9" ,  "#009E73" ,  
            "#F0E442" ,  "#0072B2" ,  "#D55E00" ,  "#CC79A7" ,  
            "#999999" ) 
        if  ( names (mod_table)[ 2 ]  ==   "condition" ) { 
           condition_no  <-   length ( unique (mod_table[,  "condition" ])) 
           plot  <-  ggplot2 ::  ggplot ()  +  ggbeeswarm ::  geom_quasirandom ( data =  data,  
               ggplot2 ::  aes ( y =  yi,  x =  moderator,  size =  scale,  
                    colour =  moderator),  alpha =  alpha)  +  ggplot2 ::  geom_hline ( yintercept =   0 ,  
                linetype =   2 ,  colour =   "black" ,  alpha =  alpha)  +   
               ggplot2 ::  geom_linerange ( data =  mod_table, ggplot2 ::  aes ( x =  name,  
                    ymin =  lowerCL,  ymax =  upperCL),  size =  branch.size,  
                    position =  ggplot2 ::  position_dodge2 ( width =   0.3 ))  +   
               ggplot2 ::  geom_pointrange ( data =  mod_table, ggplot2 ::  aes ( y =  estimate,  
                    x =  name,  ymin =  lowerPR,  ymax =  upperPR,  shape =   as.factor (condition),  
                    fill =  name),  size =  twig.size,  position =  ggplot2 ::  position_dodge2 ( width =   0.3 ),  
                    fatten =  trunk.size)  +  ggplot2 ::  scale_shape_manual ( values =   20   +   
               ( 1  : condition_no))  +  ggplot2 ::  coord_flip ()  +  ggplot2 ::  theme_bw ()  +   
               ggplot2 ::  guides ( fill =   "none" ,  colour =   "none" )  +   
               ggplot2 ::  theme ( legend.position =   c ( 0 ,  1 ),  legend.justification =   c ( 0 ,  
                    1 ))  +  ggplot2 ::  theme ( legend.title =  ggplot2 ::  element_text ( size =   9 ))  +   
               ggplot2 ::  theme ( legend.direction =   "horizontal" )  +   
               ggplot2 ::  theme ( legend.background =  ggplot2 ::  element_blank ())  +   
               ggplot2 ::  labs ( y =  label,  x =   "" ,  size =  legend)  +   
               ggplot2 ::  labs ( shape =  condition.lab)  +  ggplot2 ::  theme ( axis.text.y =  ggplot2 ::  element_text ( size =   10 ,  
                colour =   "black" ,  hjust =   0.5 ,  angle =  angle)) 
       } 
        else  { 
           plot  <-  ggplot2 ::  ggplot ()  +  ggbeeswarm ::  geom_quasirandom ( data =  data,  
               ggplot2 ::  aes ( y =  yi,  x =  moderator,  size =  scale,  
                    fill =  moderator),  alpha =  alpha,  width=  0.4 ,  pch=  21 ,  stroke=  1.1 ,  col=  "black" )  +   # Change point shape (21, with black borders)  
               ggplot2 ::  geom_hline ( yintercept =   0 ,  
                linetype =   2 ,  colour =   "black" ,  alpha =   0.3 ,  lwd=  1.3 )  +   # Change thickness 0 line  
               ggplot2 ::  geom_errorbar ( data =  mod_table, ggplot2 ::  aes ( x =  name,  
                    ymin =  lowerCL,  ymax =  upperCL),  size =  branch.size,  width=  whisker)  +   # Added variable whisker size  
               ggplot2 ::  geom_pointrange ( data =  mod_table, ggplot2 ::  aes ( y =  estimate,  
                    x =  name,  ymin =  lowerPR,  ymax =  upperPR,  fill =  name),  
                    size =  twig.size,  fatten =  trunk.size,  shape =   23 ,  stroke=  2.2 )  +   # Change point shape  
                scale_size_continuous ( range =   c ( 1 ,  14 )) +   # change point scaling  
               ggplot2 ::  coord_flip ()  +   
               ggplot2 ::  theme_bw ()  +   
               ggplot2 ::  guides ( fill =   "none" ,  colour =   "none" )  +   
               ggplot2 ::  theme ( text=  element_text ( size=  26 ,  colour=  "black" )) +   # Change font size  
               ggplot2 ::  theme ( legend.title =  ggplot2 ::  element_text ( size =   16 ))  +   # Increased font legend title  
               ggplot2 ::  theme ( legend.text =  ggplot2 ::  element_text ( size =   14 ))  +  
               ggplot2 ::  theme ( legend.direction =   "horizontal" )  +   
               ggplot2 ::  theme ( legend.background =  ggplot2 ::  element_blank ())  +   
               ggplot2 ::  labs ( y =  label,  x =   "" ,  size =  legend)  +   
               ggplot2 ::  theme ( axis.text.y =  ggplot2 ::  element_text ( size =   20 ,  
                    colour =   "black" ,  hjust =   0.5 ,  angle =  angle))  +   # Increased size title axis label  
               ggplot2 ::  theme ( axis.text.x =  ggplot2 ::  element_text ( size =   20 ))  +   # Increase size axis ticks  
               ggplot2 ::  theme ( panel.border =   element_rect ( colour =   "black" ,  fill=  NA ,  size=  1.3 )) 
                
       } 
        if  (legend.pos  ==   "bottom.right" ) { 
           plot  <-  plot  +  ggplot2 ::  theme ( legend.position =   c ( 1 ,  
                0 ),  legend.justification =   c ( 1 ,  0 )) 
       } 
        else   if  (legend.pos  ==   "bottom.left" ) { 
           plot  <-  plot  +  ggplot2 ::  theme ( legend.position =   c ( 0 ,  
                0 ),  legend.justification =   c ( 0 ,  0 )) 
       } 
        else   if  (legend.pos  ==   "top.right" ) { 
           plot  <-  plot  +  ggplot2 ::  theme ( legend.position =   c ( 1 ,  
                1 ),  legend.justification =   c ( 1 ,  1 )) 
       } 
        else   if  (legend.pos  ==   "top.left" ) { 
           plot  <-  plot  +  ggplot2 ::  theme ( legend.position =   c ( 0 ,  
                1 ),  legend.justification =   c ( 0 ,  1 )) 
       } 
        else   if  (legend.pos  ==   "top.out" ) { 
           plot  <-  plot  +  ggplot2 ::  theme ( legend.position =   "top" ) 
       } 
        else   if  (legend.pos  ==   "bottom.out" ) { 
           plot  <-  plot  +  ggplot2 ::  theme ( legend.position =   "bottom" ) 
       } 
        if  (cb  ==   TRUE ) { 
           plot  <-  plot  +  ggplot2 ::  scale_fill_manual ( values =  cbpl)  +   
               ggplot2 ::  scale_colour_manual ( values =  cbpl) 
       } 
        if  (k  ==   TRUE   &&  g  ==   FALSE   &&  k.pos  ==   "right" ) { 
           plot  <-  plot  +  ggplot2 ::  annotate ( "text" ,  y =  ( max (data $ yi)  +   
               ( max (data $ yi)  *   0.1 )),  x =  ( seq ( 1 , group_no,  1 )  +   
                0.3 ),  label =   paste ( "italic(k)==" , mod_table $ K[ 1  : group_no]),  # Size changed to 5.5  
                parse =   TRUE ,  hjust =   "right" ,  size =   6.5 ) 
       } 
        else   if  (k  ==   TRUE   &&  g  ==   FALSE   &&  k.pos  ==   "left" ) { 
           plot  <-  plot  +  ggplot2 ::  annotate ( "text" ,  y =  ( min (data $ yi)  +   
               ( min (data $ yi)  *   0.1 )),  x =  ( seq ( 1 , group_no,  1 )  +   
                0.3 ),  label =   paste ( "italic(k)==" , mod_table $ K[ 1  : group_no]),  
                parse =   TRUE ,  hjust =   "left" ,  size =   6.5 )  # Size changed to 5.5  
       } 
        else   if  (k  ==   TRUE   &&  g  ==   TRUE   &&  k.pos  ==   "right" ) { 
           plot  <-  plot  +  ggplot2 ::  annotate ( "text" ,  y =  ( max (data $ yi)  +   
               ( max (data $ yi)  *   0.1 )),  x =  ( seq ( 1 , group_no,  1 )  +   
                0.3 ),  label =   paste ( "italic(k)==" , mod_table $ K[ 1  : group_no],  
                " (" , mod_table $ g[ 1  : group_no],  ")" ),  
                parse =   TRUE ,  hjust =   "right" ,  size =   6.5 )  # Size changed to 5.5  
       } 
        else   if  (k  ==   TRUE   &&  g  ==   TRUE   &&  k.pos  ==   "left" ) { 
           plot  <-  plot  +  ggplot2 ::  annotate ( "text" ,  y =  ( min (data $ yi)  +   
               ( min (data $ yi)  *   0.1 )),  x =  ( seq ( 1 , group_no,  1 )  +   
                0.3 ),  label =   paste ( "italic(k)==" , mod_table $ K[ 1  : group_no],  
                " (" , mod_table $ g[ 1  : group_no],  ")" ),  
                parse =   TRUE ,  hjust =   "left" ,  size =   6.5 )  # Size changed to 5.5  
       } 
        return (plot) 
   }    
 
 
 Plot continuous moderator 
      plot_continuous <-  function (data, model, moderator, xlab){ 
    
   pred <-  predict.rma (model)  # Generate predictions from the model  
    
   data <-  data  %>%   mutate ( fit= pred $ pred,   # Mutate predicted values and prediction/confidence intervals to the dataset  
                   ci.lb= pred $ ci.lb, 
                   ci.ub= pred $ ci.ub, 
                   pr.lb= pred $ cr.lb, 
                   pr.ub= pred $ cr.ub) 
      
   plot <-  ggplot ( data= data,  aes ( x =  moderator,  y =  dARR))  +   # Plot the results  
         geom_ribbon ( aes ( ymin =  pr.lb,  ymax =  pr.ub,  color =   NULL ),  alpha =  . 1 )  +   # Shaded area for prediction intervals  
         geom_ribbon ( aes ( ymin =  ci.lb,  ymax =  ci.ub,  color =   NULL ),  alpha =  . 3 )  +   # Shaded area for confidence intervals  
         geom_point ( aes ( size= precision),  shape=  21 ,  alpha=  0.7 ,  fill=  "sienna1" ,  col=  "gray25" , stroke=  1 )  +   # Plot scaled data points  
         geom_line ( aes ( y =  fit),  size =   1.5 ) +    # Display predicted regression line  
      labs ( x =  xlab,  y =   "dARR" ,  size =   "Precison (1/SE)" )  +  
      theme_bw ()  +   # Black and white theme  
      scale_size_continuous ( range=  c ( 1 , 12 ))  +   # CHange extent of point scaling  
      geom_hline ( yintercept =   0 , linetype =   2 ,  colour =   "black" , alpha=  0.5 ) +     # horizontal line at dARR = 0  
      theme ( text =   element_text ( size =   18 ,  colour =   "black" ,  hjust =   0.5 ),  # change font sizes and legend position  
              legend.text=  element_text ( size=  14 ), 
              legend.position=  c ( 0 , 0 ),  
              legend.justification =   c ( 0 , 0 ), 
              legend.background =   element_blank (),  
              legend.direction=  "horizontal" , 
              legend.title =   element_text ( size=  15 ),  
              panel.border=  element_rect ( colour=  "black" ,  fill=  NA ,  size=  1.2 )) 
    return (plot) 
   }    
 
 
 
  Intercept Meta-analytic model  
      int_model  <-   run.model (d,  ~  1 ) 
    
    summary (int_model)    
  ## 
## Multivariate Meta-Analysis Model (k = 1089; method: REML)
## 
##    logLik   Deviance        AIC        BIC       AICc  ​ 
## -226.3154   452.6309   460.6309   480.5992   460.6678   
## 
## Variance Components:
## 
##             estim    sqrt  nlvls  fixed      factor    R 
## sigma^2.1  0.0095  0.0974    138     no  species_ID   no 
## sigma^2.2  0.0249  0.1578    138     no   phylogeny  yes 
## sigma^2.3  0.0605  0.2460   1089     no       es_ID   no 
## 
## Test for Heterogeneity:
## Q(df = 1088) = 63442.0311, p-val < .0001
## 
## Model Results:
## 
## estimate      se    tval   df    pval   ci.lb   ci.ub   ​ 
##   0.1898  0.0882  2.1525  137  0.0331  0.0154  0.3641  * 
## 
## ---
## Signif. codes:  0 '***' 0.001 '**' 0.01 '*' 0.05 '.' 0.1 ' ' 1  
       mod_results (int_model,  mod =   "1" ,  data =  d,  group =   "species_ID" )   # For prediction intervals     
  ##      name estimate lowerCL upperCL   lowerPR  upperPR
## 1 Intrcpt        0       0       0 -0.609065 0.609065  
       i2_ml (int_model)   # Estimate heterogeneity     
  ##      I2_Total I2_species_ID  I2_phylogeny      I2_es_ID 
##     99.481565      9.951012     26.095005     63.435548  
       my.orchard (int_model,  mod =   "1" ,  xlab =   "dARR" ,  alpha =   0.1 ,  data =  d,  group =   "species_ID" , 
        whisker =   0.04 )   # Display orchard plot     
   
 
 
  Single moderator metaregressions  
 
  Habitat  
 
 Individual coefficients 
      mod.habitat  <-   run.model (d,  ~ habitat  -   1 ) 
    summary (mod.habitat)    
  ## 
## Multivariate Meta-Analysis Model (k = 1089; method: REML)
## 
##    logLik   Deviance        AIC        BIC       AICc  ​ 
## -223.0240   446.0480   456.0480   481.0039   456.1035   
## 
## Variance Components:
## 
##             estim    sqrt  nlvls  fixed      factor    R 
## sigma^2.1  0.0099  0.0994    138     no  species_ID   no 
## sigma^2.2  0.0134  0.1156    138     no   phylogeny  yes 
## sigma^2.3  0.0606  0.2461   1089     no       es_ID   no 
## 
## Test for Residual Heterogeneity:
## QE(df = 1087) = 56246.7341, p-val < .0001
## 
## Test of Moderators (coefficients 1:2):
## F(df1 = 2, df2 = 136) = 7.2679, p-val = 0.0010
## 
## Model Results:
## 
##                     estimate      se    tval   df    pval    ci.lb   ci.ub    ​ 
## habitataquatic        0.2087  0.0664  3.1422  136  0.0021   0.0773  0.3400  ** 
## habitatterrestrial    0.0672  0.0817  0.8216  136  0.4127  -0.0945  0.2288     
## 
## ---
## Signif. codes:  0 '***' 0.001 '**' 0.01 '*' 0.05 '.' 0.1 ' ' 1  
       mod_results (mod.habitat,  mod =   "habitat" ,  data =  d,  group =   "species_ID" )    
  ##          name   estimate     lowerCL   upperCL    lowerPR   upperPR
## 1     Aquatic 0.20867269  0.07734442 0.3400010 -0.3786494 0.7959948
## 2 Terrestrial 0.06715867 -0.09448001 0.2287973 -0.5276751 0.6619924  
       r2_ml (mod.habitat)   # Calculate R2 (percentage of explained variation). Note that, throughout the manuscript, we report the marginal R2, which is the percentage of variation explained by the fixed effects     
  ##    R2_marginal R2_conditional 
##     0.02910927     0.29842002  
       my.orchard (mod.habitat,  mod =   "habitat" ,  xlab =   "dARR" ,  alpha =   0.1 ,  data =  d,  group =   "species_ID" , 
        whisker =   0.07 )    
   
 
 
 Contrasts 
      mod.habitat_cont  <-   run.model (d,  ~ habitat)   # Keep intercept for contrasts  
    summary (mod.habitat_cont)    
  ## 
## Multivariate Meta-Analysis Model (k = 1089; method: REML)
## 
##    logLik   Deviance        AIC        BIC       AICc  ​ 
## -223.0240   446.0480   456.0480   481.0039   456.1035   
## 
## Variance Components:
## 
##             estim    sqrt  nlvls  fixed      factor    R 
## sigma^2.1  0.0099  0.0994    138     no  species_ID   no 
## sigma^2.2  0.0134  0.1156    138     no   phylogeny  yes 
## sigma^2.3  0.0606  0.2461   1089     no       es_ID   no 
## 
## Test for Residual Heterogeneity:
## QE(df = 1087) = 56246.7341, p-val < .0001
## 
## Test of Moderators (coefficient 2):
## F(df1 = 1, df2 = 136) = 6.3394, p-val = 0.0130
## 
## Model Results:
## 
##                     estimate      se     tval   df    pval    ci.lb    ci.ub​ 
## intrcpt               0.2087  0.0664   3.1422  136  0.0021   0.0773   0.3400 
## habitatterrestrial   -0.1415  0.0562  -2.5178  136  0.0130  -0.2527  -0.0304 
##  
## intrcpt             ** 
## habitatterrestrial   * 
## 
## ---
## Signif. codes:  0 '***' 0.001 '**' 0.01 '*' 0.05 '.' 0.1 ' ' 1  
 
 
 Heteroscedasticity check 
 Here, we can clearly see that the plastic responses of aquatic
animals are more variable than the ones of terrestrial animals. 
       qplot ( y =   sqrt ( residuals (mod.habitat) ^  2 ),  x =   fitted (mod.habitat))  +   # plot sqrt(residuals^2) against fitted values  
          geom_point ()  +  
          geom_smooth ( method =   "lm" )  +   # method ="lm" to generate a straight line   
          geom_hline ( yintercept =   0 ,  colour=  "red" )   # Seems heteroscedastic     
   
 
 
 Individual coefficients (with heteroscedasticity) 
 The  rma.mv  function has options to model
heteroscedasticity. In our case, most of the heterogeneity is explained
by residual variation (es_ID; within-species variation). Below, we model
the heteroscedasticity at effect size level and compare AIC values to
determine whether accounting for variance differences between habitats
improves model’s fit. 
       ##### Heteroscedasticity modeled at the effect size level  
   mod.habitat_het  <-    rma.mv ( yi= dARR,  
                               V= VCV_dARR,  # run the model, as described earlier  
                               mods=   ~ habitat -1 , 
                               method=  "REML" , 
                               test=  "t" , 
                               dfs=  "contain" , 
                               random=  list ( ~  1  | species_ID, 
                                           ~  1  | phylogeny, 
                                           ~ habitat | es_ID),  # To model heteroscedasticity at the effect size level, for each habitat  
                               struct=  "HCS" ,  # heteroscedastic compound symmetric structure  
                               rho=  0 ,  # Correlation of 0 for the HCS structure  
                               R =   list ( phylogeny =  phylo_matrix), 
                               data= d, 
                               sparse=  TRUE ) 
    
    AICc (mod.habitat)    
  ## [1] 456.1034  
       AICc (mod.habitat_het)  # Best fit     
  ## [1] 319.5823  
       summary (mod.habitat_het)    
  ## 
## Multivariate Meta-Analysis Model (k = 1089; method: REML)
## 
##    logLik   Deviance        AIC        BIC       AICc  ​ 
## -153.7523   307.5047   319.5047   349.4517   319.5824   
## 
## Variance Components:
## 
##             estim    sqrt  nlvls  fixed      factor    R 
## sigma^2.1  0.0090  0.0948    138     no  species_ID   no 
## sigma^2.2  0.0129  0.1136    138     no   phylogeny  yes 
## 
## outer factor: es_ID   (nlvls = 1089)
## inner factor: habitat (nlvls = 2)
## 
##             estim    sqrt  k.lvl  fixed        level 
## tau^2.1    0.0716  0.2677    929     no      aquatic 
## tau^2.2    0.0082  0.0907    160     no  terrestrial 
## rho        0.0000                   yes              
## 
## Test for Residual Heterogeneity:
## QE(df = 1087) = 56246.7341, p-val < .0001
## 
## Test of Moderators (coefficients 1:2):
## F(df1 = 2, df2 = 136) = 8.5055, p-val = 0.0003
## 
## Model Results:
## 
##                     estimate      se    tval   df    pval    ci.lb   ci.ub    ​ 
## habitataquatic        0.2087  0.0655  3.1856  136  0.0018   0.0792  0.3383  ** 
## habitatterrestrial    0.0595  0.0762  0.7814  136  0.4359  -0.0911  0.2102     
## 
## ---
## Signif. codes:  0 '***' 0.001 '**' 0.01 '*' 0.05 '.' 0.1 ' ' 1  
       mod_results (mod.habitat_het,  mod=  "habitat" , data= d,  group=  "species_ID" )     
  ##          name   estimate     lowerCL   upperCL    lowerPR   upperPR
## 1     Aquatic 0.20872360  0.07915124 0.3382960 -0.4098660 0.8273132
## 2 Terrestrial 0.05952518 -0.09112232 0.2101727 -0.3152993 0.4343497  
       r2_ml (mod.habitat_het)    
  ##    R2_marginal R2_conditional 
##      0.1130771      0.4772616  
       my.orchard (mod.habitat_het,  mod=  "habitat" ,  xlab=  "dARR" ,  alpha=  0.1 ,  data= d,  group=  "species_ID" ,  whisker=  0.07 )    
   
 
 
 Contrasts (with heteroscedasticity) 
       ### Contrasts with heteroscedasticity   
   mod.habitat_het_cont  <-    rma.mv ( yi= dARR,  
                               V= VCV_dARR,  
                               mods=   ~ habitat,  # Keep intercept  
                               method=  "REML" , 
                               test=  "t" , 
                               dfs=  "contain" , 
                               random=  list ( ~  1  | species_ID, 
                                           ~  1  | phylogeny, 
                                           ~ habitat | es_ID), 
                               struct=  "HCS" ,  
                               rho=  0 ,  
                               R =   list ( phylogeny =  phylo_matrix), 
                               data= d, 
                               sparse=  TRUE ) 
    
    summary (mod.habitat_het_cont)    
  ## 
## Multivariate Meta-Analysis Model (k = 1089; method: REML)
## 
##    logLik   Deviance        AIC        BIC       AICc  ​ 
## -153.7523   307.5047   319.5047   349.4517   319.5824   
## 
## Variance Components:
## 
##             estim    sqrt  nlvls  fixed      factor    R 
## sigma^2.1  0.0090  0.0948    138     no  species_ID   no 
## sigma^2.2  0.0129  0.1136    138     no   phylogeny  yes 
## 
## outer factor: es_ID   (nlvls = 1089)
## inner factor: habitat (nlvls = 2)
## 
##             estim    sqrt  k.lvl  fixed        level 
## tau^2.1    0.0716  0.2677    929     no      aquatic 
## tau^2.2    0.0082  0.0907    160     no  terrestrial 
## rho        0.0000                   yes              
## 
## Test for Residual Heterogeneity:
## QE(df = 1087) = 56246.7341, p-val < .0001
## 
## Test of Moderators (coefficient 2):
## F(df1 = 1, df2 = 136) = 9.2383, p-val = 0.0028
## 
## Model Results:
## 
##                     estimate      se     tval   df    pval    ci.lb    ci.ub​ 
## intrcpt               0.2087  0.0655   3.1856  136  0.0018   0.0792   0.3383 
## habitatterrestrial   -0.1492  0.0491  -3.0394  136  0.0028  -0.2463  -0.0521 
##  
## intrcpt             ** 
## habitatterrestrial  ** 
## 
## ---
## Signif. codes:  0 '***' 0.001 '**' 0.01 '*' 0.05 '.' 0.1 ' ' 1  
 
 
  
 
 
 
  Taxonomic
groups  
 
 Individual coefficients 
      mod.taxa  <-   run.model (d,  ~ taxonomic_group  -   1 ) 
    summary (mod.taxa)    
  ## 
## Multivariate Meta-Analysis Model (k = 1089; method: REML)
## 
##    logLik   Deviance        AIC        BIC       AICc  ​ 
## -220.0038   440.0075   456.0075   495.9148   456.1415   
## 
## Variance Components:
## 
##             estim    sqrt  nlvls  fixed      factor    R 
## sigma^2.1  0.0092  0.0957    138     no  species_ID   no 
## sigma^2.2  0.0242  0.1554    138     no   phylogeny  yes 
## sigma^2.3  0.0605  0.2459   1089     no       es_ID   no 
## 
## Test for Residual Heterogeneity:
## QE(df = 1084) = 56018.6724, p-val < .0001
## 
## Test of Moderators (coefficients 1:5):
## F(df1 = 5, df2 = 133) = 2.0368, p-val = 0.0775
## 
## Model Results:
## 
##                                          estimate      se    tval   df    pval​ 
## taxonomic_groupamphibian                   0.2083  0.1541  1.3521  133  0.1786 
## taxonomic_groupaquatic invertebrate        0.1904  0.1131  1.6838  133  0.0946 
## taxonomic_groupfish                        0.2413  0.1161  2.0783  133  0.0396 
## taxonomic_groupreptile                     0.0667  0.1634  0.4082  133  0.6838 
## taxonomic_groupterrestrial invertebrate    0.0535  0.1288  0.4157  133  0.6783 
##                                            ci.lb   ci.ub 
## taxonomic_groupamphibian                 -0.0964  0.5131    
## taxonomic_groupaquatic invertebrate      -0.0333  0.4142  . 
## taxonomic_groupfish                       0.0116  0.4709  * 
## taxonomic_groupreptile                   -0.2564  0.3898    
## taxonomic_groupterrestrial invertebrate  -0.2012  0.3082    
## 
## ---
## Signif. codes:  0 '***' 0.001 '**' 0.01 '*' 0.05 '.' 0.1 ' ' 1  
       mod_results (mod.taxa,  mod =   "taxonomic_group" ,  data =  d,  group =   "species_ID" )    
  ##                       name   estimate     lowerCL   upperCL    lowerPR
## 1                Amphibian 0.20831727 -0.09642755 0.5130621 -0.4697867
## 2     Aquatic invertebrate 0.19043896 -0.03327497 0.4141529 -0.4553191
## 3                     Fish 0.24127906  0.01164449 0.4709136 -0.4065539
## 4                  Reptile 0.06668682 -0.25643776 0.3898114 -0.6198735
## 5 Terrestrial invertebrate 0.05352402 -0.20117964 0.3082277 -0.6036133
##     upperPR
## 1 0.8864212
## 2 0.8361970
## 3 0.8891120
## 4 0.7532471
## 5 0.7106614  
       r2_ml (mod.taxa)    
  ##    R2_marginal R2_conditional 
##     0.04420462     0.38371519  
       my.orchard (mod.taxa,  mod =   "taxonomic_group" ,  xlab =   "dARR" ,  alpha =   0.1 ,  data =  d, 
        group =   "species_ID" ,  whisker =   0.09 )    
   
 
 
 Contrasts 
       # Reptiles as the reference group  
   mod.taxa_reptile_ref_cont  <-   run.model (d,  ~  relevel ( factor (taxonomic_group),  ref =   "reptile" )) 
    summary (mod.taxa_reptile_ref_cont)    
  ## 
## Multivariate Meta-Analysis Model (k = 1089; method: REML)
## 
##    logLik   Deviance        AIC        BIC       AICc  ​ 
## -220.0038   440.0075   456.0075   495.9148   456.1415   
## 
## Variance Components:
## 
##             estim    sqrt  nlvls  fixed      factor    R 
## sigma^2.1  0.0092  0.0957    138     no  species_ID   no 
## sigma^2.2  0.0242  0.1554    138     no   phylogeny  yes 
## sigma^2.3  0.0605  0.2459   1089     no       es_ID   no 
## 
## Test for Residual Heterogeneity:
## QE(df = 1084) = 56018.6724, p-val < .0001
## 
## Test of Moderators (coefficients 2:5):
## F(df1 = 4, df2 = 133) = 1.3538, p-val = 0.2535
## 
## Model Results:
## 
##                                                                            estimate​ 
## intrcpt                                                                      0.0667 
## relevel(factor(taxonomic_group), ref = "reptile")amphibian                   0.1416 
## relevel(factor(taxonomic_group), ref = "reptile")aquatic invertebrate        0.1238 
## relevel(factor(taxonomic_group), ref = "reptile")fish                        0.1746 
## relevel(factor(taxonomic_group), ref = "reptile")terrestrial invertebrate   -0.0132 
##                                                                                se 
## intrcpt                                                                    0.1634 
## relevel(factor(taxonomic_group), ref = "reptile")amphibian                 0.1070 
## relevel(factor(taxonomic_group), ref = "reptile")aquatic invertebrate      0.1837 
## relevel(factor(taxonomic_group), ref = "reptile")fish                      0.1550 
## relevel(factor(taxonomic_group), ref = "reptile")terrestrial invertebrate  0.1949 
##                                                                               tval 
## intrcpt                                                                     0.4082 
## relevel(factor(taxonomic_group), ref = "reptile")amphibian                  1.3231 
## relevel(factor(taxonomic_group), ref = "reptile")aquatic invertebrate       0.6737 
## relevel(factor(taxonomic_group), ref = "reptile")fish                       1.1261 
## relevel(factor(taxonomic_group), ref = "reptile")terrestrial invertebrate  -0.0675 
##                                                                             df 
## intrcpt                                                                    133 
## relevel(factor(taxonomic_group), ref = "reptile")amphibian                 133 
## relevel(factor(taxonomic_group), ref = "reptile")aquatic invertebrate      133 
## relevel(factor(taxonomic_group), ref = "reptile")fish                      133 
## relevel(factor(taxonomic_group), ref = "reptile")terrestrial invertebrate  133 
##                                                                              pval 
## intrcpt                                                                    0.6838 
## relevel(factor(taxonomic_group), ref = "reptile")amphibian                 0.1881 
## relevel(factor(taxonomic_group), ref = "reptile")aquatic invertebrate      0.5016 
## relevel(factor(taxonomic_group), ref = "reptile")fish                      0.2621 
## relevel(factor(taxonomic_group), ref = "reptile")terrestrial invertebrate  0.9463 
##                                                                              ci.lb 
## intrcpt                                                                    -0.2564 
## relevel(factor(taxonomic_group), ref = "reptile")amphibian                 -0.0701 
## relevel(factor(taxonomic_group), ref = "reptile")aquatic invertebrate      -0.2396 
## relevel(factor(taxonomic_group), ref = "reptile")fish                      -0.1321 
## relevel(factor(taxonomic_group), ref = "reptile")terrestrial invertebrate  -0.3987 
##                                                                             ci.ub 
## intrcpt                                                                    0.3898 
## relevel(factor(taxonomic_group), ref = "reptile")amphibian                 0.3534 
## relevel(factor(taxonomic_group), ref = "reptile")aquatic invertebrate      0.4871 
## relevel(factor(taxonomic_group), ref = "reptile")fish                      0.4813 
## relevel(factor(taxonomic_group), ref = "reptile")terrestrial invertebrate  0.3724 
##  
## intrcpt 
## relevel(factor(taxonomic_group), ref = "reptile")amphibian 
## relevel(factor(taxonomic_group), ref = "reptile")aquatic invertebrate 
## relevel(factor(taxonomic_group), ref = "reptile")fish 
## relevel(factor(taxonomic_group), ref = "reptile")terrestrial invertebrate 
## 
## ---
## Signif. codes:  0 '***' 0.001 '**' 0.01 '*' 0.05 '.' 0.1 ' ' 1  
       # Terrestrial invertebrates as the reference group  
   mod.taxa_tinvert_ref_cont  <-   run.model (d,  ~  relevel ( factor (taxonomic_group),  ref =   "terrestrial invertebrate" )) 
    summary (mod.taxa_tinvert_ref_cont)    
  ## 
## Multivariate Meta-Analysis Model (k = 1089; method: REML)
## 
##    logLik   Deviance        AIC        BIC       AICc  ​ 
## -220.0038   440.0075   456.0075   495.9148   456.1415   
## 
## Variance Components:
## 
##             estim    sqrt  nlvls  fixed      factor    R 
## sigma^2.1  0.0092  0.0957    138     no  species_ID   no 
## sigma^2.2  0.0242  0.1554    138     no   phylogeny  yes 
## sigma^2.3  0.0605  0.2459   1089     no       es_ID   no 
## 
## Test for Residual Heterogeneity:
## QE(df = 1084) = 56018.6724, p-val < .0001
## 
## Test of Moderators (coefficients 2:5):
## F(df1 = 4, df2 = 133) = 1.3538, p-val = 0.2535
## 
## Model Results:
## 
##                                                                                         estimate​ 
## intrcpt                                                                                   0.0535 
## relevel(factor(taxonomic_group), ref = "terrestrial invertebrate")amphibian               0.1548 
## relevel(factor(taxonomic_group), ref = "terrestrial invertebrate")aquatic invertebrate    0.1369 
## relevel(factor(taxonomic_group), ref = "terrestrial invertebrate")fish                    0.1878 
## relevel(factor(taxonomic_group), ref = "terrestrial invertebrate")reptile                 0.0132 
##                                                                                             se 
## intrcpt                                                                                 0.1288 
## relevel(factor(taxonomic_group), ref = "terrestrial invertebrate")amphibian             0.1872 
## relevel(factor(taxonomic_group), ref = "terrestrial invertebrate")aquatic invertebrate  0.0768 
## relevel(factor(taxonomic_group), ref = "terrestrial invertebrate")fish                  0.1575 
## relevel(factor(taxonomic_group), ref = "terrestrial invertebrate")reptile               0.1949 
##                                                                                           tval 
## intrcpt                                                                                 0.4157 
## relevel(factor(taxonomic_group), ref = "terrestrial invertebrate")amphibian             0.8268 
## relevel(factor(taxonomic_group), ref = "terrestrial invertebrate")aquatic invertebrate  1.7824 
## relevel(factor(taxonomic_group), ref = "terrestrial invertebrate")fish                  1.1924 
## relevel(factor(taxonomic_group), ref = "terrestrial invertebrate")reptile               0.0675 
##                                                                                          df 
## intrcpt                                                                                 133 
## relevel(factor(taxonomic_group), ref = "terrestrial invertebrate")amphibian             133 
## relevel(factor(taxonomic_group), ref = "terrestrial invertebrate")aquatic invertebrate  133 
## relevel(factor(taxonomic_group), ref = "terrestrial invertebrate")fish                  133 
## relevel(factor(taxonomic_group), ref = "terrestrial invertebrate")reptile               133 
##                                                                                           pval 
## intrcpt                                                                                 0.6783 
## relevel(factor(taxonomic_group), ref = "terrestrial invertebrate")amphibian             0.4098 
## relevel(factor(taxonomic_group), ref = "terrestrial invertebrate")aquatic invertebrate  0.0770 
## relevel(factor(taxonomic_group), ref = "terrestrial invertebrate")fish                  0.2352 
## relevel(factor(taxonomic_group), ref = "terrestrial invertebrate")reptile               0.9463 
##                                                                                           ci.lb 
## intrcpt                                                                                 -0.2012 
## relevel(factor(taxonomic_group), ref = "terrestrial invertebrate")amphibian             -0.2155 
## relevel(factor(taxonomic_group), ref = "terrestrial invertebrate")aquatic invertebrate  -0.0150 
## relevel(factor(taxonomic_group), ref = "terrestrial invertebrate")fish                  -0.1237 
## relevel(factor(taxonomic_group), ref = "terrestrial invertebrate")reptile               -0.3724 
##                                                                                          ci.ub 
## intrcpt                                                                                 0.3082 
## relevel(factor(taxonomic_group), ref = "terrestrial invertebrate")amphibian             0.5251 
## relevel(factor(taxonomic_group), ref = "terrestrial invertebrate")aquatic invertebrate  0.2888 
## relevel(factor(taxonomic_group), ref = "terrestrial invertebrate")fish                  0.4992 
## relevel(factor(taxonomic_group), ref = "terrestrial invertebrate")reptile               0.3987 
##  
## intrcpt 
## relevel(factor(taxonomic_group), ref = "terrestrial invertebrate")amphibian 
## relevel(factor(taxonomic_group), ref = "terrestrial invertebrate")aquatic invertebrate  . 
## relevel(factor(taxonomic_group), ref = "terrestrial invertebrate")fish 
## relevel(factor(taxonomic_group), ref = "terrestrial invertebrate")reptile 
## 
## ---
## Signif. codes:  0 '***' 0.001 '**' 0.01 '*' 0.05 '.' 0.1 ' ' 1  
       # Aquatic invertebrates as the reference group  
   mod.taxa_ainvert_ref_cont  <-   run.model (d,  ~  relevel ( factor (taxonomic_group),  ref =   "aquatic invertebrate" )) 
    summary (mod.taxa_ainvert_ref_cont)    
  ## 
## Multivariate Meta-Analysis Model (k = 1089; method: REML)
## 
##    logLik   Deviance        AIC        BIC       AICc  ​ 
## -220.0038   440.0075   456.0075   495.9148   456.1415   
## 
## Variance Components:
## 
##             estim    sqrt  nlvls  fixed      factor    R 
## sigma^2.1  0.0092  0.0957    138     no  species_ID   no 
## sigma^2.2  0.0242  0.1554    138     no   phylogeny  yes 
## sigma^2.3  0.0605  0.2459   1089     no       es_ID   no 
## 
## Test for Residual Heterogeneity:
## QE(df = 1084) = 56018.6724, p-val < .0001
## 
## Test of Moderators (coefficients 2:5):
## F(df1 = 4, df2 = 133) = 1.3538, p-val = 0.2535
## 
## Model Results:
## 
##                                                                                         estimate​ 
## intrcpt                                                                                   0.1904 
## relevel(factor(taxonomic_group), ref = "aquatic invertebrate")amphibian                   0.0179 
## relevel(factor(taxonomic_group), ref = "aquatic invertebrate")fish                        0.0508 
## relevel(factor(taxonomic_group), ref = "aquatic invertebrate")reptile                    -0.1238 
## relevel(factor(taxonomic_group), ref = "aquatic invertebrate")terrestrial invertebrate   -0.1369 
##                                                                                             se 
## intrcpt                                                                                 0.1131 
## relevel(factor(taxonomic_group), ref = "aquatic invertebrate")amphibian                 0.1755 
## relevel(factor(taxonomic_group), ref = "aquatic invertebrate")fish                      0.1433 
## relevel(factor(taxonomic_group), ref = "aquatic invertebrate")reptile                   0.1837 
## relevel(factor(taxonomic_group), ref = "aquatic invertebrate")terrestrial invertebrate  0.0768 
##                                                                                            tval 
## intrcpt                                                                                  1.6838 
## relevel(factor(taxonomic_group), ref = "aquatic invertebrate")amphibian                  0.1019 
## relevel(factor(taxonomic_group), ref = "aquatic invertebrate")fish                       0.3548 
## relevel(factor(taxonomic_group), ref = "aquatic invertebrate")reptile                   -0.6737 
## relevel(factor(taxonomic_group), ref = "aquatic invertebrate")terrestrial invertebrate  -1.7824 
##                                                                                          df 
## intrcpt                                                                                 133 
## relevel(factor(taxonomic_group), ref = "aquatic invertebrate")amphibian                 133 
## relevel(factor(taxonomic_group), ref = "aquatic invertebrate")fish                      133 
## relevel(factor(taxonomic_group), ref = "aquatic invertebrate")reptile                   133 
## relevel(factor(taxonomic_group), ref = "aquatic invertebrate")terrestrial invertebrate  133 
##                                                                                           pval 
## intrcpt                                                                                 0.0946 
## relevel(factor(taxonomic_group), ref = "aquatic invertebrate")amphibian                 0.9190 
## relevel(factor(taxonomic_group), ref = "aquatic invertebrate")fish                      0.7233 
## relevel(factor(taxonomic_group), ref = "aquatic invertebrate")reptile                   0.5016 
## relevel(factor(taxonomic_group), ref = "aquatic invertebrate")terrestrial invertebrate  0.0770 
##                                                                                           ci.lb 
## intrcpt                                                                                 -0.0333 
## relevel(factor(taxonomic_group), ref = "aquatic invertebrate")amphibian                 -0.3292 
## relevel(factor(taxonomic_group), ref = "aquatic invertebrate")fish                      -0.2326 
## relevel(factor(taxonomic_group), ref = "aquatic invertebrate")reptile                   -0.4871 
## relevel(factor(taxonomic_group), ref = "aquatic invertebrate")terrestrial invertebrate  -0.2888 
##                                                                                          ci.ub 
## intrcpt                                                                                 0.4142 
## relevel(factor(taxonomic_group), ref = "aquatic invertebrate")amphibian                 0.3649 
## relevel(factor(taxonomic_group), ref = "aquatic invertebrate")fish                      0.3342 
## relevel(factor(taxonomic_group), ref = "aquatic invertebrate")reptile                   0.2396 
## relevel(factor(taxonomic_group), ref = "aquatic invertebrate")terrestrial invertebrate  0.0150 
##  
## intrcpt                                                                                 . 
## relevel(factor(taxonomic_group), ref = "aquatic invertebrate")amphibian 
## relevel(factor(taxonomic_group), ref = "aquatic invertebrate")fish 
## relevel(factor(taxonomic_group), ref = "aquatic invertebrate")reptile 
## relevel(factor(taxonomic_group), ref = "aquatic invertebrate")terrestrial invertebrate  . 
## 
## ---
## Signif. codes:  0 '***' 0.001 '**' 0.01 '*' 0.05 '.' 0.1 ' ' 1  
       # Amphibians as the reference group  
   mod.taxa_amph_ref_cont  <-   run.model (d,  ~  relevel ( factor (taxonomic_group),  ref =   "amphibian" )) 
    summary (mod.taxa_amph_ref_cont)    
  ## 
## Multivariate Meta-Analysis Model (k = 1089; method: REML)
## 
##    logLik   Deviance        AIC        BIC       AICc  ​ 
## -220.0038   440.0075   456.0075   495.9148   456.1415   
## 
## Variance Components:
## 
##             estim    sqrt  nlvls  fixed      factor    R 
## sigma^2.1  0.0092  0.0957    138     no  species_ID   no 
## sigma^2.2  0.0242  0.1554    138     no   phylogeny  yes 
## sigma^2.3  0.0605  0.2459   1089     no       es_ID   no 
## 
## Test for Residual Heterogeneity:
## QE(df = 1084) = 56018.6724, p-val < .0001
## 
## Test of Moderators (coefficients 2:5):
## F(df1 = 4, df2 = 133) = 1.3538, p-val = 0.2535
## 
## Model Results:
## 
##                                                                              estimate​ 
## intrcpt                                                                        0.2083 
## relevel(factor(taxonomic_group), ref = "amphibian")aquatic invertebrate       -0.0179 
## relevel(factor(taxonomic_group), ref = "amphibian")fish                        0.0330 
## relevel(factor(taxonomic_group), ref = "amphibian")reptile                    -0.1416 
## relevel(factor(taxonomic_group), ref = "amphibian")terrestrial invertebrate   -0.1548 
##                                                                                  se 
## intrcpt                                                                      0.1541 
## relevel(factor(taxonomic_group), ref = "amphibian")aquatic invertebrate      0.1755 
## relevel(factor(taxonomic_group), ref = "amphibian")fish                      0.1452 
## relevel(factor(taxonomic_group), ref = "amphibian")reptile                   0.1070 
## relevel(factor(taxonomic_group), ref = "amphibian")terrestrial invertebrate  0.1872 
##                                                                                 tval 
## intrcpt                                                                       1.3521 
## relevel(factor(taxonomic_group), ref = "amphibian")aquatic invertebrate      -0.1019 
## relevel(factor(taxonomic_group), ref = "amphibian")fish                       0.2270 
## relevel(factor(taxonomic_group), ref = "amphibian")reptile                   -1.3231 
## relevel(factor(taxonomic_group), ref = "amphibian")terrestrial invertebrate  -0.8268 
##                                                                               df 
## intrcpt                                                                      133 
## relevel(factor(taxonomic_group), ref = "amphibian")aquatic invertebrate      133 
## relevel(factor(taxonomic_group), ref = "amphibian")fish                      133 
## relevel(factor(taxonomic_group), ref = "amphibian")reptile                   133 
## relevel(factor(taxonomic_group), ref = "amphibian")terrestrial invertebrate  133 
##                                                                                pval 
## intrcpt                                                                      0.1786 
## relevel(factor(taxonomic_group), ref = "amphibian")aquatic invertebrate      0.9190 
## relevel(factor(taxonomic_group), ref = "amphibian")fish                      0.8208 
## relevel(factor(taxonomic_group), ref = "amphibian")reptile                   0.1881 
## relevel(factor(taxonomic_group), ref = "amphibian")terrestrial invertebrate  0.4098 
##                                                                                ci.lb 
## intrcpt                                                                      -0.0964 
## relevel(factor(taxonomic_group), ref = "amphibian")aquatic invertebrate      -0.3649 
## relevel(factor(taxonomic_group), ref = "amphibian")fish                      -0.2543 
## relevel(factor(taxonomic_group), ref = "amphibian")reptile                   -0.3534 
## relevel(factor(taxonomic_group), ref = "amphibian")terrestrial invertebrate  -0.5251 
##                                                                               ci.ub 
## intrcpt                                                                      0.5131 
## relevel(factor(taxonomic_group), ref = "amphibian")aquatic invertebrate      0.3292 
## relevel(factor(taxonomic_group), ref = "amphibian")fish                      0.3202 
## relevel(factor(taxonomic_group), ref = "amphibian")reptile                   0.0701 
## relevel(factor(taxonomic_group), ref = "amphibian")terrestrial invertebrate  0.2155 
##  
## intrcpt 
## relevel(factor(taxonomic_group), ref = "amphibian")aquatic invertebrate 
## relevel(factor(taxonomic_group), ref = "amphibian")fish 
## relevel(factor(taxonomic_group), ref = "amphibian")reptile 
## relevel(factor(taxonomic_group), ref = "amphibian")terrestrial invertebrate 
## 
## ---
## Signif. codes:  0 '***' 0.001 '**' 0.01 '*' 0.05 '.' 0.1 ' ' 1  
       # Fishes as the reference group  
   mod.taxa_fish_ref_cont  <-   run.model (d,  ~  relevel ( factor (taxonomic_group),  ref =   "fish" )) 
    summary (mod.taxa_fish_ref_cont)    
  ## 
## Multivariate Meta-Analysis Model (k = 1089; method: REML)
## 
##    logLik   Deviance        AIC        BIC       AICc  ​ 
## -220.0038   440.0075   456.0075   495.9148   456.1415   
## 
## Variance Components:
## 
##             estim    sqrt  nlvls  fixed      factor    R 
## sigma^2.1  0.0092  0.0957    138     no  species_ID   no 
## sigma^2.2  0.0242  0.1554    138     no   phylogeny  yes 
## sigma^2.3  0.0605  0.2459   1089     no       es_ID   no 
## 
## Test for Residual Heterogeneity:
## QE(df = 1084) = 56018.6724, p-val < .0001
## 
## Test of Moderators (coefficients 2:5):
## F(df1 = 4, df2 = 133) = 1.3538, p-val = 0.2535
## 
## Model Results:
## 
##                                                                         estimate​ 
## intrcpt                                                                   0.2413 
## relevel(factor(taxonomic_group), ref = "fish")amphibian                  -0.0330 
## relevel(factor(taxonomic_group), ref = "fish")aquatic invertebrate       -0.0508 
## relevel(factor(taxonomic_group), ref = "fish")reptile                    -0.1746 
## relevel(factor(taxonomic_group), ref = "fish")terrestrial invertebrate   -0.1878 
##                                                                             se 
## intrcpt                                                                 0.1161 
## relevel(factor(taxonomic_group), ref = "fish")amphibian                 0.1452 
## relevel(factor(taxonomic_group), ref = "fish")aquatic invertebrate      0.1433 
## relevel(factor(taxonomic_group), ref = "fish")reptile                   0.1550 
## relevel(factor(taxonomic_group), ref = "fish")terrestrial invertebrate  0.1575 
##                                                                            tval 
## intrcpt                                                                  2.0783 
## relevel(factor(taxonomic_group), ref = "fish")amphibian                 -0.2270 
## relevel(factor(taxonomic_group), ref = "fish")aquatic invertebrate      -0.3548 
## relevel(factor(taxonomic_group), ref = "fish")reptile                   -1.1261 
## relevel(factor(taxonomic_group), ref = "fish")terrestrial invertebrate  -1.1924 
##                                                                          df 
## intrcpt                                                                 133 
## relevel(factor(taxonomic_group), ref = "fish")amphibian                 133 
## relevel(factor(taxonomic_group), ref = "fish")aquatic invertebrate      133 
## relevel(factor(taxonomic_group), ref = "fish")reptile                   133 
## relevel(factor(taxonomic_group), ref = "fish")terrestrial invertebrate  133 
##                                                                           pval 
## intrcpt                                                                 0.0396 
## relevel(factor(taxonomic_group), ref = "fish")amphibian                 0.8208 
## relevel(factor(taxonomic_group), ref = "fish")aquatic invertebrate      0.7233 
## relevel(factor(taxonomic_group), ref = "fish")reptile                   0.2621 
## relevel(factor(taxonomic_group), ref = "fish")terrestrial invertebrate  0.2352 
##                                                                           ci.lb 
## intrcpt                                                                  0.0116 
## relevel(factor(taxonomic_group), ref = "fish")amphibian                 -0.3202 
## relevel(factor(taxonomic_group), ref = "fish")aquatic invertebrate      -0.3342 
## relevel(factor(taxonomic_group), ref = "fish")reptile                   -0.4813 
## relevel(factor(taxonomic_group), ref = "fish")terrestrial invertebrate  -0.4992 
##                                                                          ci.ub 
## intrcpt                                                                 0.4709 
## relevel(factor(taxonomic_group), ref = "fish")amphibian                 0.2543 
## relevel(factor(taxonomic_group), ref = "fish")aquatic invertebrate      0.2326 
## relevel(factor(taxonomic_group), ref = "fish")reptile                   0.1321 
## relevel(factor(taxonomic_group), ref = "fish")terrestrial invertebrate  0.1237 
##  
## intrcpt                                                                 * 
## relevel(factor(taxonomic_group), ref = "fish")amphibian 
## relevel(factor(taxonomic_group), ref = "fish")aquatic invertebrate 
## relevel(factor(taxonomic_group), ref = "fish")reptile 
## relevel(factor(taxonomic_group), ref = "fish")terrestrial invertebrate 
## 
## ---
## Signif. codes:  0 '***' 0.001 '**' 0.01 '*' 0.05 '.' 0.1 ' ' 1  
 
 
 Heteroscedasticity check 
       qplot ( y =   sqrt ( residuals (mod.taxa) ^  2 ),  x =   fitted (mod.taxa))  +   geom_point ()  +   geom_smooth ( method =   "lm" )  +  
        geom_hline ( yintercept =   0 ,  colour =   "red" )   # Seems heteroscedastic     
   
 
 
 Individual coefficients (with heteroscedasticity) 
 Here, the heteroscedasticity is also modeled at both the effect size
and cohort levels, which improved the model fit. 
      mod.taxa_het  <-   rma.mv ( yi =  dARR,  V =  VCV_dARR,  mods =   ~ taxonomic_group  -   1 ,  method =   "REML" , 
        test =   "t" ,  dfs =   "contain" ,  random =   list ( ~  1   |  species_ID,  ~  1   |  phylogeny,  ~ taxonomic_group  |  
           es_ID),  struct =   "HCS" ,  rho =   0 ,  R =   list ( phylogeny =  phylo_matrix),  data =  d, 
        sparse =   TRUE ) 
    AICc (mod.taxa)    
  ## [1] 456.1409  
       AICc (mod.taxa_het)   # Improved model fit     
  ## [1] 133.3114  
       summary (mod.taxa_het)    
  ## 
## Multivariate Meta-Analysis Model (k = 1089; method: REML)
## 
##   logLik  Deviance       AIC       BIC      AICc  <U+200B> 
## -54.5107  109.0214  133.0214  192.8824  133.3128   
## 
## Variance Components:
## 
##             estim    sqrt  nlvls  fixed      factor    R 
## sigma^2.1  0.0090  0.0946    138     no  species_ID   no 
## sigma^2.2  0.0296  0.1722    138     no   phylogeny  yes 
## 
## outer factor: es_ID           (nlvls = 1089)
## inner factor: taxonomic_group (nlvls = 5)
## 
##             estim    sqrt  k.lvl  fixed                     level 
## tau^2.1    0.1878  0.4334     71     no                 amphibian 
## tau^2.2    0.1357  0.3684    221     no      aquatic invertebrate 
## tau^2.3    0.0329  0.1813    623     no                      fish 
## tau^2.4    0.0163  0.1278     27     no                   reptile 
## tau^2.5    0.0069  0.0833    147     no  terrestrial invertebrate 
## rho        0.0000                   yes                           
## 
## Test for Residual Heterogeneity:
## QE(df = 1084) = 56018.6724, p-val < .0001
## 
## Test of Moderators (coefficients 1:5):
## F(df1 = 5, df2 = 133) = 1.9108, p-val = 0.0966
## 
## Model Results:
## 
##                                          estimate      se    tval   df    pval<U+200B> 
## taxonomic_groupamphibian                   0.1966  0.1761  1.1167  133  0.2661 
## taxonomic_groupaquatic invertebrate        0.1992  0.1286  1.5492  133  0.1237 
## taxonomic_groupfish                        0.2538  0.1265  2.0058  133  0.0469 
## taxonomic_groupreptile                     0.0702  0.1733  0.4053  133  0.6859 
## taxonomic_groupterrestrial invertebrate    0.0488  0.1410  0.3464  133  0.7296 
##                                            ci.lb   ci.ub 
## taxonomic_groupamphibian                 -0.1517  0.5449    
## taxonomic_groupaquatic invertebrate      -0.0551  0.4535    
## taxonomic_groupfish                       0.0035  0.5041  * 
## taxonomic_groupreptile                   -0.2726  0.4131    
## taxonomic_groupterrestrial invertebrate  -0.2301  0.3278    
## 
## ---
## Signif. codes:  0 '***' 0.001 '**' 0.01 '*' 0.05 '.' 0.1 ' ' 1  
       mod_results (mod.taxa_het,  mod =   "taxonomic_group" ,  data =  d,  group =   "species_ID" )    
  ##                       name   estimate      lowerCL   upperCL    lowerPR
## 1                Amphibian 0.19664022 -0.151655428 0.5449359 -0.8069271
## 2     Aquatic invertebrate 0.19919762 -0.055126478 0.4535217 -0.6649603
## 3                     Fish 0.25382273  0.003518141 0.5041273 -0.3312188
## 4                  Reptile 0.07024502 -0.272560393 0.4130504 -0.5063220
## 5 Terrestrial invertebrate 0.04884628 -0.230059070 0.3277516 -0.4571120
##     upperPR
## 1 1.2002076
## 2 1.0633556
## 3 0.8388642
## 4 0.6468121
## 5 0.5548046  
       r2_ml (mod.taxa_het)    
  ##    R2_marginal R2_conditional 
##      0.1170636      0.3219044  
       my.orchard (mod.taxa_het,  mod =   "taxonomic_group" ,  xlab =   "dARR" ,  alpha =   0.1 ,  data =  d, 
        group =   "species_ID" ,  whisker =   0.09 )    
   
 
 
 Contrasts (with heteroscedasticity) 
       ## Reptiles as the reference group  
   mod.taxa_het_cont_rept_ref  <-   rma.mv ( yi =  dARR,  V =  VCV_dARR,  mods =   ~  relevel ( factor (taxonomic_group), 
        ref =   "reptile" ),  method =   "REML" ,  test =   "t" ,  dfs =   "contain" ,  random =   list ( ~  1   |  
       species_ID,  ~  1   |  phylogeny,  ~ taxonomic_group  |  es_ID),  struct =   "HCS" ,  rho =   0 , 
        R =   list ( phylogeny =  phylo_matrix),  data =  d,  sparse =   TRUE ) 
    
    summary (mod.taxa_het_cont_rept_ref)    
  ## 
## Multivariate Meta-Analysis Model (k = 1089; method: REML)
## 
##   logLik  Deviance       AIC       BIC      AICc  <U+200B> 
## -54.5107  109.0214  133.0214  192.8824  133.3128   
## 
## Variance Components:
## 
##             estim    sqrt  nlvls  fixed      factor    R 
## sigma^2.1  0.0090  0.0946    138     no  species_ID   no 
## sigma^2.2  0.0296  0.1722    138     no   phylogeny  yes 
## 
## outer factor: es_ID           (nlvls = 1089)
## inner factor: taxonomic_group (nlvls = 5)
## 
##             estim    sqrt  k.lvl  fixed                     level 
## tau^2.1    0.1878  0.4334     71     no                 amphibian 
## tau^2.2    0.1357  0.3684    221     no      aquatic invertebrate 
## tau^2.3    0.0329  0.1813    623     no                      fish 
## tau^2.4    0.0163  0.1278     27     no                   reptile 
## tau^2.5    0.0069  0.0833    147     no  terrestrial invertebrate 
## rho        0.0000                   yes                           
## 
## Test for Residual Heterogeneity:
## QE(df = 1084) = 56018.6724, p-val < .0001
## 
## Test of Moderators (coefficients 2:5):
## F(df1 = 4, df2 = 133) = 1.3982, p-val = 0.2380
## 
## Model Results:
## 
##                                                                            estimate<U+200B> 
## intrcpt                                                                      0.0702 
## relevel(factor(taxonomic_group), ref = "reptile")amphibian                   0.1264 
## relevel(factor(taxonomic_group), ref = "reptile")aquatic invertebrate        0.1290 
## relevel(factor(taxonomic_group), ref = "reptile")fish                        0.1836 
## relevel(factor(taxonomic_group), ref = "reptile")terrestrial invertebrate   -0.0214 
##                                                                                se 
## intrcpt                                                                    0.1733 
## relevel(factor(taxonomic_group), ref = "reptile")amphibian                 0.1140 
## relevel(factor(taxonomic_group), ref = "reptile")aquatic invertebrate      0.2002 
## relevel(factor(taxonomic_group), ref = "reptile")fish                      0.1615 
## relevel(factor(taxonomic_group), ref = "reptile")terrestrial invertebrate  0.2098 
##                                                                               tval 
## intrcpt                                                                     0.4053 
## relevel(factor(taxonomic_group), ref = "reptile")amphibian                  1.1090 
## relevel(factor(taxonomic_group), ref = "reptile")aquatic invertebrate       0.6442 
## relevel(factor(taxonomic_group), ref = "reptile")fish                       1.1365 
## relevel(factor(taxonomic_group), ref = "reptile")terrestrial invertebrate  -0.1020 
##                                                                             df 
## intrcpt                                                                    133 
## relevel(factor(taxonomic_group), ref = "reptile")amphibian                 133 
## relevel(factor(taxonomic_group), ref = "reptile")aquatic invertebrate      133 
## relevel(factor(taxonomic_group), ref = "reptile")fish                      133 
## relevel(factor(taxonomic_group), ref = "reptile")terrestrial invertebrate  133 
##                                                                              pval 
## intrcpt                                                                    0.6859 
## relevel(factor(taxonomic_group), ref = "reptile")amphibian                 0.2694 
## relevel(factor(taxonomic_group), ref = "reptile")aquatic invertebrate      0.5206 
## relevel(factor(taxonomic_group), ref = "reptile")fish                      0.2578 
## relevel(factor(taxonomic_group), ref = "reptile")terrestrial invertebrate  0.9189 
##                                                                              ci.lb 
## intrcpt                                                                    -0.2726 
## relevel(factor(taxonomic_group), ref = "reptile")amphibian                 -0.0990 
## relevel(factor(taxonomic_group), ref = "reptile")aquatic invertebrate      -0.2670 
## relevel(factor(taxonomic_group), ref = "reptile")fish                      -0.1359 
## relevel(factor(taxonomic_group), ref = "reptile")terrestrial invertebrate  -0.4363 
##                                                                             ci.ub 
## intrcpt                                                                    0.4131 
## relevel(factor(taxonomic_group), ref = "reptile")amphibian                 0.3518 
## relevel(factor(taxonomic_group), ref = "reptile")aquatic invertebrate      0.5249 
## relevel(factor(taxonomic_group), ref = "reptile")fish                      0.5031 
## relevel(factor(taxonomic_group), ref = "reptile")terrestrial invertebrate  0.3935 
##  
## intrcpt 
## relevel(factor(taxonomic_group), ref = "reptile")amphibian 
## relevel(factor(taxonomic_group), ref = "reptile")aquatic invertebrate 
## relevel(factor(taxonomic_group), ref = "reptile")fish 
## relevel(factor(taxonomic_group), ref = "reptile")terrestrial invertebrate 
## 
## ---
## Signif. codes:  0 '***' 0.001 '**' 0.01 '*' 0.05 '.' 0.1 ' ' 1  
       ## Terrestrial invertebrates as the reference group  
   mod.taxa_het_cont_tinvert_ref  <-   rma.mv ( yi =  dARR,  V =  VCV_dARR,  mods =   ~  relevel ( factor (taxonomic_group), 
        ref =   "terrestrial invertebrate" ),  method =   "REML" ,  test =   "t" ,  dfs =   "contain" , 
        random =   list ( ~  1   |  species_ID,  ~  1   |  phylogeny,  ~ taxonomic_group  |  es_ID),  struct =   "HCS" , 
        rho =   0 ,  R =   list ( phylogeny =  phylo_matrix),  data =  d,  sparse =   TRUE ) 
    summary (mod.taxa_het_cont_tinvert_ref)    
  ## 
## Multivariate Meta-Analysis Model (k = 1089; method: REML)
## 
##   logLik  Deviance       AIC       BIC      AICc  <U+200B> 
## -54.5107  109.0214  133.0214  192.8824  133.3128   
## 
## Variance Components:
## 
##             estim    sqrt  nlvls  fixed      factor    R 
## sigma^2.1  0.0090  0.0946    138     no  species_ID   no 
## sigma^2.2  0.0296  0.1722    138     no   phylogeny  yes 
## 
## outer factor: es_ID           (nlvls = 1089)
## inner factor: taxonomic_group (nlvls = 5)
## 
##             estim    sqrt  k.lvl  fixed                     level 
## tau^2.1    0.1878  0.4334     71     no                 amphibian 
## tau^2.2    0.1357  0.3684    221     no      aquatic invertebrate 
## tau^2.3    0.0329  0.1813    623     no                      fish 
## tau^2.4    0.0163  0.1278     27     no                   reptile 
## tau^2.5    0.0069  0.0833    147     no  terrestrial invertebrate 
## rho        0.0000                   yes                           
## 
## Test for Residual Heterogeneity:
## QE(df = 1084) = 56018.6724, p-val < .0001
## 
## Test of Moderators (coefficients 2:5):
## F(df1 = 4, df2 = 133) = 1.3982, p-val = 0.2380
## 
## Model Results:
## 
##                                                                                         estimate<U+200B> 
## intrcpt                                                                                   0.0488 
## relevel(factor(taxonomic_group), ref = "terrestrial invertebrate")amphibian               0.1478 
## relevel(factor(taxonomic_group), ref = "terrestrial invertebrate")aquatic invertebrate    0.1504 
## relevel(factor(taxonomic_group), ref = "terrestrial invertebrate")fish                    0.2050 
## relevel(factor(taxonomic_group), ref = "terrestrial invertebrate")reptile                 0.0214 
##                                                                                             se 
## intrcpt                                                                                 0.1410 
## relevel(factor(taxonomic_group), ref = "terrestrial invertebrate")amphibian             0.2121 
## relevel(factor(taxonomic_group), ref = "terrestrial invertebrate")aquatic invertebrate  0.0794 
## relevel(factor(taxonomic_group), ref = "terrestrial invertebrate")fish                  0.1731 
## relevel(factor(taxonomic_group), ref = "terrestrial invertebrate")reptile               0.2098 
##                                                                                           tval 
## intrcpt                                                                                 0.3464 
## relevel(factor(taxonomic_group), ref = "terrestrial invertebrate")amphibian             0.6969 
## relevel(factor(taxonomic_group), ref = "terrestrial invertebrate")aquatic invertebrate  1.8938 
## relevel(factor(taxonomic_group), ref = "terrestrial invertebrate")fish                  1.1839 
## relevel(factor(taxonomic_group), ref = "terrestrial invertebrate")reptile               0.1020 
##                                                                                          df 
## intrcpt                                                                                 133 
## relevel(factor(taxonomic_group), ref = "terrestrial invertebrate")amphibian             133 
## relevel(factor(taxonomic_group), ref = "terrestrial invertebrate")aquatic invertebrate  133 
## relevel(factor(taxonomic_group), ref = "terrestrial invertebrate")fish                  133 
## relevel(factor(taxonomic_group), ref = "terrestrial invertebrate")reptile               133 
##                                                                                           pval 
## intrcpt                                                                                 0.7296 
## relevel(factor(taxonomic_group), ref = "terrestrial invertebrate")amphibian             0.4871 
## relevel(factor(taxonomic_group), ref = "terrestrial invertebrate")aquatic invertebrate  0.0604 
## relevel(factor(taxonomic_group), ref = "terrestrial invertebrate")fish                  0.2386 
## relevel(factor(taxonomic_group), ref = "terrestrial invertebrate")reptile               0.9189 
##                                                                                           ci.lb 
## intrcpt                                                                                 -0.2301 
## relevel(factor(taxonomic_group), ref = "terrestrial invertebrate")amphibian             -0.2717 
## relevel(factor(taxonomic_group), ref = "terrestrial invertebrate")aquatic invertebrate  -0.0067 
## relevel(factor(taxonomic_group), ref = "terrestrial invertebrate")fish                  -0.1375 
## relevel(factor(taxonomic_group), ref = "terrestrial invertebrate")reptile               -0.3935 
##                                                                                          ci.ub 
## intrcpt                                                                                 0.3278 
## relevel(factor(taxonomic_group), ref = "terrestrial invertebrate")amphibian             0.5672 
## relevel(factor(taxonomic_group), ref = "terrestrial invertebrate")aquatic invertebrate  0.3074 
## relevel(factor(taxonomic_group), ref = "terrestrial invertebrate")fish                  0.5474 
## relevel(factor(taxonomic_group), ref = "terrestrial invertebrate")reptile               0.4363 
##  
## intrcpt 
## relevel(factor(taxonomic_group), ref = "terrestrial invertebrate")amphibian 
## relevel(factor(taxonomic_group), ref = "terrestrial invertebrate")aquatic invertebrate  . 
## relevel(factor(taxonomic_group), ref = "terrestrial invertebrate")fish 
## relevel(factor(taxonomic_group), ref = "terrestrial invertebrate")reptile 
## 
## ---
## Signif. codes:  0 '***' 0.001 '**' 0.01 '*' 0.05 '.' 0.1 ' ' 1  
       ## Aquatic invertebrates as the reference group  
   mod.taxa_het_cont_ainvert_ref  <-   rma.mv ( yi =  dARR,  V =  VCV_dARR,  mods =   ~  relevel ( factor (taxonomic_group), 
        ref =   "aquatic invertebrate" ),  method =   "REML" ,  test =   "t" ,  dfs =   "contain" , 
        random =   list ( ~  1   |  species_ID,  ~  1   |  phylogeny,  ~ taxonomic_group  |  es_ID),  struct =   "HCS" , 
        rho =   0 ,  R =   list ( phylogeny =  phylo_matrix),  data =  d,  sparse =   TRUE ) 
    summary (mod.taxa_het_cont_ainvert_ref)    
  ## 
## Multivariate Meta-Analysis Model (k = 1089; method: REML)
## 
##   logLik  Deviance       AIC       BIC      AICc  <U+200B> 
## -54.5107  109.0214  133.0214  192.8824  133.3128   
## 
## Variance Components:
## 
##             estim    sqrt  nlvls  fixed      factor    R 
## sigma^2.1  0.0090  0.0946    138     no  species_ID   no 
## sigma^2.2  0.0296  0.1722    138     no   phylogeny  yes 
## 
## outer factor: es_ID           (nlvls = 1089)
## inner factor: taxonomic_group (nlvls = 5)
## 
##             estim    sqrt  k.lvl  fixed                     level 
## tau^2.1    0.1878  0.4334     71     no                 amphibian 
## tau^2.2    0.1357  0.3684    221     no      aquatic invertebrate 
## tau^2.3    0.0329  0.1813    623     no                      fish 
## tau^2.4    0.0163  0.1278     27     no                   reptile 
## tau^2.5    0.0069  0.0833    147     no  terrestrial invertebrate 
## rho        0.0000                   yes                           
## 
## Test for Residual Heterogeneity:
## QE(df = 1084) = 56018.6724, p-val < .0001
## 
## Test of Moderators (coefficients 2:5):
## F(df1 = 4, df2 = 133) = 1.3982, p-val = 0.2380
## 
## Model Results:
## 
##                                                                                         estimate<U+200B> 
## intrcpt                                                                                   0.1992 
## relevel(factor(taxonomic_group), ref = "aquatic invertebrate")amphibian                  -0.0026 
## relevel(factor(taxonomic_group), ref = "aquatic invertebrate")fish                        0.0546 
## relevel(factor(taxonomic_group), ref = "aquatic invertebrate")reptile                    -0.1290 
## relevel(factor(taxonomic_group), ref = "aquatic invertebrate")terrestrial invertebrate   -0.1504 
##                                                                                             se 
## intrcpt                                                                                 0.1286 
## relevel(factor(taxonomic_group), ref = "aquatic invertebrate")amphibian                 0.2026 
## relevel(factor(taxonomic_group), ref = "aquatic invertebrate")fish                      0.1614 
## relevel(factor(taxonomic_group), ref = "aquatic invertebrate")reptile                   0.2002 
## relevel(factor(taxonomic_group), ref = "aquatic invertebrate")terrestrial invertebrate  0.0794 
##                                                                                            tval 
## intrcpt                                                                                  1.5492 
## relevel(factor(taxonomic_group), ref = "aquatic invertebrate")amphibian                 -0.0126 
## relevel(factor(taxonomic_group), ref = "aquatic invertebrate")fish                       0.3385 
## relevel(factor(taxonomic_group), ref = "aquatic invertebrate")reptile                   -0.6442 
## relevel(factor(taxonomic_group), ref = "aquatic invertebrate")terrestrial invertebrate  -1.8938 
##                                                                                          df 
## intrcpt                                                                                 133 
## relevel(factor(taxonomic_group), ref = "aquatic invertebrate")amphibian                 133 
## relevel(factor(taxonomic_group), ref = "aquatic invertebrate")fish                      133 
## relevel(factor(taxonomic_group), ref = "aquatic invertebrate")reptile                   133 
## relevel(factor(taxonomic_group), ref = "aquatic invertebrate")terrestrial invertebrate  133 
##                                                                                           pval 
## intrcpt                                                                                 0.1237 
## relevel(factor(taxonomic_group), ref = "aquatic invertebrate")amphibian                 0.9899 
## relevel(factor(taxonomic_group), ref = "aquatic invertebrate")fish                      0.7355 
## relevel(factor(taxonomic_group), ref = "aquatic invertebrate")reptile                   0.5206 
## relevel(factor(taxonomic_group), ref = "aquatic invertebrate")terrestrial invertebrate  0.0604 
##                                                                                           ci.lb 
## intrcpt                                                                                 -0.0551 
## relevel(factor(taxonomic_group), ref = "aquatic invertebrate")amphibian                 -0.4033 
## relevel(factor(taxonomic_group), ref = "aquatic invertebrate")fish                      -0.2646 
## relevel(factor(taxonomic_group), ref = "aquatic invertebrate")reptile                   -0.5249 
## relevel(factor(taxonomic_group), ref = "aquatic invertebrate")terrestrial invertebrate  -0.3074 
##                                                                                          ci.ub 
## intrcpt                                                                                 0.4535 
## relevel(factor(taxonomic_group), ref = "aquatic invertebrate")amphibian                 0.3981 
## relevel(factor(taxonomic_group), ref = "aquatic invertebrate")fish                      0.3738 
## relevel(factor(taxonomic_group), ref = "aquatic invertebrate")reptile                   0.2670 
## relevel(factor(taxonomic_group), ref = "aquatic invertebrate")terrestrial invertebrate  0.0067 
##  
## intrcpt 
## relevel(factor(taxonomic_group), ref = "aquatic invertebrate")amphibian 
## relevel(factor(taxonomic_group), ref = "aquatic invertebrate")fish 
## relevel(factor(taxonomic_group), ref = "aquatic invertebrate")reptile 
## relevel(factor(taxonomic_group), ref = "aquatic invertebrate")terrestrial invertebrate  . 
## 
## ---
## Signif. codes:  0 '***' 0.001 '**' 0.01 '*' 0.05 '.' 0.1 ' ' 1  
       ## Amphibians as the reference group  
   mod.taxa_het_cont_amph_ref  <-   rma.mv ( yi =  dARR,  V =  VCV_dARR,  mods =   ~  relevel ( factor (taxonomic_group), 
        ref =   "amphibian" ),  method =   "REML" ,  test =   "t" ,  dfs =   "contain" ,  random =   list ( ~  1   |  
       species_ID,  ~  1   |  phylogeny,  ~ taxonomic_group  |  es_ID),  struct =   "HCS" ,  rho =   0 , 
        R =   list ( phylogeny =  phylo_matrix),  data =  d,  sparse =   TRUE ) 
    summary (mod.taxa_het_cont_amph_ref)    
  ## 
## Multivariate Meta-Analysis Model (k = 1089; method: REML)
## 
##   logLik  Deviance       AIC       BIC      AICc  <U+200B> 
## -54.5107  109.0214  133.0214  192.8824  133.3128   
## 
## Variance Components:
## 
##             estim    sqrt  nlvls  fixed      factor    R 
## sigma^2.1  0.0090  0.0946    138     no  species_ID   no 
## sigma^2.2  0.0296  0.1722    138     no   phylogeny  yes 
## 
## outer factor: es_ID           (nlvls = 1089)
## inner factor: taxonomic_group (nlvls = 5)
## 
##             estim    sqrt  k.lvl  fixed                     level 
## tau^2.1    0.1878  0.4334     71     no                 amphibian 
## tau^2.2    0.1357  0.3684    221     no      aquatic invertebrate 
## tau^2.3    0.0329  0.1813    623     no                      fish 
## tau^2.4    0.0163  0.1278     27     no                   reptile 
## tau^2.5    0.0069  0.0833    147     no  terrestrial invertebrate 
## rho        0.0000                   yes                           
## 
## Test for Residual Heterogeneity:
## QE(df = 1084) = 56018.6724, p-val < .0001
## 
## Test of Moderators (coefficients 2:5):
## F(df1 = 4, df2 = 133) = 1.3982, p-val = 0.2380
## 
## Model Results:
## 
##                                                                              estimate<U+200B> 
## intrcpt                                                                        0.1966 
## relevel(factor(taxonomic_group), ref = "amphibian")aquatic invertebrate        0.0026 
## relevel(factor(taxonomic_group), ref = "amphibian")fish                        0.0572 
## relevel(factor(taxonomic_group), ref = "amphibian")reptile                    -0.1264 
## relevel(factor(taxonomic_group), ref = "amphibian")terrestrial invertebrate   -0.1478 
##                                                                                  se 
## intrcpt                                                                      0.1761 
## relevel(factor(taxonomic_group), ref = "amphibian")aquatic invertebrate      0.2026 
## relevel(factor(taxonomic_group), ref = "amphibian")fish                      0.1645 
## relevel(factor(taxonomic_group), ref = "amphibian")reptile                   0.1140 
## relevel(factor(taxonomic_group), ref = "amphibian")terrestrial invertebrate  0.2121 
##                                                                                 tval 
## intrcpt                                                                       1.1167 
## relevel(factor(taxonomic_group), ref = "amphibian")aquatic invertebrate       0.0126 
## relevel(factor(taxonomic_group), ref = "amphibian")fish                       0.3476 
## relevel(factor(taxonomic_group), ref = "amphibian")reptile                   -1.1090 
## relevel(factor(taxonomic_group), ref = "amphibian")terrestrial invertebrate  -0.6969 
##                                                                               df 
## intrcpt                                                                      133 
## relevel(factor(taxonomic_group), ref = "amphibian")aquatic invertebrate      133 
## relevel(factor(taxonomic_group), ref = "amphibian")fish                      133 
## relevel(factor(taxonomic_group), ref = "amphibian")reptile                   133 
## relevel(factor(taxonomic_group), ref = "amphibian")terrestrial invertebrate  133 
##                                                                                pval 
## intrcpt                                                                      0.2661 
## relevel(factor(taxonomic_group), ref = "amphibian")aquatic invertebrate      0.9899 
## relevel(factor(taxonomic_group), ref = "amphibian")fish                      0.7287 
## relevel(factor(taxonomic_group), ref = "amphibian")reptile                   0.2694 
## relevel(factor(taxonomic_group), ref = "amphibian")terrestrial invertebrate  0.4871 
##                                                                                ci.lb 
## intrcpt                                                                      -0.1517 
## relevel(factor(taxonomic_group), ref = "amphibian")aquatic invertebrate      -0.3981 
## relevel(factor(taxonomic_group), ref = "amphibian")fish                      -0.2682 
## relevel(factor(taxonomic_group), ref = "amphibian")reptile                   -0.3518 
## relevel(factor(taxonomic_group), ref = "amphibian")terrestrial invertebrate  -0.5672 
##                                                                               ci.ub 
## intrcpt                                                                      0.5449 
## relevel(factor(taxonomic_group), ref = "amphibian")aquatic invertebrate      0.4033 
## relevel(factor(taxonomic_group), ref = "amphibian")fish                      0.3826 
## relevel(factor(taxonomic_group), ref = "amphibian")reptile                   0.0990 
## relevel(factor(taxonomic_group), ref = "amphibian")terrestrial invertebrate  0.2717 
##  
## intrcpt 
## relevel(factor(taxonomic_group), ref = "amphibian")aquatic invertebrate 
## relevel(factor(taxonomic_group), ref = "amphibian")fish 
## relevel(factor(taxonomic_group), ref = "amphibian")reptile 
## relevel(factor(taxonomic_group), ref = "amphibian")terrestrial invertebrate 
## 
## ---
## Signif. codes:  0 '***' 0.001 '**' 0.01 '*' 0.05 '.' 0.1 ' ' 1  
       ## Fishes as the reference group  
   mod.taxa_het_cont_fish_ref  <-   rma.mv ( yi =  dARR,  V =  VCV_dARR,  mods =   ~  relevel ( factor (taxonomic_group), 
        ref =   "fish" ),  method =   "REML" ,  test =   "t" ,  dfs =   "contain" ,  random =   list ( ~  1   |  
       species_ID,  ~  1   |  phylogeny,  ~ taxonomic_group  |  es_ID),  struct =   "HCS" ,  rho =   0 , 
        R =   list ( phylogeny =  phylo_matrix),  data =  d,  sparse =   TRUE ) 
    summary (mod.taxa_het_cont_fish_ref)    
  ## 
## Multivariate Meta-Analysis Model (k = 1089; method: REML)
## 
##   logLik  Deviance       AIC       BIC      AICc  <U+200B> 
## -54.5107  109.0214  133.0214  192.8824  133.3128   
## 
## Variance Components:
## 
##             estim    sqrt  nlvls  fixed      factor    R 
## sigma^2.1  0.0090  0.0946    138     no  species_ID   no 
## sigma^2.2  0.0296  0.1722    138     no   phylogeny  yes 
## 
## outer factor: es_ID           (nlvls = 1089)
## inner factor: taxonomic_group (nlvls = 5)
## 
##             estim    sqrt  k.lvl  fixed                     level 
## tau^2.1    0.1878  0.4334     71     no                 amphibian 
## tau^2.2    0.1357  0.3684    221     no      aquatic invertebrate 
## tau^2.3    0.0329  0.1813    623     no                      fish 
## tau^2.4    0.0163  0.1278     27     no                   reptile 
## tau^2.5    0.0069  0.0833    147     no  terrestrial invertebrate 
## rho        0.0000                   yes                           
## 
## Test for Residual Heterogeneity:
## QE(df = 1084) = 56018.6724, p-val < .0001
## 
## Test of Moderators (coefficients 2:5):
## F(df1 = 4, df2 = 133) = 1.3982, p-val = 0.2380
## 
## Model Results:
## 
##                                                                         estimate<U+200B> 
## intrcpt                                                                   0.2538 
## relevel(factor(taxonomic_group), ref = "fish")amphibian                  -0.0572 
## relevel(factor(taxonomic_group), ref = "fish")aquatic invertebrate       -0.0546 
## relevel(factor(taxonomic_group), ref = "fish")reptile                    -0.1836 
## relevel(factor(taxonomic_group), ref = "fish")terrestrial invertebrate   -0.2050 
##                                                                             se 
## intrcpt                                                                 0.1265 
## relevel(factor(taxonomic_group), ref = "fish")amphibian                 0.1645 
## relevel(factor(taxonomic_group), ref = "fish")aquatic invertebrate      0.1614 
## relevel(factor(taxonomic_group), ref = "fish")reptile                   0.1615 
## relevel(factor(taxonomic_group), ref = "fish")terrestrial invertebrate  0.1731 
##                                                                            tval 
## intrcpt                                                                  2.0058 
## relevel(factor(taxonomic_group), ref = "fish")amphibian                 -0.3476 
## relevel(factor(taxonomic_group), ref = "fish")aquatic invertebrate      -0.3385 
## relevel(factor(taxonomic_group), ref = "fish")reptile                   -1.1365 
## relevel(factor(taxonomic_group), ref = "fish")terrestrial invertebrate  -1.1839 
##                                                                          df 
## intrcpt                                                                 133 
## relevel(factor(taxonomic_group), ref = "fish")amphibian                 133 
## relevel(factor(taxonomic_group), ref = "fish")aquatic invertebrate      133 
## relevel(factor(taxonomic_group), ref = "fish")reptile                   133 
## relevel(factor(taxonomic_group), ref = "fish")terrestrial invertebrate  133 
##                                                                           pval 
## intrcpt                                                                 0.0469 
## relevel(factor(taxonomic_group), ref = "fish")amphibian                 0.7287 
## relevel(factor(taxonomic_group), ref = "fish")aquatic invertebrate      0.7355 
## relevel(factor(taxonomic_group), ref = "fish")reptile                   0.2578 
## relevel(factor(taxonomic_group), ref = "fish")terrestrial invertebrate  0.2386 
##                                                                           ci.lb 
## intrcpt                                                                  0.0035 
## relevel(factor(taxonomic_group), ref = "fish")amphibian                 -0.3826 
## relevel(factor(taxonomic_group), ref = "fish")aquatic invertebrate      -0.3738 
## relevel(factor(taxonomic_group), ref = "fish")reptile                   -0.5031 
## relevel(factor(taxonomic_group), ref = "fish")terrestrial invertebrate  -0.5474 
##                                                                          ci.ub 
## intrcpt                                                                 0.5041 
## relevel(factor(taxonomic_group), ref = "fish")amphibian                 0.2682 
## relevel(factor(taxonomic_group), ref = "fish")aquatic invertebrate      0.2646 
## relevel(factor(taxonomic_group), ref = "fish")reptile                   0.1359 
## relevel(factor(taxonomic_group), ref = "fish")terrestrial invertebrate  0.1375 
##  
## intrcpt                                                                 * 
## relevel(factor(taxonomic_group), ref = "fish")amphibian 
## relevel(factor(taxonomic_group), ref = "fish")aquatic invertebrate 
## relevel(factor(taxonomic_group), ref = "fish")reptile 
## relevel(factor(taxonomic_group), ref = "fish")terrestrial invertebrate 
## 
## ---
## Signif. codes:  0 '***' 0.001 '**' 0.01 '*' 0.05 '.' 0.1 ' ' 1  
 
 
  
 
 
 
  Persistence of
developmental effects  
 Here the effect sizes estimating the “initial” and “persistent”
effects of developmental temperatures on heat tolerance are
compared. 
 
 Individual coefficients 
      mod.persist  <-   run.model (d,  ~ brought_common_temp  -   1 ) 
    summary (mod.persist)    
  ## 
## Multivariate Meta-Analysis Model (k = 1089; method: REML)
## 
##    logLik   Deviance        AIC        BIC       AICc  <U+200B> 
## -212.9028   425.8057   435.8057   460.7616   435.8612   
## 
## Variance Components:
## 
##             estim    sqrt  nlvls  fixed      factor    R 
## sigma^2.1  0.0064  0.0800    138     no  species_ID   no 
## sigma^2.2  0.0142  0.1193    138     no   phylogeny  yes 
## sigma^2.3  0.0602  0.2454   1089     no       es_ID   no 
## 
## Test for Residual Heterogeneity:
## QE(df = 1087) = 56507.5086, p-val < .0001
## 
## Test of Moderators (coefficients 1:2):
## F(df1 = 2, df2 = 1087) = 19.2928, p-val < .0001
## 
## Model Results:
## 
##                         estimate      se    tval    df    pval    ci.lb   ci.ub<U+200B> 
## brought_common_tempno     0.2214  0.0678  3.2640  1087  0.0011   0.0883  0.3544 
## brought_common_tempyes    0.0394  0.0728  0.5419  1087  0.5880  -0.1033  0.1822 
##  
## brought_common_tempno   ** 
## brought_common_tempyes 
## 
## ---
## Signif. codes:  0 '***' 0.001 '**' 0.01 '*' 0.05 '.' 0.1 ' ' 1  
       mod_results (mod.persist,  mod =   "brought_common_temp" ,  data =  d,  group =   "species_ID" )    
  ##   name   estimate     lowerCL   upperCL    lowerPR   upperPR
## 1   No 0.22135272  0.08828745 0.3544180 -0.3522484 0.7949539
## 2  Yes 0.03943152 -0.10334461 0.1822077 -0.5364998 0.6153629  
       r2_ml (mod.persist)    
  ##    R2_marginal R2_conditional 
##     0.06253941     0.30188023  
       my.orchard (mod.persist,  mod =   "brought_common_temp" ,  xlab =   "dARR" ,  alpha =   0.1 , 
        data =  d,  group =   "species_ID" ,  whisker =   0.07 )    
   
 
 
 Contrasts 
      mod.persist_cont  <-   run.model (d,  ~ brought_common_temp) 
    summary (mod.persist_cont)    
  ## 
## Multivariate Meta-Analysis Model (k = 1089; method: REML)
## 
##    logLik   Deviance        AIC        BIC       AICc  <U+200B> 
## -212.9028   425.8057   435.8057   460.7616   435.8612   
## 
## Variance Components:
## 
##             estim    sqrt  nlvls  fixed      factor    R 
## sigma^2.1  0.0064  0.0800    138     no  species_ID   no 
## sigma^2.2  0.0142  0.1193    138     no   phylogeny  yes 
## sigma^2.3  0.0602  0.2454   1089     no       es_ID   no 
## 
## Test for Residual Heterogeneity:
## QE(df = 1087) = 56507.5086, p-val < .0001
## 
## Test of Moderators (coefficient 2):
## F(df1 = 1, df2 = 1087) = 30.7459, p-val < .0001
## 
## Model Results:
## 
##                         estimate      se     tval    df    pval    ci.lb<U+200B> 
## intrcpt                   0.2214  0.0678   3.2640   136  0.0014   0.0872 
## brought_common_tempyes   -0.1819  0.0328  -5.5449  1087  <.0001  -0.2463 
##                           ci.ub 
## intrcpt                  0.3555   ** 
## brought_common_tempyes  -0.1175  *** 
## 
## ---
## Signif. codes:  0 '***' 0.001 '**' 0.01 '*' 0.05 '.' 0.1 ' ' 1  
 
 
 Heteroscedasticity check 
       qplot ( y =   sqrt ( residuals (mod.persist) ^  2 ),  x =   fitted (mod.persist))  +   geom_point ()  +  
        geom_smooth ( method =   "lm" )  +   geom_hline ( yintercept =   0 ,  colour =   "red" )   # Seems fine     
   
 
 
 Individual coefficients (with heteroscedasticity) 
      mod.persist_het  <-   rma.mv ( yi =  dARR,  V =  VCV_dARR,  mods =   ~ brought_common_temp  -  
        1 ,  method =   "REML" ,  test =   "t" ,  dfs =   "contain" ,  random =   list ( ~  1   |  species_ID, 
        ~  1   |  phylogeny,  ~ brought_common_temp  |  es_ID),  struct =   "HCS" ,  rho =   0 ,  R =   list ( phylogeny =  phylo_matrix), 
        data =  d,  sparse =   TRUE ) 
    AICc (mod.persist)    
  ## [1] 435.8611  
       AICc (mod.persist_het)   # Improved model fit     
  ## [1] 380.4215  
       summary (mod.persist_het)    
  ## 
## Multivariate Meta-Analysis Model (k = 1089; method: REML)
## 
##    logLik   Deviance        AIC        BIC       AICc  <U+200B> 
## -184.1719   368.3439   380.3439   410.2909   380.4216   
## 
## Variance Components:
## 
##             estim    sqrt  nlvls  fixed      factor    R 
## sigma^2.1  0.0059  0.0765    138     no  species_ID   no 
## sigma^2.2  0.0155  0.1245    138     no   phylogeny  yes 
## 
## outer factor: es_ID               (nlvls = 1089)
## inner factor: brought_common_temp (nlvls = 2)
## 
##             estim    sqrt  k.lvl  fixed  level 
## tau^2.1    0.0696  0.2639    866     no     no 
## tau^2.2    0.0210  0.1450    223     no    yes 
## rho        0.0000                   yes        
## 
## Test for Residual Heterogeneity:
## QE(df = 1087) = 56507.5086, p-val < .0001
## 
## Test of Moderators (coefficients 1:2):
## F(df1 = 2, df2 = 1087) = 22.8750, p-val < .0001
## 
## Model Results:
## 
##                         estimate      se    tval    df    pval    ci.lb   ci.ub<U+200B> 
## brought_common_tempno     0.2244  0.0705  3.1811  1087  0.0015   0.0860  0.3628 
## brought_common_tempyes    0.0485  0.0733  0.6626  1087  0.5078  -0.0952  0.1923 
##  
## brought_common_tempno   ** 
## brought_common_tempyes 
## 
## ---
## Signif. codes:  0 '***' 0.001 '**' 0.01 '*' 0.05 '.' 0.1 ' ' 1  
       mod_results (mod.persist_het,  mod =   "brought_common_temp" ,  data =  d,  group =   "species_ID" )    
  ##   name   estimate     lowerCL   upperCL    lowerPR   upperPR
## 1   No 0.22439541  0.08598476 0.3628061 -0.3834860 0.8322768
## 2  Yes 0.04853997 -0.09521006 0.1922900 -0.3802659 0.4773458  
       r2_ml (mod.persist_het)    
  ##    R2_marginal R2_conditional 
##      0.1908900      0.4127618  
       my.orchard (mod.persist_het,  mod =   "brought_common_temp" ,  xlab =   "dARR" ,  alpha =   0.1 , 
        data =  d,  group =   "species_ID" ,  whisker =   0.07 )    
   
 
 
 Contrasts (with heteroscedasticity) 
      mod.persist_het_cont  <-   rma.mv ( yi =  dARR,  V =  VCV_dARR,  mods =   ~ brought_common_temp, 
        method =   "REML" ,  test =   "t" ,  dfs =   "contain" ,  random =   list ( ~  1   |  species_ID, 
            ~  1   |  phylogeny,  ~ brought_common_temp  |  es_ID),  struct =   "HCS" ,  rho =   0 ,  R =   list ( phylogeny =  phylo_matrix), 
        data =  d,  sparse =   TRUE ) 
    
    summary (mod.persist_het_cont)    
  ## 
## Multivariate Meta-Analysis Model (k = 1089; method: REML)
## 
##    logLik   Deviance        AIC        BIC       AICc  <U+200B> 
## -184.1719   368.3439   380.3439   410.2909   380.4216   
## 
## Variance Components:
## 
##             estim    sqrt  nlvls  fixed      factor    R 
## sigma^2.1  0.0059  0.0765    138     no  species_ID   no 
## sigma^2.2  0.0155  0.1245    138     no   phylogeny  yes 
## 
## outer factor: es_ID               (nlvls = 1089)
## inner factor: brought_common_temp (nlvls = 2)
## 
##             estim    sqrt  k.lvl  fixed  level 
## tau^2.1    0.0696  0.2639    866     no     no 
## tau^2.2    0.0210  0.1450    223     no    yes 
## rho        0.0000                   yes        
## 
## Test for Residual Heterogeneity:
## QE(df = 1087) = 56507.5086, p-val < .0001
## 
## Test of Moderators (coefficient 2):
## F(df1 = 1, df2 = 1087) = 39.1689, p-val < .0001
## 
## Model Results:
## 
##                         estimate      se     tval    df    pval    ci.lb<U+200B> 
## intrcpt                   0.2244  0.0705   3.1811   136  0.0018   0.0849 
## brought_common_tempyes   -0.1759  0.0281  -6.2585  1087  <.0001  -0.2310 
##                           ci.ub 
## intrcpt                  0.3639   ** 
## brought_common_tempyes  -0.1207  *** 
## 
## ---
## Signif. codes:  0 '***' 0.001 '**' 0.01 '*' 0.05 '.' 0.1 ' ' 1  
 
 
  
 
 
 
  Life history variation
and persistence  
 Here, the types of experimental designs presented in Figure 2 are
compared to estimate the life history variation and persistence of
developmental effects. 
 
 Individual coefficients 
      mod.design  <-   run.model (d,  ~ exp_design  -   1 ) 
    summary (mod.design)    
  ## 
## Multivariate Meta-Analysis Model (k = 1089; method: REML)
## 
##    logLik   Deviance        AIC        BIC       AICc  <U+200B> 
## -205.9256   411.8513   429.8513   474.7387   430.0190   
## 
## Variance Components:
## 
##             estim    sqrt  nlvls  fixed      factor    R 
## sigma^2.1  0.0059  0.0768    138     no  species_ID   no 
## sigma^2.2  0.0140  0.1182    138     no   phylogeny  yes 
## sigma^2.3  0.0597  0.2444   1089     no       es_ID   no 
## 
## Test for Residual Heterogeneity:
## QE(df = 1083) = 47905.2266, p-val < .0001
## 
## Test of Moderators (coefficients 1:6):
## F(df1 = 6, df2 = 1083) = 9.0167, p-val < .0001
## 
## Model Results:
## 
##              estimate      se     tval    df    pval    ci.lb   ci.ub     <U+200B> 
## exp_designA    0.2219  0.0676   3.2805  1083  0.0011   0.0892  0.3547   ** 
## exp_designB    0.1170  0.0964   1.2139  1083  0.2250  -0.0721  0.3061      
## exp_designC    0.2503  0.0756   3.3123  1083  0.0010   0.1020  0.3986  *** 
## exp_designD   -0.0930  0.0821  -1.1319  1083  0.2579  -0.2541  0.0682      
## exp_designE    0.1057  0.0832   1.2695  1083  0.2045  -0.0577  0.2690      
## exp_designF    0.1131  0.0815   1.3880  1083  0.1654  -0.0468  0.2731      
## 
## ---
## Signif. codes:  0 '***' 0.001 '**' 0.01 '*' 0.05 '.' 0.1 ' ' 1  
       mod_results (mod.design,  mod =   "exp_design" ,  data =  d,  group =   "species_ID" )    
  ##   name    estimate     lowerCL    upperCL    lowerPR   upperPR
## 1    A  0.22192299  0.08918504 0.35466095 -0.3474271 0.7912731
## 2    B  0.11700190 -0.07211415 0.30611795 -0.4680665 0.7020703
## 3    C  0.25030917  0.10202997 0.39858836 -0.3228635 0.8234818
## 4    D -0.09295895 -0.25410017 0.06818226 -0.6695928 0.4836749
## 5    E  0.10567936 -0.05765618 0.26901490 -0.4715716 0.6829303
## 6    F  0.11313922 -0.04680240 0.27308084 -0.4631606 0.6894390  
       r2_ml (mod.design)    
  ##    R2_marginal R2_conditional 
##     0.09918076     0.32410191  
       my.orchard (mod.design,  mod =   "exp_design" ,  xlab =   "dARR" ,  alpha =   0.1 ,  data =  d, 
        group =   "species_ID" ,  whisker =   0.09 )    
   
 
 
 Contrasts 
       ## Design A as the reference group  
   mod.design_cont_A_ref  <-   run.model (d,  ~  relevel ( factor (exp_design),  ref =   "A" )) 
    summary (mod.design_cont_A_ref)    
  ## 
## Multivariate Meta-Analysis Model (k = 1089; method: REML)
## 
##    logLik   Deviance        AIC        BIC       AICc  <U+200B> 
## -205.9256   411.8513   429.8513   474.7387   430.0190   
## 
## Variance Components:
## 
##             estim    sqrt  nlvls  fixed      factor    R 
## sigma^2.1  0.0059  0.0768    138     no  species_ID   no 
## sigma^2.2  0.0140  0.1182    138     no   phylogeny  yes 
## sigma^2.3  0.0597  0.2444   1089     no       es_ID   no 
## 
## Test for Residual Heterogeneity:
## QE(df = 1083) = 47905.2266, p-val < .0001
## 
## Test of Moderators (coefficients 2:6):
## F(df1 = 5, df2 = 1083) = 9.2212, p-val < .0001
## 
## Model Results:
## 
##                                          estimate      se     tval    df<U+200B> 
## intrcpt                                    0.2219  0.0676   3.2805   132 
## relevel(factor(exp_design), ref = "A")B   -0.1049  0.0726  -1.4458  1083 
## relevel(factor(exp_design), ref = "A")C    0.0284  0.0421   0.6750  1083 
## relevel(factor(exp_design), ref = "A")D   -0.3149  0.0513  -6.1329  1083 
## relevel(factor(exp_design), ref = "A")E   -0.1162  0.0530  -2.1921  1083 
## relevel(factor(exp_design), ref = "A")F   -0.1088  0.0503  -2.1613  1083 
##                                            pval    ci.lb    ci.ub 
## intrcpt                                  0.0013   0.0881   0.3557   ** 
## relevel(factor(exp_design), ref = "A")B  0.1485  -0.2473   0.0375      
## relevel(factor(exp_design), ref = "A")C  0.4998  -0.0541   0.1109      
## relevel(factor(exp_design), ref = "A")D  <.0001  -0.4156  -0.2141  *** 
## relevel(factor(exp_design), ref = "A")E  0.0286  -0.2203  -0.0122    * 
## relevel(factor(exp_design), ref = "A")F  0.0309  -0.2075  -0.0100    * 
## 
## ---
## Signif. codes:  0 '***' 0.001 '**' 0.01 '*' 0.05 '.' 0.1 ' ' 1  
       ## Design B as the reference group  
   mod.design_cont_B_ref  <-   run.model (d,  ~  relevel ( factor (exp_design),  ref =   "B" )) 
    summary (mod.design_cont_B_ref)    
  ## 
## Multivariate Meta-Analysis Model (k = 1089; method: REML)
## 
##    logLik   Deviance        AIC        BIC       AICc  <U+200B> 
## -205.9256   411.8513   429.8513   474.7387   430.0190   
## 
## Variance Components:
## 
##             estim    sqrt  nlvls  fixed      factor    R 
## sigma^2.1  0.0059  0.0768    138     no  species_ID   no 
## sigma^2.2  0.0140  0.1182    138     no   phylogeny  yes 
## sigma^2.3  0.0597  0.2444   1089     no       es_ID   no 
## 
## Test for Residual Heterogeneity:
## QE(df = 1083) = 47905.2266, p-val < .0001
## 
## Test of Moderators (coefficients 2:6):
## F(df1 = 5, df2 = 1083) = 9.2212, p-val < .0001
## 
## Model Results:
## 
##                                          estimate      se     tval    df<U+200B> 
## intrcpt                                    0.1170  0.0964   1.2139   132 
## relevel(factor(exp_design), ref = "B")A    0.1049  0.0726   1.4458  1083 
## relevel(factor(exp_design), ref = "B")C    0.1333  0.0789   1.6892  1083 
## relevel(factor(exp_design), ref = "B")D   -0.2100  0.0872  -2.4089  1083 
## relevel(factor(exp_design), ref = "B")E   -0.0113  0.0885  -0.1279  1083 
## relevel(factor(exp_design), ref = "B")F   -0.0039  0.0862  -0.0448  1083 
##                                            pval    ci.lb    ci.ub 
## intrcpt                                  0.2269  -0.0737   0.3077    
## relevel(factor(exp_design), ref = "B")A  0.1485  -0.0375   0.2473    
## relevel(factor(exp_design), ref = "B")C  0.0915  -0.0215   0.2882  . 
## relevel(factor(exp_design), ref = "B")D  0.0162  -0.3810  -0.0389  * 
## relevel(factor(exp_design), ref = "B")E  0.8982  -0.1850   0.1624    
## relevel(factor(exp_design), ref = "B")F  0.9643  -0.1730   0.1653    
## 
## ---
## Signif. codes:  0 '***' 0.001 '**' 0.01 '*' 0.05 '.' 0.1 ' ' 1  
       ## Design C as the reference group  
   mod.design_cont_C_ref  <-   run.model (d,  ~  relevel ( factor (exp_design),  ref =   "C" )) 
    summary (mod.design_cont_C_ref)    
  ## 
## Multivariate Meta-Analysis Model (k = 1089; method: REML)
## 
##    logLik   Deviance        AIC        BIC       AICc  <U+200B> 
## -205.9256   411.8513   429.8513   474.7387   430.0190   
## 
## Variance Components:
## 
##             estim    sqrt  nlvls  fixed      factor    R 
## sigma^2.1  0.0059  0.0768    138     no  species_ID   no 
## sigma^2.2  0.0140  0.1182    138     no   phylogeny  yes 
## sigma^2.3  0.0597  0.2444   1089     no       es_ID   no 
## 
## Test for Residual Heterogeneity:
## QE(df = 1083) = 47905.2266, p-val < .0001
## 
## Test of Moderators (coefficients 2:6):
## F(df1 = 5, df2 = 1083) = 9.2212, p-val < .0001
## 
## Model Results:
## 
##                                          estimate      se     tval    df<U+200B> 
## intrcpt                                    0.2503  0.0756   3.3123   132 
## relevel(factor(exp_design), ref = "C")A   -0.0284  0.0421  -0.6750  1083 
## relevel(factor(exp_design), ref = "C")B   -0.1333  0.0789  -1.6892  1083 
## relevel(factor(exp_design), ref = "C")D   -0.3433  0.0635  -5.4077  1083 
## relevel(factor(exp_design), ref = "C")E   -0.1446  0.0631  -2.2925  1083 
## relevel(factor(exp_design), ref = "C")F   -0.1372  0.0619  -2.2147  1083 
##                                            pval    ci.lb    ci.ub 
## intrcpt                                  0.0012   0.1008   0.3998   ** 
## relevel(factor(exp_design), ref = "C")A  0.4998  -0.1109   0.0541      
## relevel(factor(exp_design), ref = "C")B  0.0915  -0.2882   0.0215    . 
## relevel(factor(exp_design), ref = "C")D  <.0001  -0.4678  -0.2187  *** 
## relevel(factor(exp_design), ref = "C")E  0.0221  -0.2684  -0.0208    * 
## relevel(factor(exp_design), ref = "C")F  0.0270  -0.2587  -0.0156    * 
## 
## ---
## Signif. codes:  0 '***' 0.001 '**' 0.01 '*' 0.05 '.' 0.1 ' ' 1  
       ## Design D as the reference group  
   mod.design_cont_D_ref  <-   run.model (d,  ~  relevel ( factor (exp_design),  ref =   "D" )) 
    summary (mod.design_cont_D_ref)    
  ## 
## Multivariate Meta-Analysis Model (k = 1089; method: REML)
## 
##    logLik   Deviance        AIC        BIC       AICc  <U+200B> 
## -205.9256   411.8513   429.8513   474.7387   430.0190   
## 
## Variance Components:
## 
##             estim    sqrt  nlvls  fixed      factor    R 
## sigma^2.1  0.0059  0.0768    138     no  species_ID   no 
## sigma^2.2  0.0140  0.1182    138     no   phylogeny  yes 
## sigma^2.3  0.0597  0.2444   1089     no       es_ID   no 
## 
## Test for Residual Heterogeneity:
## QE(df = 1083) = 47905.2266, p-val < .0001
## 
## Test of Moderators (coefficients 2:6):
## F(df1 = 5, df2 = 1083) = 9.2212, p-val < .0001
## 
## Model Results:
## 
##                                          estimate      se     tval    df<U+200B> 
## intrcpt                                   -0.0930  0.0821  -1.1319   132 
## relevel(factor(exp_design), ref = "D")A    0.3149  0.0513   6.1329  1083 
## relevel(factor(exp_design), ref = "D")B    0.2100  0.0872   2.4089  1083 
## relevel(factor(exp_design), ref = "D")C    0.3433  0.0635   5.4077  1083 
## relevel(factor(exp_design), ref = "D")E    0.1986  0.0683   2.9104  1083 
## relevel(factor(exp_design), ref = "D")F    0.2061  0.0697   2.9567  1083 
##                                            pval    ci.lb   ci.ub 
## intrcpt                                  0.2597  -0.2554  0.0695      
## relevel(factor(exp_design), ref = "D")A  <.0001   0.2141  0.4156  *** 
## relevel(factor(exp_design), ref = "D")B  0.0162   0.0389  0.3810    * 
## relevel(factor(exp_design), ref = "D")C  <.0001   0.2187  0.4678  *** 
## relevel(factor(exp_design), ref = "D")E  0.0037   0.0647  0.3326   ** 
## relevel(factor(exp_design), ref = "D")F  0.0032   0.0693  0.3429   ** 
## 
## ---
## Signif. codes:  0 '***' 0.001 '**' 0.01 '*' 0.05 '.' 0.1 ' ' 1  
       ## Design E as the reference group  
   mod.design_cont_E_ref  <-   run.model (d,  ~  relevel ( factor (exp_design),  ref =   "E" )) 
    summary (mod.design_cont_E_ref)    
  ## 
## Multivariate Meta-Analysis Model (k = 1089; method: REML)
## 
##    logLik   Deviance        AIC        BIC       AICc  <U+200B> 
## -205.9256   411.8513   429.8513   474.7387   430.0190   
## 
## Variance Components:
## 
##             estim    sqrt  nlvls  fixed      factor    R 
## sigma^2.1  0.0059  0.0768    138     no  species_ID   no 
## sigma^2.2  0.0140  0.1182    138     no   phylogeny  yes 
## sigma^2.3  0.0597  0.2444   1089     no       es_ID   no 
## 
## Test for Residual Heterogeneity:
## QE(df = 1083) = 47905.2266, p-val < .0001
## 
## Test of Moderators (coefficients 2:6):
## F(df1 = 5, df2 = 1083) = 9.2212, p-val < .0001
## 
## Model Results:
## 
##                                          estimate      se     tval    df<U+200B> 
## intrcpt                                    0.1057  0.0832   1.2695   132 
## relevel(factor(exp_design), ref = "E")A    0.1162  0.0530   2.1921  1083 
## relevel(factor(exp_design), ref = "E")B    0.0113  0.0885   0.1279  1083 
## relevel(factor(exp_design), ref = "E")C    0.1446  0.0631   2.2925  1083 
## relevel(factor(exp_design), ref = "E")D   -0.1986  0.0683  -2.9104  1083 
## relevel(factor(exp_design), ref = "E")F    0.0075  0.0683   0.1093  1083 
##                                            pval    ci.lb    ci.ub 
## intrcpt                                  0.2065  -0.0590   0.2703     
## relevel(factor(exp_design), ref = "E")A  0.0286   0.0122   0.2203   * 
## relevel(factor(exp_design), ref = "E")B  0.8982  -0.1624   0.1850     
## relevel(factor(exp_design), ref = "E")C  0.0221   0.0208   0.2684   * 
## relevel(factor(exp_design), ref = "E")D  0.0037  -0.3326  -0.0647  ** 
## relevel(factor(exp_design), ref = "E")F  0.9130  -0.1265   0.1414     
## 
## ---
## Signif. codes:  0 '***' 0.001 '**' 0.01 '*' 0.05 '.' 0.1 ' ' 1  
       ## Design F as the reference group  
   mod.design_cont_F_ref  <-   run.model (d,  ~  relevel ( factor (exp_design),  ref =   "F" )) 
    summary (mod.design_cont_F_ref)    
  ## 
## Multivariate Meta-Analysis Model (k = 1089; method: REML)
## 
##    logLik   Deviance        AIC        BIC       AICc  <U+200B> 
## -205.9256   411.8513   429.8513   474.7387   430.0190   
## 
## Variance Components:
## 
##             estim    sqrt  nlvls  fixed      factor    R 
## sigma^2.1  0.0059  0.0768    138     no  species_ID   no 
## sigma^2.2  0.0140  0.1182    138     no   phylogeny  yes 
## sigma^2.3  0.0597  0.2444   1089     no       es_ID   no 
## 
## Test for Residual Heterogeneity:
## QE(df = 1083) = 47905.2266, p-val < .0001
## 
## Test of Moderators (coefficients 2:6):
## F(df1 = 5, df2 = 1083) = 9.2212, p-val < .0001
## 
## Model Results:
## 
##                                          estimate      se     tval    df<U+200B> 
## intrcpt                                    0.1131  0.0815   1.3880   132 
## relevel(factor(exp_design), ref = "F")A    0.1088  0.0503   2.1613  1083 
## relevel(factor(exp_design), ref = "F")B    0.0039  0.0862   0.0448  1083 
## relevel(factor(exp_design), ref = "F")C    0.1372  0.0619   2.2147  1083 
## relevel(factor(exp_design), ref = "F")D   -0.2061  0.0697  -2.9567  1083 
## relevel(factor(exp_design), ref = "F")E   -0.0075  0.0683  -0.1093  1083 
##                                            pval    ci.lb    ci.ub 
## intrcpt                                  0.1675  -0.0481   0.2744     
## relevel(factor(exp_design), ref = "F")A  0.0309   0.0100   0.2075   * 
## relevel(factor(exp_design), ref = "F")B  0.9643  -0.1653   0.1730     
## relevel(factor(exp_design), ref = "F")C  0.0270   0.0156   0.2587   * 
## relevel(factor(exp_design), ref = "F")D  0.0032  -0.3429  -0.0693  ** 
## relevel(factor(exp_design), ref = "F")E  0.9130  -0.1414   0.1265     
## 
## ---
## Signif. codes:  0 '***' 0.001 '**' 0.01 '*' 0.05 '.' 0.1 ' ' 1  
 
 
 Heteroscedasticity check 
       qplot ( y =   sqrt ( residuals (mod.design) ^  2 ),  x =   fitted (mod.design))  +   geom_point ()  +  
        geom_smooth ( method =   "lm" )  +   geom_hline ( yintercept =   0 ,  colour =   "red" )    
   
 
 
 Individual coefficients (with heteroscedasticity) 
      mod.design_het  <-   rma.mv ( yi =  dARR,  V =  VCV_dARR,  mods =   ~ exp_design  -   1 ,  method =   "REML" , 
        test =   "t" ,  dfs =   "contain" ,  random =   list ( ~  1   |  species_ID,  ~  1   |  phylogeny,  ~ exp_design  |  
           es_ID),  struct =   "HCS" ,  rho =   0 ,  R =   list ( phylogeny =  phylo_matrix),  data =  d, 
        sparse =   TRUE ) 
    AICc (mod.design)    
  ## [1] 430.0181  
       AICc (mod.design_het)   # Better fit     
  ## [1] 274.205  
       summary (mod.design_het)    
  ## 
## Multivariate Meta-Analysis Model (k = 1089; method: REML)
## 
##    logLik   Deviance        AIC        BIC       AICc  <U+200B> 
## -122.9070   245.8140   273.8140   343.6388   274.2072   
## 
## Variance Components:
## 
##             estim    sqrt  nlvls  fixed      factor    R 
## sigma^2.1  0.0064  0.0798    138     no  species_ID   no 
## sigma^2.2  0.0170  0.1306    138     no   phylogeny  yes 
## 
## outer factor: es_ID      (nlvls = 1089)
## inner factor: exp_design (nlvls = 6)
## 
##             estim    sqrt  k.lvl  fixed  level 
## tau^2.1    0.0753  0.2745    700     no      A 
## tau^2.2    0.3209  0.5665     20     no      B 
## tau^2.3    0.0155  0.1244    146     no      C 
## tau^2.4    0.0352  0.1876     92     no      D 
## tau^2.5    0.0134  0.1156     76     no      E 
## tau^2.6    0.0074  0.0859     55     no      F 
## rho        0.0000                   yes        
## 
## Test for Residual Heterogeneity:
## QE(df = 1083) = 47905.2266, p-val < .0001
## 
## Test of Moderators (coefficients 1:6):
## F(df1 = 6, df2 = 1083) = 10.4670, p-val < .0001
## 
## Model Results:
## 
##              estimate      se     tval    df    pval    ci.lb   ci.ub    <U+200B> 
## exp_designA    0.2302  0.0740   3.1108  1083  0.0019   0.0850  0.3755  ** 
## exp_designB    0.0981  0.1570   0.6249  1083  0.5321  -0.2099  0.4062     
## exp_designC    0.2502  0.0782   3.2004  1083  0.0014   0.0968  0.4036  ** 
## exp_designD   -0.0818  0.0849  -0.9630  1083  0.3358  -0.2483  0.0848     
## exp_designE    0.1181  0.0825   1.4316  1083  0.1525  -0.0438  0.2800     
## exp_designF    0.1015  0.0784   1.2956  1083  0.1954  -0.0522  0.2553     
## 
## ---
## Signif. codes:  0 '***' 0.001 '**' 0.01 '*' 0.05 '.' 0.1 ' ' 1  
       mod_results (mod.design_het,  mod =   "exp_design" ,  data =  d,  group =   "species_ID" )    
  ##   name    estimate     lowerCL    upperCL    lowerPR   upperPR
## 1    A  0.23024864  0.08501963 0.37547765 -0.4032517 0.8637490
## 2    B  0.09811306 -0.20993386 0.40615999 -1.0937752 1.2900013
## 3    C  0.25017802  0.09679244 0.40356360 -0.1661248 0.6664808
## 4    D -0.08176060 -0.24834760 0.08482639 -0.5851651 0.4216439
## 5    E  0.11811684 -0.04377422 0.28000790 -0.2915607 0.5277944
## 6    F  0.10153337 -0.05223750 0.25530425 -0.2756146 0.4786813  
       r2_ml (mod.design_het)    
  ##    R2_marginal R2_conditional 
##      0.2705497      0.4689134  
       my.orchard (mod.design_het,  mod =   "exp_design" ,  xlab =   "dARR" ,  alpha =   0.1 ,  data =  d, 
        group =   "species_ID" ,  whisker =   0.09 )    
   
 
 
 Contrasts (with heteroscedasticity) 
       ## Design A as the reference group  
   mod.design_het_cont_A_ref  <-   rma.mv ( yi =  dARR,  V =  VCV_dARR,  mods =   ~  relevel ( factor (exp_design), 
        ref =   "A" ),  method =   "REML" ,  test =   "t" ,  dfs =   "contain" ,  random =   list ( ~  1   |  
       species_ID,  ~  1   |  phylogeny,  ~ exp_design  |  es_ID),  struct =   "HCS" ,  rho =   0 ,  R =   list ( phylogeny =  phylo_matrix), 
        data =  d,  sparse =   TRUE ) 
    summary (mod.design_het_cont_A_ref)    
  ## 
## Multivariate Meta-Analysis Model (k = 1089; method: REML)
## 
##    logLik   Deviance        AIC        BIC       AICc  <U+200B> 
## -122.9070   245.8140   273.8140   343.6388   274.2072   
## 
## Variance Components:
## 
##             estim    sqrt  nlvls  fixed      factor    R 
## sigma^2.1  0.0064  0.0798    138     no  species_ID   no 
## sigma^2.2  0.0170  0.1306    138     no   phylogeny  yes 
## 
## outer factor: es_ID      (nlvls = 1089)
## inner factor: exp_design (nlvls = 6)
## 
##             estim    sqrt  k.lvl  fixed  level 
## tau^2.1    0.0753  0.2745    700     no      A 
## tau^2.2    0.3209  0.5665     20     no      B 
## tau^2.3    0.0155  0.1244    146     no      C 
## tau^2.4    0.0352  0.1876     92     no      D 
## tau^2.5    0.0134  0.1156     76     no      E 
## tau^2.6    0.0074  0.0859     55     no      F 
## rho        0.0000                   yes        
## 
## Test for Residual Heterogeneity:
## QE(df = 1083) = 47905.2266, p-val < .0001
## 
## Test of Moderators (coefficients 2:6):
## F(df1 = 5, df2 = 1083) = 11.2372, p-val < .0001
## 
## Model Results:
## 
##                                          estimate      se     tval    df<U+200B> 
## intrcpt                                    0.2302  0.0740   3.1108   132 
## relevel(factor(exp_design), ref = "A")B   -0.1321  0.1406  -0.9397  1083 
## relevel(factor(exp_design), ref = "A")C    0.0199  0.0368   0.5412  1083 
## relevel(factor(exp_design), ref = "A")D   -0.3120  0.0477  -6.5421  1083 
## relevel(factor(exp_design), ref = "A")E   -0.1121  0.0429  -2.6142  1083 
## relevel(factor(exp_design), ref = "A")F   -0.1287  0.0343  -3.7561  1083 
##                                            pval    ci.lb    ci.ub 
## intrcpt                                  0.0023   0.0838   0.3767   ** 
## relevel(factor(exp_design), ref = "A")B  0.3476  -0.4081   0.1438      
## relevel(factor(exp_design), ref = "A")C  0.5885  -0.0523   0.0922      
## relevel(factor(exp_design), ref = "A")D  <.0001  -0.4056  -0.2184  *** 
## relevel(factor(exp_design), ref = "A")E  0.0091  -0.1963  -0.0280   ** 
## relevel(factor(exp_design), ref = "A")F  0.0002  -0.1960  -0.0615  *** 
## 
## ---
## Signif. codes:  0 '***' 0.001 '**' 0.01 '*' 0.05 '.' 0.1 ' ' 1  
       ## Design B as the reference group  
   mod.design_het_cont_B_ref  <-   rma.mv ( yi =  dARR,  V =  VCV_dARR,  mods =   ~  relevel ( factor (exp_design), 
        ref =   "B" ),  method =   "REML" ,  test =   "t" ,  dfs =   "contain" ,  random =   list ( ~  1   |  
       species_ID,  ~  1   |  phylogeny,  ~ exp_design  |  es_ID),  struct =   "HCS" ,  rho =   0 ,  R =   list ( phylogeny =  phylo_matrix), 
        data =  d,  sparse =   TRUE ) 
    summary (mod.design_het_cont_B_ref)    
  ## 
## Multivariate Meta-Analysis Model (k = 1089; method: REML)
## 
##    logLik   Deviance        AIC        BIC       AICc  <U+200B> 
## -122.9070   245.8140   273.8140   343.6388   274.2072   
## 
## Variance Components:
## 
##             estim    sqrt  nlvls  fixed      factor    R 
## sigma^2.1  0.0064  0.0798    138     no  species_ID   no 
## sigma^2.2  0.0170  0.1306    138     no   phylogeny  yes 
## 
## outer factor: es_ID      (nlvls = 1089)
## inner factor: exp_design (nlvls = 6)
## 
##             estim    sqrt  k.lvl  fixed  level 
## tau^2.1    0.0753  0.2745    700     no      A 
## tau^2.2    0.3209  0.5665     20     no      B 
## tau^2.3    0.0155  0.1244    146     no      C 
## tau^2.4    0.0352  0.1876     92     no      D 
## tau^2.5    0.0134  0.1156     76     no      E 
## tau^2.6    0.0074  0.0859     55     no      F 
## rho        0.0000                   yes        
## 
## Test for Residual Heterogeneity:
## QE(df = 1083) = 47905.2266, p-val < .0001
## 
## Test of Moderators (coefficients 2:6):
## F(df1 = 5, df2 = 1083) = 11.2372, p-val < .0001
## 
## Model Results:
## 
##                                          estimate      se     tval    df<U+200B> 
## intrcpt                                    0.0981  0.1570   0.6249   132 
## relevel(factor(exp_design), ref = "B")A    0.1321  0.1406   0.9397  1083 
## relevel(factor(exp_design), ref = "B")C    0.1521  0.1420   1.0710  1083 
## relevel(factor(exp_design), ref = "B")D   -0.1799  0.1473  -1.2214  1083 
## relevel(factor(exp_design), ref = "B")E    0.0200  0.1458   0.1372  1083 
## relevel(factor(exp_design), ref = "B")F    0.0034  0.1427   0.0240  1083 
##                                            pval    ci.lb   ci.ub 
## intrcpt                                  0.5331  -0.2124  0.4087    
## relevel(factor(exp_design), ref = "B")A  0.3476  -0.1438  0.4081    
## relevel(factor(exp_design), ref = "B")C  0.2844  -0.1265  0.4307    
## relevel(factor(exp_design), ref = "B")D  0.2222  -0.4688  0.1091    
## relevel(factor(exp_design), ref = "B")E  0.8909  -0.2661  0.3061    
## relevel(factor(exp_design), ref = "B")F  0.9809  -0.2765  0.2834    
## 
## ---
## Signif. codes:  0 '***' 0.001 '**' 0.01 '*' 0.05 '.' 0.1 ' ' 1  
       ## Design C as the reference group  
   mod.design_het_cont_C_ref  <-   rma.mv ( yi =  dARR,  V =  VCV_dARR,  mods =   ~  relevel ( factor (exp_design), 
        ref =   "C" ),  method =   "REML" ,  test =   "t" ,  dfs =   "contain" ,  random =   list ( ~  1   |  
       species_ID,  ~  1   |  phylogeny,  ~ exp_design  |  es_ID),  struct =   "HCS" ,  rho =   0 ,  R =   list ( phylogeny =  phylo_matrix), 
        data =  d,  sparse =   TRUE ) 
    summary (mod.design_het_cont_C_ref)    
  ## 
## Multivariate Meta-Analysis Model (k = 1089; method: REML)
## 
##    logLik   Deviance        AIC        BIC       AICc  <U+200B> 
## -122.9070   245.8140   273.8140   343.6388   274.2072   
## 
## Variance Components:
## 
##             estim    sqrt  nlvls  fixed      factor    R 
## sigma^2.1  0.0064  0.0798    138     no  species_ID   no 
## sigma^2.2  0.0170  0.1306    138     no   phylogeny  yes 
## 
## outer factor: es_ID      (nlvls = 1089)
## inner factor: exp_design (nlvls = 6)
## 
##             estim    sqrt  k.lvl  fixed  level 
## tau^2.1    0.0753  0.2745    700     no      A 
## tau^2.2    0.3209  0.5665     20     no      B 
## tau^2.3    0.0155  0.1244    146     no      C 
## tau^2.4    0.0352  0.1876     92     no      D 
## tau^2.5    0.0134  0.1156     76     no      E 
## tau^2.6    0.0074  0.0859     55     no      F 
## rho        0.0000                   yes        
## 
## Test for Residual Heterogeneity:
## QE(df = 1083) = 47905.2266, p-val < .0001
## 
## Test of Moderators (coefficients 2:6):
## F(df1 = 5, df2 = 1083) = 11.2372, p-val < .0001
## 
## Model Results:
## 
##                                          estimate      se     tval    df<U+200B> 
## intrcpt                                    0.2502  0.0782   3.2004   132 
## relevel(factor(exp_design), ref = "C")A   -0.0199  0.0368  -0.5412  1083 
## relevel(factor(exp_design), ref = "C")B   -0.1521  0.1420  -1.0710  1083 
## relevel(factor(exp_design), ref = "C")D   -0.3319  0.0562  -5.9095  1083 
## relevel(factor(exp_design), ref = "C")E   -0.1321  0.0512  -2.5801  1083 
## relevel(factor(exp_design), ref = "C")F   -0.1486  0.0450  -3.3017  1083 
##                                            pval    ci.lb    ci.ub 
## intrcpt                                  0.0017   0.0955   0.4048   ** 
## relevel(factor(exp_design), ref = "C")A  0.5885  -0.0922   0.0523      
## relevel(factor(exp_design), ref = "C")B  0.2844  -0.4307   0.1265      
## relevel(factor(exp_design), ref = "C")D  <.0001  -0.4422  -0.2217  *** 
## relevel(factor(exp_design), ref = "C")E  0.0100  -0.2325  -0.0316    * 
## relevel(factor(exp_design), ref = "C")F  0.0010  -0.2370  -0.0603  *** 
## 
## ---
## Signif. codes:  0 '***' 0.001 '**' 0.01 '*' 0.05 '.' 0.1 ' ' 1  
       ## Design D as the reference group  
   mod.design_het_cont_D_ref  <-   rma.mv ( yi =  dARR,  V =  VCV_dARR,  mods =   ~  relevel ( factor (exp_design), 
        ref =   "D" ),  method =   "REML" ,  test =   "t" ,  dfs =   "contain" ,  random =   list ( ~  1   |  
       species_ID,  ~  1   |  phylogeny,  ~ exp_design  |  es_ID),  struct =   "HCS" ,  rho =   0 ,  R =   list ( phylogeny =  phylo_matrix), 
        data =  d,  sparse =   TRUE ) 
    summary (mod.design_het_cont_D_ref)    
  ## 
## Multivariate Meta-Analysis Model (k = 1089; method: REML)
## 
##    logLik   Deviance        AIC        BIC       AICc  <U+200B> 
## -122.9070   245.8140   273.8140   343.6388   274.2072   
## 
## Variance Components:
## 
##             estim    sqrt  nlvls  fixed      factor    R 
## sigma^2.1  0.0064  0.0798    138     no  species_ID   no 
## sigma^2.2  0.0170  0.1306    138     no   phylogeny  yes 
## 
## outer factor: es_ID      (nlvls = 1089)
## inner factor: exp_design (nlvls = 6)
## 
##             estim    sqrt  k.lvl  fixed  level 
## tau^2.1    0.0753  0.2745    700     no      A 
## tau^2.2    0.3209  0.5665     20     no      B 
## tau^2.3    0.0155  0.1244    146     no      C 
## tau^2.4    0.0352  0.1876     92     no      D 
## tau^2.5    0.0134  0.1156     76     no      E 
## tau^2.6    0.0074  0.0859     55     no      F 
## rho        0.0000                   yes        
## 
## Test for Residual Heterogeneity:
## QE(df = 1083) = 47905.2266, p-val < .0001
## 
## Test of Moderators (coefficients 2:6):
## F(df1 = 5, df2 = 1083) = 11.2372, p-val < .0001
## 
## Model Results:
## 
##                                          estimate      se     tval    df<U+200B> 
## intrcpt                                   -0.0818  0.0849  -0.9630   132 
## relevel(factor(exp_design), ref = "D")A    0.3120  0.0477   6.5421  1083 
## relevel(factor(exp_design), ref = "D")B    0.1799  0.1473   1.2214  1083 
## relevel(factor(exp_design), ref = "D")C    0.3319  0.0562   5.9095  1083 
## relevel(factor(exp_design), ref = "D")E    0.1999  0.0535   3.7378  1083 
## relevel(factor(exp_design), ref = "D")F    0.1833  0.0550   3.3308  1083 
##                                            pval    ci.lb   ci.ub 
## intrcpt                                  0.3373  -0.2497  0.0862      
## relevel(factor(exp_design), ref = "D")A  <.0001   0.2184  0.4056  *** 
## relevel(factor(exp_design), ref = "D")B  0.2222  -0.1091  0.4688      
## relevel(factor(exp_design), ref = "D")C  <.0001   0.2217  0.4422  *** 
## relevel(factor(exp_design), ref = "D")E  0.0002   0.0950  0.3048  *** 
## relevel(factor(exp_design), ref = "D")F  0.0009   0.0753  0.2913  *** 
## 
## ---
## Signif. codes:  0 '***' 0.001 '**' 0.01 '*' 0.05 '.' 0.1 ' ' 1  
       ## Design E as the reference group  
   mod.design_het_cont_E_ref  <-   rma.mv ( yi =  dARR,  V =  VCV_dARR,  mods =   ~  relevel ( factor (exp_design), 
        ref =   "E" ),  method =   "REML" ,  test =   "t" ,  dfs =   "contain" ,  random =   list ( ~  1   |  
       species_ID,  ~  1   |  phylogeny,  ~ exp_design  |  es_ID),  struct =   "HCS" ,  rho =   0 ,  R =   list ( phylogeny =  phylo_matrix), 
        data =  d,  sparse =   TRUE ) 
    summary (mod.design_het_cont_E_ref)    
  ## 
## Multivariate Meta-Analysis Model (k = 1089; method: REML)
## 
##    logLik   Deviance        AIC        BIC       AICc  <U+200B> 
## -122.9070   245.8140   273.8140   343.6388   274.2072   
## 
## Variance Components:
## 
##             estim    sqrt  nlvls  fixed      factor    R 
## sigma^2.1  0.0064  0.0798    138     no  species_ID   no 
## sigma^2.2  0.0170  0.1306    138     no   phylogeny  yes 
## 
## outer factor: es_ID      (nlvls = 1089)
## inner factor: exp_design (nlvls = 6)
## 
##             estim    sqrt  k.lvl  fixed  level 
## tau^2.1    0.0753  0.2745    700     no      A 
## tau^2.2    0.3209  0.5665     20     no      B 
## tau^2.3    0.0155  0.1244    146     no      C 
## tau^2.4    0.0352  0.1876     92     no      D 
## tau^2.5    0.0134  0.1156     76     no      E 
## tau^2.6    0.0074  0.0859     55     no      F 
## rho        0.0000                   yes        
## 
## Test for Residual Heterogeneity:
## QE(df = 1083) = 47905.2266, p-val < .0001
## 
## Test of Moderators (coefficients 2:6):
## F(df1 = 5, df2 = 1083) = 11.2372, p-val < .0001
## 
## Model Results:
## 
##                                          estimate      se     tval    df<U+200B> 
## intrcpt                                    0.1181  0.0825   1.4316   132 
## relevel(factor(exp_design), ref = "E")A    0.1121  0.0429   2.6142  1083 
## relevel(factor(exp_design), ref = "E")B   -0.0200  0.1458  -0.1372  1083 
## relevel(factor(exp_design), ref = "E")C    0.1321  0.0512   2.5801  1083 
## relevel(factor(exp_design), ref = "E")D   -0.1999  0.0535  -3.7378  1083 
## relevel(factor(exp_design), ref = "E")F   -0.0166  0.0485  -0.3418  1083 
##                                            pval    ci.lb    ci.ub 
## intrcpt                                  0.1546  -0.0451   0.2813      
## relevel(factor(exp_design), ref = "E")A  0.0091   0.0280   0.1963   ** 
## relevel(factor(exp_design), ref = "E")B  0.8909  -0.3061   0.2661      
## relevel(factor(exp_design), ref = "E")C  0.0100   0.0316   0.2325    * 
## relevel(factor(exp_design), ref = "E")D  0.0002  -0.3048  -0.0950  *** 
## relevel(factor(exp_design), ref = "E")F  0.7326  -0.1118   0.0786      
## 
## ---
## Signif. codes:  0 '***' 0.001 '**' 0.01 '*' 0.05 '.' 0.1 ' ' 1  
       ## Design F as the reference group  
   mod.design_het_cont_F_ref  <-   rma.mv ( yi =  dARR,  V =  VCV_dARR,  mods =   ~  relevel ( factor (exp_design), 
        ref =   "F" ),  method =   "REML" ,  test =   "t" ,  dfs =   "contain" ,  random =   list ( ~  1   |  
       species_ID,  ~  1   |  phylogeny,  ~ exp_design  |  es_ID),  struct =   "HCS" ,  rho =   0 ,  R =   list ( phylogeny =  phylo_matrix), 
        data =  d,  sparse =   TRUE ) 
    summary (mod.design_het_cont_F_ref)    
  ## 
## Multivariate Meta-Analysis Model (k = 1089; method: REML)
## 
##    logLik   Deviance        AIC        BIC       AICc  <U+200B> 
## -122.9070   245.8140   273.8140   343.6388   274.2072   
## 
## Variance Components:
## 
##             estim    sqrt  nlvls  fixed      factor    R 
## sigma^2.1  0.0064  0.0798    138     no  species_ID   no 
## sigma^2.2  0.0170  0.1306    138     no   phylogeny  yes 
## 
## outer factor: es_ID      (nlvls = 1089)
## inner factor: exp_design (nlvls = 6)
## 
##             estim    sqrt  k.lvl  fixed  level 
## tau^2.1    0.0753  0.2745    700     no      A 
## tau^2.2    0.3209  0.5665     20     no      B 
## tau^2.3    0.0155  0.1244    146     no      C 
## tau^2.4    0.0352  0.1876     92     no      D 
## tau^2.5    0.0134  0.1156     76     no      E 
## tau^2.6    0.0074  0.0859     55     no      F 
## rho        0.0000                   yes        
## 
## Test for Residual Heterogeneity:
## QE(df = 1083) = 47905.2266, p-val < .0001
## 
## Test of Moderators (coefficients 2:6):
## F(df1 = 5, df2 = 1083) = 11.2372, p-val < .0001
## 
## Model Results:
## 
##                                          estimate      se     tval    df<U+200B> 
## intrcpt                                    0.1015  0.0784   1.2956   132 
## relevel(factor(exp_design), ref = "F")A    0.1287  0.0343   3.7561  1083 
## relevel(factor(exp_design), ref = "F")B   -0.0034  0.1427  -0.0240  1083 
## relevel(factor(exp_design), ref = "F")C    0.1486  0.0450   3.3017  1083 
## relevel(factor(exp_design), ref = "F")D   -0.1833  0.0550  -3.3308  1083 
## relevel(factor(exp_design), ref = "F")E    0.0166  0.0485   0.3418  1083 
##                                            pval    ci.lb    ci.ub 
## intrcpt                                  0.1974  -0.0535   0.2566      
## relevel(factor(exp_design), ref = "F")A  0.0002   0.0615   0.1960  *** 
## relevel(factor(exp_design), ref = "F")B  0.9809  -0.2834   0.2765      
## relevel(factor(exp_design), ref = "F")C  0.0010   0.0603   0.2370  *** 
## relevel(factor(exp_design), ref = "F")D  0.0009  -0.2913  -0.0753  *** 
## relevel(factor(exp_design), ref = "F")E  0.7326  -0.0786   0.1118      
## 
## ---
## Signif. codes:  0 '***' 0.001 '**' 0.01 '*' 0.05 '.' 0.1 ' ' 1  
 
 
  
 
 
 
  Time in a common garden
condition  
 
 Run model and plot results 
 We log transformed this variable because it was heavily
right-skewed. 
      mod.common_temp  <-   run.model (d,  ~  scale (time_common_temp,  center =  T,  scale =  F))   # Center to zero mean  
    summary (mod.common_temp)    
  ## 
## Multivariate Meta-Analysis Model (k = 204; method: REML)
## 
##   logLik  Deviance       AIC       BIC      AICc  <U+200B> 
##  24.3962  -48.7923  -38.7923  -22.2510  -38.4862   
## 
## Variance Components:
## 
##             estim    sqrt  nlvls  fixed      factor    R 
## sigma^2.1  0.0038  0.0618     21     no  species_ID   no 
## sigma^2.2  0.0516  0.2273     21     no   phylogeny  yes 
## sigma^2.3  0.0203  0.1425    204     no       es_ID   no 
## 
## Test for Residual Heterogeneity:
## QE(df = 202) = 7121.3816, p-val < .0001
## 
## Test of Moderators (coefficient 2):
## F(df1 = 1, df2 = 202) = 8.9912, p-val = 0.0031
## 
## Model Results:
## 
##                                                 estimate      se    tval   df<U+200B> 
## intrcpt                                           0.0677  0.1324  0.5115   19 
## scale(time_common_temp, center = T, scale = F)    0.0014  0.0005  2.9985  202 
##                                                   pval    ci.lb   ci.ub 
## intrcpt                                         0.6149  -0.2094  0.3449     
## scale(time_common_temp, center = T, scale = F)  0.0031   0.0005  0.0024  ** 
## 
## ---
## Signif. codes:  0 '***' 0.001 '**' 0.01 '*' 0.05 '.' 0.1 ' ' 1  
       r2_ml (mod.common_temp)    
  ##    R2_marginal R2_conditional 
##     0.04787401     0.74470467  
      mod.common_temp_log  <-   run.model (d,  ~  scale ( log (time_common_temp),  center =  T,  scale =  F))   # Center to zero meanand logged  
    summary (mod.common_temp_log)    
  ## 
## Multivariate Meta-Analysis Model (k = 204; method: REML)
## 
##   logLik  Deviance       AIC       BIC      AICc  <U+200B> 
##  21.5624  -43.1248  -33.1248  -16.5834  -32.8186   
## 
## Variance Components:
## 
##             estim    sqrt  nlvls  fixed      factor    R 
## sigma^2.1  0.0007  0.0265     21     no  species_ID   no 
## sigma^2.2  0.0223  0.1493     21     no   phylogeny  yes 
## sigma^2.3  0.0228  0.1509    204     no       es_ID   no 
## 
## Test for Residual Heterogeneity:
## QE(df = 202) = 6434.3906, p-val < .0001
## 
## Test of Moderators (coefficient 2):
## F(df1 = 1, df2 = 202) = 0.4765, p-val = 0.4908
## 
## Model Results:
## 
##                                                      estimate      se     tval<U+200B> 
## intrcpt                                                0.0500  0.0879   0.5691 
## scale(log(time_common_temp), center = T, scale = F)   -0.0087  0.0127  -0.6903 
##                                                       df    pval    ci.lb 
## intrcpt                                               19  0.5759  -0.1340 
## scale(log(time_common_temp), center = T, scale = F)  202  0.4908  -0.0337 
##                                                       ci.ub 
## intrcpt                                              0.2340    
## scale(log(time_common_temp), center = T, scale = F)  0.0162    
## 
## ---
## Signif. codes:  0 '***' 0.001 '**' 0.01 '*' 0.05 '.' 0.1 ' ' 1  
       r2_ml (mod.common_temp_log)    
  ##    R2_marginal R2_conditional 
##     0.00356605     0.50425531  
      d.common_temp  <-   filter (d, time_common_temp  !=   "NA" ) 
    plot_continuous (d.common_temp, mod.common_temp_log, d.common_temp $ time_common_temp, 
        "Time at common temperature (days)" )    
   
 
 
 Heteroscedasticity check 
       qplot ( y =   sqrt ( residuals (mod.common_temp) ^  2 ),  x =   fitted (mod.common_temp))  +   geom_point ()  +  
        geom_smooth ( method =   "lm" )  +   geom_hline ( yintercept =   0 ,  colour =   "red" )   # Seems relatively stable     
   
 
 
  
 
 
 
  Type of metric  
 
 Individual coefficients 
      mod.metric  <-   run.model (d,  ~ metric  -   1 ) 
    summary (mod.metric)    
  ## 
## Multivariate Meta-Analysis Model (k = 1089; method: REML)
## 
##    logLik   Deviance        AIC        BIC       AICc  <U+200B> 
## -225.7190   451.4380   461.4380   486.3939   461.4935   
## 
## Variance Components:
## 
##             estim    sqrt  nlvls  fixed      factor    R 
## sigma^2.1  0.0096  0.0979    138     no  species_ID   no 
## sigma^2.2  0.0257  0.1603    138     no   phylogeny  yes 
## sigma^2.3  0.0604  0.2458   1089     no       es_ID   no 
## 
## Test for Residual Heterogeneity:
## QE(df = 1087) = 63440.4166, p-val < .0001
## 
## Test of Moderators (coefficients 1:2):
## F(df1 = 2, df2 = 1087) = 2.9226, p-val = 0.0542
## 
## Model Results:
## 
##              estimate      se    tval    df    pval    ci.lb   ci.ub   <U+200B> 
## metricCTmax    0.1967  0.0897  2.1930  1087  0.0285   0.0207  0.3727  * 
## metricLT50     0.1646  0.0921  1.7868  1087  0.0743  -0.0162  0.3453  . 
## 
## ---
## Signif. codes:  0 '***' 0.001 '**' 0.01 '*' 0.05 '.' 0.1 ' ' 1  
       mod_results (mod.metric,  mod =   "metric" ,  data =  d,  group =   "species_ID" )    
  ##    name  estimate     lowerCL   upperCL    lowerPR   upperPR
## 1 CTmax 0.1966785  0.02070034 0.3726566 -0.4352999 0.8286569
## 2  LT50 0.1645513 -0.01615094 0.3452536 -0.4687588 0.7978615  
       r2_ml (mod.metric)    
  ##    R2_marginal R2_conditional 
##    0.001772338    0.369636784  
       my.orchard (mod.metric,  mod =   "metric" ,  xlab =   "dARR" ,  alpha =   0.1 ,  data =  d,  group =   "species_ID" , 
        whisker =   0.07 )    
   
 
 
 Contrasts 
      mod.metric_cont  <-   run.model (d,  ~ metric) 
    summary (mod.metric_cont)    
  ## 
## Multivariate Meta-Analysis Model (k = 1089; method: REML)
## 
##    logLik   Deviance        AIC        BIC       AICc  <U+200B> 
## -225.7190   451.4380   461.4380   486.3939   461.4935   
## 
## Variance Components:
## 
##             estim    sqrt  nlvls  fixed      factor    R 
## sigma^2.1  0.0096  0.0979    138     no  species_ID   no 
## sigma^2.2  0.0257  0.1603    138     no   phylogeny  yes 
## sigma^2.3  0.0604  0.2458   1089     no       es_ID   no 
## 
## Test for Residual Heterogeneity:
## QE(df = 1087) = 63440.4166, p-val < .0001
## 
## Test of Moderators (coefficient 2):
## F(df1 = 1, df2 = 1087) = 1.3473, p-val = 0.2460
## 
## Model Results:
## 
##             estimate      se     tval    df    pval    ci.lb   ci.ub   <U+200B> 
## intrcpt       0.1967  0.0897   2.1930   136  0.0300   0.0193  0.3740  * 
## metricLT50   -0.0321  0.0277  -1.1608  1087  0.2460  -0.0864  0.0222    
## 
## ---
## Signif. codes:  0 '***' 0.001 '**' 0.01 '*' 0.05 '.' 0.1 ' ' 1  
 
 
 Heteroscedasticity check 
       qplot ( y =   sqrt ( residuals (mod.metric) ^  2 ),  x =   fitted (mod.metric))  +   geom_point ()  +  
        geom_smooth ( method =   "lm" )  +   geom_hline ( yintercept =   0 ,  colour =   "red" )   # Seems fine     
   
 
 
 Individual coefficients (with heteroscedasticity) 
      mod.metric_het  <-   rma.mv ( yi =  dARR,  V =  VCV_dARR,  mods =   ~ metric  -   1 ,  method =   "REML" , 
        test =   "t" ,  dfs =   "contain" ,  random =   list ( ~  1   |  species_ID,  ~  1   |  phylogeny,  ~ metric  |  
           es_ID),  struct =   "HCS" ,  rho =   0 ,  R =   list ( phylogeny =  phylo_matrix),  data =  d, 
        sparse =   TRUE ) 
    AICc (mod.metric)    
  ## [1] 461.4934  
       AICc (mod.metric_het)   # Better fit     
  ## [1] 454.4918  
       summary (mod.metric_het)    
  ## 
## Multivariate Meta-Analysis Model (k = 1089; method: REML)
## 
##    logLik   Deviance        AIC        BIC       AICc  <U+200B> 
## -221.2071   442.4142   454.4142   484.3612   454.4920   
## 
## Variance Components:
## 
##             estim    sqrt  nlvls  fixed      factor    R 
## sigma^2.1  0.0095  0.0976    138     no  species_ID   no 
## sigma^2.2  0.0263  0.1622    138     no   phylogeny  yes 
## 
## outer factor: es_ID  (nlvls = 1089)
## inner factor: metric (nlvls = 2)
## 
##             estim    sqrt  k.lvl  fixed  level 
## tau^2.1    0.0645  0.2539    863     no  CTmax 
## tau^2.2    0.0409  0.2022    226     no   LT50 
## rho        0.0000                   yes        
## 
## Test for Residual Heterogeneity:
## QE(df = 1087) = 63440.4166, p-val < .0001
## 
## Test of Moderators (coefficients 1:2):
## F(df1 = 2, df2 = 1087) = 2.9452, p-val = 0.0530
## 
## Model Results:
## 
##              estimate      se    tval    df    pval    ci.lb   ci.ub   <U+200B> 
## metricCTmax    0.1945  0.0907  2.1453  1087  0.0321   0.0166  0.3724  * 
## metricLT50     0.1616  0.0925  1.7470  1087  0.0809  -0.0199  0.3430  . 
## 
## ---
## Signif. codes:  0 '***' 0.001 '**' 0.01 '*' 0.05 '.' 0.1 ' ' 1  
       mod_results (mod.metric_het,  mod =   "metric" ,  data =  d,  group =   "species_ID" )    
  ##    name  estimate     lowerCL   upperCL    lowerPR   upperPR
## 1 CTmax 0.1945252  0.01660836 0.3724420 -0.4519017 0.8409521
## 2  LT50 0.1615711 -0.01989991 0.3430421 -0.4114067 0.7345489  
       r2_ml (mod.metric_het)    
  ##    R2_marginal R2_conditional 
##    0.004964216    0.269282977  
       my.orchard (mod.metric_het,  mod =   "metric" ,  xlab =   "dARR" ,  alpha =   0.1 ,  data =  d, 
        group =   "species_ID" ,  whisker =   0.07 )    
   
 
 
 Contrasts (with heteroscedasticity) 
      mod.metric_het_cont  <-   rma.mv ( yi =  dARR,  V =  VCV_dARR,  mods =   ~ metric,  method =   "REML" , 
        test =   "t" ,  dfs =   "contain" ,  random =   list ( ~  1   |  species_ID,  ~  1   |  phylogeny,  ~ metric  |  
           es_ID),  struct =   "HCS" ,  rho =   0 ,  R =   list ( phylogeny =  phylo_matrix),  data =  d, 
        sparse =   TRUE ) 
    summary (mod.metric_het_cont)    
  ## 
## Multivariate Meta-Analysis Model (k = 1089; method: REML)
## 
##    logLik   Deviance        AIC        BIC       AICc  <U+200B> 
## -221.2071   442.4142   454.4142   484.3612   454.4920   
## 
## Variance Components:
## 
##             estim    sqrt  nlvls  fixed      factor    R 
## sigma^2.1  0.0095  0.0976    138     no  species_ID   no 
## sigma^2.2  0.0263  0.1622    138     no   phylogeny  yes 
## 
## outer factor: es_ID  (nlvls = 1089)
## inner factor: metric (nlvls = 2)
## 
##             estim    sqrt  k.lvl  fixed  level 
## tau^2.1    0.0645  0.2539    863     no  CTmax 
## tau^2.2    0.0409  0.2022    226     no   LT50 
## rho        0.0000                   yes        
## 
## Test for Residual Heterogeneity:
## QE(df = 1087) = 63440.4166, p-val < .0001
## 
## Test of Moderators (coefficient 2):
## F(df1 = 1, df2 = 1087) = 1.6464, p-val = 0.1997
## 
## Model Results:
## 
##             estimate      se     tval    df    pval    ci.lb   ci.ub   <U+200B> 
## intrcpt       0.1945  0.0907   2.1453   136  0.0337   0.0152  0.3738  * 
## metricLT50   -0.0330  0.0257  -1.2831  1087  0.1997  -0.0833  0.0174    
## 
## ---
## Signif. codes:  0 '***' 0.001 '**' 0.01 '*' 0.05 '.' 0.1 ' ' 1  
 
 
  
 
 
 
  Heating rate  
 
 Run model and plot results 
      mod.ramping  <-   run.model (d,  ~  scale (ramping,  center =  T,  scale =  F))   # Center to zero mean  
    summary (mod.ramping)    
  ## 
## Multivariate Meta-Analysis Model (k = 855; method: REML)
## 
##    logLik   Deviance        AIC        BIC       AICc  <U+200B> 
## -165.8218   331.6436   341.6436   365.3874   341.7144   
## 
## Variance Components:
## 
##             estim    sqrt  nlvls  fixed      factor    R 
## sigma^2.1  0.0072  0.0847    114     no  species_ID   no 
## sigma^2.2  0.0347  0.1863    114     no   phylogeny  yes 
## sigma^2.3  0.0638  0.2526    855     no       es_ID   no 
## 
## Test for Residual Heterogeneity:
## QE(df = 853) = 55693.6919, p-val < .0001
## 
## Test of Moderators (coefficient 2):
## F(df1 = 1, df2 = 853) = 0.3524, p-val = 0.5529
## 
## Model Results:
## 
##                                        estimate      se    tval   df    pval<U+200B> 
## intrcpt                                  0.2116  0.1045  2.0241  112  0.0453 
## scale(ramping, center = T, scale = F)    0.0194  0.0327  0.5937  853  0.5529 
##                                          ci.lb   ci.ub 
## intrcpt                                 0.0045  0.4187  * 
## scale(ramping, center = T, scale = F)  -0.0448  0.0836    
## 
## ---
## Signif. codes:  0 '***' 0.001 '**' 0.01 '*' 0.05 '.' 0.1 ' ' 1  
       r2_ml (mod.ramping)    
  ##    R2_marginal R2_conditional 
##    0.000681348    0.396621099  
      d.ramping  <-   filter (d, ramping  !=   "NA" ) 
    plot_continuous (d.ramping, mod.ramping, d.ramping $ ramping,  "Heating rate (degrees/min)" )    
   
 
 
 Heteroscedasticity check 
       qplot ( y =   sqrt ( residuals (mod.ramping) ^  2 ),  x =   fitted (mod.ramping))  +   geom_point ()  +  
        geom_smooth ( method =   "lm" )  +   geom_hline ( yintercept =   0 ,  colour =   "red" )    
   
 
 
  
 
 
 
 
  Mean adjusted for habitat differences  
 
 Overall mean with habitat weighted equally 
      mod.adjusted_mean  <-   mod_results ( model =  mod.habitat_het,  data =  d,  mod =   "1" ,  weights =   "equal" , 
        group =   "species_ID" ) 
   mod.adjusted_mean $ mod_table    
  ##      name  estimate     lowerCL   upperCL    lowerPR   upperPR
## 1 Intrcpt 0.1341244 0.002268286 0.2659805 -0.4547758 0.7230246  
       my.orchard (mod.adjusted_mean,  mod =   "1" ,  xlab =   "dARR" ,  alpha =   0.1 ,  data =  d,  group =   "species_ID" , 
        whisker =   0.04 )    
   
 
 
 
  Multi-model inference  
 We used the  MuMin  package to generate all combinations
of moderators, and determine the set of models explaining the most
variation 
       eval (metafor ::: .MuMIn)  # Required for the models to run.   
      
   full_model.MuMIn <-   rma.mv (dARR,  V= VCV_dARR,   
                       method=  "ML" ,  # maximum likelihood for model selection  
                       test=  "t" , 
                       dfs=  "contain" , 
                       mods=   ~  habitat  +   # All moderators without missing values  
                              taxonomic_group  +  
                              metric  +  
                              exp_design, 
                       random=  list ( ~  1  | species_ID,  
                                   ~  1  | phylogeny, 
                                   ~  1  | es_ID), 
                       R =   list ( phylogeny =  phylo_matrix), 
                       data= d, 
                       sparse=  TRUE ) 
    
    options ( na.action =   "na.fail" )  #required for dredge to run  
   candidate_models <-  dredge (full_model.MuMIn)  # Generate all possible combinations of moderators  
    options ( na.action =   "na.omit" )  #set back to default  
    
    subset (candidate_models, delta <=  2 )  # Display all models within 2 values of AICc     
  ## Global model call: rma.mv(yi = dARR, V = VCV_dARR, mods = ~habitat + taxonomic_group + 
##     metric + exp_design, random = list(~1 | species_ID, ~1 | 
##     phylogeny, ~1 | es_ID), data = d, method = "ML", test = "t", 
##     dfs = "contain", R = list(phylogeny = phylo_matrix), sparse = TRUE)
## ---
## Model selection table 
##    (Int) exp_dsg hbt mtr txn_grp df   logLik  AICc delta weight
## 8      +       +   +   +         11 -204.748 431.7  0.00  0.334
## 4      +       +   +             10 -206.212 432.6  0.89  0.214
## 6      +       +       +         10 -206.531 433.3  1.53  0.156
## 2      +       +                  9 -207.569 433.3  1.56  0.153
## 14     +       +       +       + 14 -202.516 433.4  1.68  0.144
## Models ranked by AICc(x)  
       sw ( model.avg (candidate_models,  subset= delta <=  2 )) # relative importance (sum of weights) of the moderators     
  ##                      exp_design metric habitat taxonomic_group
## Sum of weights:      1.00       0.63   0.55    0.14           
## N containing models:    5          3      2       1  
 
 Best models 
 
 Best model 
      best_model <-   run.model (d,  ~  habitat  +   # Paste best model  
                               metric  +  
                               exp_design) 
    
    summary (best_model)  # summary of the best model identified      
  ## 
## Multivariate Meta-Analysis Model (k = 1089; method: REML)
## 
##    logLik   Deviance        AIC        BIC       AICc  ​ 
## -204.1003   408.2006   430.2006   485.0427   430.4476   
## 
## Variance Components:
## 
##             estim    sqrt  nlvls  fixed      factor    R 
## sigma^2.1  0.0063  0.0793    138     no  species_ID   no 
## sigma^2.2  0.0098  0.0992    138     no   phylogeny  yes 
## sigma^2.3  0.0597  0.2444   1089     no       es_ID   no 
## 
## Test for Residual Heterogeneity:
## QE(df = 1081) = 45713.3143, p-val < .0001
## 
## Test of Moderators (coefficients 2:8):
## F(df1 = 7, df2 = 130) = 7.2357, p-val < .0001
## 
## Model Results:
## 
##                     estimate      se     tval    df    pval    ci.lb    ci.ub​ 
## intrcpt               0.2369  0.0583   4.0654   130  <.0001   0.1216   0.3522 
## habitatterrestrial   -0.0632  0.0532  -1.1866   130  0.2375  -0.1685   0.0422 
## metricLT50           -0.0416  0.0275  -1.5106  1081  0.1312  -0.0956   0.0124 
## exp_designB          -0.0820  0.0744  -1.1022  1081  0.2706  -0.2280   0.0640 
## exp_designC           0.0317  0.0422   0.7526  1081  0.4519  -0.0510   0.1145 
## exp_designD          -0.3035  0.0533  -5.6932  1081  <.0001  -0.4081  -0.1989 
## exp_designE          -0.1114  0.0544  -2.0486  1081  0.0407  -0.2182  -0.0047 
## exp_designF          -0.1153  0.0505  -2.2839  1081  0.0226  -0.2144  -0.0162 
##  
## intrcpt             *** 
## habitatterrestrial 
## metricLT50 
## exp_designB 
## exp_designC 
## exp_designD         *** 
## exp_designE           * 
## exp_designF           * 
## 
## ---
## Signif. codes:  0 '***' 0.001 '**' 0.01 '*' 0.05 '.' 0.1 ' ' 1  
       r2_ml (best_model)  # R-squared of the best model      
  ##    R2_marginal R2_conditional 
##      0.1043967      0.2947820  
       # Assumptions  
    
    qplot ( y =   sqrt ( residuals (best_model) ^  2 ),  x =   fitted (best_model))  +  
      geom_point ()  +  
      geom_smooth ( method =   "lm" )  +  
      geom_hline ( yintercept =   0 ,  colour=  "red" )     
   
 
 
 Second best model 
      best_model_n2  <-   run.model (d,  ~ habitat  +  exp_design) 
    
    summary (best_model_n2)    
  ## 
## Multivariate Meta-Analysis Model (k = 1089; method: REML)
## 
##    logLik   Deviance        AIC        BIC       AICc  ​ 
## -205.1119   410.2238   430.2238   480.0894   430.4292   
## 
## Variance Components:
## 
##             estim    sqrt  nlvls  fixed      factor    R 
## sigma^2.1  0.0062  0.0785    138     no  species_ID   no 
## sigma^2.2  0.0104  0.1022    138     no   phylogeny  yes 
## sigma^2.3  0.0598  0.2446   1089     no       es_ID   no 
## 
## Test for Residual Heterogeneity:
## QE(df = 1082) = 45719.9218, p-val < .0001
## 
## Test of Moderators (coefficients 2:7):
## F(df1 = 6, df2 = 131) = 8.0454, p-val < .0001
## 
## Model Results:
## 
##                     estimate      se     tval    df    pval    ci.lb    ci.ub​ 
## intrcpt               0.2283  0.0595   3.8344   131  0.0002   0.1105   0.3461 
## habitatterrestrial   -0.0574  0.0535  -1.0740   131  0.2848  -0.1632   0.0483 
## exp_designB          -0.1068  0.0725  -1.4731  1082  0.1410  -0.2491   0.0355 
## exp_designC           0.0275  0.0420   0.6535  1082  0.5136  -0.0550   0.1099 
## exp_designD          -0.3010  0.0532  -5.6557  1082  <.0001  -0.4055  -0.1966 
## exp_designE          -0.1058  0.0542  -1.9507  1082  0.0514  -0.2122   0.0006 
## exp_designF          -0.1078  0.0503  -2.1443  1082  0.0322  -0.2064  -0.0092 
##  
## intrcpt             *** 
## habitatterrestrial 
## exp_designB 
## exp_designC 
## exp_designD         *** 
## exp_designE           . 
## exp_designF           * 
## 
## ---
## Signif. codes:  0 '***' 0.001 '**' 0.01 '*' 0.05 '.' 0.1 ' ' 1  
       r2_ml (best_model_n2)    
  ##    R2_marginal R2_conditional 
##      0.1030582      0.2979860  
       # Assumptions  
    
    qplot ( y =   sqrt ( residuals (best_model_n2) ^  2 ),  x =   fitted (best_model_n2))  +   geom_point ()  +  
        geom_smooth ( method =   "lm" )  +   geom_hline ( yintercept =   0 ,  colour =   "red" )    
   
 
 
 Third best model 
      best_model_n3  <-   run.model (d,  ~ metric  +  exp_design) 
    
    summary (best_model_n3)    
  ## 
## Multivariate Meta-Analysis Model (k = 1089; method: REML)
## 
##    logLik   Deviance        AIC        BIC       AICc  ​ 
## -205.0087   410.0174   430.0174   479.8831   430.2229   
## 
## Variance Components:
## 
##             estim    sqrt  nlvls  fixed      factor    R 
## sigma^2.1  0.0059  0.0768    138     no  species_ID   no 
## sigma^2.2  0.0144  0.1201    138     no   phylogeny  yes 
## sigma^2.3  0.0597  0.2442   1089     no       es_ID   no 
## 
## Test for Residual Heterogeneity:
## QE(df = 1082) = 47853.3762, p-val < .0001
## 
## Test of Moderators (coefficients 2:7):
## F(df1 = 6, df2 = 1082) = 8.0079, p-val < .0001
## 
## Model Results:
## 
##              estimate      se     tval    df    pval    ci.lb    ci.ub     ​ 
## intrcpt        0.2295  0.0688   3.3358   131  0.0011   0.0934   0.3656   ** 
## metricLT50    -0.0398  0.0275  -1.4456  1082  0.1486  -0.0939   0.0142      
## exp_designB   -0.0808  0.0744  -1.0852  1082  0.2781  -0.2268   0.0653      
## exp_designC    0.0331  0.0422   0.7856  1082  0.4323  -0.0496   0.1159      
## exp_designD   -0.3180  0.0514  -6.1822  1082  <.0001  -0.4190  -0.2171  *** 
## exp_designE   -0.1218  0.0533  -2.2876  1082  0.0224  -0.2263  -0.0173    * 
## exp_designF   -0.1162  0.0506  -2.2957  1082  0.0219  -0.2154  -0.0169    * 
## 
## ---
## Signif. codes:  0 '***' 0.001 '**' 0.01 '*' 0.05 '.' 0.1 ' ' 1  
       r2_ml (best_model_n3)    
  ##    R2_marginal R2_conditional 
##     0.09859645     0.32754533  
       # Assumptions  
    
    qplot ( y =   sqrt ( residuals (best_model_n3) ^  2 ),  x =   fitted (best_model_n3))  +   geom_point ()  +  
        geom_smooth ( method =   "lm" )  +   geom_hline ( yintercept =   0 ,  colour =   "red" )    
   
 
 
 Fourth best model 
      best_model_n4  <-   run.model (d,  ~ exp_design) 
    
    summary (best_model_n4)    
  ## 
## Multivariate Meta-Analysis Model (k = 1089; method: REML)
## 
##    logLik   Deviance        AIC        BIC       AICc  ​ 
## -205.9256   411.8513   429.8513   474.7387   430.0190   
## 
## Variance Components:
## 
##             estim    sqrt  nlvls  fixed      factor    R 
## sigma^2.1  0.0059  0.0768    138     no  species_ID   no 
## sigma^2.2  0.0140  0.1182    138     no   phylogeny  yes 
## sigma^2.3  0.0597  0.2444   1089     no       es_ID   no 
## 
## Test for Residual Heterogeneity:
## QE(df = 1083) = 47905.2266, p-val < .0001
## 
## Test of Moderators (coefficients 2:6):
## F(df1 = 5, df2 = 1083) = 9.2212, p-val < .0001
## 
## Model Results:
## 
##              estimate      se     tval    df    pval    ci.lb    ci.ub     ​ 
## intrcpt        0.2219  0.0676   3.2805   132  0.0013   0.0881   0.3557   ** 
## exp_designB   -0.1049  0.0726  -1.4458  1083  0.1485  -0.2473   0.0375      
## exp_designC    0.0284  0.0421   0.6750  1083  0.4998  -0.0541   0.1109      
## exp_designD   -0.3149  0.0513  -6.1329  1083  <.0001  -0.4156  -0.2141  *** 
## exp_designE   -0.1162  0.0530  -2.1921  1083  0.0286  -0.2203  -0.0122    * 
## exp_designF   -0.1088  0.0503  -2.1613  1083  0.0309  -0.2075  -0.0100    * 
## 
## ---
## Signif. codes:  0 '***' 0.001 '**' 0.01 '*' 0.05 '.' 0.1 ' ' 1  
       r2_ml (best_model_n4)    
  ##    R2_marginal R2_conditional 
##     0.09918076     0.32410191  
       # Assumptions  
    
    qplot ( y =   sqrt ( residuals (best_model_n4) ^  2 ),  x =   fitted (best_model_n4))  +   geom_point ()  +  
        geom_smooth ( method =   "lm" )  +   geom_hline ( yintercept =   0 ,  colour =   "red" )    
   
 
 
 Fifth best model 
      best_model_n5  <-   run.model (d,  ~ exp_design  +  metric  +  taxonomic_group) 
    
    summary (best_model_n5)    
  ## 
## Multivariate Meta-Analysis Model (k = 1089; method: REML)
## 
##    logLik   Deviance        AIC        BIC       AICc  ​ 
## -200.4149   400.8297   428.8297   498.5898   429.2248   
## 
## Variance Components:
## 
##             estim    sqrt  nlvls  fixed      factor    R 
## sigma^2.1  0.0053  0.0730    138     no  species_ID   no 
## sigma^2.2  0.0165  0.1283    138     no   phylogeny  yes 
## sigma^2.3  0.0597  0.2444   1089     no       es_ID   no 
## 
## Test for Residual Heterogeneity:
## QE(df = 1078) = 43868.7717, p-val < .0001
## 
## Test of Moderators (coefficients 2:11):
## F(df1 = 10, df2 = 127) = 5.2608, p-val < .0001
## 
## Model Results:
## 
##                                          estimate      se     tval    df​ 
## intrcpt                                    0.1951  0.1293   1.5084   127 
## exp_designB                               -0.0785  0.0744  -1.0548  1078 
## exp_designC                                0.0390  0.0420   0.9279  1078 
## exp_designD                               -0.3164  0.0527  -6.0025  1078 
## exp_designE                               -0.0976  0.0541  -1.8050  1078 
## exp_designF                               -0.1097  0.0507  -2.1612  1078 
## metricLT50                                -0.0428  0.0276  -1.5502  1078 
## taxonomic_groupaquatic invertebrate        0.0411  0.1494   0.2752   127 
## taxonomic_groupfish                        0.0688  0.1222   0.5626   127 
## taxonomic_groupreptile                     0.0196  0.0980   0.1998   127 
## taxonomic_groupterrestrial invertebrate   -0.0836  0.1591  -0.5253   127 
##                                            pval    ci.lb    ci.ub 
## intrcpt                                  0.1339  -0.0608   0.4510      
## exp_designB                              0.2917  -0.2246   0.0675      
## exp_designC                              0.3537  -0.0435   0.1214      
## exp_designD                              <.0001  -0.4199  -0.2130  *** 
## exp_designE                              0.0714  -0.2038   0.0085    . 
## exp_designF                              0.0309  -0.2092  -0.0101    * 
## metricLT50                               0.1214  -0.0970   0.0114      
## taxonomic_groupaquatic invertebrate      0.7836  -0.2545   0.3367      
## taxonomic_groupfish                      0.5747  -0.1731   0.3106      
## taxonomic_groupreptile                   0.8419  -0.1744   0.2136      
## taxonomic_groupterrestrial invertebrate  0.6003  -0.3985   0.2313      
## 
## ---
## Signif. codes:  0 '***' 0.001 '**' 0.01 '*' 0.05 '.' 0.1 ' ' 1  
       r2_ml (best_model_n5)    
  ##    R2_marginal R2_conditional 
##      0.1120662      0.3493867  
       # Assumptions  
    
    qplot ( y =   sqrt ( residuals (best_model_n5) ^  2 ),  x =   fitted (best_model_n5))  +   geom_point ()  +  
        geom_smooth ( method =   "lm" )  +   geom_hline ( yintercept =   0 ,  colour =   "red" )    
   
 
 
  
 
 
 
 
  Publication bias  
 
 Funnel plot 
       funnel (int_model,  
          yaxis=  "seinv" ,  # Inverse of standard error (precision) as the y axis  
          level =   c ( 90 ,  95 ),   # levels of statistical significance highlighted   
          shade =   c ( "white" ,  "gray55" ),  # shades for different levels of statistical significance  
          legend =   TRUE ,  # display legend  
          ylab=  "Precision (1/SE)" ,  
          cex.lab=  1.5 ,  
          digits=  1 ,  
          xlim=  c ( -  1.8 , 1.8 ), 
          col=  ifelse (d $ imputed ==  "no" ,  "blue" ,  "red" ))  #imputed values in red, original values in blue     
   
 
 
 Data type 
 Whether the data was  published  (reported in the
publication) or  unpublished  (provided by authors) 
 
 Run model and plot results 
      mod.data_type  <-   run.model (d,  ~ data_type  -   1 ) 
    
    summary (mod.data_type)    
  ## 
## Multivariate Meta-Analysis Model (k = 1089; method: REML)
## 
##    logLik   Deviance        AIC        BIC       AICc  ​ 
## -226.2466   452.4933   462.4933   487.4492   462.5488   
## 
## Variance Components:
## 
##             estim    sqrt  nlvls  fixed      factor    R 
## sigma^2.1  0.0096  0.0978    138     no  species_ID   no 
## sigma^2.2  0.0241  0.1553    138     no   phylogeny  yes 
## sigma^2.3  0.0606  0.2461   1089     no       es_ID   no 
## 
## Test for Residual Heterogeneity:
## QE(df = 1087) = 58602.4405, p-val < .0001
## 
## Test of Moderators (coefficients 1:2):
## F(df1 = 2, df2 = 1087) = 2.4945, p-val = 0.0830
## 
## Model Results:
## 
##                     estimate      se    tval    df    pval    ci.lb   ci.ub   ​ 
## data_typeobtained     0.1692  0.0973  1.7380  1087  0.0825  -0.0218  0.3602  . 
## data_typepublished    0.1912  0.0869  2.1997  1087  0.0280   0.0206  0.3617  * 
## 
## ---
## Signif. codes:  0 '***' 0.001 '**' 0.01 '*' 0.05 '.' 0.1 ' ' 1  
       my.orchard (mod.data_type,  mod =   "data_type" ,  xlab =   "dARR" ,  alpha =   0.1 ,  data =  d, 
        group =   "species_ID" ,  whisker =   0.07 )    
   
 
 
 Heteroscedasticity check 
       qplot ( y =   sqrt ( residuals (mod.data_type) ^  2 ),  x =   fitted (mod.data_type))  +   # plot sqrt(residuals^2) against fitted values  
          geom_point ()  +  
          geom_smooth ( method =   "lm" )  +   # method ="lm" to generate a straight line   
          geom_hline ( yintercept =   0 ,  colour=  "red" )     
   
 
 
 Contrasts 
      mod.data_type_cont  <-   run.model (d,  ~ data_type) 
    
    summary (mod.data_type_cont)    
  ## 
## Multivariate Meta-Analysis Model (k = 1089; method: REML)
## 
##    logLik   Deviance        AIC        BIC       AICc  ​ 
## -226.2466   452.4933   462.4933   487.4492   462.5488   
## 
## Variance Components:
## 
##             estim    sqrt  nlvls  fixed      factor    R 
## sigma^2.1  0.0096  0.0978    138     no  species_ID   no 
## sigma^2.2  0.0241  0.1553    138     no   phylogeny  yes 
## sigma^2.3  0.0606  0.2461   1089     no       es_ID   no 
## 
## Test for Residual Heterogeneity:
## QE(df = 1087) = 58602.4405, p-val < .0001
## 
## Test of Moderators (coefficient 2):
## F(df1 = 1, df2 = 1087) = 0.2176, p-val = 0.6409
## 
## Model Results:
## 
##                     estimate      se    tval    df    pval    ci.lb   ci.ub   ​ 
## intrcpt               0.1692  0.0973  1.7380   136  0.0845  -0.0233  0.3617  . 
## data_typepublished    0.0220  0.0471  0.4665  1087  0.6409  -0.0705  0.1144    
## 
## ---
## Signif. codes:  0 '***' 0.001 '**' 0.01 '*' 0.05 '.' 0.1 ' ' 1  
 
 
 
 Publication type 
 Whether the data was  peer-reviewed  or
 not-peer-reviewed  (PhD or MSc dissertations) 
 
 Run model and plot results 
      mod.pub_type  <-   run.model (d,  ~ peer.reviewed  -   1 ) 
    
    summary (mod.pub_type)    
  ## 
## Multivariate Meta-Analysis Model (k = 1089; method: REML)
## 
##    logLik   Deviance        AIC        BIC       AICc 
## -225.1086   450.2173   460.2173   485.1731   460.2728   
## 
## Variance Components:
## 
##             estim    sqrt  nlvls  fixed      factor    R 
## sigma^2.1  0.0092  0.0960    138     no  species_ID   no 
## sigma^2.2  0.0234  0.1530    138     no   phylogeny  yes 
## sigma^2.3  0.0606  0.2462   1089     no       es_ID   no 
## 
## Test for Residual Heterogeneity:
## QE(df = 1087) = 57727.1219, p-val < .0001
## 
## Test of Moderators (coefficients 1:2):
## F(df1 = 2, df2 = 1087) = 3.5577, p-val = 0.0288
## 
## Model Results:
## 
##                                 estimate      se    tval    df    pval    ci.lb 
## peer.reviewednot-peer-reviewed    0.1332  0.0937  1.4214  1087  0.1555  -0.0507 
## peer.reviewedpeer-reviewed        0.1962  0.0857  2.2886  1087  0.0223   0.0280 
##                                  ci.ub 
## peer.reviewednot-peer-reviewed  0.3170    
## peer.reviewedpeer-reviewed      0.3645  * 
## 
## ---
## Signif. codes:  0 '***' 0.001 '**' 0.01 '*' 0.05 '.' 0.1 ' ' 1  
       my.orchard (mod.pub_type,  mod =   "peer.reviewed" ,  xlab =   "dARR" ,  alpha =   0.1 ,  data =  d, 
        group =   "species_ID" ,  whisker =   0.07 )    
   
 
 
 Heteroscedasticity check 
       qplot ( y =   sqrt ( residuals (mod.pub_type) ^  2 ),  x =   fitted (mod.pub_type))  +   # plot sqrt(residuals^2) against fitted values  
          geom_point ()  +  
          geom_smooth ( method =   "lm" )  +   # method ="lm" to generate a straight line   
          geom_hline ( yintercept =   0 ,  colour=  "red" )     
   
 
 
 Contrasts 
      mod.pub_type_cont  <-   run.model (d,  ~ peer.reviewed) 
    
    summary (mod.pub_type_cont)    
  ## 
## Multivariate Meta-Analysis Model (k = 1089; method: REML)
## 
##    logLik   Deviance        AIC        BIC       AICc  ​ 
## -225.1086   450.2173   460.2173   485.1731   460.2728   
## 
## Variance Components:
## 
##             estim    sqrt  nlvls  fixed      factor    R 
## sigma^2.1  0.0092  0.0960    138     no  species_ID   no 
## sigma^2.2  0.0234  0.1530    138     no   phylogeny  yes 
## sigma^2.3  0.0606  0.2462   1089     no       es_ID   no 
## 
## Test for Residual Heterogeneity:
## QE(df = 1087) = 57727.1219, p-val < .0001
## 
## Test of Moderators (coefficient 2):
## F(df1 = 1, df2 = 1087) = 2.2070, p-val = 0.1377
## 
## Model Results:
## 
##                             estimate      se    tval    df    pval    ci.lb​ 
## intrcpt                       0.1332  0.0937  1.4214   136  0.1575  -0.0521 
## peer.reviewedpeer-reviewed    0.0630  0.0424  1.4856  1087  0.1377  -0.0202 
##                              ci.ub 
## intrcpt                     0.3185    
## peer.reviewedpeer-reviewed  0.1463    
## 
## ---
## Signif. codes:  0 '***' 0.001 '**' 0.01 '*' 0.05 '.' 0.1 ' ' 1  
 
 
 
 Publication year 
 Test for time-lag bias 
 
 Run model and plot results 
      mod.pub_year  <-   run.model (d,  ~  scale (pub_year,  center =  T,  scale =  F))   # Center to zero mean  
    
    summary (mod.pub_year)    
  ## 
## Multivariate Meta-Analysis Model (k = 1089; method: REML)
## 
##    logLik   Deviance        AIC        BIC       AICc  ​ 
## -225.9969   451.9939   461.9939   486.9497   462.0494   
## 
## Variance Components:
## 
##             estim    sqrt  nlvls  fixed      factor    R 
## sigma^2.1  0.0095  0.0976    138     no  species_ID   no 
## sigma^2.2  0.0250  0.1581    138     no   phylogeny  yes 
## sigma^2.3  0.0605  0.2460   1089     no       es_ID   no 
## 
## Test for Residual Heterogeneity:
## QE(df = 1087) = 61209.7562, p-val < .0001
## 
## Test of Moderators (coefficient 2):
## F(df1 = 1, df2 = 1087) = 0.1047, p-val = 0.7463
## 
## Model Results:
## 
##                                         estimate      se     tval    df    pval​ 
## intrcpt                                   0.1913  0.0885   2.1621   136  0.0324 
## scale(pub_year, center = T, scale = F)   -0.0004  0.0013  -0.3236  1087  0.7463 
##                                           ci.lb   ci.ub 
## intrcpt                                  0.0163  0.3663  * 
## scale(pub_year, center = T, scale = F)  -0.0029  0.0021    
## 
## ---
## Signif. codes:  0 '***' 0.001 '**' 0.01 '*' 0.05 '.' 0.1 ' ' 1  
       plot_continuous (d, mod.pub_year, d $ pub_year,  "Publication year" )    
   
 
 
 Heteroscedasticity check 
       qplot ( y =   sqrt ( residuals (mod.pub_year) ^  2 ),  x =   fitted (mod.pub_year))  +   # plot sqrt(residuals^2) against fitted values  
          geom_point ()  +  
          geom_smooth ( method =   "lm" )  +   # method ="lm" to generate a straight line   
          geom_hline ( yintercept =   0 ,  colour=  "red" )     
   
 
 
 
 Egger’s regression 
 We performed an Egger’s regression by fitting standard error
(sqrt(Var_dARR)) as a moderator 
      mod.Egger  <-   run.model (d,  ~  sqrt (Var_dARR))   # standard error = sqrt(Var_dARRD)  
    summary (mod.Egger)    
  ## 
## Multivariate Meta-Analysis Model (k = 1089; method: REML)
## 
##    logLik   Deviance        AIC        BIC       AICc  ​ 
## -224.7914   449.5829   459.5829   484.5387   459.6384   
## 
## Variance Components:
## 
##             estim    sqrt  nlvls  fixed      factor    R 
## sigma^2.1  0.0096  0.0979    138     no  species_ID   no 
## sigma^2.2  0.0248  0.1574    138     no   phylogeny  yes 
## sigma^2.3  0.0605  0.2460   1089     no       es_ID   no 
## 
## Test for Residual Heterogeneity:
## QE(df = 1087) = 63292.8875, p-val < .0001
## 
## Test of Moderators (coefficient 2):
## F(df1 = 1, df2 = 1087) = 0.0853, p-val = 0.7703
## 
## Model Results:
## 
##                 estimate      se     tval    df    pval    ci.lb   ci.ub   ​ 
## intrcpt           0.1930  0.0887   2.1759   136  0.0313   0.0176  0.3685  * 
## sqrt(Var_dARR)   -0.0396  0.1356  -0.2921  1087  0.7703  -0.3056  0.2264    
## 
## ---
## Signif. codes:  0 '***' 0.001 '**' 0.01 '*' 0.05 '.' 0.1 ' ' 1  
      pred <-  predict.rma (mod.Egger) 
    
   d    %>%   mutate ( fit= pred $ pred,  
                   ci.lb= pred $ ci.lb, 
                   ci.ub= pred $ ci.ub, 
                   pr.lb= pred $ cr.lb, 
                   pr.ub= pred $ cr.ub)  %>%   
    ggplot ( aes ( x =   sqrt (Var_dARR),  y =  dARR))  +  
         geom_ribbon ( aes ( ymin =  pr.lb,  ymax =  pr.ub,  color =   NULL ),  alpha =  . 1 )  +  
         geom_ribbon ( aes ( ymin =  ci.lb,  ymax =  ci.ub,  color =   NULL ),  alpha =  . 3 )  +  
         geom_point ( size=  2 , shape=  21 ,  alpha=  0.7 ,  fill=  "sienna1" ,  col=  "gray25" , stroke=  1 )  +  
         geom_line ( aes ( y =  fit),  size =   1.2 ) +    
      labs ( x =   "Standard error" ,  y =   "dARR" )  +  
      theme_bw ()  +  
      geom_hline ( yintercept =   0 , linetype =   2 ,  colour =   "black" , alpha=  0.5 ) +     # horizontal line at lnRR = 0  
      theme ( text =   element_text ( size =   18 ,  colour =   "black" ,  hjust =   0.5 ),  # change font sizes and legend position  
              legend.text=  element_text ( size=  14 ), 
              legend.position=  c ( 0 , 0 ),  
              legend.justification =   c ( 0 , 0 ), 
              legend.background =   element_blank (),  
              legend.direction=  "horizontal" , 
              legend.title =   element_text ( size=  15 ),  
              panel.border=  element_rect ( colour=  "black" ,  fill=  NA ,  size=  1.2 ))    
   
 
 
 Sampling variance fitted as a moderator 
      mod.var  <-   run.model (d,  ~ Var_dARR)   
    summary (mod.var)    
  ## 
## Multivariate Meta-Analysis Model (k = 1089; method: REML)
## 
##    logLik   Deviance        AIC        BIC       AICc  ​ 
## -223.6832   447.3664   457.3664   482.3223   457.4219   
## 
## Variance Components:
## 
##             estim    sqrt  nlvls  fixed      factor    R 
## sigma^2.1  0.0095  0.0975    138     no  species_ID   no 
## sigma^2.2  0.0247  0.1572    138     no   phylogeny  yes 
## sigma^2.3  0.0605  0.2460   1089     no       es_ID   no 
## 
## Test for Residual Heterogeneity:
## QE(df = 1087) = 63441.4554, p-val < .0001
## 
## Test of Moderators (coefficient 2):
## F(df1 = 1, df2 = 1087) = 0.1562, p-val = 0.6928
## 
## Model Results:
## 
##           estimate      se     tval    df    pval    ci.lb   ci.ub   ​ 
## intrcpt     0.1903  0.0879   2.1653   136  0.0321   0.0165  0.3641  * 
## Var_dARR   -0.0341  0.0863  -0.3952  1087  0.6928  -0.2034  0.1352    
## 
## ---
## Signif. codes:  0 '***' 0.001 '**' 0.01 '*' 0.05 '.' 0.1 ' ' 1  
      pred <-  predict.rma (mod.var) 
    
   d    %>%   mutate ( fit= pred $ pred,  
                   ci.lb= pred $ ci.lb, 
                   ci.ub= pred $ ci.ub, 
                   pr.lb= pred $ cr.lb, 
                   pr.ub= pred $ cr.ub)  %>%   
    ggplot ( aes ( x =  Var_dARR,  y =  dARR))  +  
         geom_ribbon ( aes ( ymin =  pr.lb,  ymax =  pr.ub,  color =   NULL ),  alpha =  . 1 )  +  
         geom_ribbon ( aes ( ymin =  ci.lb,  ymax =  ci.ub,  color =   NULL ),  alpha =  . 3 )  +  
         geom_point ( size=  2 , shape=  21 ,  alpha=  0.7 ,  fill=  "sienna1" ,  col=  "gray25" , stroke=  1 )  +  
         geom_line ( aes ( y =  fit),  size =   1.2 ) +    
      labs ( x =   "Sampling variance" ,  y =   "dARR" )  +  
      theme_bw ()  +  
      geom_hline ( yintercept =   0 , linetype =   2 ,  colour =   "black" , alpha=  0.5 ) +     # horizontal line at lnRR = 0  
      theme ( text =   element_text ( size =   18 ,  colour =   "black" ,  hjust =   0.5 ),  # change font sizes and legend position  
              legend.text=  element_text ( size=  14 ), 
              legend.position=  c ( 0 , 0 ),  
              legend.justification =   c ( 0 , 0 ), 
              legend.background =   element_blank (),  
              legend.direction=  "horizontal" , 
              legend.title =   element_text ( size=  15 ),  
              panel.border=  element_rect ( colour=  "black" ,  fill=  NA ,  size=  1.2 ))    
   
 
 
 
  Sensitivity analyses  
 
  Leave-one-out
analyses  
 Here, we iteratively removed one study or one species at a time and
investigated how it affected the overall mean. We also tried to
iteratively remove one independent comparison (shared_trt_ID) because it
was too computationally extensive. 
 
 Leave one study out 
      d $ study_ID <-  as.factor (d $ study_ID) 
   d <-  as.data.frame (d)  # Only work with a dataframe  
   VCV_matrix <-  list ()  # will need new VCV matrices because the sample size will be iteratively reduced  
   Leave1studyout <-  list ()  # create a list that will host the results of each model   
    for (i  in   1  :  length ( levels (d $ study_ID))){  # N models = N studies   
     VCV_matrix[[i]] <-  make_VCV_matrix (d[d $ study_ID  !=   levels (d $ study_ID)[i], ],  V=  "Var_dARR" ,  cluster=  "shared_trt_ID" ,  obs=  "es_ID" )  # Create a new VCV matrix for each new model  
     Leave1studyout[[i]]  <-   rma.mv ( yi =  dARR,  V =  VCV_matrix[[i]],  # Same model structure as all the models we fitted  
                                    method=  "REML" , 
                                    test=  "t" , 
                                    dfs=  "contain" , 
                                    random =   list ( ~  1  | species_ID, 
                                                  ~  1  | phylogeny,  
                                                  ~  1  | es_ID), 
                                    R=   list ( phylogeny =  phylo_matrix),  
                                    data =  d[d $ study_ID  !=   levels (d $ study_ID)[i], ], 
                                    sparse=  TRUE )  # Generate a new model for each new data (iterative removal of one study at a time)  
   } 
     
    
    
    # The output is a list so we need to summarise the coefficients of all the models performed  
    
   results.Leave1studyout <-  as.data.frame ( cbind ( 
                                               sapply (Leave1studyout,  function (x)  summary (x) $ beta),  # extract the beta coefficient from all models  
                                               sapply (Leave1studyout,  function (x)  summary (x) $ se),  # extract the standard error from all models  
                                               sapply (Leave1studyout,  function (x)  summary (x) $ zval),   # extract the z value from all models  
                                               sapply (Leave1studyout,  function (x)  summary (x) $ pval),  # extract the p value from all models  
                                               sapply (Leave1studyout,  function (x)  summary (x) $ ci.lb),  # extract the lower confidence interval for all models  
                                               sapply (Leave1studyout,  function (x)  summary (x) $ ci.ub)))  # extract the upper confidence interval for all models  
    
    colnames (results.Leave1studyout) =  c ( "Estimate" ,  "SE" ,  "zval" ,  "pval" ,  "ci.lb" ,  "ci.ub" )  # change column names   
    kable (results.Leave1studyout) %>%   kable_styling ( "striped" ,  position=  "left" )  %>%   scroll_box ( width=  "100%" ,  height=  "500px" )  # Table of the results from all models  
    
    ggplot (results.Leave1studyout) +  stat_dots ( aes ( x= Estimate),  alpha=  0.8 ,  dotsize=  1.5 ,  shape=  16 ,  show.legend= F,  col=  "darkcyan" ) 
    
   results.Leave1studyout  %>%   
                              summarise ( estimate=   mean (Estimate),  # calculate the mean coefficients across the models generated  
                                        se=  mean (SE),  
                                        zval=  mean (zval),  
                                        pval=  mean (pval),  
                                        ci.lb=  mean (ci.lb),  
                                        ci.ub=  mean (ci.ub))  %>%   
                                                              kable () %>%   kable_styling ( "striped" ,  position=  "left" )    
       load ( here ( "Rdata" ,  "Leaveonestudyout.RData" ))  # Load the output of the models to avoid running them   
    
    kable (results.Leave1studyout) %>%   kable_styling ( "striped" ,  position=  "left" )  %>%   scroll_box ( width=  "100%" ,  height=  "500px" )  # Table of the results from all models     
 
 
 
 
 
Estimate
 
 
SE
 
 
zval
 
 
pval
 
 
ci.lb
 
 
ci.ub
 
 
 
 
 
 
0.1891962
 
 
0.0888518
 
 
2.129345
 
 
0.0350140
 
 
0.0134978
 
 
0.3648945
 
 
 
 
0.1896830
 
 
0.0881920
 
 
2.150796
 
 
0.0332471
 
 
0.0152894
 
 
0.3640766
 
 
 
 
0.1935150
 
 
0.0922495
 
 
2.097736
 
 
0.0377789
 
 
0.0110861
 
 
0.3759439
 
 
 
 
0.1893077
 
 
0.0890196
 
 
2.126585
 
 
0.0352472
 
 
0.0132776
 
 
0.3653377
 
 
 
 
0.1901109
 
 
0.0868494
 
 
2.188972
 
 
0.0303053
 
 
0.0183609
 
 
0.3618609
 
 
 
 
0.1898975
 
 
0.0887416
 
 
2.139893
 
 
0.0341484
 
 
0.0144056
 
 
0.3653894
 
 
 
 
0.1901953
 
 
0.0921256
 
 
2.064522
 
 
0.0408684
 
 
0.0080114
 
 
0.3723792
 
 
 
 
0.1897198
 
 
0.0879738
 
 
2.156548
 
 
0.0327865
 
 
0.0157576
 
 
0.3636819
 
 
 
 
0.1917912
 
 
0.0921903
 
 
2.080384
 
 
0.0393950
 
 
0.0094549
 
 
0.3741275
 
 
 
 
0.1895508
 
 
0.0891313
 
 
2.126646
 
 
0.0352420
 
 
0.0132997
 
 
0.3658019
 
 
 
 
0.1898976
 
 
0.0885795
 
 
2.143809
 
 
0.0338270
 
 
0.0147262
 
 
0.3650690
 
 
 
 
0.1884328
 
 
0.0876465
 
 
2.149918
 
 
0.0333308
 
 
0.0151065
 
 
0.3617591
 
 
 
 
0.1899570
 
 
0.0841901
 
 
2.256288
 
 
0.0256482
 
 
0.0234661
 
 
0.3564480
 
 
 
 
0.1856386
 
 
0.0897414
 
 
2.068596
 
 
0.0404643
 
 
0.0081812
 
 
0.3630960
 
 
 
 
0.1897209
 
 
0.0881998
 
 
2.151035
 
 
0.0332278
 
 
0.0153118
 
 
0.3641299
 
 
 
 
0.1895477
 
 
0.0874451
 
 
2.167619
 
 
0.0319157
 
 
0.0166310
 
 
0.3624644
 
 
 
 
0.1896174
 
 
0.0883108
 
 
2.147161
 
 
0.0335540
 
 
0.0149775
 
 
0.3642573
 
 
 
 
0.1895146
 
 
0.0885351
 
 
2.140560
 
 
0.0340804
 
 
0.0144426
 
 
0.3645866
 
 
 
 
0.1895751
 
 
0.0878098
 
 
2.158929
 
 
0.0326105
 
 
0.0159259
 
 
0.3632243
 
 
 
 
0.1917748
 
 
0.0855142
 
 
2.242608
 
 
0.0265285
 
 
0.0226763
 
 
0.3608732
 
 
 
 
0.1880524
 
 
0.0919729
 
 
2.044649
 
 
0.0428044
 
 
0.0061822
 
 
0.3699225
 
 
 
 
0.1891769
 
 
0.0872782
 
 
2.167515
 
 
0.0319496
 
 
0.0165674
 
 
0.3617863
 
 
 
 
0.1908020
 
 
0.0914758
 
 
2.085819
 
 
0.0388634
 
 
0.0099030
 
 
0.3717009
 
 
 
 
0.1894118
 
 
0.0888204
 
 
2.132526
 
 
0.0347470
 
 
0.0137756
 
 
0.3650481
 
 
 
 
0.1994114
 
 
0.0907991
 
 
2.196184
 
 
0.0297610
 
 
0.0198625
 
 
0.3789603
 
 
 
 
0.1899018
 
 
0.0876054
 
 
2.167696
 
 
0.0319098
 
 
0.0166682
 
 
0.3631354
 
 
 
 
0.1888038
 
 
0.0894890
 
 
2.109800
 
 
0.0367075
 
 
0.0118339
 
 
0.3657737
 
 
 
 
0.1925240
 
 
0.0852852
 
 
2.257412
 
 
0.0255879
 
 
0.0238560
 
 
0.3611919
 
 
 
 
0.1899587
 
 
0.0877059
 
 
2.165860
 
 
0.0320528
 
 
0.0165263
 
 
0.3633911
 
 
 
 
0.1897574
 
 
0.0880179
 
 
2.155894
 
 
0.0328516
 
 
0.0156966
 
 
0.3638182
 
 
 
 
0.1900257
 
 
0.0874075
 
 
2.174021
 
 
0.0314341
 
 
0.0171721
 
 
0.3628792
 
 
 
 
0.1900081
 
 
0.0882910
 
 
2.152067
 
 
0.0331578
 
 
0.0154073
 
 
0.3646090
 
 
 
 
0.1900366
 
 
0.0883163
 
 
2.151771
 
 
0.0331816
 
 
0.0153856
 
 
0.3646875
 
 
 
 
0.1870585
 
 
0.0908157
 
 
2.059760
 
 
0.0413146
 
 
0.0074768
 
 
0.3666403
 
 
 
 
0.1913818
 
 
0.0880400
 
 
2.173804
 
 
0.0314381
 
 
0.0172886
 
 
0.3654749
 
 
 
 
0.1920468
 
 
0.0860894
 
 
2.230782
 
 
0.0273707
 
 
0.0217652
 
 
0.3623284
 
 
 
 
0.1927610
 
 
0.0853660
 
 
2.258053
 
 
0.0255233
 
 
0.0239556
 
 
0.3615664
 
 
 
 
0.1892257
 
 
0.0866435
 
 
2.183956
 
 
0.0306674
 
 
0.0178941
 
 
0.3605573
 
 
 
 
0.1893363
 
 
0.0856187
 
 
2.211390
 
 
0.0286787
 
 
0.0200201
 
 
0.3586525
 
 
 
 
0.1872263
 
 
0.0872593
 
 
2.145631
 
 
0.0336784
 
 
0.0146657
 
 
0.3597869
 
 
 
 
0.1863281
 
 
0.0921198
 
 
2.022672
 
 
0.0450530
 
 
0.0041676
 
 
0.3684886
 
 
 
 
0.1902387
 
 
0.0875720
 
 
2.172370
 
 
0.0315483
 
 
0.0170712
 
 
0.3634063
 
 
 
 
0.1905243
 
 
0.0871710
 
 
2.185640
 
 
0.0305412
 
 
0.0181497
 
 
0.3628988
 
 
 
 
0.1890701
 
 
0.0906983
 
 
2.084605
 
 
0.0389754
 
 
0.0097087
 
 
0.3684314
 
 
 
 
0.1892476
 
 
0.0887162
 
 
2.133180
 
 
0.0346923
 
 
0.0138174
 
 
0.3646778
 
 
 
 
0.1934850
 
 
0.0884912
 
 
2.186488
 
 
0.0304904
 
 
0.0184882
 
 
0.3684818
 
 
 
 
0.1968182
 
 
0.1151566
 
 
1.709135
 
 
0.0897237
 
 
-0.0309261
 
 
0.4245626
 
 
 
 
0.1890500
 
 
0.0863240
 
 
2.190005
 
 
0.0302286
 
 
0.0183391
 
 
0.3597610
 
 
 
 
0.1885415
 
 
0.0894374
 
 
2.108085
 
 
0.0368582
 
 
0.0116737
 
 
0.3654093
 
 
 
 
0.1892877
 
 
0.0855761
 
 
2.211923
 
 
0.0286410
 
 
0.0200558
 
 
0.3585197
 
 
 
 
0.1896529
 
 
0.0889030
 
 
2.133256
 
 
0.0346860
 
 
0.0138533
 
 
0.3654525
 
 
 
 
0.1894624
 
 
0.0882922
 
 
2.145858
 
 
0.0336599
 
 
0.0148593
 
 
0.3640655
 
 
 
 
0.1917401
 
 
0.0935210
 
 
2.050237
 
 
0.0422624
 
 
0.0067967
 
 
0.3766835
 
 
 
 
0.1894905
 
 
0.0885835
 
 
2.139117
 
 
0.0342124
 
 
0.0143112
 
 
0.3646698
 
 
 
 
0.1894695
 
 
0.0876072
 
 
2.162716
 
 
0.0323118
 
 
0.0162209
 
 
0.3627180
 
 
 
 
0.1897645
 
 
0.0880294
 
 
2.155695
 
 
0.0328546
 
 
0.0156924
 
 
0.3638366
 
 
 
 
0.1898430
 
 
0.0861527
 
 
2.203564
 
 
0.0292376
 
 
0.0194708
 
 
0.3602151
 
 
 
 
0.1935889
 
 
0.0897753
 
 
2.156370
 
 
0.0328136
 
 
0.0160527
 
 
0.3711251
 
 
 
 
0.1901114
 
 
0.0835333
 
 
2.275874
 
 
0.0244164
 
 
0.0249191
 
 
0.3553036
 
 
 
 
0.1921936
 
 
0.0928420
 
 
2.070115
 
 
0.0403335
 
 
0.0085929
 
 
0.3757942
 
 
 
 
0.1897451
 
 
0.0880317
 
 
2.155418
 
 
0.0328895
 
 
0.0156571
 
 
0.3638332
 
 
 
 
0.1892785
 
 
0.0882959
 
 
2.143685
 
 
0.0338372
 
 
0.0146681
 
 
0.3638889
 
 
 
 
0.1902220
 
 
0.0870065
 
 
2.186297
 
 
0.0304921
 
 
0.0181727
 
 
0.3622713
 
 
 
 
0.1886690
 
 
0.0866849
 
 
2.176493
 
 
0.0312451
 
 
0.0172444
 
 
0.3600937
 
 
 
 
0.1905635
 
 
0.0856221
 
 
2.225634
 
 
0.0276974
 
 
0.0212293
 
 
0.3598978
 
 
 
 
0.1895705
 
 
0.0884484
 
 
2.143288
 
 
0.0338565
 
 
0.0146698
 
 
0.3644712
 
 
 
 
0.1914289
 
 
0.0855009
 
 
2.238912
 
 
0.0267741
 
 
0.0223569
 
 
0.3605010
 
 
 
 
0.1898751
 
 
0.0880624
 
 
2.156143
 
 
0.0328188
 
 
0.0157378
 
 
0.3640125
 
 
 
 
0.1898301
 
 
0.0880984
 
 
2.154751
 
 
0.0329299
 
 
0.0156216
 
 
0.3640386
 
 
 
 
0.1899318
 
 
0.0880288
 
 
2.157608
 
 
0.0327023
 
 
0.0158608
 
 
0.3640027
 
 
 
 
0.1905511
 
 
0.0902907
 
 
2.110419
 
 
0.0366532
 
 
0.0119959
 
 
0.3691064
 
 
 
 
0.1902141
 
 
0.0858846
 
 
2.214764
 
 
0.0284283
 
 
0.0203833
 
 
0.3600450
 
 
 
 
0.1891178
 
 
0.0888197
 
 
2.129233
 
 
0.0350501
 
 
0.0134598
 
 
0.3647758
 
 
 
 
0.1874340
 
 
0.0824692
 
 
2.272774
 
 
0.0246078
 
 
0.0243460
 
 
0.3505219
 
 
 
 
0.1892929
 
 
0.0877294
 
 
2.157690
 
 
0.0326958
 
 
0.0158140
 
 
0.3627719
 
 
 
 
0.1898535
 
 
0.0866550
 
 
2.190912
 
 
0.0301615
 
 
0.0184879
 
 
0.3612191
 
 
 
 
0.1897554
 
 
0.0881877
 
 
2.151723
 
 
0.0331725
 
 
0.0153704
 
 
0.3641405
 
 
 
 
0.1899841
 
 
0.0867373
 
 
2.190340
 
 
0.0301913
 
 
0.0184671
 
 
0.3615012
 
 
 
 
0.1896501
 
 
0.0879459
 
 
2.156441
 
 
0.0327951
 
 
0.0157432
 
 
0.3635570
 
 
 
 
0.1897206
 
 
0.0880195
 
 
2.155438
 
 
0.0328879
 
 
0.0156567
 
 
0.3637846
 
 
 
 
0.1892194
 
 
0.0863928
 
 
2.190221
 
 
0.0302001
 
 
0.0183836
 
 
0.3600553
 
 
 
 
0.1893224
 
 
0.0875846
 
 
2.161595
 
 
0.0323871
 
 
0.0161299
 
 
0.3625149
 
 
 
 
0.1898400
 
 
0.0880539
 
 
2.155954
 
 
0.0328339
 
 
0.0157196
 
 
0.3639605
 
 
 
 
0.1794748
 
 
0.0857652
 
 
2.092630
 
 
0.0382404
 
 
0.0098689
 
 
0.3490807
 
 
 
 
0.1900616
 
 
0.0846729
 
 
2.244658
 
 
0.0264050
 
 
0.0226159
 
 
0.3575074
 
 
 
 
0.1903354
 
 
0.0871325
 
 
2.184437
 
 
0.0306313
 
 
0.0180369
 
 
0.3626339
 
 
 
 
0.1898280
 
 
0.0886914
 
 
2.140320
 
 
0.0341132
 
 
0.0144354
 
 
0.3652207
 
 
 
 
0.1902226
 
 
0.0865903
 
 
2.196813
 
 
0.0297275
 
 
0.0189851
 
 
0.3614602
 
 
 
 
0.1903063
 
 
0.0893678
 
 
2.129474
 
 
0.0350164
 
 
0.0135761
 
 
0.3670365
 
 
 
 
0.1905583
 
 
0.0906505
 
 
2.102120
 
 
0.0373729
 
 
0.0113031
 
 
0.3698135
 
 
 
 
0.1896650
 
 
0.0877076
 
 
2.162469
 
 
0.0323442
 
 
0.0162064
 
 
0.3631237
 
 
 
 
0.1900185
 
 
0.0885966
 
 
2.144761
 
 
0.0337362
 
 
0.0148248
 
 
0.3652122
 
 
 
 
0.1895990
 
 
0.0886836
 
 
2.137927
 
 
0.0342976
 
 
0.0142334
 
 
0.3649647
 
 
 
 
0.1892893
 
 
0.0880145
 
 
2.150660
 
 
0.0332710
 
 
0.0152352
 
 
0.3633433
 
 
 
 
0.1892568
 
 
0.0901717
 
 
2.098850
 
 
0.0376788
 
 
0.0109368
 
 
0.3675768
 
 
 
 
0.1899536
 
 
0.0894095
 
 
2.124534
 
 
0.0354346
 
 
0.0131408
 
 
0.3667664
 
 
 
 
0.1896331
 
 
0.0882847
 
 
2.147972
 
 
0.0334752
 
 
0.0150562
 
 
0.3642099
 
 
 
 
0.1897318
 
 
0.0880843
 
 
2.153979
 
 
0.0329915
 
 
0.0155511
 
 
0.3639125
 
 
 
 
0.1899094
 
 
0.0892838
 
 
2.127031
 
 
0.0352227
 
 
0.0133453
 
 
0.3664736
 
 
 
 
0.1896161
 
 
0.0887412
 
 
2.136731
 
 
0.0344098
 
 
0.0141250
 
 
0.3651072
 
 
 
 
0.1893939
 
 
0.0874822
 
 
2.164942
 
 
0.0321245
 
 
0.0164039
 
 
0.3623840
 
 
 
 
0.1893680
 
 
0.0857105
 
 
2.209391
 
 
0.0288206
 
 
0.0198703
 
 
0.3588657
 
 
 
 
0.1895283
 
 
0.0863523
 
 
2.194826
 
 
0.0298730
 
 
0.0187613
 
 
0.3602952
 
 
 
 
0.1896672
 
 
0.0883806
 
 
2.146028
 
 
0.0336460
 
 
0.0148892
 
 
0.3644451
 
 
 
 
0.1897249
 
 
0.0882227
 
 
2.150522
 
 
0.0332691
 
 
0.0152705
 
 
0.3641792
 
 
 
 
0.1906219
 
 
0.0853974
 
 
2.232174
 
 
0.0272391
 
 
0.0217433
 
 
0.3595005
 
 
 
 
0.1896140
 
 
0.0884483
 
 
2.143784
 
 
0.0338290
 
 
0.0147022
 
 
0.3645258
 
 
 
 
0.1912048
 
 
0.0844211
 
 
2.264893
 
 
0.0251004
 
 
0.0242569
 
 
0.3581527
 
 
 
 
0.1895172
 
 
0.0886370
 
 
2.138126
 
 
0.0342943
 
 
0.0142320
 
 
0.3648023
 
 
 
 
0.1899587
 
 
0.0874207
 
 
2.172925
 
 
0.0315183
 
 
0.0170789
 
 
0.3628386
 
 
 
 
0.1906898
 
 
0.0884795
 
 
2.155188
 
 
0.0329079
 
 
0.0157163
 
 
0.3656633
 
 
 
 
0.1885328
 
 
0.0871918
 
 
2.162278
 
 
0.0323462
 
 
0.0161058
 
 
0.3609598
 
 
 
 
0.1894506
 
 
0.0882631
 
 
2.146431
 
 
0.0336133
 
 
0.0149050
 
 
0.3639963
 
 
 
 
0.1900917
 
 
0.0884407
 
 
2.149370
 
 
0.0333751
 
 
0.0151949
 
 
0.3649886
 
 
 
 
0.1874470
 
 
0.0850272
 
 
2.204553
 
 
0.0291665
 
 
0.0193006
 
 
0.3555934
 
 
 
 
0.1911127
 
 
0.0882898
 
 
2.164607
 
 
0.0321635
 
 
0.0165143
 
 
0.3657111
 
 
 
 
0.1895040
 
 
0.0884554
 
 
2.142368
 
 
0.0339319
 
 
0.0145895
 
 
0.3644185
 
 
 
 
0.1907109
 
 
0.0854679
 
 
2.231373
 
 
0.0273056
 
 
0.0216816
 
 
0.3597402
 
 
 
 
0.1893340
 
 
0.0917568
 
 
2.063432
 
 
0.0409594
 
 
0.0078912
 
 
0.3707768
 
 
 
 
0.1898826
 
 
0.0877062
 
 
2.164986
 
 
0.0321211
 
 
0.0164497
 
 
0.3633156
 
 
 
 
0.1897615
 
 
0.0877221
 
 
2.163213
 
 
0.0322599
 
 
0.0162971
 
 
0.3632258
 
 
 
 
0.1896416
 
 
0.0884668
 
 
2.143646
 
 
0.0338272
 
 
0.0147045
 
 
0.3645787
 
 
 
 
0.1897274
 
 
0.0882802
 
 
2.149151
 
 
0.0333798
 
 
0.0151594
 
 
0.3642955
 
 
 
 
0.1874839
 
 
0.0871073
 
 
2.152334
 
 
0.0331364
 
 
0.0152240
 
 
0.3597438
 
 
 
 
0.1896744
 
 
0.0876632
 
 
2.163673
 
 
0.0322367
 
 
0.0163151
 
 
0.3630336
 
 
 
 
0.1894249
 
 
0.0881903
 
 
2.147911
 
 
0.0334932
 
 
0.0150232
 
 
0.3638266
 
 
 
 
0.1896158
 
 
0.0879146
 
 
2.156818
 
 
0.0327780
 
 
0.0157594
 
 
0.3634722
 
 
 
 
0.1886234
 
 
0.0835740
 
 
2.256963
 
 
0.0256048
 
 
0.0233507
 
 
0.3538960
 
 
 
 
0.1892933
 
 
0.0897818
 
 
2.108370
 
 
0.0368331
 
 
0.0117443
 
 
0.3668423
 
 
 
 
0.1885648
 
 
0.0831488
 
 
2.267800
 
 
0.0249177
 
 
0.0241331
 
 
0.3529965
 
 
 
 
0.1893586
 
 
0.0893978
 
 
2.118157
 
 
0.0359808
 
 
0.0125690
 
 
0.3661482
 
 
 
 
0.1893304
 
 
0.0890414
 
 
2.126318
 
 
0.0352831
 
 
0.0132455
 
 
0.3654153
 
 
 
 
0.1894142
 
 
0.0889088
 
 
2.130432
 
 
0.0349358
 
 
0.0135916
 
 
0.3652368
 
 
 
 
0.1890185
 
 
0.0880312
 
 
2.147177
 
 
0.0335527
 
 
0.0149315
 
 
0.3631055
 
 
 
 
0.1852496
 
 
0.0794997
 
 
2.330194
 
 
0.0212675
 
 
0.0280342
 
 
0.3424651
 
 
 
 
0.1886422
 
 
0.0860301
 
 
2.192747
 
 
0.0300134
 
 
0.0185236
 
 
0.3587608
 
 
 
 
0.1896607
 
 
0.0875926
 
 
2.165258
 
 
0.0321126
 
 
0.0164409
 
 
0.3628804
 
 
 
 
0.1890887
 
 
0.0881816
 
 
2.144309
 
 
0.0337731
 
 
0.0147156
 
 
0.3634618
 
 
 
 
0.1900426
 
 
0.0910718
 
 
2.086734
 
 
0.0387792
 
 
0.0099426
 
 
0.3701427
 
 
 
 
0.1897864
 
 
0.0879173
 
 
2.158691
 
 
0.0326164
 
 
0.0159359
 
 
0.3636369
 
 
 
 
0.1876850
 
 
0.0879293
 
 
2.134500
 
 
0.0345953
 
 
0.0137995
 
 
0.3615704
 
 
 
 
0.1898034
 
 
0.0880563
 
 
2.155477
 
 
0.0328848
 
 
0.0156666
 
 
0.3639401
 
 
 
 
0.1897518
 
 
0.0880519
 
 
2.154999
 
 
0.0329100
 
 
0.0156352
 
 
0.3638685
 
 
 
 
0.1890736
 
 
0.0848397
 
 
2.228598
 
 
0.0274822
 
 
0.0212979
 
 
0.3568494
 
 
 
 
0.1902266
 
 
0.0869692
 
 
2.187286
 
 
0.0304182
 
 
0.0182509
 
 
0.3622023
 
 
 
 
0.1901475
 
 
0.0864046
 
 
2.200663
 
 
0.0294473
 
 
0.0192771
 
 
0.3610179
 
 
 
 
0.1912187
 
 
0.0876875
 
 
2.180685
 
 
0.0309927
 
 
0.0177520
 
 
0.3646855
 
 
 
 
0.1897455
 
 
0.0867766
 
 
2.186599
 
 
0.0304695
 
 
0.0181509
 
 
0.3613402
 
 
 
 
0.1899367
 
 
0.0871532
 
 
2.179343
 
 
0.0310156
 
 
0.0175972
 
 
0.3622762
 
 
 
 
0.1895812
 
 
0.0876759
 
 
2.162295
 
 
0.0323448
 
 
0.0161968
 
 
0.3629656
 
 
 
 
 
       ggplot (results.Leave1studyout) +  stat_dots ( aes ( x= Estimate),  alpha=  0.8 ,  dotsize=  1.5 ,  shape=  16 ,  show.legend= F,  col=  "darkcyan" )  # Distribution of overall mean after removal of one study at a time     
   
      results.Leave1studyout  %>%   
                              summarise ( estimate=   mean (Estimate),  # calculate the mean coefficients across the models generated  
                                        se=  mean (SE),  
                                        zval=  mean (zval),  
                                        pval=  mean (pval),  
                                        ci.lb=  mean (ci.lb),  
                                        ci.ub=  mean (ci.ub))  %>%   
                                                              kable () %>%   kable_styling ( "striped" ,  position=  "left" )    
 
 
 
 
estimate
 
 
se
 
 
zval
 
 
pval
 
 
ci.lb
 
 
ci.ub
 
 
 
 
 
 
0.1897728
 
 
0.0880607
 
 
2.156954
 
 
0.0331004
 
 
0.0156305
 
 
0.363915
 
 
 
 
 
 
 Leave one species out 
      d $ species_ID <-  as.factor (d $ species_ID) 
   d <-  as.data.frame (d)  # Only work with a dataframe  
   VCV_matrix <-  list ()  # will need new VCV matrices because the sample size will be iteratively reduced  
   Leave1speciesout <-  list ()  # create a list that will host the results of each model   
    for (i  in   1  :  length ( levels (d $ species_ID))){  # N models = N species   
     VCV_matrix[[i]] <-  make_VCV_matrix (d[d $ species_ID  !=   levels (d $ species_ID)[i], ],  V=  "Var_dARR" ,  cluster=  "shared_trt_ID" ,  obs=  "es_ID" )  # Create a new VCV matrix for each new model  
     Leave1speciesout[[i]]  <-   rma.mv ( yi =  dARR,  V =  VCV_matrix[[i]],  # Same model structure as all the models we fitted  
                                    method=  "REML" , 
                                    test=  "t" , 
                                    dfs=  "contain" , 
                                    random =   list ( ~  1  | species_ID, 
                                                  ~  1  | phylogeny,  
                                                  ~  1  | es_ID), 
                                    R=   list ( phylogeny =  phylo_matrix),  
                                    data =  d[d $ species_ID  !=   levels (d $ species_ID)[i], ], 
                                    sparse=  TRUE )  # Generate a new model for each new data (iterative removal of one species at a time)  
   } 
     
    
    
    # The output is a list so we need to summarise the coefficients of all the models performed  
    
   results.Leave1speciesout <-  as.data.frame ( cbind ( 
                                               sapply (Leave1speciesout,  function (x)  summary (x) $ beta),  # extract the beta coefficient from all models  
                                               sapply (Leave1speciesout,  function (x)  summary (x) $ se),  # extract the standard error from all models  
                                               sapply (Leave1speciesout,  function (x)  summary (x) $ zval),   # extract the z value from all models  
                                               sapply (Leave1speciesout,  function (x)  summary (x) $ pval),  # extract the p value from all models  
                                               sapply (Leave1speciesout,  function (x)  summary (x) $ ci.lb),  # extract the lower confidence interval for all models  
                                               sapply (Leave1speciesout,  function (x)  summary (x) $ ci.ub)))  # extract the upper confidence interval for all models  
    
    colnames (results.Leave1speciesout) =  c ( "Estimate" ,  "SE" ,  "zval" ,  "pval" ,  "ci.lb" ,  "ci.ub" )  # change column names   
    kable (results.Leave1speciesout) %>%   kable_styling ( "striped" ,  position=  "left" )  %>%   scroll_box ( width=  "100%" ,  height=  "500px" )  # Table of the results from all models  
    
    ggplot (results.Leave1speciesout) +  stat_dots ( aes ( x= Estimate),  alpha=  0.8 ,  dotsize=  1.5 ,  shape=  16 ,  show.legend= F,  col=  "darkcyan" )  # Distribution of overall mean after removal of one species at a time  
    
   results.Leave1speciesout  %>%   
                              summarise ( estimate=   mean (Estimate),  # calculate the mean coefficients across the models generated  
                                        se=  mean (SE),  
                                        zval=  mean (zval),  
                                        pval=  mean (pval),  
                                        ci.lb=  mean (ci.lb),  
                                        ci.ub=  mean (ci.ub))  %>%   
                                                              kable () %>%   kable_styling ( "striped" ,  position=  "left" )    
       load ( here ( "Rdata" ,  "Leaveonespeciesout.RData" ))  # Load the output of the models to avoid running them   
    
    kable (results.Leave1speciesout) %>%   kable_styling ( "striped" ,  position=  "left" )  %>%   scroll_box ( width=  "100%" ,  height=  "500px" )  # Table of the results from all models     
 
 
 
 
 
Estimate
 
 
SE
 
 
zval
 
 
pval
 
 
ci.lb
 
 
ci.ub
 
 
 
 
 
 
0.1924767
 
 
0.0834555
 
 
2.306339
 
 
0.0226034
 
 
0.0274384
 
 
0.3575151
 
 
 
 
0.1897048
 
 
0.0882498
 
 
2.149635
 
 
0.0333537
 
 
0.0151855
 
 
0.3642240
 
 
 
 
0.1908438
 
 
0.0901151
 
 
2.117779
 
 
0.0360134
 
 
0.0126357
 
 
0.3690520
 
 
 
 
0.1902537
 
 
0.0888599
 
 
2.141053
 
 
0.0340530
 
 
0.0145279
 
 
0.3659795
 
 
 
 
0.1898509
 
 
0.0882153
 
 
2.152133
 
 
0.0331525
 
 
0.0153999
 
 
0.3643019
 
 
 
 
0.1898976
 
 
0.0885795
 
 
2.143809
 
 
0.0338270
 
 
0.0147262
 
 
0.3650690
 
 
 
 
0.1884328
 
 
0.0876465
 
 
2.149918
 
 
0.0333308
 
 
0.0151065
 
 
0.3617591
 
 
 
 
0.1867680
 
 
0.0898680
 
 
2.078248
 
 
0.0395663
 
 
0.0090486
 
 
0.3644874
 
 
 
 
0.1896174
 
 
0.0883108
 
 
2.147161
 
 
0.0335540
 
 
0.0149775
 
 
0.3642573
 
 
 
 
0.1895751
 
 
0.0878098
 
 
2.158929
 
 
0.0326105
 
 
0.0159259
 
 
0.3632243
 
 
 
 
0.1867280
 
 
0.0941494
 
 
1.983316
 
 
0.0493463
 
 
0.0005419
 
 
0.3729141
 
 
 
 
0.1893618
 
 
0.0877713
 
 
2.157446
 
 
0.0327281
 
 
0.0157887
 
 
0.3629349
 
 
 
 
0.1894765
 
 
0.0872333
 
 
2.172067
 
 
0.0315843
 
 
0.0169675
 
 
0.3619856
 
 
 
 
0.1895850
 
 
0.0876949
 
 
2.161870
 
 
0.0323783
 
 
0.0161630
 
 
0.3630071
 
 
 
 
0.1908020
 
 
0.0914758
 
 
2.085819
 
 
0.0388634
 
 
0.0099030
 
 
0.3717009
 
 
 
 
0.1904728
 
 
0.0874760
 
 
2.177428
 
 
0.0311738
 
 
0.0174836
 
 
0.3634620
 
 
 
 
0.1905516
 
 
0.0875934
 
 
2.175410
 
 
0.0313278
 
 
0.0173303
 
 
0.3637729
 
 
 
 
0.1897574
 
 
0.0880179
 
 
2.155894
 
 
0.0328516
 
 
0.0156966
 
 
0.3638182
 
 
 
 
0.1900257
 
 
0.0874075
 
 
2.174021
 
 
0.0314341
 
 
0.0171721
 
 
0.3628792
 
 
 
 
0.1900081
 
 
0.0882910
 
 
2.152067
 
 
0.0331578
 
 
0.0154073
 
 
0.3646090
 
 
 
 
0.1900366
 
 
0.0883163
 
 
2.151771
 
 
0.0331816
 
 
0.0153856
 
 
0.3646875
 
 
 
 
0.1920676
 
 
0.0857235
 
 
2.240549
 
 
0.0266771
 
 
0.0225442
 
 
0.3615910
 
 
 
 
0.1893830
 
 
0.0885411
 
 
2.138929
 
 
0.0342279
 
 
0.0142877
 
 
0.3644784
 
 
 
 
0.1899570
 
 
0.0841901
 
 
2.256288
 
 
0.0256482
 
 
0.0234661
 
 
0.3564480
 
 
 
 
0.1903609
 
 
0.0876692
 
 
2.171354
 
 
0.0316393
 
 
0.0169897
 
 
0.3637320
 
 
 
 
0.1901264
 
 
0.0875716
 
 
2.171097
 
 
0.0316591
 
 
0.0169483
 
 
0.3633045
 
 
 
 
0.1900914
 
 
0.0878720
 
 
2.163276
 
 
0.0322678
 
 
0.0163192
 
 
0.3638636
 
 
 
 
0.1900200
 
 
0.0880308
 
 
2.158563
 
 
0.0326395
 
 
0.0159337
 
 
0.3641063
 
 
 
 
0.1893363
 
 
0.0856187
 
 
2.211390
 
 
0.0286787
 
 
0.0200201
 
 
0.3586525
 
 
 
 
0.1872263
 
 
0.0872593
 
 
2.145631
 
 
0.0336784
 
 
0.0146657
 
 
0.3597869
 
 
 
 
0.1846879
 
 
0.0928533
 
 
1.989030
 
 
0.0487044
 
 
0.0010650
 
 
0.3683109
 
 
 
 
0.1890701
 
 
0.0906983
 
 
2.084605
 
 
0.0389754
 
 
0.0097087
 
 
0.3684314
 
 
 
 
0.1934850
 
 
0.0884912
 
 
2.186488
 
 
0.0304904
 
 
0.0184882
 
 
0.3684818
 
 
 
 
0.1924329
 
 
0.0985551
 
 
1.952541
 
 
0.0529279
 
 
-0.0024658
 
 
0.3873316
 
 
 
 
0.1931318
 
 
0.0998147
 
 
1.934903
 
 
0.0550780
 
 
-0.0042578
 
 
0.3905215
 
 
 
 
0.1890500
 
 
0.0863240
 
 
2.190005
 
 
0.0302286
 
 
0.0183391
 
 
0.3597610
 
 
 
 
0.1885415
 
 
0.0894374
 
 
2.108085
 
 
0.0368582
 
 
0.0116737
 
 
0.3654093
 
 
 
 
0.1892877
 
 
0.0855761
 
 
2.211923
 
 
0.0286410
 
 
0.0200558
 
 
0.3585197
 
 
 
 
0.1894624
 
 
0.0882922
 
 
2.145858
 
 
0.0336599
 
 
0.0148593
 
 
0.3640655
 
 
 
 
0.1917401
 
 
0.0935210
 
 
2.050237
 
 
0.0422624
 
 
0.0067967
 
 
0.3766835
 
 
 
 
0.1895160
 
 
0.0885735
 
 
2.139646
 
 
0.0341688
 
 
0.0143565
 
 
0.3646755
 
 
 
 
0.1893531
 
 
0.0884143
 
 
2.141658
 
 
0.0340032
 
 
0.0145085
 
 
0.3641977
 
 
 
 
0.1898430
 
 
0.0861527
 
 
2.203564
 
 
0.0292376
 
 
0.0194708
 
 
0.3602151
 
 
 
 
0.1935889
 
 
0.0897753
 
 
2.156370
 
 
0.0328136
 
 
0.0160527
 
 
0.3711251
 
 
 
 
0.1901114
 
 
0.0835333
 
 
2.275874
 
 
0.0244164
 
 
0.0249191
 
 
0.3553036
 
 
 
 
0.1903242
 
 
0.0873715
 
 
2.178334
 
 
0.0311048
 
 
0.0175419
 
 
0.3631066
 
 
 
 
0.1893566
 
 
0.0887014
 
 
2.134765
 
 
0.0345733
 
 
0.0139442
 
 
0.3647690
 
 
 
 
0.1899446
 
 
0.0888877
 
 
2.136906
 
 
0.0343953
 
 
0.0141638
 
 
0.3657254
 
 
 
 
0.1916686
 
 
0.0859927
 
 
2.228893
 
 
0.0274620
 
 
0.0216128
 
 
0.3617245
 
 
 
 
0.1895825
 
 
0.0875805
 
 
2.164667
 
 
0.0321588
 
 
0.0163868
 
 
0.3627782
 
 
 
 
0.1921936
 
 
0.0928420
 
 
2.070115
 
 
0.0403335
 
 
0.0085929
 
 
0.3757942
 
 
 
 
0.1897451
 
 
0.0880317
 
 
2.155418
 
 
0.0328895
 
 
0.0156571
 
 
0.3638332
 
 
 
 
0.1881906
 
 
0.0825785
 
 
2.278930
 
 
0.0242290
 
 
0.0248866
 
 
0.3514946
 
 
 
 
0.1886690
 
 
0.0866849
 
 
2.176493
 
 
0.0312451
 
 
0.0172444
 
 
0.3600937
 
 
 
 
0.1892308
 
 
0.0880637
 
 
2.148794
 
 
0.0334217
 
 
0.0150794
 
 
0.3633821
 
 
 
 
0.1897710
 
 
0.0878699
 
 
2.159681
 
 
0.0325510
 
 
0.0160029
 
 
0.3635390
 
 
 
 
0.1894221
 
 
0.0886188
 
 
2.137493
 
 
0.0343467
 
 
0.0141730
 
 
0.3646713
 
 
 
 
0.1898464
 
 
0.0872887
 
 
2.174926
 
 
0.0313648
 
 
0.0172278
 
 
0.3624651
 
 
 
 
0.1905001
 
 
0.0863099
 
 
2.207165
 
 
0.0289793
 
 
0.0198171
 
 
0.3611831
 
 
 
 
0.1905511
 
 
0.0902907
 
 
2.110419
 
 
0.0366532
 
 
0.0119959
 
 
0.3691064
 
 
 
 
0.1899535
 
 
0.0864437
 
 
2.197423
 
 
0.0296829
 
 
0.0190057
 
 
0.3609012
 
 
 
 
0.1891397
 
 
0.0888859
 
 
2.127893
 
 
0.0351498
 
 
0.0133624
 
 
0.3649170
 
 
 
 
0.1897736
 
 
0.0880071
 
 
2.156343
 
 
0.0328158
 
 
0.0157341
 
 
0.3638131
 
 
 
 
0.1874340
 
 
0.0824692
 
 
2.272774
 
 
0.0246078
 
 
0.0243460
 
 
0.3505219
 
 
 
 
0.1908732
 
 
0.0858545
 
 
2.223216
 
 
0.0278516
 
 
0.0210906
 
 
0.3606557
 
 
 
 
0.1898535
 
 
0.0866550
 
 
2.190912
 
 
0.0301615
 
 
0.0184879
 
 
0.3612191
 
 
 
 
0.1897371
 
 
0.0881657
 
 
2.152052
 
 
0.0331590
 
 
0.0153842
 
 
0.3640900
 
 
 
 
0.1899333
 
 
0.0869840
 
 
2.183544
 
 
0.0307110
 
 
0.0179173
 
 
0.3619494
 
 
 
 
0.1897206
 
 
0.0880195
 
 
2.155438
 
 
0.0328879
 
 
0.0156567
 
 
0.3637846
 
 
 
 
0.1892903
 
 
0.0871312
 
 
2.172476
 
 
0.0315529
 
 
0.0169832
 
 
0.3615975
 
 
 
 
0.1794748
 
 
0.0857652
 
 
2.092630
 
 
0.0382404
 
 
0.0098689
 
 
0.3490807
 
 
 
 
0.1900616
 
 
0.0846729
 
 
2.244658
 
 
0.0264050
 
 
0.0226159
 
 
0.3575074
 
 
 
 
0.1898280
 
 
0.0886914
 
 
2.140320
 
 
0.0341132
 
 
0.0144354
 
 
0.3652207
 
 
 
 
0.1902226
 
 
0.0865903
 
 
2.196813
 
 
0.0297275
 
 
0.0189851
 
 
0.3614602
 
 
 
 
0.1903063
 
 
0.0893678
 
 
2.129474
 
 
0.0350164
 
 
0.0135761
 
 
0.3670365
 
 
 
 
0.1904055
 
 
0.0862630
 
 
2.207267
 
 
0.0289720
 
 
0.0198151
 
 
0.3609959
 
 
 
 
0.1911552
 
 
0.0925271
 
 
2.065937
 
 
0.0407325
 
 
0.0081772
 
 
0.3741332
 
 
 
 
0.1904730
 
 
0.0844938
 
 
2.254283
 
 
0.0257773
 
 
0.0233813
 
 
0.3575646
 
 
 
 
0.1897109
 
 
0.0881263
 
 
2.152716
 
 
0.0331057
 
 
0.0154358
 
 
0.3639861
 
 
 
 
0.1896835
 
 
0.0882637
 
 
2.149055
 
 
0.0334005
 
 
0.0151367
 
 
0.3642303
 
 
 
 
0.1892072
 
 
0.0908954
 
 
2.081593
 
 
0.0392544
 
 
0.0094561
 
 
0.3689583
 
 
 
 
0.1892893
 
 
0.0880145
 
 
2.150660
 
 
0.0332710
 
 
0.0152352
 
 
0.3633433
 
 
 
 
0.1892568
 
 
0.0901717
 
 
2.098850
 
 
0.0376788
 
 
0.0109368
 
 
0.3675768
 
 
 
 
0.1899536
 
 
0.0894095
 
 
2.124534
 
 
0.0354346
 
 
0.0131408
 
 
0.3667664
 
 
 
 
0.1899094
 
 
0.0892838
 
 
2.127031
 
 
0.0352227
 
 
0.0133453
 
 
0.3664736
 
 
 
 
0.1896161
 
 
0.0887412
 
 
2.136731
 
 
0.0344098
 
 
0.0141250
 
 
0.3651072
 
 
 
 
0.1902362
 
 
0.0867392
 
 
2.193198
 
 
0.0299927
 
 
0.0187042
 
 
0.3617683
 
 
 
 
0.1860602
 
 
0.0799310
 
 
2.327760
 
 
0.0214005
 
 
0.0279918
 
 
0.3441287
 
 
 
 
0.1893680
 
 
0.0857105
 
 
2.209391
 
 
0.0288206
 
 
0.0198703
 
 
0.3588657
 
 
 
 
0.1895283
 
 
0.0863523
 
 
2.194826
 
 
0.0298730
 
 
0.0187613
 
 
0.3602952
 
 
 
 
0.1896672
 
 
0.0883806
 
 
2.146028
 
 
0.0336460
 
 
0.0148892
 
 
0.3644451
 
 
 
 
0.1906219
 
 
0.0853974
 
 
2.232174
 
 
0.0272391
 
 
0.0217433
 
 
0.3595005
 
 
 
 
0.1896140
 
 
0.0884483
 
 
2.143784
 
 
0.0338290
 
 
0.0147022
 
 
0.3645258
 
 
 
 
0.1895172
 
 
0.0886370
 
 
2.138126
 
 
0.0342943
 
 
0.0142320
 
 
0.3648023
 
 
 
 
0.1899587
 
 
0.0874207
 
 
2.172925
 
 
0.0315183
 
 
0.0170789
 
 
0.3628386
 
 
 
 
0.1906898
 
 
0.0884795
 
 
2.155188
 
 
0.0329079
 
 
0.0157163
 
 
0.3656633
 
 
 
 
0.1885328
 
 
0.0871918
 
 
2.162278
 
 
0.0323462
 
 
0.0161058
 
 
0.3609598
 
 
 
 
0.1912048
 
 
0.0844211
 
 
2.264893
 
 
0.0251004
 
 
0.0242569
 
 
0.3581527
 
 
 
 
0.1894506
 
 
0.0882631
 
 
2.146431
 
 
0.0336133
 
 
0.0149050
 
 
0.3639963
 
 
 
 
0.1900917
 
 
0.0884407
 
 
2.149370
 
 
0.0333751
 
 
0.0151949
 
 
0.3649886
 
 
 
 
0.1874470
 
 
0.0850272
 
 
2.204553
 
 
0.0291665
 
 
0.0193006
 
 
0.3555934
 
 
 
 
0.1911127
 
 
0.0882898
 
 
2.164607
 
 
0.0321635
 
 
0.0165143
 
 
0.3657111
 
 
 
 
0.1901108
 
 
0.0869487
 
 
2.186470
 
 
0.0304917
 
 
0.0181644
 
 
0.3620572
 
 
 
 
0.1901068
 
 
0.0869605
 
 
2.186128
 
 
0.0305173
 
 
0.0181371
 
 
0.3620765
 
 
 
 
0.1901130
 
 
0.0868949
 
 
2.187850
 
 
0.0303888
 
 
0.0182730
 
 
0.3619531
 
 
 
 
0.1874839
 
 
0.0871073
 
 
2.152334
 
 
0.0331364
 
 
0.0152240
 
 
0.3597438
 
 
 
 
0.1896744
 
 
0.0876632
 
 
2.163673
 
 
0.0322367
 
 
0.0163151
 
 
0.3630336
 
 
 
 
0.1894249
 
 
0.0881903
 
 
2.147911
 
 
0.0334932
 
 
0.0150232
 
 
0.3638266
 
 
 
 
0.1893406
 
 
0.0916130
 
 
2.066745
 
 
0.0406551
 
 
0.0081704
 
 
0.3705108
 
 
 
 
0.1896158
 
 
0.0879146
 
 
2.156818
 
 
0.0327780
 
 
0.0157594
 
 
0.3634722
 
 
 
 
0.1886234
 
 
0.0835740
 
 
2.256963
 
 
0.0256048
 
 
0.0233507
 
 
0.3538960
 
 
 
 
0.1892933
 
 
0.0897818
 
 
2.108370
 
 
0.0368331
 
 
0.0117443
 
 
0.3668423
 
 
 
 
0.1893586
 
 
0.0893978
 
 
2.118157
 
 
0.0359808
 
 
0.0125690
 
 
0.3661482
 
 
 
 
0.1893304
 
 
0.0890414
 
 
2.126318
 
 
0.0352831
 
 
0.0132455
 
 
0.3654153
 
 
 
 
0.1894142
 
 
0.0889088
 
 
2.130432
 
 
0.0349358
 
 
0.0135916
 
 
0.3652368
 
 
 
 
0.1890185
 
 
0.0880312
 
 
2.147177
 
 
0.0335527
 
 
0.0149315
 
 
0.3631055
 
 
 
 
0.1852496
 
 
0.0794997
 
 
2.330194
 
 
0.0212675
 
 
0.0280342
 
 
0.3424651
 
 
 
 
0.1885648
 
 
0.0831488
 
 
2.267800
 
 
0.0249177
 
 
0.0241331
 
 
0.3529965
 
 
 
 
0.1896607
 
 
0.0875926
 
 
2.165258
 
 
0.0321126
 
 
0.0164409
 
 
0.3628804
 
 
 
 
0.1980547
 
 
0.0903518
 
 
2.192039
 
 
0.0300781
 
 
0.0193785
 
 
0.3767310
 
 
 
 
0.1900426
 
 
0.0910718
 
 
2.086734
 
 
0.0387792
 
 
0.0099426
 
 
0.3701427
 
 
 
 
0.1876850
 
 
0.0879293
 
 
2.134500
 
 
0.0345953
 
 
0.0137995
 
 
0.3615704
 
 
 
 
0.1898034
 
 
0.0880563
 
 
2.155477
 
 
0.0328848
 
 
0.0156666
 
 
0.3639401
 
 
 
 
0.1900343
 
 
0.0856026
 
 
2.219958
 
 
0.0280774
 
 
0.0207499
 
 
0.3593187
 
 
 
 
0.1890736
 
 
0.0848397
 
 
2.228598
 
 
0.0274822
 
 
0.0212979
 
 
0.3568494
 
 
 
 
0.1901475
 
 
0.0864046
 
 
2.200663
 
 
0.0294473
 
 
0.0192771
 
 
0.3610179
 
 
 
 
0.1905272
 
 
0.0877197
 
 
2.172001
 
 
0.0315895
 
 
0.0170562
 
 
0.3639982
 
 
 
 
0.1900132
 
 
0.0880952
 
 
2.156906
 
 
0.0327710
 
 
0.0157995
 
 
0.3642269
 
 
 
 
0.1897120
 
 
0.0879491
 
 
2.157066
 
 
0.0327583
 
 
0.0157873
 
 
0.3636367
 
 
 
 
0.1898449
 
 
0.0881269
 
 
2.154223
 
 
0.0329850
 
 
0.0155687
 
 
0.3641212
 
 
 
 
0.1901572
 
 
0.0884399
 
 
2.150128
 
 
0.0333139
 
 
0.0152618
 
 
0.3650525
 
 
 
 
0.1894199
 
 
0.0877824
 
 
2.157835
 
 
0.0326972
 
 
0.0158249
 
 
0.3630148
 
 
 
 
0.1895812
 
 
0.0876759
 
 
2.162295
 
 
0.0323448
 
 
0.0161968
 
 
0.3629656
 
 
 
 
0.1935150
 
 
0.0922495
 
 
2.097736
 
 
0.0377789
 
 
0.0110861
 
 
0.3759439
 
 
 
 
0.1901109
 
 
0.0868494
 
 
2.188972
 
 
0.0303053
 
 
0.0183609
 
 
0.3618609
 
 
 
 
0.1898975
 
 
0.0887416
 
 
2.139893
 
 
0.0341484
 
 
0.0144056
 
 
0.3653894
 
 
 
 
0.1901953
 
 
0.0921256
 
 
2.064522
 
 
0.0408684
 
 
0.0080113
 
 
0.3723792
 
 
 
 
0.1893608
 
 
0.0895556
 
 
2.114450
 
 
0.0363015
 
 
0.0122592
 
 
0.3664625
 
 
 
 
 
       ggplot (results.Leave1speciesout) +  stat_dots ( aes ( x= Estimate),  alpha=  0.8 ,  dotsize=  1.5 ,  shape=  16 ,  show.legend= F,  col=  "darkcyan" )  # Distribution of overall mean after removal of one species at a time     
   
      results.Leave1speciesout  %>%   
                              summarise ( estimate=   mean (Estimate),  # calculate the mean coefficients across the models generated  
                                        se=  mean (SE),  
                                        zval=  mean (zval),  
                                        pval=  mean (pval),  
                                        ci.lb=  mean (ci.lb),  
                                        ci.ub=  mean (ci.ub))  %>%   
                                                              kable () %>%   kable_styling ( "striped" ,  position=  "left" )    
 
 
 
 
estimate
 
 
se
 
 
zval
 
 
pval
 
 
ci.lb
 
 
ci.ub
 
 
 
 
 
 
0.1897393
 
 
0.0879348
 
 
2.159462
 
 
0.0328797
 
 
0.0158429
 
 
0.3636356
 
 
 
 
 
 
  
 
 
 
  Analyses with initial
designs only  
 
 Habitat 
 
 Individual coefficients 
      d.initial  <-   filter (d, brought_common_temp  ==   "no" ) 
   mod.initial_habitat  <-   run.model (d.initial,  ~ habitat  -   1 ) 
    summary (mod.initial_habitat)    
  ## 
## Multivariate Meta-Analysis Model (k = 866; method: REML)
## 
##    logLik   Deviance        AIC        BIC       AICc  ​ 
## -207.4173   414.8347   424.8347   448.6425   424.9046   
## 
## Variance Components:
## 
##             estim    sqrt  nlvls  fixed      factor    R 
## sigma^2.1  0.0057  0.0758    121     no  species_ID   no 
## sigma^2.2  0.0074  0.0861    121     no   phylogeny  yes 
## sigma^2.3  0.0693  0.2633    866     no       es_ID   no 
## 
## Test for Residual Heterogeneity:
## QE(df = 864) = 37465.3848, p-val < .0001
## 
## Test of Moderators (coefficients 1:2):
## F(df1 = 2, df2 = 119) = 12.3613, p-val < .0001
## 
## Model Results:
## 
##                     estimate      se    tval   df    pval    ci.lb   ci.ub     ​ 
## habitataquatic        0.2334  0.0511  4.5674  119  <.0001   0.1322  0.3346  *** 
## habitatterrestrial    0.0727  0.0767  0.9473  119  0.3454  -0.0793  0.2246      
## 
## ---
## Signif. codes:  0 '***' 0.001 '**' 0.01 '*' 0.05 '.' 0.1 ' ' 1  
       mod_results (mod.initial_habitat,  mod =   "habitat" ,  data =  d.initial,  group =   "species_ID" )    
  ##          name   estimate     lowerCL   upperCL    lowerPR   upperPR
## 1     Aquatic 0.23341363  0.13222224 0.3346050 -0.3441887 0.8110160
## 2 Terrestrial 0.07268808 -0.07925124 0.2246274 -0.5159292 0.6613054  
       my.orchard (mod.initial_habitat,  xlab =   "dARR" ,  mod =   "habitat" ,  alpha =   0.1 ,  data =  d.initial, 
        group =   "species_ID" ,  whisker =   0.07 )    
   
 
 
 Heteroscedasticity check 
       qplot ( y =   sqrt ( residuals (mod.initial_habitat) ^  2 ),  x =   fitted (mod.initial_habitat))  +  
        geom_point ()  +   geom_smooth ( method =   "lm" )  +   geom_hline ( yintercept =   0 ,  colour =   "red" )    
   
 
 
 Individual coefficients (with heteroscedasticity) 
      VCV_initial  <-   make_VCV_matrix (d.initial,  V =   "Var_dARR" ,  cluster =   "shared_trt_ID" , 
        obs =   "es_ID" ) 
   mod.initial_habitat_het  <-   rma.mv ( yi =  dARR,  V =  VCV_initial,  mods =   ~ habitat  -   1 , 
        method =   "REML" ,  test =   "t" ,  dfs =   "contain" ,  random =   list ( ~  1   |  species_ID, 
            ~  1   |  phylogeny,  ~ habitat  |  es_ID),  struct =   "HCS" ,  rho =   0 ,  R =   list ( phylogeny =  phylo_matrix), 
        data =  d.initial,  sparse =   TRUE ) 
    AICc (mod.initial_habitat)    
  ## [1] 424.9044  
       AICc (mod.initial_habitat_het)    
  ## [1] 341.5476  
       summary (mod.initial_habitat_het)    
  ## 
## Multivariate Meta-Analysis Model (k = 866; method: REML)
## 
##    logLik   Deviance        AIC        BIC       AICc  ​ 
## -164.7249   329.4498   341.4498   370.0192   341.5478   
## 
## Variance Components:
## 
##             estim    sqrt  nlvls  fixed      factor    R 
## sigma^2.1  0.0060  0.0777    121     no  species_ID   no 
## sigma^2.2  0.0063  0.0796    121     no   phylogeny  yes 
## 
## outer factor: es_ID   (nlvls = 866)
## inner factor: habitat (nlvls = 2)
## 
##             estim    sqrt  k.lvl  fixed        level 
## tau^2.1    0.0776  0.2785    781     no      aquatic 
## tau^2.2    0.0085  0.0924     85     no  terrestrial 
## rho        0.0000                   yes              
## 
## Test for Residual Heterogeneity:
## QE(df = 864) = 37465.3848, p-val < .0001
## 
## Test of Moderators (coefficients 1:2):
## F(df1 = 2, df2 = 119) = 14.5396, p-val < .0001
## 
## Model Results:
## 
##                     estimate      se    tval   df    pval    ci.lb   ci.ub     ​ 
## habitataquatic        0.2341  0.0480  4.8775  119  <.0001   0.1391  0.3292  *** 
## habitatterrestrial    0.0738  0.0645  1.1447  119  0.2546  -0.0539  0.2016      
## 
## ---
## Signif. codes:  0 '***' 0.001 '**' 0.01 '*' 0.05 '.' 0.1 ' ' 1  
       mod_results (mod.initial_habitat_het,  mod =   "habitat" ,  data =  d.initial,  group =   "species_ID" )    
  ##          name   estimate     lowerCL   upperCL    lowerPR   upperPR
## 1     Aquatic 0.23412219  0.13907723 0.3291672 -0.3672915 0.8355359
## 2 Terrestrial 0.07384853 -0.05389323 0.2015903 -0.2396942 0.3873912  
       r2_ml (mod.initial_habitat_het)    
  ##    R2_marginal R2_conditional 
##      0.1553241      0.5672050  
       my.orchard (mod.initial_habitat_het,  mod =   "habitat" ,  xlab =   "dARR" ,  alpha =   0.1 , 
        data =  d.initial,  group =   "species_ID" ,  whisker =   0.07 )    
   
 
 
 Mean initial effects with habitat weighted equally 
      mod.adjusted_initial  <-   mod_results ( model =  mod.initial_habitat_het,  data =  d.initial, 
        mod =   "1" ,  weights =   "equal" ,  group =   "species_ID" ) 
   mod.adjusted_initial $ mod_table    
  ##      name  estimate    lowerCL   upperCL    lowerPR   upperPR
## 1 Intrcpt 0.1539854 0.05381858 0.2541521 -0.4257827 0.7337534  
       my.orchard (mod.adjusted_initial,  mod =   "1" ,  xlab =   "dARR" ,  alpha =   0.1 ,  data =  d.initial, 
        group =   "species_ID" ,  whisker =   0.04 )    
   
 
 
 
 Taxonomic groups 
 
 Individual coefficients 
      mod.initial_taxa  <-   run.model (d.initial,  ~ taxonomic_group  -   1 ) 
    summary (mod.initial_taxa)    
  ## 
## Multivariate Meta-Analysis Model (k = 866; method: REML)
## 
##    logLik   Deviance        AIC        BIC       AICc  ​ 
## -204.7335   409.4670   425.4670   463.5318   425.6361   
## 
## Variance Components:
## 
##             estim    sqrt  nlvls  fixed      factor    R 
## sigma^2.1  0.0051  0.0714    121     no  species_ID   no 
## sigma^2.2  0.0139  0.1180    121     no   phylogeny  yes 
## sigma^2.3  0.0692  0.2631    866     no       es_ID   no 
## 
## Test for Residual Heterogeneity:
## QE(df = 861) = 37180.6026, p-val < .0001
## 
## Test of Moderators (coefficients 1:5):
## F(df1 = 5, df2 = 116) = 3.4192, p-val = 0.0064
## 
## Model Results:
## 
##                                          estimate      se    tval   df    pval​ 
## taxonomic_groupamphibian                   0.2247  0.1215  1.8496  116  0.0669 
## taxonomic_groupaquatic invertebrate        0.1947  0.0928  2.0995  116  0.0379 
## taxonomic_groupfish                        0.2843  0.0915  3.1073  116  0.0024 
## taxonomic_groupreptile                     0.1580  0.1462  1.0810  116  0.2819 
## taxonomic_groupterrestrial invertebrate    0.0306  0.1089  0.2809  116  0.7793 
##                                            ci.lb   ci.ub 
## taxonomic_groupamphibian                 -0.0159  0.4652   . 
## taxonomic_groupaquatic invertebrate       0.0110  0.3785   * 
## taxonomic_groupfish                       0.1031  0.4656  ** 
## taxonomic_groupreptile                   -0.1315  0.4476     
## taxonomic_groupterrestrial invertebrate  -0.1851  0.2462     
## 
## ---
## Signif. codes:  0 '***' 0.001 '**' 0.01 '*' 0.05 '.' 0.1 ' ' 1  
       mod_results (mod.initial_taxa,  mod =   "taxonomic_group" ,  data =  d.initial,  group =   "species_ID" )    
  ##                       name   estimate     lowerCL   upperCL    lowerPR
## 1                Amphibian 0.22465082 -0.01590805 0.4652097 -0.4110431
## 2     Aquatic invertebrate 0.19474979  0.01102876 0.3784708 -0.4216848
## 3                     Fish 0.28434087  0.10309755 0.4655842 -0.3313598
## 4                  Reptile 0.15803905 -0.13152214 0.4476002 -0.4977688
## 5 Terrestrial invertebrate 0.03058815 -0.18506361 0.2462399 -0.5961047
##     upperPR
## 1 0.8603448
## 2 0.8111844
## 3 0.9000416
## 4 0.8138469
## 5 0.6572810  
       my.orchard (mod.initial_taxa,  xlab =   "dARR" ,  mod =   "taxonomic_group" ,  data =  d.initial, 
        alpha =   0.1 ,  group =   "species_ID" ,  whisker =   0.09 )    
   
 
 
 Heteroscedasticity check 
       qplot ( y =   sqrt ( residuals (mod.initial_taxa) ^  2 ),  x =   fitted (mod.initial_taxa))  +   geom_point ()  +  
        geom_smooth ( method =   "lm" )  +   geom_hline ( yintercept =   0 ,  colour =   "red" )    
   
 
 
 Individual coefficients (with heteroscedasticity) 
      mod.initial_taxa_het  <-   rma.mv ( yi =  dARR,  V =  VCV_initial,  mods =   ~ taxonomic_group  -  
        1 ,  method =   "REML" ,  test =   "t" ,  dfs =   "contain" ,  random =   list ( ~  1   |  species_ID, 
        ~  1   |  phylogeny,  ~ taxonomic_group  |  es_ID),  struct =   "HCS" ,  rho =   0 ,  R =   list ( phylogeny =  phylo_matrix), 
        data =  d.initial,  sparse =   TRUE ) 
    AICc (mod.initial_taxa)    
  ## [1] 425.6351  
       AICc (mod.initial_taxa_het)    
  ## [1] 162.6418  
       summary (mod.initial_taxa_het)    
  ## 
## Multivariate Meta-Analysis Model (k = 866; method: REML)
## 
##   logLik  Deviance       AIC       BIC      AICc  ​ 
## -69.1380  138.2760  162.2760  219.3732  162.6440   
## 
## Variance Components:
## 
##             estim    sqrt  nlvls  fixed      factor    R 
## sigma^2.1  0.0051  0.0716    121     no  species_ID   no 
## sigma^2.2  0.0210  0.1449    121     no   phylogeny  yes 
## 
## outer factor: es_ID           (nlvls = 866)
## inner factor: taxonomic_group (nlvls = 5)
## 
##             estim    sqrt  k.lvl  fixed                     level 
## tau^2.1    0.1977  0.4446     65     no                 amphibian 
## tau^2.2    0.1463  0.3824    208     no      aquatic invertebrate 
## tau^2.3    0.0318  0.1783    496     no                      fish 
## tau^2.4    0.0046  0.0678     12     no                   reptile 
## tau^2.5    0.0085  0.0921     85     no  terrestrial invertebrate 
## rho        0.0000                   yes                           
## 
## Test for Residual Heterogeneity:
## QE(df = 861) = 37180.6026, p-val < .0001
## 
## Test of Moderators (coefficients 1:5):
## F(df1 = 5, df2 = 116) = 2.8829, p-val = 0.0172
## 
## Model Results:
## 
##                                          estimate      se    tval   df    pval​ 
## taxonomic_groupamphibian                   0.2165  0.1534  1.4113  116  0.1608 
## taxonomic_groupaquatic invertebrate        0.2024  0.1154  1.7535  116  0.0822 
## taxonomic_groupfish                        0.2959  0.1084  2.7285  116  0.0074 
## taxonomic_groupreptile                     0.1432  0.1495  0.9577  116  0.3402 
## taxonomic_groupterrestrial invertebrate    0.0309  0.1253  0.2468  116  0.8055 
##                                            ci.lb   ci.ub 
## taxonomic_groupamphibian                 -0.0873  0.5203     
## taxonomic_groupaquatic invertebrate      -0.0262  0.4310   . 
## taxonomic_groupfish                       0.0811  0.5106  ** 
## taxonomic_groupreptile                   -0.1529  0.4393     
## taxonomic_groupterrestrial invertebrate  -0.2172  0.2790     
## 
## ---
## Signif. codes:  0 '***' 0.001 '**' 0.01 '*' 0.05 '.' 0.1 ' ' 1  
       mod_results (mod.initial_taxa_het,  mod =   "taxonomic_group" ,  data =  d.initial,  group =   "species_ID" )    
  ##                       name   estimate     lowerCL   upperCL    lowerPR
## 1                Amphibian 0.21650061 -0.08734726 0.5203485 -0.7685470
## 2     Aquatic invertebrate 0.20238847 -0.02621472 0.4309917 -0.6511378
## 3                     Fish 0.29585838  0.08109085 0.5106259 -0.2269040
## 4                  Reptile 0.14317390 -0.15290973 0.4392575 -0.3131056
## 5 Terrestrial invertebrate 0.03091705 -0.21721312 0.2790472 -0.4133293
##     upperPR
## 1 1.2015482
## 2 1.0559147
## 3 0.8186208
## 4 0.5994534
## 5 0.4751634  
       r2_ml (mod.initial_taxa_het)    
  ##    R2_marginal R2_conditional 
##      0.2015604      0.3583558  
       my.orchard (mod.initial_taxa_het,  mod =   "taxonomic_group" ,  xlab =   "dARR" ,  alpha =   0.1 , 
        data =  d.initial,  group =   "species_ID" ,  whisker =   0.09 )    
   
 
 
 
 Type of metric 
 
 Individual coefficients 
      mod.initial_metric  <-   run.model (d.initial,  ~ metric  -   1 ) 
    summary (mod.initial_metric)    
  ## 
## Multivariate Meta-Analysis Model (k = 866; method: REML)
## 
##    logLik   Deviance        AIC        BIC       AICc  ​ 
## -209.0317   418.0635   428.0635   451.8713   428.1334   
## 
## Variance Components:
## 
##             estim    sqrt  nlvls  fixed      factor    R 
## sigma^2.1  0.0057  0.0754    121     no  species_ID   no 
## sigma^2.2  0.0159  0.1263    121     no   phylogeny  yes 
## sigma^2.3  0.0690  0.2627    866     no       es_ID   no 
## 
## Test for Residual Heterogeneity:
## QE(df = 864) = 47790.0581, p-val < .0001
## 
## Test of Moderators (coefficients 1:2):
## F(df1 = 2, df2 = 864) = 6.0643, p-val = 0.0024
## 
## Model Results:
## 
##              estimate      se    tval   df    pval   ci.lb   ci.ub    ​ 
## metricCTmax    0.2269  0.0722  3.1450  864  0.0017  0.0853  0.3685  ** 
## metricLT50     0.1753  0.0753  2.3275  864  0.0202  0.0275  0.3232   * 
## 
## ---
## Signif. codes:  0 '***' 0.001 '**' 0.01 '*' 0.05 '.' 0.1 ' ' 1  
       mod_results (mod.initial_metric,  mod =   "metric" ,  data =  d.initial,  group =   "species_ID" )    
  ##    name  estimate    lowerCL   upperCL    lowerPR   upperPR
## 1 CTmax 0.2269223 0.08530761 0.3685370 -0.3807252 0.8345698
## 2  LT50 0.1753155 0.02747981 0.3231512 -0.4338118 0.7844429  
       my.orchard (mod.initial_metric,  xlab =   "dARR" ,  mod =   "metric" ,  alpha =   0.1 ,  data =  d.initial, 
        group =   "species_ID" ,  whisker =   0.07 )    
   
 
 
 Heteroscedasticity check 
       qplot ( y =   sqrt ( residuals (mod.initial_metric) ^  2 ),  x =   fitted (mod.initial_metric))  +  
        geom_point ()  +   geom_smooth ( method =   "lm" )  +   geom_hline ( yintercept =   0 ,  colour =   "red" )    
   
 
 
 Individual coefficients (with heteroscedasticity) 
      mod.initial_metric_het  <-   rma.mv ( yi =  dARR,  V =  VCV_initial,  mods =   ~ metric  -   1 , 
        method =   "REML" ,  test =   "t" ,  dfs =   "contain" ,  random =   list ( ~  1   |  species_ID, 
            ~  1   |  phylogeny,  ~ metric  |  es_ID),  struct =   "HCS" ,  rho =   0 ,  R =   list ( phylogeny =  phylo_matrix), 
        data =  d.initial,  sparse =   TRUE ) 
    AICc (mod.initial_metric)    
  ## [1] 428.1332  
       AICc (mod.initial_metric_het)    
  ## [1] 414.7547  
       summary (mod.initial_metric_het)    
  ## 
## Multivariate Meta-Analysis Model (k = 866; method: REML)
## 
##    logLik   Deviance        AIC        BIC       AICc  ​ 
## -201.3285   402.6569   414.6569   443.2264   414.7550   
## 
## Variance Components:
## 
##             estim    sqrt  nlvls  fixed      factor    R 
## sigma^2.1  0.0061  0.0783    121     no  species_ID   no 
## sigma^2.2  0.0159  0.1260    121     no   phylogeny  yes 
## 
## outer factor: es_ID  (nlvls = 866)
## inner factor: metric (nlvls = 2)
## 
##             estim    sqrt  k.lvl  fixed  level 
## tau^2.1    0.0763  0.2762    647     no  CTmax 
## tau^2.2    0.0414  0.2035    219     no   LT50 
## rho        0.0000                   yes        
## 
## Test for Residual Heterogeneity:
## QE(df = 864) = 47790.0581, p-val < .0001
## 
## Test of Moderators (coefficients 1:2):
## F(df1 = 2, df2 = 864) = 6.3190, p-val = 0.0019
## 
## Model Results:
## 
##              estimate      se    tval   df    pval   ci.lb   ci.ub    ​ 
## metricCTmax    0.2250  0.0720  3.1247  864  0.0018  0.0837  0.3664  ** 
## metricLT50     0.1718  0.0742  2.3156  864  0.0208  0.0262  0.3173   * 
## 
## ---
## Signif. codes:  0 '***' 0.001 '**' 0.01 '*' 0.05 '.' 0.1 ' ' 1  
       mod_results (mod.initial_metric_het,  mod =   "metric" ,  data =  d.initial,  group =   "species_ID" )    
  ##    name  estimate    lowerCL   upperCL    lowerPR   upperPR
## 1 CTmax 0.2250164 0.08367535 0.3663575 -0.4062437 0.8562766
## 2  LT50 0.1717566 0.02617401 0.3173391 -0.3433756 0.6868888  
       r2_ml (mod.initial_metric_het)    
  ##    R2_marginal R2_conditional 
##     0.02381791     0.29568891  
       my.orchard (mod.initial_metric_het,  mod =   "metric" ,  xlab =   "dARR" ,  alpha =   0.1 ,  data =  d.initial, 
        group =   "species_ID" ,  whisker =   0.07 )    
   
 
 
 
 Heating rate 
 
 Run model and plot results 
      mod.initial_ramping  <-   run.model (d.initial,  ~  scale (ramping,  center =  T,  scale =  F)) 
    summary (mod.initial_ramping)    
  ## 
## Multivariate Meta-Analysis Model (k = 653; method: REML)
## 
##    logLik   Deviance        AIC        BIC       AICc  ​ 
## -147.7601   295.5202   305.5202   327.9127   305.6132   
## 
## Variance Components:
## 
##             estim    sqrt  nlvls  fixed      factor    R 
## sigma^2.1  0.0009  0.0301     99     no  species_ID   no 
## sigma^2.2  0.0308  0.1755     99     no   phylogeny  yes 
## sigma^2.3  0.0747  0.2732    653     no       es_ID   no 
## 
## Test for Residual Heterogeneity:
## QE(df = 651) = 41157.4313, p-val < .0001
## 
## Test of Moderators (coefficient 2):
## F(df1 = 1, df2 = 651) = 0.1759, p-val = 0.6750
## 
## Model Results:
## 
##                                        estimate      se    tval   df    pval​ 
## intrcpt                                  0.2337  0.0990  2.3596   97  0.0203 
## scale(ramping, center = T, scale = F)    0.0137  0.0327  0.4195  651  0.6750 
##                                          ci.lb   ci.ub 
## intrcpt                                 0.0371  0.4303  * 
## scale(ramping, center = T, scale = F)  -0.0505  0.0779    
## 
## ---
## Signif. codes:  0 '***' 0.001 '**' 0.01 '*' 0.05 '.' 0.1 ' ' 1  
      d.initial_ramping  <-   filter (d, ramping  !=   "NA"   &  brought_common_temp  ==   "no" ) 
    plot_continuous (d.initial_ramping, mod.initial_ramping, d.initial_ramping $ ramping, 
        "Heating rate (degrees/min)" )    
   
 
 
 
  
 
 
 
  Analyses with persistent
designs only  
 
 Habitat 
 
 Individual coefficients 
      d.persistent  <-   filter (d, brought_common_temp  ==   "yes" )   # Only keep persistent data  
   mod.persistent_habitat  <-   run.model (d.persistent,  ~ habitat  -   1 ) 
    summary (mod.persistent_habitat)    
  ## 
## Multivariate Meta-Analysis Model (k = 223; method: REML)
## 
##   logLik  Deviance       AIC       BIC      AICc  ​ 
##  28.5430  -57.0860  -47.0860  -30.0952  -46.8069   
## 
## Variance Components:
## 
##             estim    sqrt  nlvls  fixed      factor    R 
## sigma^2.1  0.0000  0.0000     23     no  species_ID   no 
## sigma^2.2  0.0311  0.1765     23     no   phylogeny  yes 
## sigma^2.3  0.0214  0.1461    223     no       es_ID   no 
## 
## Test for Residual Heterogeneity:
## QE(df = 221) = 6128.2502, p-val < .0001
## 
## Test of Moderators (coefficients 1:2):
## F(df1 = 2, df2 = 21) = 0.1327, p-val = 0.8765
## 
## Model Results:
## 
##                     estimate      se    tval  df    pval    ci.lb   ci.ub   ​ 
## habitataquatic        0.0533  0.1049  0.5078  21  0.6169  -0.1649  0.2715    
## habitatterrestrial    0.0519  0.1141  0.4549  21  0.6539  -0.1854  0.2891    
## 
## ---
## Signif. codes:  0 '***' 0.001 '**' 0.01 '*' 0.05 '.' 0.1 ' ' 1  
       mod_results (mod.persistent_habitat,  mod =   "habitat" ,  data =  d.persistent,  group =   "species_ID" )    
  ##          name   estimate    lowerCL   upperCL    lowerPR   upperPR
## 1     Aquatic 0.05329268 -0.1649379 0.2715232 -0.4708093 0.5773947
## 2 Terrestrial 0.05189110 -0.1853570 0.2891393 -0.4804105 0.5841927  
       my.orchard (mod.persistent_habitat,  xlab =   "dARR" ,  mod =   "habitat" ,  alpha =   0.1 ,  data =  d.persistent, 
        group =   "species_ID" ,  whisker =   0.07 )    
   
 
 
 Heteroscedasticity check 
       qplot ( y =   sqrt ( residuals (mod.persistent_habitat) ^  2 ),  x =   fitted (mod.persistent_habitat))  +  
        geom_point ()  +   geom_smooth ( method =   "lm" )  +   geom_hline ( yintercept =   0 ,  colour =   "red" )    
   
 
 
 Individual coefficients (with heteroscedasticity) 
      VCV_persistent  <-   make_VCV_matrix (d.persistent,  V =   "Var_dARR" ,  cluster =   "shared_trt_ID" , 
        obs =   "es_ID" )   # Re calculate VCV matrix with the new data  
   mod.persistent_habitat_het  <-   rma.mv ( yi =  dARR,  V =  VCV_persistent,  mods =   ~ habitat  -  
        1 ,  method =   "REML" ,  test =   "t" ,  dfs =   "contain" ,  random =   list ( ~  1   |  species_ID, 
        ~  1   |  phylogeny,  ~ habitat  |  es_ID),  struct =   "HCS" ,  rho =   0 ,  R =   list ( phylogeny =  phylo_matrix), 
        data =  d.persistent,  sparse =   TRUE ) 
    AICc (mod.persistent_habitat)    
  ## [1] -46.80952  
       AICc (mod.persistent_habitat_het)    
  ## [1] -64.95965  
       summary (mod.persistent_habitat_het)    
  ## 
## Multivariate Meta-Analysis Model (k = 223; method: REML)
## 
##   logLik  Deviance       AIC       BIC      AICc  ​ 
##  38.6743  -77.3485  -65.3485  -44.9596  -64.9560   
## 
## Variance Components:
## 
##             estim    sqrt  nlvls  fixed      factor    R 
## sigma^2.1  0.0003  0.0167     23     no  species_ID   no 
## sigma^2.2  0.0272  0.1650     23     no   phylogeny  yes 
## 
## outer factor: es_ID   (nlvls = 223)
## inner factor: habitat (nlvls = 2)
## 
##             estim    sqrt  k.lvl  fixed        level 
## tau^2.1    0.0309  0.1758    148     no      aquatic 
## tau^2.2    0.0079  0.0889     75     no  terrestrial 
## rho        0.0000                   yes              
## 
## Test for Residual Heterogeneity:
## QE(df = 221) = 6128.2502, p-val < .0001
## 
## Test of Moderators (coefficients 1:2):
## F(df1 = 2, df2 = 21) = 0.1348, p-val = 0.8746
## 
## Model Results:
## 
##                     estimate      se    tval  df    pval    ci.lb   ci.ub   ​ 
## habitataquatic        0.0507  0.0998  0.5075  21  0.6171  -0.1569  0.2582    
## habitatterrestrial    0.0492  0.1066  0.4616  21  0.6491  -0.1726  0.2710    
## 
## ---
## Signif. codes:  0 '***' 0.001 '**' 0.01 '*' 0.05 '.' 0.1 ' ' 1  
       mod_results (mod.persistent_habitat_het,  mod =   "habitat" ,  data =  d.persistent,  group =   "species_ID" )    
  ##          name   estimate    lowerCL   upperCL    lowerPR   upperPR
## 1     Aquatic 0.05065728 -0.1569176 0.2582322 -0.4930975 0.5944120
## 2 Terrestrial 0.04922949 -0.1725603 0.2710193 -0.4005826 0.4990416  
       r2_ml (mod.persistent_habitat_het)    
  ##    R2_marginal R2_conditional 
##   1.662009e-05   1.015787e-02  
       my.orchard (mod.persistent_habitat_het,  mod =   "habitat" ,  xlab =   "dARR" ,  alpha =   0.1 , 
        data =  d.persistent,  group =   "species_ID" ,  whisker =   0.07 )    
   
 
 
 Mean persistent effects with habitat weighted equally 
      mod.adjusted_persistent  <-   mod_results ( model =  mod.persistent_habitat_het,  data =  d.persistent, 
        mod =   "1" ,  weights =   "equal" ,  group =   "species_ID" ) 
   mod.adjusted_persistent $ mod_table    
  ##      name   estimate    lowerCL   upperCL    lowerPR   upperPR
## 1 Intrcpt 0.04994338 -0.1521334 0.2520201 -0.4599315 0.5598183  
       my.orchard (mod.adjusted_persistent,  mod =   "1" ,  xlab =   "dARR" ,  alpha =   0.1 ,  data =  d.persistent, 
        group =   "species_ID" ,  whisker =   0.04 )    
   
 
 
 
 Taxonomic groups 
 
 Individual coefficients 
      mod.persistent_taxa  <-   run.model (d.persistent,  ~ taxonomic_group  -   1 ) 
    summary (mod.persistent_taxa)    
  ## 
## Multivariate Meta-Analysis Model (k = 223; method: REML)
## 
##   logLik  Deviance       AIC       BIC      AICc  ​ 
##  30.2273  -60.4546  -44.4546  -17.3786  -43.7656   
## 
## Variance Components:
## 
##             estim    sqrt  nlvls  fixed      factor    R 
## sigma^2.1  0.0000  0.0000     23     no  species_ID   no 
## sigma^2.2  0.0402  0.2006     23     no   phylogeny  yes 
## sigma^2.3  0.0214  0.1463    223     no       es_ID   no 
## 
## Test for Residual Heterogeneity:
## QE(df = 218) = 5315.6964, p-val < .0001
## 
## Test of Moderators (coefficients 1:5):
## F(df1 = 5, df2 = 18) = 0.2834, p-val = 0.9161
## 
## Model Results:
## 
##                                          estimate      se     tval  df    pval​ 
## taxonomic_groupamphibian                   0.0091  0.2120   0.0427  18  0.9664 
## taxonomic_groupaquatic invertebrate        0.1523  0.1518   1.0029  18  0.3292 
## taxonomic_groupfish                       -0.0279  0.1608  -0.1735  18  0.8642 
## taxonomic_groupreptile                    -0.0511  0.1999  -0.2554  18  0.8013 
## taxonomic_groupterrestrial invertebrate    0.1190  0.1647   0.7223  18  0.4794 
##                                            ci.lb   ci.ub 
## taxonomic_groupamphibian                 -0.4363  0.4544    
## taxonomic_groupaquatic invertebrate      -0.1667  0.4713    
## taxonomic_groupfish                      -0.3657  0.3099    
## taxonomic_groupreptile                   -0.4710  0.3689    
## taxonomic_groupterrestrial invertebrate  -0.2270  0.4650    
## 
## ---
## Signif. codes:  0 '***' 0.001 '**' 0.01 '*' 0.05 '.' 0.1 ' ' 1  
       mod_results (mod.persistent_taxa,  mod =   "taxonomic_group" ,  data =  d.persistent,  group =   "species_ID" )    
  ##                       name     estimate    lowerCL   upperCL    lowerPR
## 1                Amphibian  0.009050625 -0.4363420 0.4544433 -0.6768539
## 2     Aquatic invertebrate  0.152282841 -0.1667180 0.4712837 -0.4591515
## 3                     Fish -0.027894294 -0.3657346 0.3099460 -0.6493655
## 4                  Reptile -0.051058808 -0.4710038 0.3688862 -0.7207186
## 5 Terrestrial invertebrate  0.118957656 -0.2270469 0.4649622 -0.5069893
##     upperPR
## 1 0.6949552
## 2 0.7637172
## 3 0.5935769
## 4 0.6186010
## 5 0.7449046  
       my.orchard (mod.persistent_taxa,  xlab =   "dARR" ,  mod =   "taxonomic_group" ,  alpha =   0.1 , 
        data =  d.persistent,  group =   "species_ID" ,  whisker =   0.09 )    
   
 
 
 Heteroscedasticity check 
       qplot ( y =   sqrt ( residuals (mod.persistent_taxa) ^  2 ),  x =   fitted (mod.persistent_taxa))  +  
        geom_point ()  +   geom_smooth ( method =   "lm" )  +   geom_hline ( yintercept =   0 ,  colour =   "red" )    
   
 
 
 Individual coefficients (with heteroscedasticity) 
      mod.persistent_taxa_het  <-   rma.mv ( yi =  dARR,  V =  VCV_persistent,  mods =   ~ taxonomic_group  -  
        1 ,  method =   "REML" ,  test =   "t" ,  dfs =   "contain" ,  random =   list ( ~  1   |  species_ID, 
        ~  1   |  phylogeny,  ~ taxonomic_group  |  es_ID),  struct =   "HCS" ,  rho =   0 ,  R =   list ( phylogeny =  phylo_matrix), 
        data =  d.persistent,  sparse =   TRUE ) 
    AICc (mod.persistent_taxa)    
  ## [1] -43.78171  
       AICc (mod.persistent_taxa_het)    
  ## [1] -75.11488  
       summary (mod.persistent_taxa_het)    
  ## 
## Multivariate Meta-Analysis Model (k = 223; method: REML)
## 
##    logLik   Deviance        AIC        BIC       AICc  ​ 
##   50.3003  -100.6006   -76.6006   -35.9866   -75.0786   
## 
## Variance Components:
## 
##             estim    sqrt  nlvls  fixed      factor    R 
## sigma^2.1  0.0005  0.0216     23     no  species_ID   no 
## sigma^2.2  0.0331  0.1821     23     no   phylogeny  yes 
## 
## outer factor: es_ID           (nlvls = 223)
## inner factor: taxonomic_group (nlvls = 5)
## 
##             estim    sqrt  k.lvl  fixed                     level 
## tau^2.1    0.0626  0.2501      6     no                 amphibian 
## tau^2.2    0.0081  0.0899     13     no      aquatic invertebrate 
## tau^2.3    0.0332  0.1822    127     no                      fish 
## tau^2.4    0.0309  0.1758     15     no                   reptile 
## tau^2.5    0.0046  0.0675     62     no  terrestrial invertebrate 
## rho        0.0000                   yes                           
## 
## Test for Residual Heterogeneity:
## QE(df = 218) = 5315.6964, p-val < .0001
## 
## Test of Moderators (coefficients 1:5):
## F(df1 = 5, df2 = 18) = 0.2830, p-val = 0.9163
## 
## Model Results:
## 
##                                          estimate      se     tval  df    pval​ 
## taxonomic_groupamphibian                   0.0050  0.2135   0.0235  18  0.9815 
## taxonomic_groupaquatic invertebrate        0.1358  0.1350   1.0063  18  0.3276 
## taxonomic_groupfish                       -0.0294  0.1469  -0.2003  18  0.8435 
## taxonomic_groupreptile                    -0.0474  0.1848  -0.2563  18  0.8006 
## taxonomic_groupterrestrial invertebrate    0.0968  0.1461   0.6625  18  0.5161 
##                                            ci.lb   ci.ub 
## taxonomic_groupamphibian                 -0.4436  0.4536    
## taxonomic_groupaquatic invertebrate      -0.1478  0.4194    
## taxonomic_groupfish                      -0.3380  0.2791    
## taxonomic_groupreptile                   -0.4357  0.3409    
## taxonomic_groupterrestrial invertebrate  -0.2102  0.4038    
## 
## ---
## Signif. codes:  0 '***' 0.001 '**' 0.01 '*' 0.05 '.' 0.1 ' ' 1  
       mod_results (mod.persistent_taxa_het,  mod =   "taxonomic_group" ,  data =  d.persistent, 
        group =   "species_ID" )    
  ##                       name     estimate    lowerCL   upperCL    lowerPR
## 1                Amphibian  0.005026263 -0.4435812 0.4536337 -0.7860333
## 2     Aquatic invertebrate  0.135833415 -0.1477534 0.4194202 -0.3784558
## 3                     Fish -0.029423673 -0.3379827 0.2791354 -0.6540624
## 4                  Reptile -0.047365567 -0.4356609 0.3409298 -0.7072814
## 5 Terrestrial invertebrate  0.096803200 -0.2101875 0.4037939 -0.4157655
##     upperPR
## 1 0.7960858
## 2 0.6501226
## 3 0.5952151
## 4 0.6125503
## 5 0.6093719  
       r2_ml (mod.persistent_taxa_het)    
  ##    R2_marginal R2_conditional 
##      0.1091420      0.1215625  
       my.orchard (mod.persistent_taxa_het,  mod =   "taxonomic_group" ,  xlab =   "dARR" ,  alpha =   0.1 , 
        data =  d.persistent,  group =   "species_ID" ,  whisker =   0.09 )    
   
 
 
 
 Type of metric 
 
 Individual coefficients 
      mod.persistent_metric  <-   run.model (d.persistent,  ~ metric  -   1 ) 
    summary (mod.persistent_metric)    
  ## 
## Multivariate Meta-Analysis Model (k = 223; method: REML)
## 
##   logLik  Deviance       AIC       BIC      AICc  ​ 
##  28.4387  -56.8773  -46.8773  -29.8865  -46.5982   
## 
## Variance Components:
## 
##             estim    sqrt  nlvls  fixed      factor    R 
## sigma^2.1  0.0000  0.0000     23     no  species_ID   no 
## sigma^2.2  0.0268  0.1636     23     no   phylogeny  yes 
## sigma^2.3  0.0215  0.1466    223     no       es_ID   no 
## 
## Test for Residual Heterogeneity:
## QE(df = 221) = 8562.9957, p-val < .0001
## 
## Test of Moderators (coefficients 1:2):
## F(df1 = 2, df2 = 221) = 0.5988, p-val = 0.5503
## 
## Model Results:
## 
##              estimate      se    tval   df    pval    ci.lb   ci.ub   ​ 
## metricCTmax    0.0441  0.0958  0.4602  221  0.6458  -0.1447  0.2329    
## metricLT50     0.1483  0.1396  1.0626  221  0.2891  -0.1267  0.4233    
## 
## ---
## Signif. codes:  0 '***' 0.001 '**' 0.01 '*' 0.05 '.' 0.1 ' ' 1  
       mod_results (mod.persistent_metric,  mod =   "metric" ,  data =  d.persistent,  group =   "species_ID" )    
  ##    name   estimate    lowerCL   upperCL    lowerPR   upperPR
## 1 CTmax 0.04408739 -0.1446967 0.2328715 -0.4282615 0.5164363
## 2  LT50 0.14829967 -0.1267440 0.4233433 -0.3646556 0.6612549  
       my.orchard (mod.persistent_metric,  xlab =   "dARR" ,  mod =   "metric" ,  alpha =   0.1 ,  data =  d.persistent, 
        group =   "species_ID" ,  whisker =   0.07 )    
   
 
 
 Heteroscedasticity check 
       qplot ( y =   sqrt ( residuals (mod.persistent_metric) ^  2 ),  x =   fitted (mod.persistent_metric))  +  
        geom_point ()  +   geom_smooth ( method =   "lm" )  +   geom_hline ( yintercept =   0 ,  colour =   "red" )    
   
 
 
 Individual coefficients (with heteroscedasticity) 
      mod.persistent_metric_het  <-   rma.mv ( yi =  dARR,  V =  VCV_persistent,  mods =   ~ metric  -  
        1 ,  method =   "REML" ,  test =   "t" ,  dfs =   "contain" ,  random =   list ( ~  1   |  species_ID, 
        ~  1   |  phylogeny,  ~ metric  |  es_ID),  struct =   "HCS" ,  rho =   0 ,  R =   list ( phylogeny =  phylo_matrix), 
        data =  d.persistent,  sparse =   TRUE ) 
    AICc (mod.persistent_metric)    
  ## [1] -46.60081  
       AICc (mod.persistent_metric_het)    
  ## [1] -44.8704  
       summary (mod.persistent_metric_het)    
  ## 
## Multivariate Meta-Analysis Model (k = 223; method: REML)
## 
##   logLik  Deviance       AIC       BIC      AICc  ​ 
##  28.6296  -57.2593  -45.2593  -24.8703  -44.8668   
## 
## Variance Components:
## 
##             estim    sqrt  nlvls  fixed      factor    R 
## sigma^2.1  0.0000  0.0000     23     no  species_ID   no 
## sigma^2.2  0.0281  0.1677     23     no   phylogeny  yes 
## 
## outer factor: es_ID  (nlvls = 223)
## inner factor: metric (nlvls = 2)
## 
##             estim    sqrt  k.lvl  fixed  level 
## tau^2.1    0.0213  0.1460    216     no  CTmax 
## tau^2.2    0.0524  0.2289      7     no   LT50 
## rho        0.0000                   yes        
## 
## Test for Residual Heterogeneity:
## QE(df = 221) = 8562.9957, p-val < .0001
## 
## Test of Moderators (coefficients 1:2):
## F(df1 = 2, df2 = 221) = 0.6520, p-val = 0.5220
## 
## Model Results:
## 
##              estimate      se    tval   df    pval    ci.lb   ci.ub   ​ 
## metricCTmax    0.0486  0.0982  0.4954  221  0.6208  -0.1449  0.2422    
## metricLT50     0.1877  0.1658  1.1321  221  0.2588  -0.1390  0.5144    
## 
## ---
## Signif. codes:  0 '***' 0.001 '**' 0.01 '*' 0.05 '.' 0.1 ' ' 1  
       mod_results (mod.persistent_metric_het,  mod =   "metric" ,  data =  d.persistent,  group =   "species_ID" )    
  ##    name   estimate    lowerCL   upperCL    lowerPR   upperPR
## 1 CTmax 0.04864284 -0.1448687 0.2421544 -0.4303011 0.5275867
## 2  LT50 0.18768761 -0.1390454 0.5144206 -0.4599910 0.8353662  
       r2_ml (mod.persistent_metric_het)    
  ##    R2_marginal R2_conditional 
##     0.02056887     0.02056887  
       my.orchard (mod.persistent_metric_het,  mod =   "metric" ,  xlab =   "dARR" ,  alpha =   0.1 , 
        data =  d.persistent,  group =   "species_ID" ,  whisker =   0.07 )    
   
 
 
 
 Heating rate 
 
 Run model and plot results 
      mod.persistent_ramping  <-   run.model ( filter (d, brought_common_temp  ==   "yes" ),  ~  scale (ramping, 
        center =  T,  scale =  F)) 
    summary (mod.persistent_ramping)    
  ## 
## Multivariate Meta-Analysis Model (k = 202; method: REML)
## 
##   logLik  Deviance       AIC       BIC      AICc  ​ 
##  27.8184  -55.6369  -45.6369  -29.1453  -45.3276   
## 
## Variance Components:
## 
##             estim    sqrt  nlvls  fixed      factor    R 
## sigma^2.1  0.0018  0.0422     20     no  species_ID   no 
## sigma^2.2  0.0243  0.1558     20     no   phylogeny  yes 
## sigma^2.3  0.0215  0.1465    202     no       es_ID   no 
## 
## Test for Residual Heterogeneity:
## QE(df = 200) = 8200.4819, p-val < .0001
## 
## Test of Moderators (coefficient 2):
## F(df1 = 1, df2 = 200) = 3.4784, p-val = 0.0636
## 
## Model Results:
## 
##                                        estimate      se    tval   df    pval​ 
## intrcpt                                  0.0391  0.0935  0.4179   18  0.6810 
## scale(ramping, center = T, scale = F)    0.1532  0.0821  1.8650  200  0.0636 
##                                          ci.lb   ci.ub 
## intrcpt                                -0.1575  0.2356    
## scale(ramping, center = T, scale = F)  -0.0088  0.3151  . 
## 
## ---
## Signif. codes:  0 '***' 0.001 '**' 0.01 '*' 0.05 '.' 0.1 ' ' 1  
       r2_ml (mod.persistent_ramping)    
  ##    R2_marginal R2_conditional 
##     0.03442667     0.56399312  
      d.persistent_ramping  <-   filter (d, ramping  !=   "NA"   &  brought_common_temp  ==   "yes" ) 
    plot_continuous (d.persistent_ramping, mod.persistent_ramping, d.persistent_ramping $ ramping, 
        "Heating rate (degrees/min)" )    
   
 
 
 
  
 
 
 
  Risk of bias
analysis  
 
 Analysis without imputed values 
      mod.imputed  <-   run.model ( filter (d, imputed  ==   "no" ),  ~  1 ) 
    summary (mod.imputed)    
  ## 
## Multivariate Meta-Analysis Model (k = 1000; method: REML)
## 
##   logLik  Deviance       AIC       BIC      AICc  ​ 
## -81.0743  162.1486  170.1486  189.7756  170.1888   
## 
## Variance Components:
## 
##             estim    sqrt  nlvls  fixed      factor    R 
## sigma^2.1  0.0107  0.1034    133     no  species_ID   no 
## sigma^2.2  0.0223  0.1492    133     no   phylogeny  yes 
## sigma^2.3  0.0416  0.2041   1000     no       es_ID   no 
## 
## Test for Heterogeneity:
## Q(df = 999) = 60216.9037, p-val < .0001
## 
## Model Results:
## 
## estimate      se    tval   df    pval   ci.lb   ci.ub   ​ 
##   0.1873  0.0834  2.2447  132  0.0265  0.0222  0.3524  * 
## 
## ---
## Signif. codes:  0 '***' 0.001 '**' 0.01 '*' 0.05 '.' 0.1 ' ' 1  
       mod_results (mod.imputed,  mod =   "1" ,  data =   filter (d, imputed  ==   "no" ),  group =   "species_ID" )    
  ##      name estimate lowerCL upperCL    lowerPR   upperPR
## 1 Intrcpt        0       0       0 -0.5403307 0.5403307  
       my.orchard (mod.imputed,  xlab =   "dARR" ,  mod =   "1" ,  alpha =   0.1 ,  data =   filter (d, imputed  ==  
        "no" ),  group =   "species_ID" ,  whisker =   0.07 )    
   
 
 
 Analysis without concerning data 
      mod.concern  <-   run.model ( filter (d, is_concern  ==   "no" ),  ~  1 ) 
    summary (mod.concern)    
  ## 
## Multivariate Meta-Analysis Model (k = 852; method: REML)
## 
##   logLik  Deviance       AIC       BIC      AICc  ​ 
## -48.5652   97.1305  105.1305  124.1161  105.1778   
## 
## Variance Components:
## 
##             estim    sqrt  nlvls  fixed      factor    R 
## sigma^2.1  0.0110  0.1047    126     no  species_ID   no 
## sigma^2.2  0.0236  0.1536    126     no   phylogeny  yes 
## sigma^2.3  0.0412  0.2031    852     no       es_ID   no 
## 
## Test for Heterogeneity:
## Q(df = 851) = 54633.5157, p-val < .0001
## 
## Model Results:
## 
## estimate      se    tval   df    pval   ci.lb   ci.ub   ​ 
##   0.1900  0.0866  2.1949  125  0.0300  0.0187  0.3614  * 
## 
## ---
## Signif. codes:  0 '***' 0.001 '**' 0.01 '*' 0.05 '.' 0.1 ' ' 1  
       mod_results (mod.concern,  mod =   "1" ,  data =   filter (d, is_concern  ==   "no" ),  group =   "species_ID" )    
  ##      name estimate lowerCL upperCL    lowerPR   upperPR
## 1 Intrcpt        0       0       0 -0.5448965 0.5448965  
       my.orchard (mod.concern,  xlab =   "dARR" ,  mod =   "1" ,  alpha =   0.1 ,  data =   filter (d, is_concern  ==  
        "no" ),  group =   "species_ID" ,  whisker =   0.07 )    
   
 
 
 Analyses without extreme values of dARR 
 Here, I performed we ran models after excluding values reaching
arbitrary cutoffs: a) all values above 1.25 and below -1.25 b) All
values under -0.5 c) All values under -0.15 (as in Gunderson &
Stillman 2015) d) With all negative values taken as 0 (as in Morley et
al. 2019) 
 While previous syntheses used such methods, it has been argued that
negative responses can be biologically relevant (Terblanche &
Hoffmann, 2020), and we believe it better captures the importance of
measurement error, as well as potential “non-adaptive” responses
ectotherms may express. 
       # Removing values above 1.25 and below -1.25  
   mod.extreme1 .25   <-   run.model ( filter (d, dARR  >   -  1.25   &  dARR  <   1.25 ),  ~  1 ) 
    summary (mod.extreme1 .25 )    
  ## 
## Multivariate Meta-Analysis Model (k = 1079; method: REML)
## 
##   logLik  Deviance       AIC       BIC      AICc  ​ 
##  11.4223  -22.8445  -14.8445    5.0869  -14.8072   
## 
## Variance Components:
## 
##             estim    sqrt  nlvls  fixed      factor    R 
## sigma^2.1  0.0100  0.0999    138     no  species_ID   no 
## sigma^2.2  0.0422  0.2054    138     no   phylogeny  yes 
## sigma^2.3  0.0327  0.1809   1079     no       es_ID   no 
## 
## Test for Heterogeneity:
## Q(df = 1078) = 58411.9685, p-val < .0001
## 
## Model Results:
## 
## estimate      se    tval   df    pval    ci.lb   ci.ub   ​ 
##   0.1953  0.1129  1.7307  137  0.0858  -0.0278  0.4185  . 
## 
## ---
## Signif. codes:  0 '***' 0.001 '**' 0.01 '*' 0.05 '.' 0.1 ' ' 1  
       mod_results (mod.extreme1 .25 ,  mod =   "1" ,  data =   filter (d, dARR  >   -  1.25   &  dARR  <   1.25 ), 
        group =   "species_ID" )    
  ##      name estimate lowerCL upperCL    lowerPR   upperPR
## 1 Intrcpt        0       0       0 -0.5761898 0.5761898  
       my.orchard (mod.extreme1 .25 ,  xlab =   "dARR" ,  mod =   "1" ,  alpha =   0.1 ,  data =   filter (d, 
       dARR  >   -  1.25   &  dARR  <   1.25 ),  group =   "species_ID" ,  whisker =   0.05 )    
   
       ## Removing values under -0.5  
   mod.extreme0 .5   <-   run.model ( filter (d, dARR  >   -  0.5 ),  ~  1 )   # Removed 34 effect sizes  
    summary (mod.extreme0 .5 )    
  ## 
## Multivariate Meta-Analysis Model (k = 1055; method: REML)
## 
##   logLik  Deviance       AIC       BIC      AICc  ​ 
##  45.8412  -91.6824  -83.6824  -63.8410  -83.6442   
## 
## Variance Components:
## 
##             estim    sqrt  nlvls  fixed      factor    R 
## sigma^2.1  0.0085  0.0920    137     no  species_ID   no 
## sigma^2.2  0.1337  0.3656    137     no   phylogeny  yes 
## sigma^2.3  0.0319  0.1786   1055     no       es_ID   no 
## 
## Test for Heterogeneity:
## Q(df = 1054) = 57071.3506, p-val < .0001
## 
## Model Results:
## 
## estimate      se    tval   df    pval    ci.lb   ci.ub   ​ 
##   0.2179  0.1980  1.1005  136  0.2731  -0.1737  0.6095    
## 
## ---
## Signif. codes:  0 '***' 0.001 '**' 0.01 '*' 0.05 '.' 0.1 ' ' 1  
       mod_results (mod.extreme0 .5 ,  mod =   "1" ,  data =   filter (d, dARR  >   -  0.5 ),  group =   "species_ID" )    
  ##      name estimate lowerCL upperCL    lowerPR   upperPR
## 1 Intrcpt        0       0       0 -0.8250144 0.8250144  
       my.orchard (mod.extreme0 .5 ,  xlab =   "dARR" ,  mod =   "1" ,  alpha =   0.1 ,  data =   filter (d, 
       dARR  >   -  0.5 ),  group =   "species_ID" ,  whisker =   0.05 )    
   
       ## Removing values under -0.15 (as in Gunderson & Stillman 2015)  
   mod.extreme0 .15   <-   run.model ( filter (d, dARR  >   -  0.15 ),  ~  1 )   # Removed 97 effect sizes  
    summary (mod.extreme0 .15 )    
  ## 
## Multivariate Meta-Analysis Model (k = 992; method: REML)
## 
##    logLik   Deviance        AIC        BIC       AICc  ​ 
##  126.9958  -253.9916  -245.9916  -226.3968  -245.9510   
## 
## Variance Components:
## 
##             estim    sqrt  nlvls  fixed      factor    R 
## sigma^2.1  0.0086  0.0928    136     no  species_ID   no 
## sigma^2.2  0.1426  0.3776    136     no   phylogeny  yes 
## sigma^2.3  0.0260  0.1611    992     no       es_ID   no 
## 
## Test for Heterogeneity:
## Q(df = 991) = 48345.9519, p-val < .0001
## 
## Model Results:
## 
## estimate      se    tval   df    pval    ci.lb   ci.ub   ​ 
##   0.2364  0.2043  1.1576  135  0.2491  -0.1675  0.6404    
## 
## ---
## Signif. codes:  0 '***' 0.001 '**' 0.01 '*' 0.05 '.' 0.1 ' ' 1  
       mod_results (mod.extreme0 .15 ,  mod =   "1" ,  data =   filter (d, dARR  >   -  0.15 ),  group =   "species_ID" )    
  ##      name estimate lowerCL upperCL    lowerPR   upperPR
## 1 Intrcpt        0       0       0 -0.8323417 0.8323417  
       my.orchard (mod.extreme0 .15 ,  xlab =   "dARR" ,  mod =   "1" ,  alpha =   0.1 ,  data =   filter (d, 
       dARR  >   -  0.15 ),  group =   "species_ID" ,  whisker =   0.05 )    
   
       ## Quantifying negative dARR values as 0 (as in Morley et al. 2019)  
   d  <-  d  %>%  
        mutate ( morley_dARR =   ifelse (dARR  <   0 ,  0 , dARR))   # Convert negative values to 0   
    sum ( with (d, morley_dARR  ==   0 ))   # 233 effect sizes concerned by this change     
  ## [1] 230  
      mod.extreme0  <-   rma.mv ( yi =  morley_dARR,  V =  VCV_dARR,  mods =   ~  1 ,  method =   "REML" , 
        test =   "t" ,  dfs =   "contain" ,  random =   list ( ~  1   |  species_ID,  ~  1   |  phylogeny,  ~  1   |  
           es_ID),  R =   list ( phylogeny =  phylo_matrix),  data =  d,  sparse =   TRUE ) 
    summary (mod.extreme0)    
  ## 
## Multivariate Meta-Analysis Model (k = 1089; method: REML)
## 
##    logLik   Deviance        AIC        BIC       AICc  ​ 
##  128.2016  -256.4033  -248.4033  -228.4349  -248.3664   
## 
## Variance Components:
## 
##             estim    sqrt  nlvls  fixed      factor    R 
## sigma^2.1  0.0081  0.0902    138     no  species_ID   no 
## sigma^2.2  0.0460  0.2144    138     no   phylogeny  yes 
## sigma^2.3  0.0268  0.1637   1089     no       es_ID   no 
## 
## Test for Heterogeneity:
## Q(df = 1088) = 45685.9027, p-val < .0001
## 
## Model Results:
## 
## estimate      se    tval   df    pval    ci.lb   ci.ub   ​ 
##   0.2256  0.1173  1.9227  137  0.0566  -0.0064  0.4575  . 
## 
## ---
## Signif. codes:  0 '***' 0.001 '**' 0.01 '*' 0.05 '.' 0.1 ' ' 1  
       mod_results (mod.extreme0,  mod =   "1" ,  data =  d,  group =   "species_ID" )    
  ##      name estimate lowerCL upperCL    lowerPR   upperPR
## 1 Intrcpt        0       0       0 -0.5623902 0.5623902  
       my.orchard (mod.extreme0,  xlab =   "dARR" ,  mod =   "1" ,  alpha =   0.1 ,  data =  d,  group =   "species_ID" , 
        whisker =   0.05 )    
   
 
 
  
 
 
 
  Comparisons with the data from Morley et al. 2019.
10.1111/geb.12911  
 In our discussion, we discuss the overall levels of plasticity we
report in developing ectotherms, and compare it with the results of
previous syntheses. Here, we provide the code used to calculate the
estimated ARR measured in Morley et al. 2019 
      d.morley  <-   read_csv ( "data/data_Morley_et_al_2019.csv" ) 
    
    # Quick processing  
   d.morley  <-   mutate (d.morley,  habitat =   ifelse (Habitat  ==   "marine"   |  Habitat  ==   "freshwater" , 
        "aquatic" ,  "terrestrial" ))   # merge freshwater and marine into 'aquatic'  
    
   d.morley  <-   filter (d.morley, life_history  ==   "adult" )   # Only keep observations for adults for comparison  
    
    n_distinct (d.morley $ Species)   # 278 species     
  ## [1] 278  
       length (d.morley $ ctmax_ARR[d.morley $ habitat  ==   "aquatic" ])   # 183 effect sizes aquatic     
  ## [1] 183  
       length (d.morley $ ctmax_ARR[d.morley $ habitat  ==   "terrestrial" ])   # 153 effect sizes terrestrial     
  ## [1] 153  
       library (lme4) 
   mod.morley  <-  lmerTest ::  lmer (ctmax_ARR  ~   1   +  ( 1   |  Species)  +  ( 1   |  Source),  data =  d.morley) 
    summary (mod.morley)    
  ## Linear mixed model fit by REML. t-tests use Satterthwaite's method [
## lmerModLmerTest]
## Formula: ctmax_ARR ~ 1 + (1 | Species) + (1 | Source)
##    Data: d.morley
## 
## REML criterion at convergence: -135.8
## 
## Scaled residuals: 
##     Min      1Q  Median      3Q     Max 
## -1.9270 -0.4131 -0.1081  0.2049  3.9181 
## 
## Random effects:
##  Groups   Name        Variance Std.Dev.
##  Species  (Intercept) 0.02558  0.1599  
##  Source   (Intercept) 0.00000  0.0000  
##  Residual             0.01636  0.1279  
## Number of obs: 336, groups:  Species, 278; Source, 2
## 
## Fixed effects:
##              Estimate Std. Error        df t value Pr(>|t|)    
## (Intercept)   0.23544    0.01203 255.96993   19.57   <2e-16 ***
## ---
## Signif. codes:  0 '***' 0.001 '**' 0.01 '*' 0.05 '.' 0.1 ' ' 1
## optimizer (nloptwrap) convergence code: 0 (OK)
## boundary (singular) fit: see help('isSingular')  
       # Remove observations classified as 'zero'  
   d.morley_nozero  <-   filter (d.morley, ctmax_ARR  !=   "0" )   # Remove ARR values of zero; as they don't represent sampled data (all negative values were converted to 0)  
    
   mod.morley_nozero  <-  lmerTest ::  lmer (ctmax_ARR  ~   1   +  ( 1   |  Species)  +  ( 1   |  Source), 
        data =  d.morley_nozero) 
    summary (mod.morley_nozero)    
  ## Linear mixed model fit by REML. t-tests use Satterthwaite's method [
## lmerModLmerTest]
## Formula: ctmax_ARR ~ 1 + (1 | Species) + (1 | Source)
##    Data: d.morley_nozero
## 
## REML criterion at convergence: -146
## 
## Scaled residuals: 
##     Min      1Q  Median      3Q     Max 
## -1.8052 -0.5049 -0.2088  0.3835  4.2889 
## 
## Random effects:
##  Groups   Name        Variance  Std.Dev. 
##  Species  (Intercept) 2.559e-02 0.1599738
##  Source   (Intercept) 3.397e-07 0.0005828
##  Residual             1.319e-02 0.1148649
## Number of obs: 294, groups:  Species, 245; Source, 2
## 
## Fixed effects:
##             Estimate Std. Error       df t value Pr(>|t|)    
## (Intercept)  0.27049    0.01239 35.03003   21.83   <2e-16 ***
## ---
## Signif. codes:  0 '***' 0.001 '**' 0.01 '*' 0.05 '.' 0.1 ' ' 1
## optimizer (nloptwrap) convergence code: 0 (OK)
## Model failed to converge with max|grad| = 0.0246118 (tol = 0.002, component 1)  
 
 
 
  Post-hoc analyses  
 Here, we present analyses that were suggested during peer-review.
Note that we did not have  a priori  hypotheses and predictions
for those analyses. 
 
 Body mass 
 
 Full dataset 
      d.mass  <-   filter (d, body_mass  !=   "NA" ) 
   mod.body_mass  <-   run.model (d.mass,  ~  log (body_mass)) 
    summary (mod.body_mass)   # 494 effect sizes     
  ## 
## Multivariate Meta-Analysis Model (k = 494; method: REML)
## 
##   logLik  Deviance       AIC       BIC      AICc  ​ 
## -80.2948  160.5895  170.5895  191.5819  170.7130   
## 
## Variance Components:
## 
##             estim    sqrt  nlvls  fixed      factor    R 
## sigma^2.1  0.0093  0.0962     67     no  species_ID   no 
## sigma^2.2  0.0013  0.0362     67     no   phylogeny  yes 
## sigma^2.3  0.0562  0.2371    494     no       es_ID   no 
## 
## Test for Residual Heterogeneity:
## QE(df = 492) = 28344.9267, p-val < .0001
## 
## Test of Moderators (coefficient 2):
## F(df1 = 1, df2 = 492) = 3.7683, p-val = 0.0528
## 
## Model Results:
## 
##                 estimate      se    tval   df    pval    ci.lb   ci.ub     ​ 
## intrcpt           0.2144  0.0303  7.0661   65  <.0001   0.1538  0.2750  *** 
## log(body_mass)    0.0122  0.0063  1.9412  492  0.0528  -0.0001  0.0246    . 
## 
## ---
## Signif. codes:  0 '***' 0.001 '**' 0.01 '*' 0.05 '.' 0.1 ' ' 1  
       plot_continuous (d.mass, mod.body_mass,  log (d.mass $ body_mass),  "ln body mass (g)" )    
   
 
 
 Initial effects 
      d.mass.initial  <-   filter (d.mass, brought_common_temp  ==   "no" ) 
   mod.body_mass.initial  <-   run.model (d.mass.initial,  ~  log (body_mass)) 
    summary (mod.body_mass.initial)   # 411 effect sizes     
  ## 
## Multivariate Meta-Analysis Model (k = 411; method: REML)
## 
##   logLik  Deviance       AIC       BIC      AICc  ​ 
## -67.6426  135.2853  145.2853  165.3539  145.4342   
## 
## Variance Components:
## 
##             estim    sqrt  nlvls  fixed      factor    R 
## sigma^2.1  0.0035  0.0592     59     no  species_ID   no 
## sigma^2.2  0.0022  0.0468     59     no   phylogeny  yes 
## sigma^2.3  0.0597  0.2444    411     no       es_ID   no 
## 
## Test for Residual Heterogeneity:
## QE(df = 409) = 21512.7867, p-val < .0001
## 
## Test of Moderators (coefficient 2):
## F(df1 = 1, df2 = 409) = 0.4708, p-val = 0.4930
## 
## Model Results:
##
[truncated: 372,833 more chars]
